# Long lasting effects of perinatal exposure to the Chlorpyrifos pesticide on sleep, breathing, and neuroinflammation in adult mice

**DOI:** 10.1371/journal.pone.0328581

**Published:** 2025-08-01

**Authors:** Elena Miglioranza, Laura Rullo, Sara Alvente, Stefano Bastianini, Dario Coraci, Viviana Lo Martire, Loredana Maria Losapio, Gabriele Matteoli, Camilla Morosini, Emilia Volino, Alessandro Silvani, Roberto Rimondini, Sanzio Candeletti, Patrizia Romualdi, Giovanna Zoccoli, Chiara Berteotti

**Affiliations:** 1 PRISM Lab, Department of Biomedical and Neuromotor Sciences, and Center for Applied Biomedical Research, Department of Medical and Surgical Sciences, S. Orsola University Hospital, Alma Mater Studiorum - University of Bologna, Bologna, Italy; 2 Department of Pharmacy and Biotechnology, Alma Mater Studiorum, University of Bologna, Bologna, Italy; 3 Department of Medical and Surgical Sciences, Alma Mater Studiorum, University of Bologna, Bologna, Italy; University of North Dakota, UNITED STATES OF AMERICA

## Abstract

Early-life exposure to environmental stressors may increase the risk of disease later in life. Chlorpyrifos (CPF), a widely used pesticide and acetylcholinesterase inhibitor, can cross the placental barrier and can be found in breast milk, leading to excessive cholinergic stimulation. Acetylcholine is involved in sleep and respiratory regulation. The main objective of this study was to investigate whether perinatal CPF exposure affects sleep-related breathing together with promotion of neuroinflammatory processes in adulthood. To explore these effects, CPF (5 mg/kg/day) or vehicle was administered orally to dams from mating to weaning. The offspring were not directly treated. At 17–18 weeks of age, male and female offspring underwent electroencephalographic and electromyographic electrode implantation to monitor sleep-wake cycles. Recordings were conducted over 48 hours in home cages, and for 7 hours in a plethysmographic chamber to assess sleep-related breathing pattern. At the end of recordings, hippocampal tissues were collected for gene expression analysis via real-time PCR. Results revealed that CPF perinatal exposure increased sighs and apneas during sleep in adult mice, especially in female. Additionally, expression of pro-inflammatory cytokines was upregulated while expression of peroxisome proliferator-activated receptor genes was downregulated in the hippocampus of female mice born to CPF-treated dams. These findings suggest that perinatal CPF exposure can induce long-lasting alterations in sleep-related respiratory patterns and hippocampal inflammatory responses, with a sex-specific susceptibility—females being more affected. This highlights the perinatal period as a critical window of vulnerability to environmental toxicants such as pesticides. The results support the hypothesis that adult sleep and brain inflammation phenotypes may be modulated by early-life chemical exposures during pregnancy and lactation.

## Introduction

Exposure to stressors during critical developmental periods can induce long-lasting biological adaptations that may enhance early survival but increase susceptibility to disease in adulthood [1]. Perinatal stressors include malnutrition, lack of maternal care [2,3], and exposure to substances of abuse (opioids, ethanol, nicotine) or toxic chemicals such as pesticides [1,4,5].

Organophosphate pesticides (OPs) are widely used in agricultural, industrial, and domestic settings [6] and represent the largest pesticide category used in the world and the most widespread due to their bioaccumulation in the environment [7–9]. OPs are important indoor and outdoor environment and food contaminants [10,11]. In adults and children, exposure to OPs mainly occurs via dietary intake [12,13]. Due to their highly lipophilic nature, OPs can also pass through the placenta to the foetus and may affect its development [14]. In this respect, various metabolites of OPs have been found in cord blood and in meconium samples of newborns from women exposed to OPs during pregnancy [15,16]. Newborns can be further exposed to these pesticides after birth via breastfeeding [17,18].

Chlorpyrifos (CPF, O,O-diethyl-O-[3,5,6-trichloro-2-pyridinyl] phosphorothioate) has been one of the top-selling, widely used OPs for decades. Despite restrictions limiting its employment in commercial and household settings, CPF still remains a commonly used insecticide and continues to represent a potential risk for people living in agricultural communities [19,20]. In humans, CPF is metabolized to CPF-oxon, an active and potent anti-cholinesterase inhibitor, resulting in the accumulation of acetylcholine at cholinergic synapses and consequently in synapse overstimulation in the central and peripheral nervous systems [21].

Since acetylcholine is a fundamental neurotransmitter in sleep and respiratory control, CPF exposure causing cholinergic hyperstimulation might mimic hyperactivity of the brain cholinergic systems that are involved in these physiological processes. This may contribute to explain why chronic pre- and postnatal exposure to CPF, from the first day of gestation and through postnatal life until adulthood, was linked to an increase in sleep apnoea index and augmented the contractility of the respiratory skeletal muscles in sleeping juvenile and adult rats [22]. Additionally, acute or chronic exposure to organophosphates such as ethion has been shown to induce lung inflammation and genotoxicity in mice, especially in synergy with endotoxins [23,24]. An association between OP exposure (including prenatal exposure) and short sleep duration was reported in adolescents and adults [25–27]. Prenatal CPF exposure has been recently linked to epigenetic modifications, including histone and DNA methylation [28]. However, the long-term consequences of isolated perinatal (i.e., from gestation to the end of lactation) CPF exposure remain unclear. Furthermore, excessive activation of the cholinergic system may induce an activation of stress-related behaviors [29]. Supporting this, exposure to CPF and dichlorvos, both organophosphate compounds, has been shown to trigger oxidative and neurogenic damage leading to anxiety-like behavior in rats [30].

Glucocorticoid overexposure has been reported among the mechanisms linking early-life stress with diseases later in life [31]. Cortisol levels naturally rise in the maternal circulation during late gestation, but fetal exposure is normally restricted by the action of the placental glucocorticoid-inactivating enzyme 11β-hydroxysteroid dehydrogenase [32]. Maternal stress, however, can impair this protective mechanism [33], resulting in excessive glucocorticoid transfer to the fetus, which can affect the responsiveness of the hormonal stress response throughout life. Among brain regions, the hippocampus is especially sensitive to early-life stress due to its pronounced postnatal development [34], high degree of plasticity [1], and abundance of corticosteroid receptors [35]. Under normal conditions, the hippocampus acts to suppress hypothalamic-pituitary-adrenal (HPA) axis activity [35] by reducing corticotropin-releasing hormone (CRH) output from the hypothalamus, thereby limiting the release of cortisol in humans and corticosterone in rodents [35]. This regulatory mechanism involves a feedback loop mediated by two corticosteroid receptors in the hippocampus—mineralocorticoid and glucocorticoid receptors—that sense circulating cortisol levels and modulate hormonal output accordingly [36]. Changes in the expression of these receptors are a potential mechanism whereby perinatal exposure to OPs, such as CPF, modify stress responsiveness in adult life.

Furthermore, it has been suggested that environmental factors, including the exposure to agrochemicals such as OPs, may contribute to the development of neurodegenerative diseases by promoting oxidative stress and neuroinflammatory processes [23,24,30,37,38]. In this respect, besides the well-known relevance of proinflammatory cytokines (IL-6, IL-1β, and TNF-α), particular interest has been recently focused on the role of peroxisome proliferator-activated receptors (PPARs) in the modulation of inflammatory gene expression and immune cell function likely related to the development of neurological diseases [39]. The aim of this study was to investigate the consequences of isolated perinatal CPF exposure on 1) sleep macrostructure, 2) breathing during sleep and 3) anxiety and cognitive behaviour in adult male and female mice. In addition, considering the above reported links between CPF exposure and epigenetic modifications and neuroinflammation, we explored the gene expression alterations of histone demethylase enzymes. These epigenetic enzymes are involved in the removal of trimethyl marks at lysine 4 (KDM5C) and lysine 27 (KDM6A and KDM6B) residues of H3, which can respectively silence or enhance the expression of specific genes. In particular, the KDM6 enzymes have been shown to drive inflammatory processes by promoting interleukin (IL) expression and repressing PPARγ expression and activity [40,41]. Moreover, CPF ability to induce long-term alterations in the expression of the pro-inflammatory cytokines IL-6, IL-1β, and TNF-α and of nuclear receptors PPARα and PPARγ has been also investigated in the hippocampus of male and female adult mice.

In addition, neuroinflammation is primarily involved in the pathogenesis of chronic neuropathic pain [42] that affects about 20% of the total population and is accompanied by spontaneous pain, hyperalgesia, and mechanical allodynia [43]. In this context CPF perinatal exposure could affect pain sensitivity in adult mice by favoring neuroinflammation [44]. Finally, the potential late dysregulation of the stress response caused by the isolated perinatal CPF exposure was investigated by evaluating plasma corticosterone levels and glucocorticoid receptor (Nr3c1) expression in the hippocampus of adult mice.

## Materials and methods

### Ethical approval

The study protocol complied with the EU Directive 2010/63/EU for animal experiments and was approved by the Committee on the Ethics of Animal Experiments of the University of Bologna (Prot. n. AEDB0.23) and the Italian Ministry of Health (Prot. n. 298/2021_PR). The experiments were carried out according to the guidelines of the animal welfare committee of the University of Bologna and ARRIVE guidelines. Surgery was performed under deep anesthesia, and all efforts were made to minimize suffering.

### Mice and drugs

Experiments were performed on wild-type C57BL/6 mice. Mice were maintained at 23 °C with a 12:12 h light–dark cycle and lights on at 9 am (i.e., Zeitgeber Time 0, ZT0) and free access to water and food (4RF21 diet, Mucedola, Settimo Milanese, Italy). The mouse colony was maintained at the Department of Biomedical and Neuromotor Sciences, University of Bologna. Mice were housed in standard cages with floor area of 530 cm^2^ or 820 cm^2^ (Cat. No 1284L and 1290D by Tecniplast, Varese, Italy), depending on the number of mice per cage, and with specific wood particles as bedding (Scobis One, Mucedola, Settimo Milanese, Italy). Breeding was achieved with a harem mating scheme. The harem was maintained until the weaning of pups was completed. Dams were administered CPF (Pestanal, Sigma-Aldrich, St. Louis, USA) at a dose of 5 mg/kg/day in peanut oil (N = 14) or vehicle (N = 10) by intraoral gavage from mating until weaning using a polypropylene 20G needle (FTP 20-38, Instech, Plymouth Meeting, USA). The dose of 5 mg/kg/day of CPF is above the no observed adverse effect level (NOAEL, i.e., the maximum dose of a compound that can be administered to an individual without causing harmful effects) for developmental toxicity [45]. Pups were never directly treated with CPF nor with vehicle. The number of pups delivered from CPF- or vehicle-treated dams was not significantly different nor was the average weight of the litters at birth (data not shown). After weaning, pups were kept under a 12:12 h light–dark cycle at 25 °C with free access to water and food (4RF21 diet; Mucedola, Milano, Italy) and with lights on at ZT0. At the end of weaning, dams were sacrificed for plasma assessment of acetylcholine esterase (AChE) activity, as a marker of the treatment efficacy. Adult mice, both male and female, born to CPF-treated dams (TRM and TRF, respectively) and to vehicle-treated control dams (CLM and CLF, respectively) were included in the present study.

### AChE activity

AChE activity was measured in plasma samples of a subset of CPF-treated and vehicle-treated dams (N = 6 and N = 5, respectively) according to Ellman’s colorimetric method [46]. The colorimetric assay was run according to the manufacturer’s instructions (AB138871, AbCam, Cambridge, UK) using a microplate reader (Spark, Tecan, Switzerland). Plasma samples were diluted 1:200 in distilled water and pre-incubated with the butyrylcholinesterase inhibitor Donepezil hydrochloride (D6821, Sigma-Aldrich, St. Louis, USA) to prevent false positive results. Readings were performed in triplicate. AChE standards (3, 10, 30, 100, 200, 300, and 1000 mU/ml) were read in duplicate to obtain an absorbance/concentration curve. Data were analysed by linear regression on a logarithmic scale and expressed in mU/ml.

### Overview of the experimental protocol

**Fig 1:**
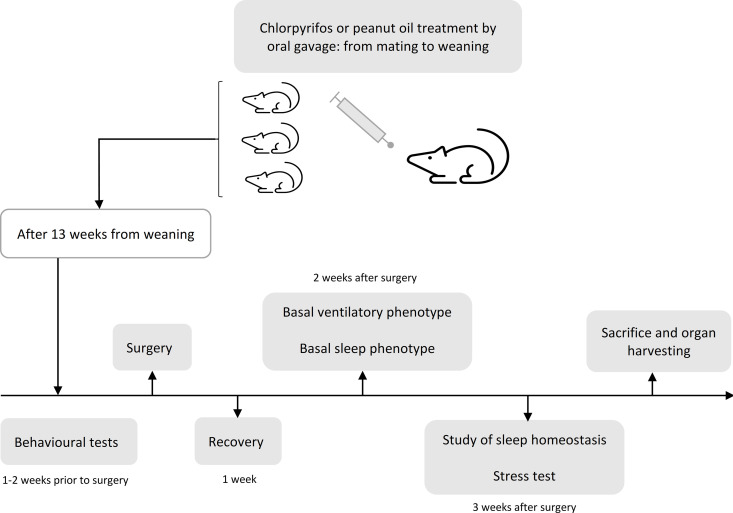
Summary of the research protocol.

The overview of the research protocol is reported in Fig 1. Seven to 10 days before mice underwent surgery, a battery of behavioural tests was performed in accordance with validated protocols (see supplementary materials). Surgery was then conducted to implant electroencephalographic (EEG) and neck electromyographic (EMG) electrodes to monitor the wake-sleep cycle. After surgery, mice were housed individually and allowed to recover for 7 days. Then, mice were briefly anesthetized (1.8–2.4% isoflurane in O_2_) to connect the previously implanted electrodes to a recording cable for habituation to the recording apparatus. The cable, in turn, was plugged to a rotating swivel (SL2 + 2C/SB, Plastics One, Roanoke, VA, USA) and a balanced cable suspensor arm, allowing unhindered movements to the mice. After 4–7 days of habituation to the recording setup, simultaneous sleep and breathing recordings were performed inside a whole-body plethysmograph for 8 h starting from ZT0. After further 2–5 days, baseline sleep recordings were performed for 48 h starting at ZT0, on animals undisturbed in their own cages, as previously described [47]. Then, mice underwent 6 h of sleep deprivation, starting at ZT0 till ZT6, as previously described [47,48], followed by 18 h of sleep recovery. Sleep deprivation was performed by gentle handling by experimenters checking the EEG and EMG signals of mice in real-time. After sleep recovery, blood from the tail vein was collected between ZT0 and ZT1 under light anaesthesia, and then all mice were restrained for the duration (2 h) of the stress protocol [49]. In particular, mice were restrained in well-ventilated 50-ml tubes, with 3- or 4-cm long additional tubes slipped over the tail to completely restrict movement [50] and left undisturbed under an opaque box. After the restraint period, mice were euthanized (isoflurane at 4% in O_2_) between ZT2 and ZT3. Blood from the carotid artery was collected and the hippocampi were dissected and immediately frozen and stored at −80 °C for subsequent gene expression analysis.

The oestrous phase of female mice was evaluated after behavioural tests, before whole-body plethysmograph recordings, after baseline recordings and at sacrifice.

### Behavioural assays

Behavioural assays included: a pain-related behaviour test (for evaluation of mechanical allodynia), Y-maze spontaneous alternation, elevated plus maze (EPM), novel object recognition task (NOR) and open-field (OF) test (see supplementary materials for details). All animal behavioural studies and analyses were performed blinded to treatment and sex. Mice were accommodated in the animal facility where tests had to be performed the day before and were allowed to habituate to the testing room for at least 1–2 h before the test, which was scheduled and performed between ZT0 and ZT8. All experiments were recorded and scored with a video tracking system (Ethovision 15.0; Noldus Information Technology B.V., Wageningen, Netherlands). Behavioural assays were performed on adult mice (TRM, N = 16–18; TRF, N = 12–15; CLM, N = 7–9; CLF, N = 15–17).

### Surgery

Adult mice underwent surgery for the implantation of electrodes for wake-sleep state discrimination. For the differential EEG signal detection, two stainless-steel screws (2.4 mm length and 1.19 mm diameter; model 00-96x3/32, Plastics One, Roanoke, VA, USA) were implanted into ipsilateral frontal and parietal bones through burr holes and positioned in contact with the *dura mater*. For the differential EMG signal recording, two stainless-steel wires (A-M Systems, WA, USA) were implanted bilaterally into the neck muscles. All electrodes were connected to miniature custom-built sockets (267–7400 RS Components, Cinisello Balsamo, Italy), which were cemented to the skull with dental cement (RelyX™ Unicem, 3M™ ESPE™, Segrate, Italy) and dental acrylic (DuraLay, Reliance Dental Manufacturing LLC, IL, USA). At the end of the surgery, mice were administered benzathine benzylpenicillin (3750 UI/mouse) and dihydrostreptomycin sulphate (1.5 mg/mouse) in 0.8 mL sterile saline subcutaneously to prevent infections and dehydration. All surgical procedures were performed under isoflurane anaesthesia (1.8–2.4% in O_2_, inhalation route) with intra-operative analgesia (Carprofen 0.1 mg subcutaneously, Zoetis, Rome, Italy) on a heating pad to prevent hypothermia.

### Plasma corticosterone levels

Blood was collected in heparin-coated tubes and centrifuged for 10 minutes at 2500 g and 4 °C. Plasma was separated from the pellet and stored at −20 °C until the analysis was performed. Plasma corticosterone levels were quantified using a competitive enzyme-linked immunosorbent assay (ELISA) kit (EIACORT, Life Technologies, Segrate, Milano).

Plasma corticosterone levels were evaluated before (T0) and after (T1) the restraint stress test. Samples were run in triplicate and compared to a standard curve according to kit instructions. Two samples (one from a female mouse born to a vehicle-treated dam and one from a female mouse born to a CPF-treated dam) were used as inter-plate controls. Absorbance was quantified using a plate reader (Spark, Tecan, Switzerland). The analysis was restricted to a subset of female mice.

### Data acquisition

To characterize the respiratory phenotype during sleep, EEG, EMG and breathing of freely-behaving mice were recorded continuously and simultaneously inside a modified 2-chamber whole-body plethysmograph (PLY4223, Buxco, Wilmington, NC, USA). The mouse chamber (volume 0.97 L) of the plethysmograph accommodated a rotating electrical swivel (SL6C/SB, Plastics One, Roanoke, VA, USA) and probes to measure temperature and humidity (PC52-4-SX-T3 sensor, Rense Instruments, Rowley, MA, USA). The differential pressure between the mouse and control chambers of the plethysmograph was measured with a high-precision pressure transducer (DP103-06, Validyne Engineering, Northridge, CA, USA). The chambers were continuously purged with air at a relatively high flow rate (1.5 L/min) to prevent CO_2_ build-up in the mouse chamber and avoid drifts in the differential pressure between the chambers. The system was calibrated dynamically with a 100 µL micro-syringe (Hamilton, Reno, NV, USA) at the end of each recording [51]. The EEG and EMG signals were transmitted via a cable connected to a rotating swivel (SL2 + 2C/ SB, Plastics One), amplified and filtered (EEG: 0.3–100 Hz with a 50-Hz notch filter; EMG: 100–1000 Hz with a 50-Hz notch filter; 7P511J amplifiers, Grass, West Warwick, RI, USA), digitized at 16-bit and 1024 Hz, and down-sampled at 128 Hz for data storage. Data acquisition was performed by means of custom software written in Labview (National Instruments, Austin, TX, USA).

### Oestrous stage evaluation

Female mice of each group underwent vaginal lavage and cytology evaluation to discriminate the oestrus cycle phase following published criteria [52] as previously described [53]. Briefly, the identification of the oestrous cycle stage is based on the proportion of cell types observed in the cytological sample. In pro-oestrus, there are mostly nucleated epithelial cells, few cornified epithelial cells, and possibly very few leukocytes; in oestrus, there are mostly cornified epithelial cells and possibly few nucleated epithelial cells; in metoestrus, there are mostly cornified epithelial cells, leukocytes in variable proportions, and possibly few nucleated epithelial cells; in dioestrus, there are leukocytes and nucleated epithelial cells in variable proportions and possibly few cornified epithelial cells [52]. The oestrus cycle stage evaluation of each female mouse was reported in S1 Table.

### Data analysis

Wakefulness, non-rapid-eye-movement sleep (NREMS), and rapid-eye-movement sleep (REMS) were automatically scored on the basis of raw EEG and EMG signals (4 s epochs) using SCOPRISM, a validated algorithm [54]. In 4 mice, due to artifacts in EEG and/or EMG recordings in the whole-body plethysmograph, wakefulness, NREMS and REMS inside the plethysmograph were scored by trained investigators based on inspection of the raw respiratory recordings with a validated procedure [51]. The analysis of sleep architecture was performed with a threshold of 12 s (i.e., 3 consecutive 4 s epochs) for wake-sleep episode duration [55].

Spectral analysis of the EEG signal during home-cage and post-deprivation recordings was performed on artefact-free 4 s epochs using the discrete Fourier transform. The EEG power spectrum in NREMS and REMS was expressed as the percentage of total EEG power in each state [47]. EEG spectral power in the delta frequency range (1–4 Hz: slow-wave activity, SWA) during NREMS at baseline and during sleep recovery was expressed as a percentage of the mean SWA during NREMS at baseline between ZT0 and ZT4. To increase the robustness of the estimates, SWA values were calculated on 2-h temporal bins [47].

Quantitative analysis of breathing during the wake-sleep cycle was restricted to stable sleep episodes because of the frequent occurrence of movement artefacts during wakefulness [56,57]. Breath-to-breath values of ventilatory period, tidal volume and minute ventilation were obtained as previously reported for each sleep state [51]. The mean values of these variables were computed for each mouse after exclusion of the breaths with ventilatory period and/or tidal volume that deviated more than 3 standard deviations from their respective mean values in the whole recording [56].These computations were thus protected from the effects of breaths with extreme values of ventilatory period and/or tidal volume. Finally, missed breaths (apnoeas) and augmented breaths (sighs) were automatically detected as breaths with values of ventilatory period (apnoeas) or tidal volume (sighs) more than three times the average values of ventilatory period or tidal volume, respectively, for each mouse and sleep state, and detection accuracy was checked by trained experimenters on raw recordings [56,57]. Because augmented breaths (sighs) often precede apnoeas during NREMS, we further categorized NREMS apnoeas as post-sigh apnoeas if they followed a sigh by ≤ 8 s or as spontaneous apnoeas if they followed a sigh by > 8 s [58].

### Gene expression analysis by real-time PCR

Total RNA was extracted from hippocampus tissue according to the method of Chomczynski and Sacchi [59] and quantified using a NanoDrop 1000 system spectrophotometer (Thermo Fisher Scientific, Waltham, Massachusetts, USA). Each hippocampal sample (N = 6/8 per group) was subjected to DNase treatment and converted to cDNA as previously described [41,47]. Quantitative real-time PCR analysis was performed on a StepOne Real-Time PCR System (Life Technologies, Monza, Italy) using the SYBR® Green PCR MasterMix (Life Technologies). The relative gene expression of different gene transcripts was calculated by the Delta-Delta Ct (ΔΔCt) method and converted to relative expression ratio (2^−ΔΔCt^) for statistical analysis [60]. All data were normalized to the housekeeping gene glyceraldehyde-3-phosphate dehydrogenase (*Gapdh*). The primers used for PCR amplification are reported in S2 Table.

### Statistics

Data were tested for normal distribution using the Shapiro-Wilk test. When the normality assumption was rejected, we first analysed data with the Kruskal-Wallis test and, in case the null hypothesis was rejected, we applied the Mann-Whitney test with the false-discovery rate correction [61] for the following pre-planned comparisons: CLM vs. TRM, CLF vs. TRF, and treated (TRM and TRF) vs. untreated (CLM and CLF) mice. When the normality assumption was not rejected, we performed two-way ANOVAs with treatment (2 levels) and sex (2 levels) as fixed factors. Simple effects were tested with Tukey’s correction for multiple comparisons in case of significant interactions between factors. Data were analysed with GraphPad Prism (version 8.0.2) and are reported as mean ± SEM or as median and range, depending on whether normality was assumed or not. Maternal AChE data were analysed using an unpaired t-test.

Significance was set at P < 0.05.

## Results

### Maternal AChE activity

**Fig 2:**
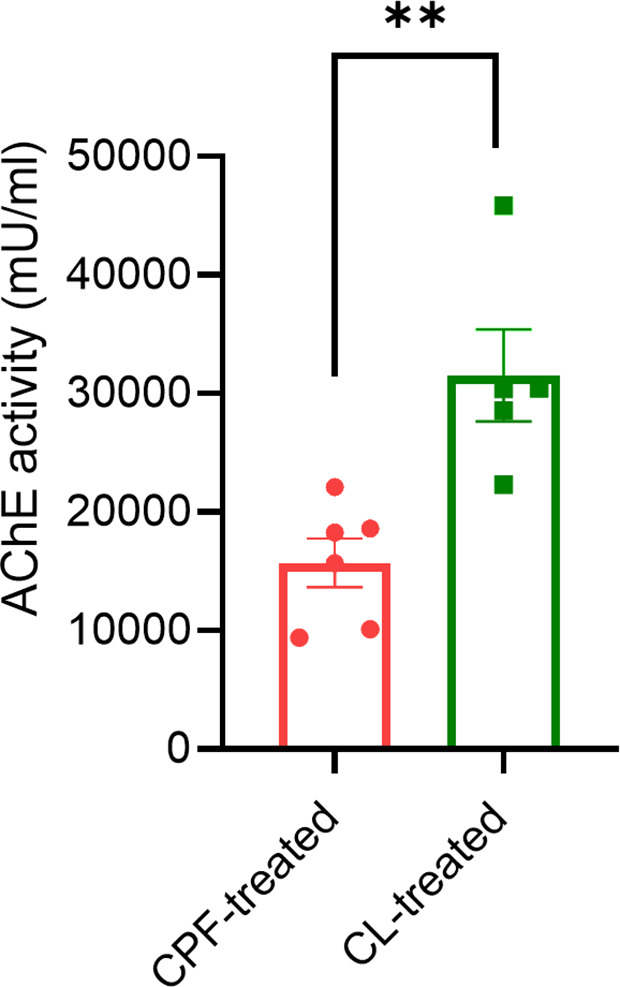
Maternal acetylcholine esterase activity after Chlorpyrifos exposure. The graph shows acetylcholine esterase (AChE) plasma activity in Chlorpyrifos (CPF)-treated dams (N = 6, red dots) compared to vehicle (CL)-treated control dams (N = 5, green squares). **, P < 0.005 CPF-treated dams vs. CL-treated dams (unpaired t-test). Bars show mean ± SEM. Dots and squares show values in individual mice.

The enzymatic activity of AChE was significantly lower in CPF-treated dams than in vehicle-treated dams (N = 6 and N = 5, respectively; P = 0.0043, unpaired t-test) (Fig 2).

### Behavioural tests

#### Mechanical allodynia.

Paw withdrawal latency and paw withdrawal threshold did not differ significantly among groups (P = 0.2158 and P = 0.2470, respectively, Kruskal-Wallis tests, S3 Table).

#### Y-maze.

**Fig 3:**
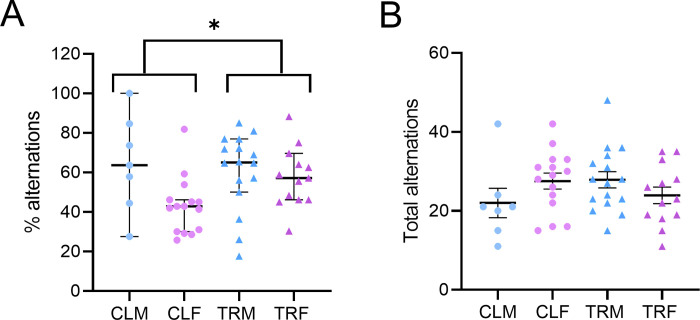
Y-maze test. Panels A and B respectively show the percentage of alternations and the total alternations in the Y-maze test by male and female mice born to vehicle-treated dams (CLM and CLF, respectively) or by male and female mice born to Chlorpyrifos-treated dams (TRM and TRF, respectively). Data are reported as median (range) in panel A, and as mean ± SEM in panel B. * indicates P < 0.05 for the post-hoc corrected comparison between mice born to vehicle-treated dams (CLM and CLF) and mice born to Chlorpyrifos-treated dams (TRM and TRF) after significant Kruskal-Wallis test. Dots and squares show values in individual mice.

The percentage of alternations differed significantly among groups (P = 0.0141, Kruskal-Wallis test) and was higher in mice born to CPF-treated dams (i.e., TRM and TRF) than in mice born to vehicle-treated dams (i.e., CLM and CLF; P = 0.0289, post-hoc corrected comparison, Fig 3, Panel A). The total number of alternations did not show significant differences at ANOVA (P > 0.1350, Fig 3, Panel B).

#### EPM.

**Fig 4:**
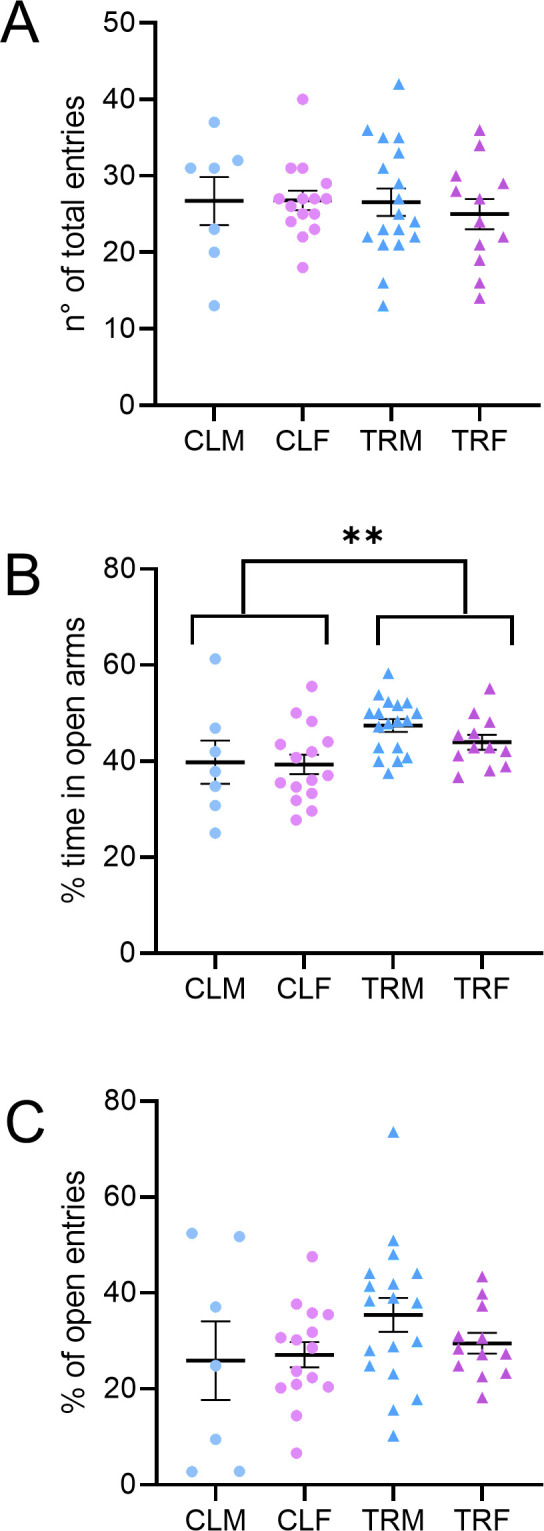
Elevated plus maze test. Panels A, B, and C respectively show the number of total entries in the open arms, the percentage of time spent in the open arms, and the percentage of open arm entries of the elevated plus maze by male and female mice born to vehicle-treated dams (CLM and CLF, respectively) or by male and female mice born to Chlorpyrifos-treated dams (TRM and TRF, respectively). ** indicates P < 0.01, main effect of treatment (two-way ANOVA). Data are reported as mean ± SEM. Dots and triangles show values in individual mice.

The total number of entries and the percentage of open-arm entries did not show significant differences among groups at ANOVA (P > 0.1401, Fig 4, Panels A and C). However, mice born to CPF-treated dams (i.e., TRM and TRF) spent more time in the open arms than mice born to vehicle-treated dams (i.e., CLM and CLF; P = 0.0065, ANOVA main effect, Fig 4, Panel B).

#### OF.

**Fig 5:**
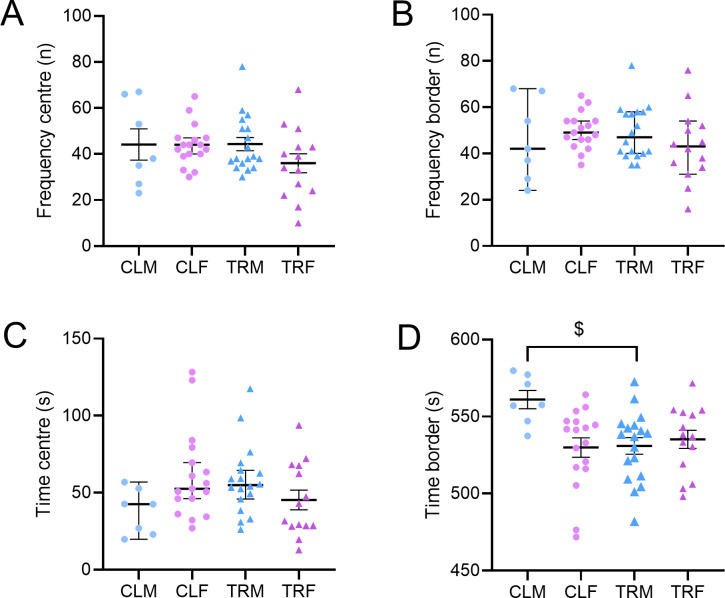
Open-field test. Panels A, B, C, and D respectively show the number of entries in the centre, the number of entries at the border, the time spent in the centre, and the time spent at the border by male and female mice born to vehicle-treated dams (CLM and CLF, respectively) or by male and female mice born to Chlorpyrifos-treated dams (TRM and TRF, respectively). In panels A, B, and C, data are reported as median (range), while in panel D, data are reported as mean ± SEM. $ indicates P < 0.05, Tukey’s multiple comparisons test after two-way ANOVA. Dots and triangles show values in individual mice.

Neither the number of entries in the centre or at the border of the open field nor the time spent in the centre of the open field differed significantly among groups (P > 0.0648, Kruskal-Wallis tests, Fig 5, Panels A-C). Instead, the time spent at the border of the open field showed a significant treatment x sex interaction at ANOVA (P = 0.0095) and was significantly lower in TRM than in CLM (P = 0.0241, Tukey’s multiple comparisons test, Fig 5, panel D).

#### NOR.

The novel object recognition index did not differ significantly among groups (P = 0.9676, Kruskal-Wallis test, S4 Table).

### Body weight and age at surgery

Body weight at surgery differed significantly among groups (P < 0.0001, Kruskal-Wallis test) as a result of higher values in male mice, without any significant simple effect due to treatment (P > 0.7385, post-hoc corrected comparisons). The age of the mice at surgery did not differ among groups (17.6 ± 0.5 weeks for CLM; 18.0 ± 0.2 weeks for CLF; 17.3 ± 0.4 weeks for TRM and 17.9 ± 0.2 weeks for TRF; P > 0.1445, ANOVA).

### Breathing during sleep

**Fig 6:**
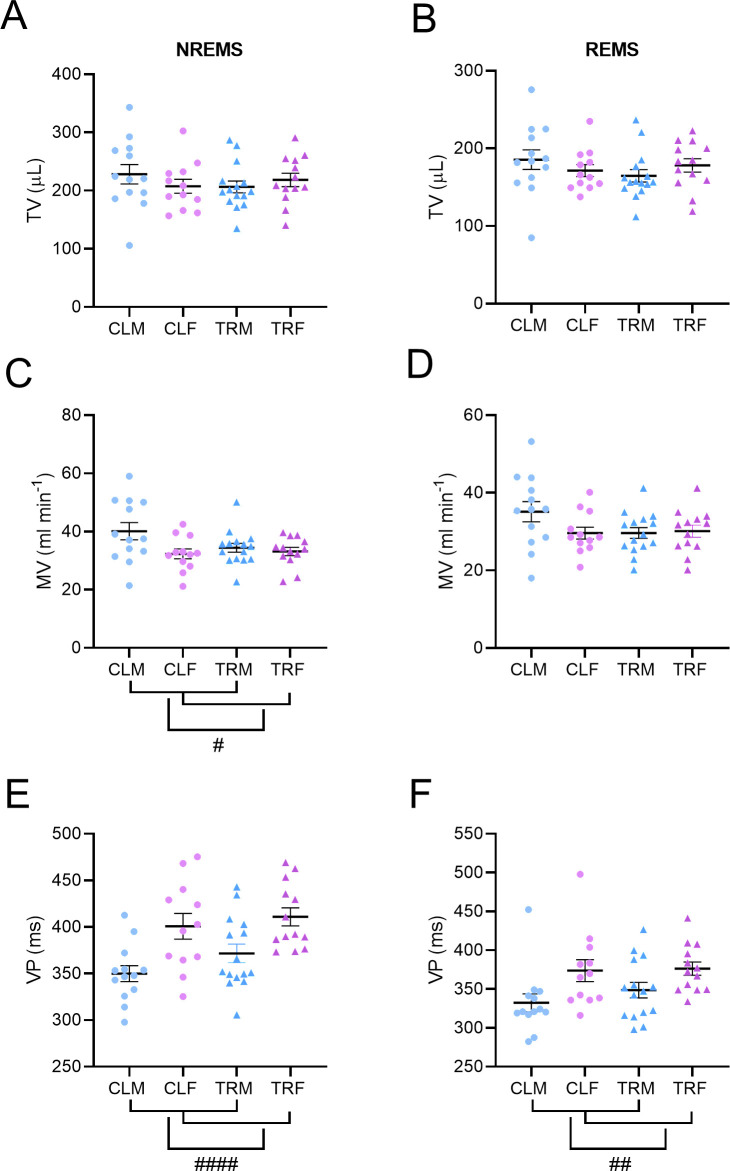
Sleep-related breathing phenotype. Panels A and B respectively show the tidal volume (TV) during non-rapid-eye-movement sleep (NREMS) and rapid-eye-movement sleep (REMS) of male and female mice born to vehicle-treated dams (CLM and CLF, respectively) or to Chlorpyrifos-treated dams (TRM and TRF, respectively). Panels C and D respectively show the minute ventilation (MV) during NREMS and REMS. Panel E and F respectively show the ventilatory period (VP) during NREMS and REMS. #, ##, and #### indicate P < 0.05, P < 0.005, and P < 0.0001, respectively, for the main effect of sex, two-way ANOVA. Data are reported as mean ± SEM. Dots and triangles show values in individual mice.

Tidal volume did not differ significantly among groups either in NREMS or in REMS (P > 0.1519, ANOVA; Fig 6, Panels A, B). The minute ventilation was significantly higher in male mice than in female mice (P = 0.0277, ANOVA main effect) in NREMS, with no significant effect of treatment or sex x treatment interaction (P > 0.1103; Fig 6, Panel C). No differences were found in minute ventilation in REMS among groups (P > 0.1078, ANOVA; Fig 6, Panel D).

The ventilatory period was significantly higher in female mice than in male mice both in NREMS and in REMS (P < 0.0001 and P = 0.0031, respectively, ANOVA main effect) without significant effects of treatment or sex x treatment interactions (P > 0.1378, Fig 6, Panels E, F).

**Fig 7:**
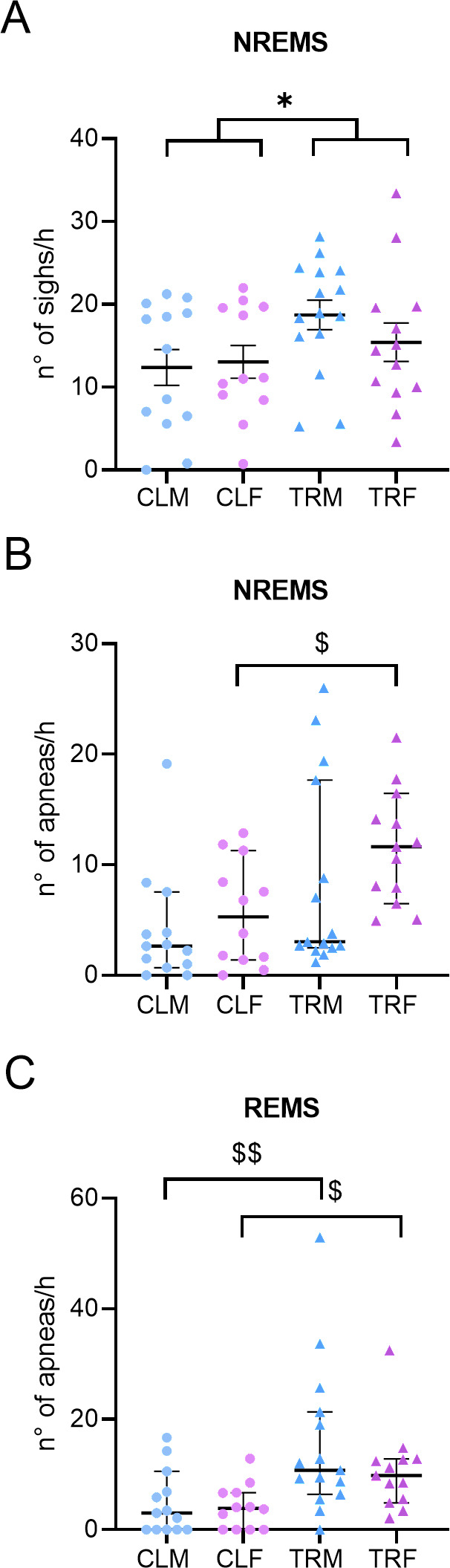
Sighs and sleep apnoeas. Panel A shows sighs (augmented breaths) occurrence rate during non-rapid-eye-movement sleep (NREMS) and rapid-eye-movement sleep (REMS) exhibited by male and female mice born to vehicle-treated dams (CLM and CLF, respectively) or to Chlorpyrifos-treated dams (TRM and TRF, respectively). Panels B and C show sleep apnoeas (breathing pauses) occurrence rate during NREMS and REMS, respectively. Data are reported as mean ± SEM in panel A and as median (range) in panels B and C. Dots and triangles show values in individual mice. Panel A: * indicates P < 0.05 for the main effect of treatment of two-way ANOVA. Panels B and C: $ and $$ indicate P < 0.05 and P < 0.005, respectively, for the post-hoc corrected comparison between CLF and TRF and between CLM and TRM after significant Kruskal-Wallis test.

The number of sighs per h of NREMS was higher in mice born to CPF-treated dams (i.e., TRM and TRF) than in mice born to vehicle-treated dams (i.e., CLM and CLF; P = 0.0407, ANOVA main effect), with no significant effects of sex or sex x treatment interactions (P > 0.3404; Fig 7, Panel A).

The apnoea occurrence rate differed significantly among groups during NREMS and REMS (P = 0.0059 and P = 0.0030, respectively, Kruskal-Wallis tests). The apnoea occurrence rate during NREMS and REMS was significantly higher in TRF than in CLF (P = 0.0125 and P = 0.0215, respectively, post-hoc corrected comparisons, Fig 7, Panels B and C). The apnoea occurrence rate during REMS was also significantly higher in TRM than in CLM (P = 0.004, post-hoc corrected comparison, Fig 7, Panel C).

### Sleep architecture during baseline home-cage recordings

**Fig 8:**
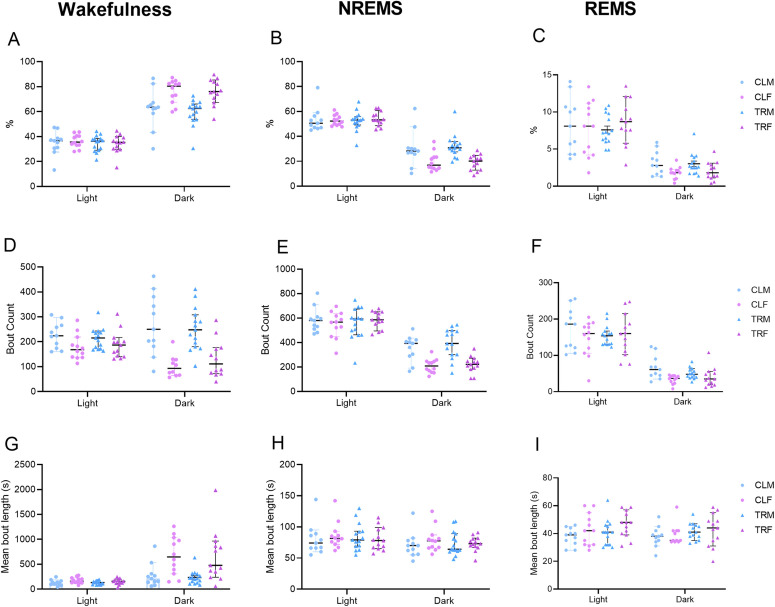
Wake-sleep cycle characterization. Panels A, B and C show the percentage of time spent in wakefulness, non-rapid-eye-movement sleep (NREMS), and rapid-eye-movement sleep (REMS), respectively, split for the light (resting) and the dark (active) period by male and female mice born to vehicle-treated dams (CLM and CLF, respectively) or to Chlorpyrifos-treated dams (TRM and TRF, respectively). Panels D, E and F show the number of episodes during wakefulness, NREMS, and REMS in the light and dark periods. Panels G, H, and I report the mean episode duration during wakefulness, NREMS, and REMS in the light and dark periods. Data are reported as median (range). Dots and triangles show values in individual mice.

Fig 8 shows the total time and the number and duration of the episodes of wakefulness, NREMS and REMS during home-cage recordings in the light and dark periods.

During the dark period, the time spent in each wake-sleep state, the number of episodes of each wake-sleep state, and the duration of wakefulness episodes differed significantly among groups (P < 0.0426, Kruskal-Wallis tests). The number of wakefulness episodes also differed significantly among groups during the light period (P = 0.0456, Kruskal-Wallis test). However, post-hoc comparisons did not reveal any significant difference in these variables due to treatment (P > 0.2908, post-hoc corrected comparisons).

### Sleep deprivation and recovery

The hourly profiles of the time spent in each wake-sleep state during sleep deprivation and recovery are reported in S1 Fig. Sleep deprivation was effective as indicated by the almost complete absence of sleep during the 6 h of this protocol (S1 Fig, Panel A), and by the rapid and wide increase in SWA during sleep recovery after deprivation (S3 Fig, Panel E) compared with baseline recordings at the same ZT (S2 Fig, Panel E).

Female mice spent more time awake and less time in NREMS than male mice during the sleep recovery period (P = 0.0003 and P = 0.0047, respectively, ANOVA main effect, S1 Fig, Panel B) without significant treatment effect of sex x treatment interaction (P > 0.6925). No significant difference among groups was found in REMS (P > 0.0975, two-way ANOVA).

### EEG power spectra during home-cage recordings

The frequency of the EEG power spectral peak in NREMS and REMS during baseline home-cage recordings (S2 Fig) and during sleep recovery (S3 Fig) did not differ significantly among groups (P > 0.1283, Kruskal-Wallis tests). Similarly, no significant difference was found in SWA values during NREMS during baseline home-cage recordings (S2 Fig) and during sleep recovery (S3 Fig) (P > 0.1474, two-way ANOVAs).

### Plasma corticosterone levels

Plasma corticosterone levels did not differ significantly between CLF (15.20 (13.98) μg/dL) and TRF (14.69 (5.34) μg/dL) before the restraining stress test (time 0, T0) (P = 0.5457, Mann-Whitney test). No significant differences were found after the stress test (T1), either. The plasma corticosterone increase at T1 with respect to T0 was 6.71 (4.83) μg/dL in CLF and 6.18 (29.2) μg/dL in TRF (P = 0.8633, Mann-Whitney test). The measured inter-assay coefficient of variation was 4.7%, whereas the measured intra-assay coefficient of variation was 4.1% for the first plate and 4.9% for the second plate.

### Gene expression analysis

**Fig 9:**
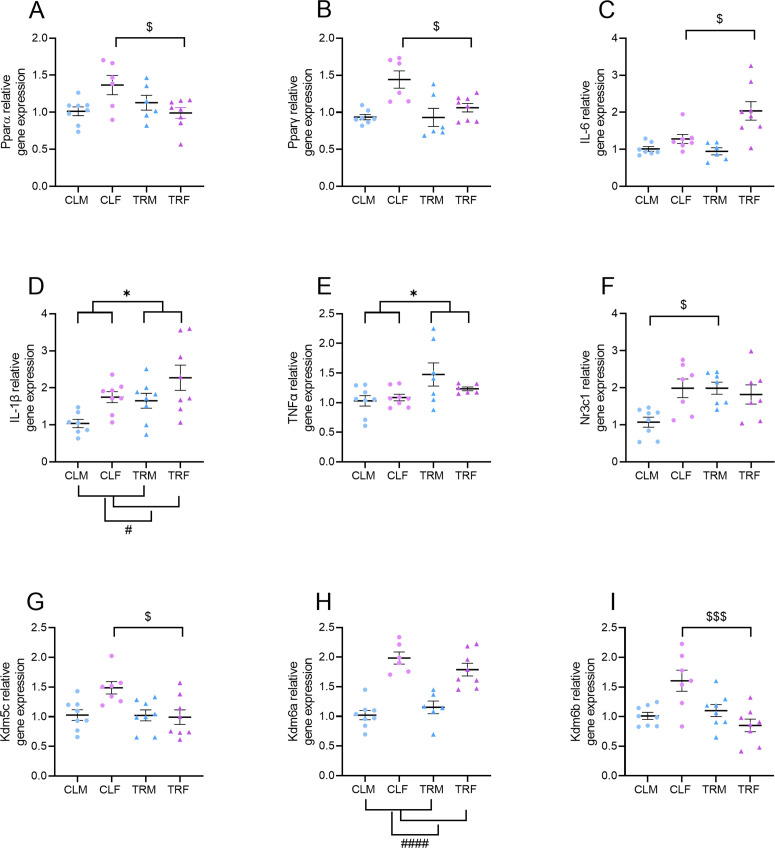
Gene expression analysis. Panels A and B show the **Ppar*α* and **Ppar*γ* relative gene expression in male and female mice born to vehicle-treated dams (CLM and CLF) or to Chlorpyrifos-treated dams (TRM and TRF). Panel C shows the *IL-6* relative gene expression. Panels D-I respectively show the relative gene expression of *IL-1β*, **Tnf*α*, *Nr3c1*, *Kdm5c*, *Kdm6a*, and *Kdm6b*. * indicates P < 0.05 for the main effect of treatment of two-way ANOVA. # and #### indicate P < 0.05 and P < 0.0001 for the main effect of sex of two-way ANOVA. $ and $$$ indicate P < 0.05 and P < 0.001, respectively, for Tukey’s multiple comparisons test after two-way ANOVA. Bars show mean ± SEM. Dots and triangles show values in individual mice.

Fig 9 shows the results of relative gene expression in the hippocampus of male and female adult mice perinatally exposed to CPF or vehicle. Two-way ANOVA revealed a significant sex x treatment interaction on the relative mRNA expression of Pparα, Pparγ, IL-6, and Nr3c1 (P < 0.0361). The expression of *Pparα* and *Pparγ* was significantly lower in TRF than in CLF (P = 0.029, P = 0.0181, respectively) while *IL-6* mRNA levels were significantly higher (P = 0.0127) in TRF than in CLF. In male mice the expression of *Nr3c1* was significantly higher in TRM than in CLM (P = 0.0175) (Tukey’s multiple comparisons tests). Moreover, the expression of *IL-1β* and *Tnfα* was higher in mice born to CPF-treated dams than in mice born to vehicle-treated dams (P = 0.0175 and P = 0.0142, respectively, main effects of treatment, two-way ANOVAs) without significant sex x treatment interactions (P > 0.1983).

The expression of the histone demethylase gene *Kdm6a* was higher in female mice than in male mice (P < 0.0001, ANOVA main effect of sex) without significant effects of treatment or sex x treatment interaction (P > 0.1150). On the other hand, the expression of the *Kdm5c* and *Kdm6b* histone demethylase genes displayed significant sex x treatment interaction at ANOVA (P < 0.0254). Post-hoc analyses indicated that the expression of both *Kdm5c* and *Kdm6b* was significantly lower in TRF than in CLF (P = 0.0126 and P = 0.0005, respectively, Tukey’s multiple comparisons tests).

## Discussion

The present study indicated that perinatal CPF exposure produced the following effects in adult mice: (a) a long-lasting reprogramming of breathing during sleep, with a higher occurrence rate of sighs and apnoeas particularly in female mice; (b) an upregulation of pro-inflammatory cytokines mRNA levels and a decrease in the gene expression of nuclear anti-inflammatory PPARs in the hippocampus of female mice.

Several molecular pathways may explain the links between perinatal exposure to OPs, such as CPF, and offspring health. CPF is metabolized to its oxygen analog (CPF-oxon) which inhibits AChE [62]. Our CPF treatment dosage was sufficient to cause a significant reduction of AChE activity in the CPF-treated dams compared to vehicle-treated dams (Fig 2).

The finding that perinatal exposure of mice to CPF was associated with a higher occurrence rate of apnoeas and sighs during sleep (Fig 7) was in line with the results of a previous study on male rats [22] and indicates that the adult respiratory phenotype during sleep may be modulated by early-life exposure to CPF during pregnancy and breastfeeding. In the work presented by Darwiche and colleagues [22], pregnant rats were exposed to oral CPF (1 or 5 mg/kg/day) or to vehicle from gestation onset up to weaning of the pups and differently to our study, measurements were made at weaning (day 21) and adulthood (day 60) over a 60-minute period, during which the rats were continuously scored for wakefulness or sleep with an observational evaluation [22].

OPs exert both acute and chronic effects on breathing. The well-characterized episodes of central apnoea observed during acute exposure to OPs are caused by AChE inhibition in the respiratory centres [63–65] with overstimulation of the muscarinic receptors in the central nervous system [65,66]. Moreover, prenatal exposure to CPF alters the development of brain cholinergic areas involved in the regulation of breathing [67–69]. CPF exposure has been associated with a higher apnoea index in adult rats than in juvenile rats, whereas in untreated control rats, maturation from the juvenile age to adulthood tended to decrease the apnoea index [22]. CPF exposure might thus inhibit the processes through which sleep apnoea decreases with age in rodents [22]. CPF-induced respiratory disturbances may result from neurotoxic effects on central respiratory networks. Specifically, the pre-Bötzinger complex and the parabrachial/Kölliker-Fuse nuclei—key regions for respiratory rhythm and phase transitions—receive both muscarinic and nicotinic central input and may be particularly susceptible to CPF exposure [70,71]. Thus, CPF may impair autonomic regulation by interfering with vagal afferents or central autonomic nuclei, potentially disrupting the control of sighs and apnoeas [72]. While our study did not directly assess these mechanisms, they represent testable hypotheses for future investigation.

These results may help clarify the mechanism underlying relationship between perinatal CPF exposure and later respiratory disturbances. Accordingly, a longitudinal human study of a birth cohort of Mexican-American mother-child pairs showed that exposure to OPs including CPF, during the third trimester of pregnancy was associated with an increased likelihood of respiratory disturbances during childhood, including sleep-disordered breathing [73]. The CPF dose used in this study (5 mg/kg/day) exceeds typical environmental exposure levels in humans. However, similar doses have been used in developmental neurotoxicity models aiming to investigate subtle, long-term effects without inducing acute toxicity [74]. Although our study did not directly assess the actual concentrations of CPF or its metabolites within the organism, such as in blood or tissues, previous biomonitoring studies have reported detectable levels of 3,5,6-trichloro-2-pyridinol (TCPy), a metabolite of CPF, in the urine of pregnant women living in agricultural areas [75]. These findings highlight the potential for CPF-related exposure during gestation and support the relevance of using rodent models to investigate CPF-induced neurodevelopmental effects. Therefore, although the administered dose is higher than environmental exposure estimates, the outcomes observed in our study may still inform risks associated with early-life CPF exposure in humans.

Furthermore, recent epidemiological evidence suggests an association between prenatal CPF exposure and increased risk for neurodevelopmental disorders such as autism spectrum disorder (ASD) and attention-deficit/hyperactivity disorder (ADHD) [76]. The mechanistic underpinnings of these associations may involve persistent neuroinflammation, oxidative stress, and dysregulation of neurotransmitter pathways.

Preclinical studies suggest that neuroprotective strategies targeting oxidative stress and inflammation — such as antioxidants (e.g., N-acetylcysteine), anti-inflammatory agents, and PPARγ agonists (e.g., rosiglitazone) — may attenuate CPF-induced neurotoxicity. For instance, Abd-Elhamid et al. (2024) demonstrated that diosmin, a bioactive flavonoid compound known for its antioxidant and anti-inflammatory effects, protected against CPF-induced brain injury in rats via PPARγ activation and suppression of NF-κB/AP-1 signalling [77]. These findings highlight the translational potential of such interventions to mitigate CPF-related effects during critical developmental windows.

Molecular results showed a significant decrease in both PPARα and PPARγ mRNA levels in the hippocampus of adult female rats perinatally exposed to CPF, thus suggesting that this pesticide might promote an inflammatory state predominantly in females. Indeed, in CPF-exposed females subjects, the reduction in PPARs was accompanied by a significant increase in the pro-inflammatory cytokines IL-1β and IL-6. In contrast, only a slight increase in IL-1β and TNFα levels in the hippocampus of male counterpart was detected (Fig 9). Consistent with previous studies, these results confirm that CPF induces neuroinflammation in this specific brain region by increasing pro-inflammatory mediators in a rat model as well [78] and, in addition, point out its ability to alter the gene expression of nuclear receptors PPARs. Differently to our study, Gómez-Giménez and colleagues evaluated the effects of peri-gestational exposure to CPF (different doses lower than 1 mg/kg/day) in 2–3 months old rats on pro- and anti-inflammatory cytokines and on neurotransmitter receptors in the hippocampus by Western blot [78]. PPARs have been identified as key regulators of immune signalling pathways including NF-κB, MAPK, JAK/STAT, and AMPK, likely involved in neuroinflammation associated with neurodegenerative diseases [39].

A negative relationship between hippocampal ILs levels and spatial learning and memory has been reported in adult male rats perinatally exposed to various pesticides [78]. In our study, however, perinatal CPF exposure resulted in a significant increase in spontaneous alternations in the Y-maze test (Fig 3), contrary to what was expected with reduced short-term memory. The discrepancy may be due to differences in the species, dose of CPF, type of treatment and/or to the differential effects of OPs depending on the cognitive domain evaluated [30,79].

We also found that perinatal exposure to CPF significantly reduced the expression of histone demethylases *Kdm5c* and *Kdm6b* in female mice only (Fig 9), further indicating a higher susceptibility of females to the long-lasting effects of CPF. Although other studies suggested a role of KDMs in increasing pro-inflammatory mediators as well as in a negative modulation of the anti-inflammatory nuclear PPARs [40,41,80], our gene expression data do not consistently support this relationship. In this regard, additional studies assessing KDMs protein expression levels and their binding to gene regulatory regions are warranted to further elucidate the specific contribution of KDM-mediated histone demethylation to the observed molecular and behavioural alterations. Noteworthy, other epigenetic mechanisms different from histone methylation, such as DNA methylation, could play a role in CPF-induced gene expression alterations of both PPARs and ILs here observed. Indeed, recent evidence has shown that prenatal CPF exposure is associated with increased DNA methylation in *Pparγ* gene promoter region in humans [28]. In addition, DNA methylation analysis in blood and saliva from subjects with long-term exposure to environmental OPs revealed an enrichment for genes involved in neurotransmission and inflammation [81].

To the best of our knowledge, the long-term effects of perinatal exposure to CPF on the wake-sleep phenotype have never been previously assessed in mice (Fig 8). Our study did not detect any such effect. The negative impact of OPs exposure on sleep among farmworkers has been documented [82,83]. In these studies, the direct exposure pattern was evaluated, and symptoms were collected with self-reported and pre-established questionnaires to diagnose insomnia and sleep problems [82,83].

As previously mentioned, our findings showed an increase in the percentage of alternations in the Y-maze in female mice subjected to perinatal CPF exposure and this result might suggest residual cholinergic hyperstimulation. Anyway, this effect could also be interpreted in the context of serotonergic dysfunction. Previous studies have shown that prenatal CPF exposure may reduce serotonergic tone [84]. Acetylcholine and serotonin are known to exert opposing influences on attention and memory tasks [85], suggesting that the altered balance between these systems may contribute to enhanced Y-maze performance without necessarily indicating improved cognition [86]. Nevertheless, this unexpected result could also be a type I statistical error and should be considered exploratory. On the other hand, we assessed anxiety-like behaviour with the OF and EPM (Figs 4 and 5), which are particularly sensitive to anxiety trait (i.e., stable anxiety over time) and state (i.e., anxiety at a given moment when facing a threat) [87]. In the OF test, the time spent close to the open-field border was lower in TRM than in CLM, suggesting that perinatal CPF treatment does not increase the anxiety trait selectively in male mice. In the EPM test, male and female mice born to CPF-treated dams spent more time in open arms than control mice, suggesting a lower anxiety state. These observations, in line with the effect induced by an acute CPF administration (250 mg/kg s.c.) in rats [88], might reflect neurochemical imbalances induced by CPF exposure during embryonic development instead of a true anxiolytic action. Interestingly, other studies have reported increased anxiety-like behaviour following repeated CPF or dichlorvos exposure, likely due to oxidative and neurogenic damage [30], suggesting that behavioural outcomes may vary depending on dose, timing, and duration of exposure. To summarize, prenatal CPF exposure might lead to long-lasting remodelling of the serotonergic system, resulting in reduced serotonin function. Such a reduction might increase exploratory behaviour, including more time spent in the open arms of the EPM [89]. Perinatal CPF exposure did not alter mechanical sensitivity in adulthood, as showed by von Frey test (S3 Table).

We found that perinatal CPF exposure was associated with upregulation of the expression of the glucocorticoid receptor (*Nr3c1*) in the hippocampus of male mice only (Fig 9). This finding would suggest a reduced activity of the HPA axis and a consequent reduction of circulating corticosterone levels in male mice with perinatal CPF exposure. Unfortunately, we were able to measure plasma corticosterone only in a subset of female mice, finding no significant effect of perinatal CPF exposure. These results suggest that perinatal CPF exposure does not lead to long-lasting changes of the HPA axis in female mice but leave open the possibility that they do so in male mice.

It is notable that most of the studies recently performed have been conducted using exclusively male animals’ models. These male-biased research results may hinder the characterization of sexually dimorphic effects [90], and it remains unclear whether the findings are specific to males or valid for both sexes. Sex is increasingly recognized as one of the major influencing factors in disorders across all medical specialties and, despite its 20 years of history, gender medicine is still little known [91]. This research was mostly realized using both male and female mice revealing that some differences were specific to one sex or the other.

Some limitations of the present study must be acknowledged. We studied adult animals (17 weeks old), but it could be possible that phenotypic alterations, such as those of the wake-sleep profile, emerged earlier in life after perinatal CPF treatment and underwent compensation at the time of study. We also have no data to support the persistence of phenotypic alterations in old age. An important limitation of our study is that we did not quantify plasma corticosterone levels in all the experimental animals but only on a subset of female mice, as previously mentioned. This limits our ability to draw firm conclusions regarding potential HPA axis dysfunction, particularly in males, where transcriptional changes in Nr3c1 were observed but not corroborated by hormonal data. Future studies should include comprehensive plasma corticosterone measurements across both sexes and time points to better assess potential long-term alterations in stress responsiveness. Another limitation is that we analysed the hippocampus as a whole, without evaluating the presence of regional differences in the gene expression analysis.

Another limitation of the present study lies in its cross-sectional design and reliance on a single time point for molecular analysis. Although we detected gene expression changes in adulthood, we cannot determine when these alterations emerged, how long they persisted, or whether compensatory mechanisms occurred at earlier or later stages of development. Longitudinal studies assessing CPF-induced phenotypic and molecular effects at multiple life stages—from early postnatal periods to aging—are essential to clarify the trajectory of CPF neurotoxicity. Such studies would be particularly valuable to determine whether observed behavioural or respiratory changes are transient, cumulative, or potentially reversible. Additionally, integrating protein-level assessments and epigenetic profiling would provide a more complete understanding of the persistence and functional relevance of CPF-induced transcriptional changes. Finally, an important limitation of our study is that we did not explore dose-response relationships for CPF, which does not allow inference on whether such effects are linear or threshold-based.

## Conclusions

In conclusion, we found evidence that perinatal CPF exposure produced long-lasting alterations in adult mice on breathing patterns during sleep and cytokine expression in the hippocampus, potentially increasing susceptibility to sleep-disordered breathing and neuroinflammation in adulthood. CPF perinatal exposure induced alterations particularly in female mice, indicating a higher susceptibility of this sex to this pesticide. An important novelty of this study was related to the use of male and female mice underlining the importance of sex-related differences also in basic research. These findings add to the literature on pregnancy and breastfeeding as a potentially vulnerable period to pesticide exposure for the offspring and support the view that our adult sleep phenotype, and its potential alterations, can be associated with early-life events during pregnancy and lactation.

## Supporting information

S1 Fig Sleep deprivation and recovery after sleep deprivation. Panel A shows the 24-h hourly profile of the time spent in wakefulness during 6 h of sleep deprivation and the following 18 h of recovery in adult male and female mice born to vehicle-treated dams (CLM and CLF, respectively) or to Chlorpyrifos-treated dams (TRM and TRF, respectively). Sleep deprivation was performed for 6 h by gentle handling from lights on (Zeitgeber Time 0, ZT0) to ZT6. Panel B shows the percentage of time spent in wakefulness (W), non-rapid-eye-movement sleep (NREMS), and rapid-eye-movement sleep (REMS) in the recovery period. Data are reported as mean ± SEM. Dots and triangles show values in individual mice. Panel B: ## and ### indicate P < 0.005 and P < 0.001, respectively, for the main effect of sex of two-way ANOVA.
(TIF)

S2 Fig Electroencephalogram analysis during sleep in baseline conditions. Electroencephalographic (EEG) power spectral density (PSD) during non-rapid-eye-movement sleep (NREMS) and rapid-eye-movement sleep (REMS) (Panels A and C, respectively), expressed as a percentage of total EEG spectral power, exhibited during baseline conditions by male and female mice born to vehicle-treated dams (CLM and CLF, respectively) or to Chlorpyrifos-treated dams (TRM and TRF, respectively). Panels B and D respectively show the individual and median (range) of the EEG peak frequency during NREMS and REMS. Panel E shows power in the delta frequency range (1–4 Hz, EEG slow-wave activity, SWA) during NREMS in baseline recordings.
(TIF)

S3 Fig Electroencephalogram analysis after 6 h of sleep deprivation. Electroencephalographic (EEG) power spectral density (PSD) during non-rapid-eye-movement sleep (NREMS) and rapid-eye-movement sleep (REMS) (Panels A and C, respectively), expressed as a percentage of total EEG spectral power, exhibited after 6 h of sleep deprivation by male and female mice born to vehicle-treated dams (CLM and CLF, respectively) or to Chlorpyrifos-treated dams (TRM and TRF, respectively). Panels B and D respectively show the individual and median (range) of the EEG peak frequency during NREMS and REMS after 6 h of sleep deprivation. Panel E shows power in the delta frequency range (1–4 Hz, EEG slow-wave activity, SWA) during NREMS after 6 h of sleep deprivation.
(TIF)

S1 Table Oestrous cycle stage evaluation. The table reports the oestrous phase (pro-oestrus, oestrus, metoestrus, dioestrus) after behavioural tests, before whole-body plethysmograph recordings, after baseline sleep recordings, and at sacrifice in female mice born to vehicle-treated dams (CLF) or to Chlorpyrifos-treated dams (TRF). The oestrous cycle phase evaluation failed in 2 CLF mice before behaviour and at sacrifice, in 4 TRF mice before behaviour, and in 1 TRF mouse at sacrifice.
(PDF)

S2 Table Primer sequences used for real-time qPCR. (PDF)

S3 Table Mechanical allodynia (von Frey test). The table reports the paw withdrawal latency (PWL, s) and the paw withdrawal threshold (PWT, g) in the von Frey test in male and female mice born to vehicle-treated dams (CLM and CLF) or to Chlorpyrifos-treated dams (TRM and TRF). Data are reported as median (range).
(PDF)

S4 Table Novel object recognition test. The novel object recognition index (discrimination index) was calculated by dividing the amount of exploration of the novel object by the total amount of object exploration during the test session by male and female mice born to vehicle-treated dams (CLM and CLF) or to Chlorpyrifos-treated dams (TRM and TRF). Data are reported as median (range).
(PDF)

S1 File Tables of manuscript dataset providing raw data. (PDF)

S2 File Supplementary methods. (PDF)
