# Supplementary figures and images for: Long lasting effects of perinatal exposure to the Chlorpyrifos pesticide on sleep, breathing, and neuroinflammation in adult mice
Source: PLoS One. 2025 Aug 1;20(8):e0328581. doi: 10.1371/journal.pone.0328581 (PMC12316233; doi:10.1371/journal.pone.0328581)

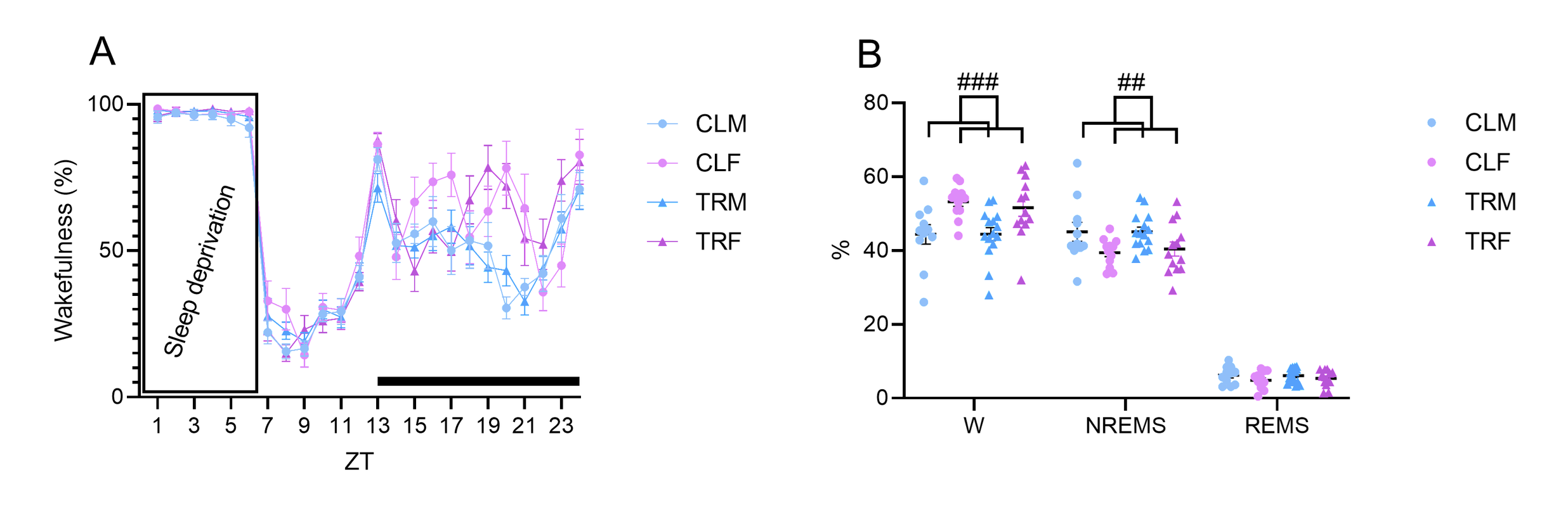

Supplement: S1 Fig — Panel A shows the 24-h hourly profile of the time spent in wakefulness during 6 h of sleep deprivation and the following 18 h of recovery in adult male and female mice born to vehicle-treated dams (CLM and CLF, respectively) or to Chlorpyrifos-treated dams (TRM and TRF, respectively). Sleep deprivation was performed for 6 h by gentle handling from lights on (Zeitgeber Time 0, ZT0) to ZT6. Panel B shows the percentage of time spent in wakefulness (W), non-rapid-eye-movement sleep (NREMS), and rapid-eye-movement sleep (REMS) in the recovery period. Data are reported as mean ± SEM. Dots and triangles show values in individual mice. Panel B: ## and ### indicate P < 0.005 and P < 0.001, respectively, for the main effect of sex of two-way ANOVA. (TIF) [file pone.0328581.s001.tif]

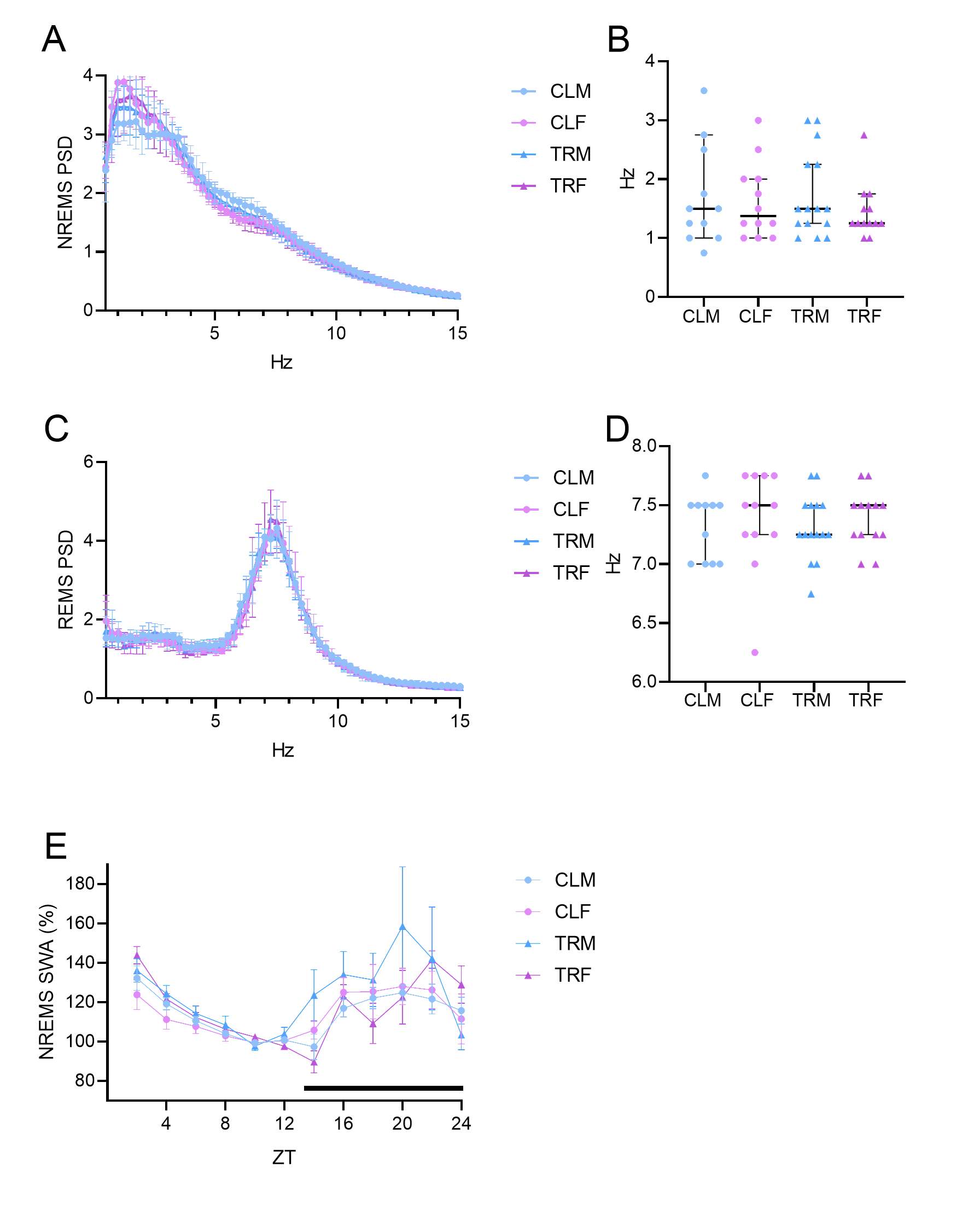

Supplement: S2 Fig — Electroencephalographic (EEG) power spectral density (PSD) during non-rapid-eye-movement sleep (NREMS) and rapid-eye-movement sleep (REMS) (Panels A and C, respectively), expressed as a percentage of total EEG spectral power, exhibited during baseline conditions by male and female mice born to vehicle-treated dams (CLM and CLF, respectively) or to Chlorpyrifos-treated dams (TRM and TRF, respectively). Panels B and D respectively show the individual and median (range) of the EEG peak frequency during NREMS and REMS. Panel E shows power in the delta frequency range (1–4 Hz, EEG slow-wave activity, SWA) during NREMS in baseline recordings. (TIF) [file pone.0328581.s002.tif]

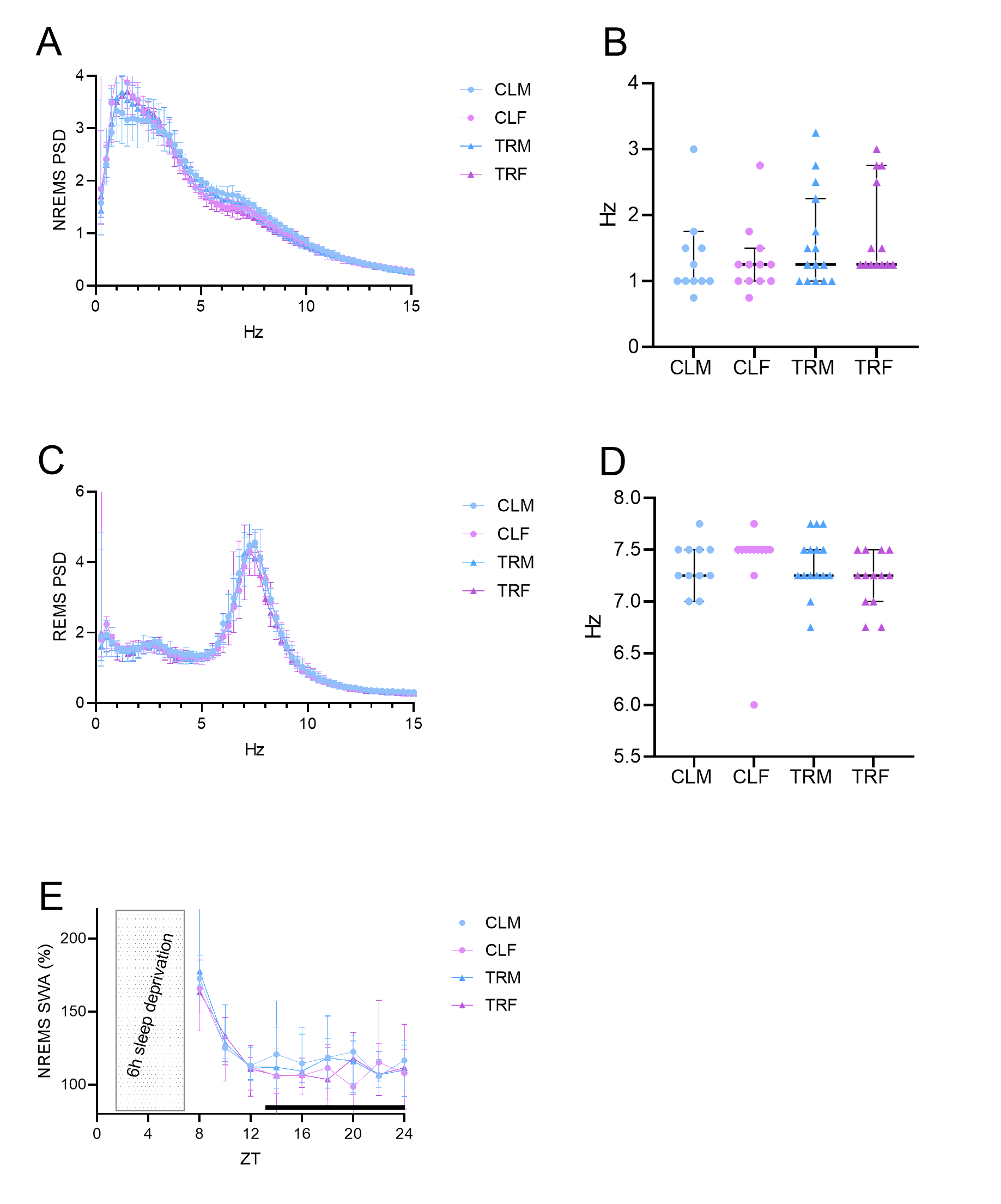

Supplement: S3 Fig — Electroencephalographic (EEG) power spectral density (PSD) during non-rapid-eye-movement sleep (NREMS) and rapid-eye-movement sleep (REMS) (Panels A and C, respectively), expressed as a percentage of total EEG spectral power, exhibited after 6 h of sleep deprivation by male and female mice born to vehicle-treated dams (CLM and CLF, respectively) or to Chlorpyrifos-treated dams (TRM and TRF, respectively). Panels B and D respectively show the individual and median (range) of the EEG peak frequency during NREMS and REMS after 6 h of sleep deprivation. Panel E shows power in the delta frequency range (1–4 Hz, EEG slow-wave activity, SWA) during NREMS after 6 h of sleep deprivation. (TIF) [file pone.0328581.s003.tif]
