# Supplementary material for: Long lasting effects of perinatal exposure to the Chlorpyrifos pesticide on sleep, breathing, and neuroinflammation in adult mice
Source: PLoS One. 2025 Aug 1;20(8):e0328581. doi: 10.1371/journal.pone.0328581 (PMC12316233; doi:10.1371/journal.pone.0328581)
Supplement: S1 Table — The table reports the oestrous phase (pro-oestrus, oestrus, metoestrus, dioestrus) after behavioural tests, before whole-body plethysmograph recordings, after baseline sleep recordings, and at sacrifice in female mice born to vehicle-treated dams (CLF) or to Chlorpyrifos-treated dams (TRF). The oestrous cycle phase evaluation failed in 2 CLF mice before behaviour and at sacrifice, in 4 TRF mice before behaviour, and in 1 TRF mouse at sacrifice. (PDF) [file pone.0328581.s004.pdf]

S1 Table. Oestrous cycle stage evaluation.

	Pro-oestrus	Oestrus	Metoestrus	Dioestrus
**Behavioural tests**				
CLF	2	7	6	4
TRF	2	3	4	1
**Plethysmography**				
CLF	3	4	2	3
TRF	3	2	3	5
**Baseline recordings**				
CLF	2	4	4	2
TRF	5	5	2	1
**Sacrifice**				
CLF	3	8	6	3
TRF	4	6	2	2

The table reports the oestrous phase (pro-oestrus, oestrus, metoestrus, dioestrus) after behavioural tests, before whole-body plethysmograph recordings, after baseline sleep recordings, and at sacrifice in female mice born to vehicle-treated dams (CLF) or to Chlorpyrifos-treated dams (TRF). The oestrous cycle phase evaluation failed in 2 CLF mice before behaviour and at sacrifice, in 4 TRF mice before behaviour, and in 1 TRF mouse at sacrifice.
