# Supplementary material for: Long lasting effects of perinatal exposure to the Chlorpyrifos pesticide on sleep, breathing, and neuroinflammation in adult mice
Source: PLoS One. 2025 Aug 1;20(8):e0328581. doi: 10.1371/journal.pone.0328581 (PMC12316233; doi:10.1371/journal.pone.0328581)
Supplement: S2 Table — (PDF) [file pone.0328581.s005.pdf]

S2 Table. Primer sequences used for real-time qPCR.

Gene	Sense Primer (5' – 3')	Antisense Primer (5' – 3')
**Glyceraldehyde-3-phosphate dehydrogenase (Gapdh)**	AACTTTGGCATTGTGGAAGG	ACACATTGGGGGTAGGAACA
**Peroxisome proliferator-activated receptor α (Pparα)**	AGGGTTGAGCTCAGTCAGGA	GGTCACCTACGAGTGGCATT
**Peroxisome proliferator-activated receptor γ (Pparγ)**	GGAAGACCACTCGCATTCCTT	GTAATCAGCAACCATTGGGTCA
**Interleukin-6 (IL-6)**	GACAGGTCTGTTGGGAGTGGTATCCT	TGCCTTCTTGGGACTGATGCTGGT
**Interleukin-1beta (IL-1β)**	ACCTGCTGGTGTGTGACGTTCCCA	TGCAGGGTGGGTGTGCCGTCTTTCA
**Tumor necrosis factor-alpha (Tnf-α)**	AAGCAGAGGAGCAGCTGGAGTGGCT	AATCGGCTGACGGTGTGGGTGAGGA
**Glucocorticoid receptor (Nr3c1)**	AATGAGACCAGATGTGAGTTC	GGTAATTGTGCTGTCCTTCC
**Histone lysine demethylase 5C enzyme (Kdm5c)**	AAGATAAGACTCTGCGGAAAAAAGAT	TTGACATCCCCACCTAATTCCT
**Histone lysine demethylase 6A enzyme (Kdm6a)**	TTTGGTCTACTTCCATTACAATGCA	AAGCCCAAGTCGTAAATGAATTTC
**Histone lysine demethylase 6B enzyme (Kdm6b)**	ACCGCCTGCGTGCCTTAC	GTGTTGCTGCTGCTGCTACTG
