# Supplementary material for: Long lasting effects of perinatal exposure to the Chlorpyrifos pesticide on sleep, breathing, and neuroinflammation in adult mice
Source: PLoS One. 2025 Aug 1;20(8):e0328581. doi: 10.1371/journal.pone.0328581 (PMC12316233; doi:10.1371/journal.pone.0328581)
Supplement: S3 Table — The table reports the paw withdrawal latency (PWL, s) and the paw withdrawal threshold (PWT, g) in the von Frey test in male and female mice born to vehicle-treated dams (CLM and CLF) or to Chlorpyrifos-treated dams (TRM and TRF). Data are reported as median (range). (PDF) [file pone.0328581.s006.pdf]

S3 Table. Mechanical allodynia (von Frey test).

	CLM (n = 9)	TRM (n = 18)	CLF (n = 15)	TRF (n = 15)
PWL (s)	7.08 (14.93)	4.19 (9.58)	4.10 (18.68)	4.33 (5.70)
PWT (g)	1.78 (2.63)	1.43 (2.97)	1.37 (3.25)	1.42 (1.28)

The table reports the paw withdrawal latency (PWL, s) and the paw withdrawal threshold (PWT, g) in the von Frey test in male and female mice born to vehicle-treated dams (CLM and CLF) or to Chlorpyrifos-treated dams (TRM and TRF). Data are reported as median (range).
