# Supplementary material for: Long lasting effects of perinatal exposure to the Chlorpyrifos pesticide on sleep, breathing, and neuroinflammation in adult mice
Source: PLoS One. 2025 Aug 1;20(8):e0328581. doi: 10.1371/journal.pone.0328581 (PMC12316233; doi:10.1371/journal.pone.0328581)
Supplement: S4 Table — The novel object recognition index (discrimination index) was calculated by dividing the amount of exploration of the novel object by the total amount of object exploration during the test session by male and female mice born to vehicle-treated dams (CLM and CLF) or to Chlorpyrifos-treated dams (TRM and TRF). Data are reported as median (range). (PDF) [file pone.0328581.s007.pdf]

S4 Table. Novel object recognition test.

	CLF (n = 17)	TRF (n = 12)	CLM (n = 7)	TRM (n = 16)
Discrimination index (%)	78.5 (95.9)	76.8 (100)	79.8 (100)	77.5 (100)

The novel object recognition index (discrimination index) was calculated by dividing the amount of exploration of the novel object by the total amount of object exploration during the test session by male and female mice born to vehicle-treated dams (CLM and CLF) or to Chlorpyrifos-treated dams (TRM and TRF). Data are reported as median (range).
