# Supplementary material for: Long lasting effects of perinatal exposure to the Chlorpyrifos pesticide on sleep, breathing, and neuroinflammation in adult mice
Source: PLoS One. 2025 Aug 1;20(8):e0328581. doi: 10.1371/journal.pone.0328581 (PMC12316233; doi:10.1371/journal.pone.0328581)
Supplement: S1 File — (PDF) [file pone.0328581.s008.pdf]

# Supporting information S1 (raw data)

## AchE Activity (mU/ml)

| CPF-treated | CL-treated |
|---|---|
| 18617.98795 | 30451.01851 |
| 18279.69057 | 28559.89387 |
| 9408.928462 | 45856.08063 |
| 15705.28649 | 30377.58145 |
| 22120.97338 | 22302.86972 |
| 10083.64152 | |

## Mechanical allodynia

Paw withdrawal latency (s)

| CLF | CLM | TRF | TRM |
|---|---|---|---|
| 1.72 | 5.90 | 6.53 | 2.18 |
| 19.13 | 19.18 | 8.30 | 10.72 |
| 17.05 | 5.75 | 3.57 | 9.40 |
| 20.40 | 7.08 | 4.13 | 10.08 |
| 4.03 | 4.25 | 4.33 | 11.77 |
| 2.97 | 4.50 | 4.95 | 3.68 |
| 4.23 | 7.65 | 7.90 | 8.17 |
| 5.53 | 7.40 | 4.93 | 6.50 |
| 2.08 | 7.47 | 4.02 | 11.25 |
| 2.78 | | 2.68 | 4.42 |
| 4.78 | | 2.6 | 3.55 |
| 3.25 | | 7.5 | 3.30 |
| 4.10 | | 2.6 | 3.97 |
| 2.87 | | 5.0 | 2.92 |
| 6.43 | | 3.95 | 5.33 |
| | | | 2.83 |
| | | | 3.03 |

| | | | |
|---|---|---|---|
| | | | 2.30 |

Paw withdrawal threshold (g)

| CLF | CLM | TRF | TRM |
|---|---|---|---|
| 0.83 | 1.78 | 1.93 | 0.95 |
| 3.25 | 4.03 | 2.32 | 3.38 |
| 4.08 | 1.73 | 1.25 | 2.57 |
| 3.55 | 1.62 | 1.45 | 2.72 |
| 1.35 | 1.40 | 1.42 | 3.92 |
| 1.12 | 1.47 | 1.57 | 1.27 |
| 1.40 | 2.18 | 2.22 | 2.28 |
| 1.68 | 2.12 | 1.40 | 1.92 |
| 0.92 | 2.13 | 1.37 | 3.00 |
| 1.23 | | 1.07 | 1.52 |
| 1.52 | | 1.03 | 1.25 |
| 1.17 | | 2.08 | 1.18 |
| 1.37 | | 1.03 | 1.35 |
| 1.10 | | 1.52 | 1.08 |
| 1.88 | | 1.33 | 1.67 |
| | | | 1.08 |
| | | | 1.13 |
| | | | 0.98 |

# Y-maze

% alternations

| CLF | CLM | TRF | TRM |
|---|---|---|---|
| 45.00 | 100.00 | 48.39 | 76.92 |
| 41.94 | 57.89 | 30.30 | 64.71 |
| 42.86 | 44.44 | 46.15 | 56.00 |
| 45.16 | 27.50 | 88.24 | 26.09 |
| 31.03 | 73.68 | 55.56 | 17.65 |
| 59.26 | 63.64 | 75.00 | 80.95 |

| | | | |
|---|---|---|---|
| 29.17 | 84.62 | 45.00 | 65.38 |
| 41.38 | | 62.50 | 36.36 |
| 25.71 | | 46.15 | 64.71 |
| 30.00 | | 58.82 | 76.92 |
| 53.85 | | 69.70 | 50.00 |
| 46.15 | | 57.14 | 72.22 |
| 42.86 | | 64.00 | 85.00 |
| 28.57 | | | 68.97 |
| 81.82 | | | 57.14 |
| | | | 71.88 |

Total alternations

| **CLF** | **CLM** | **TRF** | **TRM** |
|---|---|---|---|
| 22 | 11 | 33 | 15 |
| 33 | 21 | 35 | 19 |
| 30 | 20 | 15 | 27 |
| 33 | 42 | 19 | 48 |
| 31 | 21 | 11 | 36 |
| 29 | 24 | 26 | 23 |
| 26 | 15 | 22 | 28 |
| 31 | | 18 | 24 |
| 37 | | 28 | 36 |
| 42 | | 19 | 28 |
| 15 | | 35 | 32 |
| 28 | | 23 | 20 |
| 16 | | 27 | 22 |
| 16 | | | 31 |
| 24 | | | 23 |
| | | | 34 |

# EPM

Total number of entries

| CLF | CLM | TRF | TRM |
|---|---|---|---|
| 26 | 31 | 34 | 35 |
| 25 | 32 | 14 | 16 |
| 24 | 13 | 16 | 23 |
| 27 | 31 | 27 | 25 |
| 29 | 23 | 29 | 42 |
| 27 | 37 | 36 | 36 |
| 25 | 20 | 19 | 31 |
| 31 | | 22 | 21 |
| 31 | | 30 | 35 |
| 40 | | 21 | 21 |
| 27 | | 24 | 29 |
| 27 | | 28 | 13 |
| 18 | | | 33 |
| 22 | | | 22 |
| 23 | | | 24 |
| | | | 23 |
| | | | 22 |
| | | | 27 |

% time in open-arm

| CLF | CLM | TRF | TRM |
|---|---|---|---|
| 34.62 | 41.94 | 41.17 | 40.00 |
| 44.00 | 46.88 | 42.86 | 37.50 |
| 33.33 | 30.77 | 50.00 | 52.17 |
| 55.56 | 61.29 | 48.15 | 40.00 |
| 48.28 | 34.78 | 55.17 | 50.00 |
| 40.74 | 37.84 | 38.89 | 47.22 |
| 36.00 | 25.00 | 42.11 | 48.39 |
| 35.48 | | 45.45 | 52.38 |
| 41.94 | | 36.67 | 42.86 |

| | | | |
|---|---|---|---|
| 50.00 | | 38.10 | 42.86 |
| 37.04 | | 45.83 | 51.72 |
| 29.63 | | 42.86 | 53.85 |
| 27.78 | | | 48.48 |
| 31.82 | | | 50.00 |
| 43.48 | | | 58.33 |
| | | | 47.83 |
| | | | 50.00 |
| | | | 40.74 |

% of open-arm entries

| CLF | CLM | TRF | TRM |
|---|---|---|---|
| 28.58 | 52.44 | 37.38 | 37.94 |
| 30.21 | 24.84 | 43.43 | 10.30 |
| 14.44 | 2.84 | 39.87 | 24.82 |
| 30.70 | 51.74 | 23.27 | 17.82 |
| 22.40 | 9.49 | 31.05 | 44.07 |
| 35.50 | 37.12 | 24.81 | 29.91 |
| 20.46 | 2.77 | 18.26 | 39.00 |
| 20.21 | | 28.38 | 28.86 |
| 37.69 | | 27.38 | 41.46 |
| 47.60 | | 30.64 | 50.99 |
| 31.83 | | 22.60 | 44.08 |
| 23.73 | | 27.19 | 73.56 |
| 6.65 | | | 41.91 |
| 20.99 | | | 15.64 |
| 35.82 | | | 23.20 |
| | | | 38.37 |
| | | | 28.04 |
| | | | 48.07 |

# OF

Frequency centre (n)

| CLF | CLM | TRF | TRM |
|---|---|---|---|
| 47 | 35 | 33 | 38 |
| 39 | 53 | 51 | 39 |
| 32 | 23 | 43 | 36 |
| 65 | 38 | 39 | 30 |
| 44 | 66 | 27 | 38 |
| 53 | 67 | 43 | 55 |
| 42 | 27 | 22 | 33 |
| 59 | | 53 | 51 |
| 33 | | 17 | 59 |
| 40 | | 10 | 47 |
| 44 | | 40 | 51 |
| 44 | | 34 | 37 |
| 42 | | 68 | 34 |
| 40 | | 24 | 38 |
| 46 | | | 78 |
| 47 | | | 57 |
| 30 | | | 34 |
| | | | 43 |

Frequency border (n)

| CLF | CLM | TRF | TRM |
|---|---|---|---|
| 48 | 37 | 38 | 49 |
| 43 | 54 | 52 | 39 |
| 35 | 24 | 65 | 41 |
| 65 | 42 | 44 | 35 |
| 46 | 67 | 42 | 41 |
| 54 | 68 | 45 | 57 |
| 47 | 29 | 25 | 58 |
| 62 | | 54 | 52 |

| | | | |
|---|---|---|---|
| 39 | | 31 | 60 |
| 49 | | 16 | 57 |
| 59 | | 50 | 59 |
| 51 | | 36 | 40 |
| 54 | | 76 | 35 |
| 42 | | 34 | 41 |
| 46 | | | 78 |
| 52 | | | 58 |
| 54 | | | 39 |
| | | | 45 |

Time centre (s)

| CLF | CLM | TRF | TRM |
|---|---|---|---|
| 84.08 | 26.96 | 31.68 | 64.56 |
| 69.6 | 42.56 | 47.44 | 33.12 |
| 56.16 | 22.88 | 93.76 | 26.16 |
| 79.44 | 42.64 | 68.16 | 45.92 |
| 123.12 | 52.8 | 29.36 | 62.72 |
| 128.32 | 56.88 | 62.48 | 117.52 |
| 34.48 | 19.84 | 28.24 | 30.96 |
| 52.56 | | 67.68 | 69.44 |
| 36.16 | | 19.68 | 55.6 |
| 50.72 | | 12.96 | 53.44 |
| 63.44 | | 43.04 | 49.44 |
| 50.8 | | 28.64 | 54.32 |
| 32.16 | | 72.16 | 38.48 |
| 46.32 | | 28.64 | 76.48 |
| 46.24 | | | 59.12 |
| 60.96 | | | 98.72 |
| 27.04 | | | 52.56 |
| | | | 59.2 |

Time border (s)

| CLF | CLM | TRF | TRM |
|---|---|---|---|
| 516 | 571.04 | 554.16 | 504.56 |
| 529.6 | 557.52 | 552.64 | 542.64 |
| 542.72 | 577.2 | 498 | 572.64 |
| 520.64 | 557.12 | 530.48 | 549.6 |
| 476.48 | 547.04 | 543.04 | 537.12 |
| 471.76 | 537.44 | 519.2 | 482.08 |
| 564.24 | 579.84 | 571.68 | 523.28 |
| 547.12 | | 503.36 | 530.16 |
| 556 | | 533.28 | 544.48 |
| 546.72 | | 538.32 | 509.44 |
| 505.36 | | 551.12 | 511.52 |
| 544.56 | | 537.04 | 545.44 |
| 541.76 | | 506.24 | 561.6 |
| 553.6 | | 554.32 | 521.44 |
| 541.36 | | | 540.96 |
| 517.04 | | | 501.36 |
| 532.96 | | | 538.56 |
| | | | 539.28 |

# NOR

Discrimination index (%)

| CLF | CLM | TRF | TRM |
|---|---|---|---|
| 32.45033 | 0 | 79.79798 | 0 |
| 95.90164 | 79.82456 | 72.5 | 100 |
| 0 | 100 | 0 | 52.27273 |
| 86.87783 | 94.05941 | 100 | 73.07692 |
| 22.91667 | 56.6474 | 20.63492 | 96.22642 |
| 70.98765 | 96.88474 | 61.50235 | 0 |
| 42.29249 | 17.53372 | 95.91837 | 86.66667 |

| | | | |
|---|---|---|---|
| 71.13402 | | 100 | 45.09466 |
| 83.07692 | | 60.97561 | 92.76213 |
| 76.47059 | | 94.45644 | 41.81818 |
| 84.96583 | | 73.75622 | 82.93333 |
| 88.52459 | | 82.75194 | 12.5 |
| 80.4878 | | | 89.26174 |
| 71.79487 | | | 61.90476 |
| 81.13208 | | | 100 |
| 78.51852 | | | 81.81818 |
| 89.26799 | | | |

# Body weight at surgery (g)

| CLF | CLM | TRF | TRM |
|---|---|---|---|
| 22.4 | 25.5 | 21.6 | 24.9 |
| 19.2 | 29.2 | 19.9 | 27 |
| 22.9 | 24.4 | 21.9 | 27.4 |
| 19.8 | 26.3 | 22.3 | 26.4 |
| 22.9 | 29 | 20.8 | 24.7 |
| 22.3 | 29.3 | 21 | 26.7 |
| 16.3 | 29.9 | 22.6 | 29.7 |
| 22 | 26.1 | 21.2 | 26.3 |
| 23.1 | 27 | 23.9 | 26.1 |
| 20.4 | 24.4 | 24.2 | 30.4 |
| 21.8 | 26.9 | 23.9 | 25.4 |
| 22.1 | 26.2 | 22.4 | 24.5 |
| | 26.3 | 22.8 | 27.9 |
| | | | 21.3 |
| | | | 28.6 |

# Age at surgery (weeks)

| CLF | CLM | TRF | TRM |
|---|---|---|---|
| 16.7 | 14.4 | 16.6 | 14.3 |
| 17 | 14.6 | 18.7 | 16.4 |
| 17.9 | 15.9 | 17.3 | 17.7 |
| 17.9 | 15.9 | 18.3 | 18.7 |
| 17.9 | 18.7 | 18.3 | 18.7 |
| 18.7 | 18.7 | 18.3 | 16.1 |
| 18.7 | 19.3 | 18 | 16.1 |
| 18.7 | 19.3 | 18 | 16.3 |
| 18.7 | 20.3 | 18 | 16.3 |
| 18.7 | 18 | 18 | 17.1 |
| 19.4 | 18 | 19 | 17.1 |
| 17.1 | 18.1 | 17.3 | 17.3 |
|  | 18.1 | 17.3 | 19 |
|  |  |  | 19.1 |
|  |  |  | 19.1 |

# Breathing during sleep

Tidal volume in NREMS (µl)

| CLF | CLM | TRF | TRM |
|---|---|---|---|
| 189.75 | 219.14 | 260.92 | 250.93 |
| 208.38 | 220.4 | 203.96 | 216.57 |
| 232.42 | 343.1 | 203.62 | 277.35 |
| 229.49 | 268.9 | 239.3 | 191.26 |
| 216.96 | 196.61 | 188.72 | 171.24 |
| 302.56 | 178.1 | 208.87 | 286.7 |
| 161.76 | 105.76 | 206.12 | 197.43 |
| 184.92 | 272.88 | 140.29 | 198.48 |
| 165.89 | 292.51 | 255.03 | 201.15 |
| 247.64 | 259.98 | 251.79 | 207.14 |
| 192.85 | 197.3 | 290.72 | 175.67 |

| | | | |
|---|---|---|---|
| 156.91 | 186.03 | 166.45 | 134.84 |
| | 224.64 | 223.41 | 191.4 |
| | | | 181.83 |
| | | | 213.38 |

Tidal volume in REMS (μl)

| CLF | CLM | TRF | TRM |
|---|---|---|---|
| 154.63 | 175.65 | 210.62 | 185.06 |
| 165.94 | 183.25 | 172.43 | 164.23 |
| 191.84 | 275.73 | 167.07 | 220.72 |
| 194.16 | 224.86 | 198.33 | 157.48 |
| 178.95 | 181.54 | 155.87 | 138.29 |
| 234.9 | 149.2 | 184.94 | 236.65 |
| 149.48 | 84.75 | 158.94 | 148.5 |
| 155.32 | 213.66 | 118.99 | 155.49 |
| 149.42 | 224.57 | 209.87 | 153.88 |
| 182.08 | 186.79 | 199.8 | 156.5 |
| 162.6 | 162.48 | 222.6 | 161.91 |
| 137.6 | 155.69 | 132.5 | 111.9 |
| | 193.25 | 183.31 | 145.28 |
| | | | 176.93 |
| | | | 156 |

Minute ventilation in NREMS (ml $min^{-1}$)

| CLF | CLM | TRF | TRM |
|---|---|---|---|
| 32.08 | 33.22 | 36.56 | 37.53 |
| 32.85 | 39.07 | 32.32 | 33.1 |
| 39.65 | 59.02 | 33.98 | 39.92 |
| 38.72 | 47.7 | 39.66 | 30.14 |
| 31.76 | 34.13 | 30.28 | 30.64 |
| 42.5 | 31.43 | 34.7 | 50.13 |
| 21.14 | 21.45 | 33.14 | 35.64 |

| | | | |
|---|---|---|---|
| 33.01 | 50.13 | 22.79 | 35.88 |
| 25.82 | 50.6 | 38.59 | 35.76 |
| 32.55 | 50.77 | 34.42 | 36.54 |
| 28.07 | 37.49 | 38.69 | 34.92 |
| 29.72 | 29.54 | 24.16 | 22.67 |
| | 37.12 | 31.57 | 30.49 |
| | | | 33.02 |
| | | | 30.1 |

Minute ventilation in REMS (ml $min^{-1}$)

| **CLF** | **CLM** | **TRF** | **TRM** |
|---|---|---|---|
| 29.2 | 24.18 | 32.39 | 32.39 |
| 27.05 | 35.8 | 26.31 | 26.31 |
| 35.26 | 53.24 | 31.7 | 31.7 |
| 36.4 | 44.09 | 34.97 | 34.97 |
| 28.52 | 35.39 | 29.25 | 29.25 |
| 40.12 | 27.29 | 33.17 | 33.17 |
| 20.83 | 18.02 | 27.13 | 27.13 |
| 30.66 | 38.23 | 20.12 | 20.12 |
| 25.02 | 43.86 | 34.01 | 34.01 |
| 27.73 | 40.61 | 32.11 | 32.11 |
| 28.57 | 31.33 | 41.2 | 41.2 |
| 25.87 | 28.51 | 22.81 | 22.81 |
| | 35.84 | 26.23 | 26.23 |
| | | | 27.8 |
| | | | 25.34 |

Ventilatory period in NREMS (ms)

| **CLF** | **CLM** | **TRF** | **TRM** |
|---|---|---|---|
| 368.81 | 412.5 | 453.49 | 408.37 |
| 395.63 | 347.91 | 386.68 | 402.78 |
| 364.53 | 353.75 | 373.62 | 434.34 |

| | | | |
|---|---|---|---|
| 368.1 | 342.96 | 376.29 | 393.39 |
| 423.8 | 354.49 | 389.69 | 349.68 |
| 440.37 | 346.51 | 373.18 | 351.98 |
| 475.3 | 297.74 | 389.33 | 340.02 |
| 346.12 | 332.72 | 392.09 | 350.99 |
| 402.88 | 353.26 | 410.78 | 345.63 |
| 468.18 | 314.03 | 462.58 | 348.99 |
| 429.05 | 325.64 | 469.02 | 305.76 |
| 325.35 | 395.14 | 429.58 | 365.87 |
| | 372.1 | 435.53 | 391.54 |
| | | | 341.59 |
| | | | 443.08 |
| | | | |

Ventilatory period in REMS (ms)

| CLF | CLM | TRF | TRM |
|---|---|---|---|
| 338.5 | 452.25 | 409.59 | 355.18 |
| 383.4 | 320.41 | 407.89 | 319.95 |
| 342.26 | 320.96 | 333.75 | 426.66 |
| 335.99 | 317.09 | 355.76 | 352.78 |
| 403.88 | 324.44 | 348.93 | 388.13 |
| 369.75 | 338.22 | 348.99 | 347.33 |
| 497.78 | 287.72 | 367.04 | 316.08 |
| 316.01 | 349.01 | 377.32 | 351.89 |
| 381.79 | 319.03 | 383.81 | 314.23 |
| 414.81 | 282.27 | 395.61 | 297.91 |
| 365.11 | 320.86 | 349.27 | 322.54 |
| 335.83 | 347.05 | 372.32 | 301.49 |
| | 341.26 | 441.48 | 342.33 |
| | | | 399.83 |
| | | | 393.63 |

## Breathing during sleep

Number of sighs per h of NREMS

| CLF | CLM | TRF | TRM |
|---|---|---|---|
| 11.00 | 20.11 | 12.72 | 5.59 |
| 10.40 | 18.95 | 3.40 | 5.27 |
| 9.09 | 8.55 | 19.63 | 16.13 |
| 18.67 | 5.58 | 10.72 | 11.53 |
| 5.50 | 0 | 14.44 | 21.37 |
| 19.72 | 0.81 | 15.11 | 28.19 |
| 0.76 | 18.52 | 19.75 | 16.45 |
| 11.14 | 20.80 | 33.41 | 24.11 |
| 8.46 | 6.52 | 6.76 | 26.23 |
| 19.57 | 14.61 | 9.34 | 23.88 |
| 21.98 | 21.26 | 10.04 | 21.77 |
| 20.49 | 18.20 | 28.10 | 18.94 |
| | 7.05 | 17.08 | 18.59 |
| | | | 18.38 |
| | | | 24.40 |

Apnoea occurrence rate per h of NREMS

| CLF | CLM | TRF | TRM |
|---|---|---|---|
| 7.56 | 19.14 | 17.75 | 1.86 |
| 6.78 | 8.39 | 7.93 | 2.2 |
| 12.87 | 0.68 | 13.71 | 8.82 |
| 1.78 | 2.79 | 16.47 | 7.03 |
| 8.43 | 0 | 21.52 | 19.39 |
| 0 | 0 | 14.14 | 3.8 |
| 3.78 | 1 | 4.94 | 2.68 |
| 1.39 | 3.7 | 10.55 | 25.99 |
| 11.85 | 1.5 | 11.63 | 2.48 |
| 0.49 | 2.21 | 8.1 | 3.04 |
| 1.67 | 3.86 | 6.49 | 2.72 |
| 11.28 | 7.53 | 12.04 | 1.2 |
| | 2.64 | 5.07 | 23.08 |
| | | | 17.67 |
| | | | 2.91 |

Apnoea occurrence rate per h of REMS

| CLF | CLM | TRF | TRM |
|---|---|---|---|
| 0 | 6.87 | 12.44 | 9.51 |
| 6.73 | 2.14 | 12.74 | 8.71 |
| 2.81 | 5.88 | 8.51 | 21.3 |
| 6.69 | 0 | 5.59 | 6.43 |
| 0 | 14.29 | 32.46 | 10.76 |
| 0 | 16.67 | 2.12 | 0 |

| | | | |
|---|---|---|---|
| 0 | 10.59 | 8.39 | 12.08 |
| 4.12 | 0 | 9.82 | 25.71 |
| 4.06 | 0 | 4.86 | 33.67 |
| 3.72 | 0 | 12.83 | 19.01 |
| 12.86 | 0 | 11.25 | 5.52 |
| 8.54 | 3.47 | 3.46 | 12.86 |
| | 3.04 | 14.8 | 9.28 |
| | | | 52.94 |
| | | | 3.49 |

# Sleep architecture during baseline home-cage recordings

% W in light and dark

| **CLF** | | **CLM** | | **TRF** | | **TRM** | |
|---|---|---|---|---|---|---|---|
| Light | Dark | Light | Dark | Light | Dark | Light | Dark |
| 40.4 | 82.4 | 47.3 | 65.2 | 35 | 76 | 40.8 | 64.1 |
| 35.1 | 72.5 | 37.7 | 64 | 40 | 89.7 | 38.4 | 62.5 |
| 40.5 | 61.7 | 31.2 | 63.5 | 35.4 | 67.3 | 28.3 | 53.7 |
| 28 | 67.5 | 38.6 | 62.6 | 28.8 | 71 | 36.3 | 64 |
| 36 | 81.7 | 27.6 | 43.4 | 36.6 | 83.9 | 31.9 | 57.8 |
| 33.1 | 79 | 46.8 | 67.2 | 32.2 | 75.4 | 29 | 59.8 |
| 43.4 | 59.8 | 36.6 | 58.6 | 42.1 | 82 | 36 | 63.9 |
| 34.1 | 84.8 | 37.2 | 82.4 | 30.4 | 76.9 | 42.2 | 56.3 |
| 43.6 | 83.9 | 13.2 | 30.2 | 44.7 | 85.3 | 44.3 | 67.5 |
| 33.3 | 87.2 | 35.2 | 62.2 | 39.2 | 74 | 36.9 | 66.1 |
| 39.1 | 82.3 | 30.7 | 86.6 | 35.5 | 64.2 | 26.9 | 48.7 |
| 28.7 | 73.1 | | | 15.2 | 54 | 38.2 | 72.8 |
| | | | | 29.8 | 86.8 | 30.8 | 69.2 |
| | | | | | | 37.7 | 53.3 |
| | | | | | | 21.4 | 30.6 |

% NREMS in light and dark

| **CLF** | | **CLM** | | **TRF** | | **TRM** | |
|---|---|---|---|---|---|---|---|
| Light | Dark | Light | Dark | Light | Dark | Light | Dark |
| 48.1 | 14.9 | 47 | 30.6 | 53.2 | 20.1 | 51.3 | 30.4 |
| 53.5 | 23.3 | 53 | 28.2 | 46.4 | 8.7 | 54.4 | 35.8 |
| 48.9 | 30 | 56.4 | 29.7 | 53 | 26 | 57.3 | 32.8 |
| 56.9 | 23.4 | 49 | 25.8 | 56.8 | 22 | 55.8 | 30.8 |
| 50.4 | 15.4 | 59.1 | 47.6 | 53.7 | 13.2 | 50.9 | 32.8 |
| 58.3 | 18.3 | 45 | 26.8 | 62.6 | 22.4 | 62.1 | 34.4 |

| | | | | | | | |
|---|---|---|---|---|---|---|---|
| 51.5 | 35.8 | 47.1 | 30.1 | 52.6 | 16.5 | 53.7 | 29.8 |
| 53.1 | 12.8 | 50.5 | 14.1 | 57.4 | 18.4 | 47.9 | 36.7 |
| 49.9 | 14.2 | 79.1 | 62.4 | 45.6 | 12.9 | 44.2 | 27.5 |
| 61.2 | 11.5 | 46.3 | 27 | 51.1 | 23 | 54 | 28.8 |
| 47.7 | 14.5 | 52.9 | 10.3 | 48.6 | 28.9 | 32.9 | 19.5 |
| 55.3 | 21.7 | | | 62.9 | 24.7 | 49.1 | 22.1 |
| | | | | 60.7 | 11.3 | 53 | 23.1 |
| | | | | | | 52.8 | 40 |
| | | | | | | 67.9 | 60 |

% REMS in light and dark

| **CLF** | | **CLM** | | **TRF** | | **TRM** | |
|---|---|---|---|---|---|---|---|
| Light | Dark | Light | Dark | Light | Dark | Light | Dark |
| 9.7 | 1.8 | 3.7 | 2 | 9.1 | 2.5 | 4.9 | 2.5 |
| 8.1 | 1.8 | 4.3 | 1.3 | 12.1 | 1.4 | 7.2 | 1.7 |
| 4.2 | 2.1 | 8.1 | 2.7 | 7.6 | 2.9 | 7.1 | 3.1 |
| 8.1 | 2.3 | 6 | 3.7 | 10.4 | 3.1 | 4.9 | 1.7 |
| 11.6 | 2.1 | 10.4 | 4.5 | 7.6 | 1.3 | 10 | 3.4 |
| 5.3 | 1 | 5.7 | 3.5 | 2.9 | 0.4 | 6 | 1.8 |
| 1.8 | 1.8 | 10.7 | 5.9 | 5.3 | 1.4 | 6.5 | 1.4 |
| 10.5 | 1.7 | 10 | 2.8 | 9.1 | 1.8 | 7.5 | 4.1 |
| 4.6 | 0.9 | 4.3 | 1.6 | 7.8 | 1.1 | 7.3 | 2.6 |
| 3.8 | 0.4 | 14.1 | 5.4 | 8.7 | 2.4 | 6.3 | 2.5 |
| 11.2 | 2.5 | 13.4 | 1.3 | 12.1 | 4.7 | 9.8 | 3.9 |
| 13.4 | 3.5 | | | 13.5 | 3.2 | 10.5 | 4.2 |
| | | | | 5.8 | 0.6 | 10.9 | 2.5 |
| | | | | | | 6.1 | 2.7 |
| | | | | | | 9 | 7.1 |

Number of episodes in W

| **CLF** | | **CLM** | | **TRF** | | **TRM** | |
|---|---|---|---|---|---|---|---|
| Light | Dark | Light | Dark | Light | Dark | Light | Dark |
| 113 | 64 | 160 | 203 | 159 | 85 | 168 | 167 |
| 219 | 200 | 221 | 343 | 133 | 40 | 235 | 229 |
| 246 | *349* | 224 | 250 | 217 | 237 | 243 | 412 |
| 286 | *353* | 308 | 381 | 264 | 287 | 170 | 197 |
| 162 | 66 | 297 | 463 | 198 | 171 | 225 | 281 |
| 187 | 114 | 193 | 182 | 164 | 133 | 248 | 308 |
| 135 | 130 | 266 | 313 | 199 | 76 | 164 | 266 |
| 137 | 57 | 258 | 81 | 132 | 177 | 188 | 215 |
| 154 | 79 | 238 | 413 | 140 | 68 | 318 | 173 |
| 182 | 107 | 174 | 205 | 144 | 72 | 172 | 180 |
| 139 | 76 | 162 | 138 | 187 | 147 | 243 | 384 |
| 174 | 128 | | | 312 | *750* | 179 | 103 |
| | | | | 201 | 89 | 237 | 248 |

| | | | | | | | |
|---|---|---|---|---|---|---|---|
| | | | | | | 215 | 335 |
| | | | | | | 206 | 284 |

Numbers in *italics* have been considered outliers (ROUT method, Q value set at 1%, with GraphPad Prism), hence have not been included in the analysis.

Number of episodes in NREMS

| **CLF** | | **CLM** | | **TRF** | | **TRM** | |
|---|---|---|---|---|---|---|---|
| Light | Dark | Light | Dark | Light | Dark | Light | Dark |
| 569 | 186 | 542 | 422 | 586 | 222 | 693 | 492 |
| 565 | 258 | 511 | 290 | 604 | 108 | 749 | 501 |
| 449 | 325 | 610 | 360 | 575 | 290 | 460 | 302 |
| 588 | 247 | 482 | 305 | 570 | 249 | 604 | 546 |
| 639 | 234 | 531 | 512 | 638 | 243 | 464 | 354 |
| 655 | 232 | 580 | 408 | 466 | 210 | 689 | 464 |
| 313 | 245 | 592 | 394 | 652 | 210 | 601 | 393 |
| 528 | 124 | 710 | 165 | 495 | 192 | 593 | 498 |
| 696 | 184 | 470 | 435 | 679 | 181 | 674 | 389 |
| 618 | 157 | 595 | 404 | 649 | 272 | 499 | 304 |
| 522 | 153 | 804 | 192 | 660 | 351 | 234 | 152 |
| 432 | 171 | | | 552 | 241 | 594 | 271 |
| | | | | 485 | 109 | 549 | 219 |
| | | | | | | 508 | 535 |
| | | | | | | 449 | 468 |

Number of episodes in REMS

| **CLF** | | **CLM** | | **TRF** | | **TRM** | |
|---|---|---|---|---|---|---|---|
| Light | Dark | Light | Dark | Light | Dark | Light | Dark |
| 178 | 44 | 106 | 45 | 160 | 37 | 131 | 60 |
| 163 | 42 | 102 | 35 | 187 | 21 | 132 | 36 |
| 106 | 46 | 187 | 61 | 131 | 56 | 145 | 57 |
| 190 | 44 | 126 | 68 | 147 | 51 | 130 | 40 |
| 177 | 42 | 186 | 90 | 112 | 19 | 202 | 70 |
| 146 | 23 | 128 | 68 | 76 | 15 | 161 | 43 |
| 30 | 37 | 220 | 124 | 76 | 25 | 159 | 27 |
| 205 | 33 | 203 | 45 | 178 | 35 | 133 | 83 |
| 120 | 20 | 121 | 50 | 163 | 32 | 154 | 46 |
| 98 | 8 | 256 | 119 | 215 | 62 | 160 | 52 |
| 157 | 36 | 251 | 27 | 248 | 108 | 130 | 38 |
| 187 | 28 | | | 244 | 48 | 177 | 64 |
| | | | | 102 | 12 | 216 | 41 |
| | | | | | | 123 | 50 |
| | | | | | | 167 | *129* |

Duration of Wakefulness (s)

| CLF | | CLM | | TRF | | TRM | |
|---|---|---|---|---|---|---|---|
| Light | Dark | Light | Dark | Light | Dark | Light | Dark |
| 279 | 1112 | 138 | 158 | 173 | 772 | 195 | 316 |
| 126 | 310 | 111 | 213 | 202 | 1988 | 133 | 223 |
| 136 | 148 | 102 | 138 | 127 | 239 | 92 | 109 |
| 78 | 162 | 75 | 78 | 86 | 209 | 154 | 267 |
| 176 | 1034 | 188 | 309 | 136 | 427 | 116 | 173 |
| 136 | 594 | 108 | 159 | 160 | 479 | 91 | 159 |
| 263 | 393 | 116 | 863 | 133 | 965 | 169 | 201 |
| 198 | 1261 | 43 | 60 | 185 | 370 | 176 | 215 |
| 227 | 906 | 165 | 253 | 246 | 1071 | 110 | 331 |
| 143 | 700 | 137 | 532 | 219 | 868 | 172 | 310 |
| 203 | 979 | | | 159 | 351 | 89 | 107 |
| 131 | 488 | | | 36 | 59 | 151 | 635 |
| | | | | 117 | 839 | 104 | 238 |
| | | | | | | 142 | 131 |
| | | | | | | 85 | 89 |

Duration of NREMS (s)

| CLF | | CLM | | TRF | | TRM | |
|---|---|---|---|---|---|---|---|
| Light | Dark | Light | Dark | Light | Dark | Light | Dark |
| 72 | 68 | 74 | 62 | 78 | 77 | 63 | 53 |
| 81 | 77 | 89 | 82 | 65 | 69 | 62 | 61 |
| 93 | 78 | 79 | 70 | 79 | 76 | 106 | 92 |
| 82 | 80 | 86 | 70 | 84 | 73 | 79 | 48 |
| 67 | 56 | 95 | 78 | 72 | 46 | 93 | 78 |
| 76 | 67 | 66 | 55 | 115 | 91 | 77 | 63 |
| 142 | 125 | 67 | 64 | 69 | 67 | 76 | 64 |
| 86 | 87 | 60 | 73 | 99 | 81 | 69 | 63 |
| 62 | 66 | 144 | 122 | 57 | 61 | 55 | 60 |
| 85 | 63 | 66 | 56 | 67 | 72 | 92 | 81 |
| 78 | 81 | 55 | 45 | 62 | 70 | 119 | 108 |
| 109 | 109 | | | 96 | 82 | 70 | 70 |
| | | | | 107 | 88 | 82 | 89 |
| | | | | | | 89 | 63 |
| | | | | | | 130 | 109 |

Duration of REMS (s)

| CLF | | CLM | | TRF | | TRM | |
|---|---|---|---|---|---|---|---|

| Light | Dark | Light | Dark | Light | Dark | Light | Dark |
|---|---|---|---|---|---|---|---|
| 46 | 34 | 28 | 34 | 48 | 57 | 30 | 34 |
| 42 | 34 | 33 | 31 | 54 | 55 | 46 | 41 |
| 32 | 35 | 35 | 35 | 48 | 44 | 41 | 45 |
| 35 | 40 | 39 | 45 | 59 | 49 | 32 | 35 |
| 55 | 42 | 46 | 39 | 57 | 59 | 41 | 38 |
| 28 | 35 | 37 | 42 | 31 | 20 | 30 | 33 |
| 50 | 42 | 40 | 39 | 59 | 47 | 32 | 39 |
| 42 | 42 | 40 | 52 | 42 | 43 | 47 | 41 |
| 31 | 35 | 28 | 24 | 39 | 29 | 38 | 47 |
| 32 | 34 | 45 | 37 | 33 | 31 | 32 | 38 |
| 60 | 59 | 44 | 38 | 40 | 35 | 64 | *84* |
| 60 | *104* | | | 45 | 49 | 49 | 54 |
| | | | | 48 | 37 | 41 | 47 |
| | | | | | | 41 | 44 |
| | | | | | | 45 | 46 |

24-h hourly profile of the time spent in wakefulness (%) during 6 h of sleep deprivation and the following 18 h of recovery

| **CLF** | | | | | | | | | | | | |
|---|---|---|---|---|---|---|---|---|---|---|---|---|
| h | | | | | | | | | | | | |
| 1 | 100 | 100 | 97.67 | 92.68 | 99.78 | 99.22 | 97.78 | 97.11 | 100 | 97.56 | 100 | 100 |
| 2 | 100 | 100 | 96 | 88.35 | 98.67 | 97.56 | 98.67 | 97.45 | 97.11 | 99.78 | 98.78 | 100 |
| 3 | 99.78 | 99.78 | 91.45 | 86.68 | 100 | 89.46 | 99.78 | 95.56 | 94.67 | 100 | 99 | 99 |
| 4 | 99.11 | 98.22 | 85.57 | 91.12 | 97.78 | 98.67 | 99.33 | 95.45 | 99.78 | 100 | 98.56 | 97.56 |
| 5 | 98.89 | 100 | 91.9 | 97 | 100 | 84.8 | 97.56 | 91.12 | 98.89 | 98.67 | 99.45 | 99.22 |
| 6 | 100 | 100 | 92 | 96.32 | 100 | 96.64 | 99.77 | 98.73 | 99.66 | 89.29 | 98.74 | 98.4 |
| 7 | 14.43 | 38.85 | 20.98 | 44.28 | 25.64 | 20.53 | 99.78 | 15.87 | 35.52 | 15.76 | 39.73 | 22.86 |
| 8 | 15.65 | 33.07 | 12.99 | 6.44 | 42.84 | 8.66 | 99.78 | 29.19 | 28.41 | 20.98 | 23.2 | 38.85 |
| 9 | 8.1 | 7.44 | 4.22 | 11.21 | 11.1 | 24.86 | 14.32 | 8.88 | 55.72 | 16.76 | 5.55 | 4.22 |
| 10 | 47.06 | 23.86 | 45.17 | 35.74 | 41.07 | 15.87 | 1.66 | 37.29 | 7.99 | 26.53 | 30.3 | 55.72 |
| 11 | 29.3 | 41.29 | 30.74 | 24.42 | 9.32 | 44.06 | 19.76 | 12.54 | 49.17 | 46.62 | 27.08 | 22.2 |
| 12 | 76.47 | 40.07 | 42.06 | 24.31 | 43.73 | 25.75 | 98.89 | 44.17 | 68.04 | 32.96 | 47.95 | 34.07 |
| 13 | 99.78 | 87.57 | 78.02 | 63.71 | 94.78 | 90.9 | 98.11 | 87.57 | 93.56 | 53.94 | 90.46 | 97.89 |
| 14 | 25.42 | 21.64 | 57.94 | 55.49 | 21.75 | 39.62 | 99.33 | 48.95 | 30.3 | 97.23 | 44.95 | 32.3 |
| 15 | 92.68 | 82.46 | 32.41 | 19.98 | 64.37 | 56.94 | 98.34 | 99.78 | 97.34 | 29.86 | 44.28 | 81.13 |
| 16 | 99.78 | 48.84 | 71.7 | 62.6 | 67.59 | 74.7 | 97.45 | 35.52 | 49.17 | 76.8 | 98.22 | 99.78 |
| 17 | 77.03 | 99.45 | 37.74 | 47.28 | 50.94 | 99.45 | 99.33 | 100 | 99 | 65.37 | 93.01 | 41.73 |
| 18 | 13.43 | 25.53 | 73.92 | 52.61 | 69.7 | 43.06 | 98.11 | 100 | 29.63 | 23.97 | 93.01 | 32.3 |
| 19 | 100 | 71.48 | 52.5 | 54.27 | 36.96 | 61.93 | 46.06 | 100 | 39.29 | 91.9 | 7.1 | 99.78 |
| 20 | 98.89 | 84.91 | 94.56 | 37.18 | 95.12 | 80.24 | 1.55 | 99.78 | 100 | 99.33 | 46.84 | 99.67 |
| 21 | 100 | 5.55 | 97 | 87.9 | 99.33 | 6.22 | 2.11 | 34.74 | 58.49 | 100 | 86.9 | 94.78 |
| 22 | 30.19 | 69.15 | 44.84 | 58.05 | 32.19 | 40.73 | 2.44 | 54.5 | 9.77 | 56.6 | 4.11 | 27.53 |
| 23 | 49.06 | 92.12 | 39.62 | 73.03 | 75.25 | 34.74 | 5.22 | 57.6 | 34.18 | 21.09 | 36.52 | 20.98 |
| 24 | 100 | 99.75 | 79.89 | 33.44 | 74 | 100 | 9.44 | 99.74 | 100 | 99.11 | 98.15 | 99.19 |

| **CLM** | | | | | | | | | | | |
|---|---|---|---|---|---|---|---|---|---|---|---|
| h | | | | | | | | | | | |
| 1 | 100 | 93.45 | 99.78 | 98.89 | 80.02 | 100 | 99.22 | 98.56 | 83.91 | 100 | 98.34 |
| 2 | 100 | 97.78 | 98.22 | 99.33 | 96.34 | 100 | 95.56 | 92.23 | 88.79 | 100 | 99.33 |
| 3 | 100 | 99 | 100 | 99.56 | 97.11 | 99.56 | 96.56 | 94.45 | 78.91 | 98.67 | 96.67 |
| 4 | 99 | 98.34 | 99.78 | 99.67 | 95.34 | 100 | 94.78 | 95.56 | 81.02 | 99.11 | 97.34 |
| 5 | 99.56 | 99.78 | 98.56 | 98.22 | 82.46 | 99.56 | 97.45 | 94.67 | 77.8 | 97.11 | 98.56 |
| 6 | 98.52 | 93.16 | 98.41 | 99.43 | 88.39 | 98.14 | 86.58 | 96.58 | 61.55 | 95.7 | 96.19 |
| 7 | 27.86 | 30.08 | 19.76 | 24.53 | 4.55 | 11.1 | 38.74 | 15.87 | 8.44 | 15.09 | 47.5 |
| 8 | 24.75 | 15.43 | 13.54 | 20.31 | 17.43 | 29.19 | 3 | 17.43 | 20.76 | 4.33 | 6.1 |
| 9 | 16.65 | 18.65 | 24.75 | 7.21 | 9.1 | 14.98 | 26.97 | 5.88 | 9.55 | 31.85 | 17.31 |
| 10 | 17.76 | 21.87 | 20.53 | 42.29 | 27.08 | 39.07 | 21.64 | 40.73 | 30.52 | 25.86 | 24.97 |
| 11 | 27.19 | 22.42 | 40.51 | 41.95 | 20.53 | 17.98 | 25.42 | 14.65 | 31.52 | 15.76 | 64.26 |
| 12 | 53.27 | 37.63 | 37.18 | 34.3 | 17.98 | 55.72 | 39.62 | 50.17 | 20.09 | 48.39 | 56.6 |
| 13 | 90.79 | 61.6 | 87.24 | 85.46 | 88.68 | 82.02 | 57.49 | 71.14 | 74.03 | 98.34 | 96 |
| 14 | 53.5 | 54.38 | 62.71 | 54.27 | 33.41 | 90.68 | 34.63 | 63.71 | 15.32 | 34.18 | 81.69 |
| 15 | 70.7 | 47.61 | 62.6 | 71.48 | 36.4 | 57.83 | 54.38 | 66.59 | 44.06 | 49.61 | 52.83 |
| 16 | 62.04 | 63.93 | 63.71 | 81.8 | 38.4 | 98.34 | 26.19 | 51.94 | 5.55 | 96.67 | 70.81 |
| 17 | 83.57 | 57.6 | 50.39 | 55.27 | 33.3 | 42.51 | 20.64 | 85.13 | 28.41 | 7.1 | 87.9 |
| 18 | 34.74 | 10.99 | 63.82 | 28.86 | 23.42 | 53.16 | 91.23 | 89.79 | 20.42 | 82.46 | 89.35 |
| 19 | 34.74 | 48.17 | 58.49 | 90.34 | 32.19 | 82.8 | 82.02 | 9.55 | 28.64 | 66.48 | 35.41 |
| 20 | 38.4 | 42.84 | 22.31 | 37.4 | 39.18 | 26.53 | 3.33 | 46.62 | 27.3 | 19.87 | 31.3 |
| 21 | 44.06 | 25.31 | 31.41 | 23.64 | 28.08 | 36.07 | 44.51 | 50.5 | 45.95 | 34.41 | 50.06 |
| 22 | 50.06 | 47.39 | 42.73 | 19.53 | 23.86 | 35.52 | 45.06 | 52.39 | 19.42 | 36.63 | 91.79 |
| 23 | 35.74 | 74.25 | 63.82 | 34.41 | 54.05 | 53.27 | 85.02 | 96 | 7.1 | 82.02 | 86.02 |
| 24 | 87.33 | 96.77 | 58.14 | 63.75 | 73.11 | 92.56 | 86.02 | 65.82 | 32.24 | 56.19 | 69.81 |

| **TRF** | | | | | | | | | | | | | |
|---|---|---|---|---|---|---|---|---|---|---|---|---|---|
| h | | | | | | | | | | | | | |
| 1 | 85.24 | 100 | 99.56 | 99.11 | 100 | 99.78 | 99.56 | 89.79 | 96.56 | 100 | 100 | 78.8 | 100 |
| 2 | 88.9 | 99.22 | 99.45 | 99.78 | 99.67 | 100 | 100 | 99.67 | 100 | 100 | 100 | 80.58 | 100 |
| 3 | 91.23 | 99.22 | 100 | 99.78 | 100 | 98.67 | 100 | 99.56 | 100 | 99.78 | 99.22 | 82.91 | 99.11 |
| 4 | 93.45 | 100 | 99.45 | 99.56 | 99.78 | 100 | 100 | 99.78 | 100 | 100 | 100 | 90.46 | 97.89 |
| 5 | 90.34 | 99.78 | 99.67 | 98.11 | 99.45 | 99.67 | 99.89 | 99.56 | 100 | 100 | 99.78 | 84.57 | 97.11 |
| 6 | 94.99 | 98.39 | 98.09 | 99.05 | 99.76 | 99.17 | 99.88 | 98.73 | 99.24 | 100 | 98.52 | 87.76 | 97.89 |
| 7 | 11.65 | 11.21 | 25.75 | 34.41 | 20.98 | 10.43 | 35.63 | 41.29 | 45.39 | 21.09 | 18.2 | 12.99 | 6.55 |
| 8 | 0.78 | 8.55 | 21.42 | 24.08 | 20.31 | 27.3 | 14.54 | 5.77 | 7.21 | 33.41 | 20.87 | 4.11 | 7.55 |
| 9 | 25.75 | 44.28 | 3.22 | 13.1 | 12.88 | 9.1 | 38.74 | 3.55 | 59.05 | 25.64 | 14.43 | 37.18 | 13.54 |
| 10 | 22.97 | 8.32 | 29.52 | 27.64 | 40.73 | 22.09 | 26.64 | 45.62 | 7.66 | 43.29 | 44.06 | 14.65 | 5.33 |
| 11 | 8.1 | 41.51 | 28.19 | 5.44 | 30.41 | 24.53 | 16.98 | 33.85 | 49.39 | 17.54 | 41.4 | 14.1 | 39.18 |
| 12 | 12.65 | 48.17 | 47.95 | 36.52 | 32.41 | 42.4 | 58.94 | 30.74 | 48.72 | 43.06 | 40.18 | 37.74 | 33.85 |
| 13 | 72.59 | 92.34 | 95.56 | 73.81 | 91.45 | 82.58 | 95.45 | 92.45 | 91.12 | 98.67 | 80.8 | 89.46 | 84.68 |
| 14 | 64.82 | 98.78 | 96.56 | 31.41 | 52.5 | 47.28 | 59.38 | 38.4 | 100 | 56.6 | 67.81 | 54.16 | 15.87 |
| 15 | 10.32 | 84.02 | 23.53 | 42.73 | 42.62 | 21.42 | 51.39 | 39.62 | 99.78 | 40.73 | 18.98 | 48.95 | 35.85 |
| 16 | 22.53 | 27.97 | 90.9 | 25.19 | 81.35 | 57.49 | 68.04 | 60.27 | 10.66 | 87.68 | 52.5 | 89.23 | 66.7 |
| 17 | 46.17 | 98.22 | 39.18 | 70.37 | 51.39 | 72.92 | 9.21 | 20.98 | 38.62 | 26.86 | 58.05 | 44.51 | 70.92 |
| 18 | 79.58 | 100 | 21.31 | 28.97 | 40.4 | 29.3 | 98.34 | 72.81 | 100 | 100 | 67.81 | 80.24 | 57.16 |

| | | | | | | | | | | | | | |
|---|---|---|---|---|---|---|---|---|---|---|---|---|---|
| 19 | 74.81 | 100 | 72.59 | 79.69 | 98.89 | 79.36 | 87.57 | 89.9 | 100 | 95.01 | 27.97 | 14.98 | 99.33 |
| 20 | 28.19 | 100 | 95.89 | 89.79 | 71.7 | 43.62 | 65.04 | 24.31 | 100 | 55.72 | 65.93 | 98.34 | 98.78 |
| 21 | 39.4 | 89.46 | 95.89 | 91.79 | 8.55 | 19.2 | 12.65 | 39.73 | 41.73 | 100 | 63.82 | 75.25 | 27.3 |
| 22 | 11.54 | 22.64 | 50.61 | 75.25 | 73.81 | 72.48 | 99.11 | 37.85 | 36.4 | 67.7 | 99.22 | 19.09 | 13.43 |
| 23 | 37.96 | 59.16 | 55.05 | 43.06 | 39.96 | 99.33 | 99.56 | 96.12 | 86.02 | 98.89 | 99.22 | 92.23 | 56.05 |
| 24 | 7.84 | 100 | 93.79 | 56.49 | 97.67 | 89.9 | 99.04 | 96.84 | 95.15 | 76.44 | 100 | 46.81 | 84.83 |

| **TRM** | | | | | | | | | | | | | | | |
|---|---|---|---|---|---|---|---|---|---|---|---|---|---|---|---|
| h | | | | | | | | | | | | | | | |
| 1 | 98.89 | 100 | 89.01 | 100 | 99.22 | 99.78 | 100 | 100 | 99.78 | 100 | 89.12 | 100 | 99.33 | 96.78 | 98.45 |
| 2 | 99.56 | 99.45 | 92.12 | 99.8 | 100 | 99.78 | 100 | 100 | 98.89 | 100 | 89.35 | 99.2 | 96.89 | 94.34 | 90.46 |
| 3 | 99.45 | 100 | 91.45 | 100 | 100 | 99.56 | 100 | 100 | 99.11 | 99.22 | 91.23 | 100 | 95.34 | 98.45 | 89.23 |
| 4 | 100 | 99.89 | 98.45 | 98.7 | 99 | 97 | 99.67 | 100 | 99.56 | 100 | 83.69 | 99.8 | 98.56 | 99.11 | 93.9 |
| 5 | 100 | 100 | 96.45 | 100 | 97.56 | 94.45 | 100 | 98.67 | 97.23 | 100 | 83.35 | 100 | 97.45 | 98.11 | 89.79 |
| 6 | 100 | 100 | 95.24 | 98.4 | 88.28 | 92.67 | 100 | 99.18 | 98.59 | 99.21 | 84.33 | 99.8 | 96.79 | 97.65 | 86.46 |
| 7 | 11.88 | 42.62 | 2.55 | 90.3 | 24.86 | 19.87 | 45.17 | 52.39 | 7.77 | 38.85 | 2.55 | 22.3 | 9.55 | 27.75 | 17.09 |
| 8 | 36.85 | 7.77 | 16.32 | 44.3 | 26.53 | 25.86 | 31.52 | 15.54 | 6.44 | 30.08 | 23.09 | 15.7 | 7.44 | 31.52 | 21.64 |
| 9 | 5.44 | 41.73 | 27.08 | 13.5 | 19.31 | 13.65 | 17.87 | 24.42 | 44.73 | 14.98 | 16.09 | 11.3 | 15.98 | 5.99 | 14.65 |
| 10 | 45.84 | 29.19 | 15.54 | 29.3 | 35.41 | 29.41 | 24.97 | 24.53 | 34.41 | 64.04 | 29.63 | 18.9 | 23.09 | 15.65 | 29.75 |
| 11 | 23.97 | 30.3 | 27.41 | 14.4 | 22.75 | 25.53 | 40.4 | 40.73 | 32.63 | 15.65 | 9.32 | 59.4 | 23.31 | 39.18 | 3.88 |
| 12 | 56.94 | 32.85 | 38.74 | 39.2 | 36.52 | 42.84 | 37.29 | 47.73 | 59.6 | 58.27 | 24.86 | 18.2 | 41.07 | 61.82 | 43.06 |
| 13 | 64.48 | 80.69 | 63.71 | 78 | 72.25 | 67.81 | 55.38 | 53.05 | 83.35 | 93.56 | 41.84 | 100 | 82.02 | 98.34 | 36.85 |
| 14 | 67.48 | 62.04 | 34.96 | 47.6 | 50.5 | 55.72 | 39.51 | 56.49 | 47.39 | 56.27 | 36.29 | 99.8 | 30.08 | 59.49 | 33.63 |
| 15 | 65.04 | 42.84 | 41.29 | 47.1 | 57.16 | 58.38 | 57.16 | 25.97 | 43.73 | 40.62 | 38.07 | 67.2 | 65.59 | 81.13 | 38.4 |
| 16 | 55.49 | 47.06 | 70.48 | 54.2 | 32.08 | 49.39 | 47.84 | 46.06 | 78.25 | 74.03 | 57.05 | 100 | 49.06 | 50.17 | 15.65 |
| 17 | 53.83 | 35.18 | 45.62 | 52.3 | 83.91 | 61.15 | 78.8 | 42.29 | 42.51 | 93.79 | 18.2 | 93.2 | 55.05 | 46.17 | 71.7 |
| 18 | 46.17 | 37.51 | 32.52 | 45.7 | 43.95 | 74.92 | 55.05 | 45.84 | 93.12 | 44.06 | 18.87 | 19.4 | 90.79 | 94.9 | 32.96 |
| 19 | 78.91 | 42.18 | 79.13 | 43.5 | 46.17 | 67.26 | 38.18 | 27.19 | 51.05 | 12.76 | 9.88 | 31.2 | 44.84 | 48.61 | 44.95 |
| 20 | 26.64 | 69.59 | 26.42 | 63.6 | 35.74 | 38.96 | 28.08 | 37.07 | 30.86 | 34.63 | 41.84 | 99.7 | 27.97 | 45.28 | 41.62 |
| 21 | 13.1 | 58.38 | 42.29 | 23.4 | 24.53 | 28.19 | 17.31 | 34.96 | 37.85 | 19.76 | 47.84 | 17.3 | 34.07 | 77.36 | 14.21 |
| 22 | 70.7 | 45.39 | 44.84 | 34.4 | 29.75 | 32.85 | 38.62 | 39.51 | 77.91 | 34.96 | 24.53 | 28.6 | 59.27 | 54.27 | 45.06 |
| 23 | 49.17 | 71.81 | 43.4 | 90.3 | 57.94 | 39.62 | 76.25 | 63.15 | 59.93 | 66.93 | 28.75 | 66.8 | 30.63 | 98.78 | 16.65 |
| 24 | 97.14 | 61.06 | 69.44 | 73.9 | 91.33 | 57.81 | 75.79 | 79.64 | 27.73 | 100 | 35.73 | 100 | 93.27 | 20.79 | 78.58 |

## % of the time spent in W. NREMS and REMS in the recovery period

| **CLF** | | | **CLM** | | | **TRF** | | | **TRM** | | |
|---|---|---|---|---|---|---|---|---|---|---|---|
| W | NREMS | REMS | W | NREMS | REMS | W | NREMS | REMS | W | NREMS | REMS |
| 59.6 | 33.9 | 5.4 | 47.2 | 47.4 | 3.1 | 32.1 | 53.3 | 7.8 | 48 | 44.2 | 4.5 |
| 53.8 | 39.8 | 4.5 | 42.8 | 48.5 | 3.6 | 63.1 | 29.3 | 6.8 | 46.5 | 45.7 | 7.8 |
| 50.9 | 41.2 | 3.3 | 45.7 | 44.5 | 5.7 | 54.8 | 37.6 | 4.5 | 40.1 | 44.6 | 5.5 |
| 44 | 41.1 | 5.8 | 45.4 | 41.8 | 7.1 | 47.2 | 42 | 7 | 49.2 | 44.8 | 3.2 |
| 53.1 | 37.2 | 8 | 33.4 | 55.1 | 8.4 | 50.4 | 41.6 | 6.4 | 43.9 | 40.2 | 8.4 |
| 47.8 | 45.9 | 4.3 | 51.1 | 40.8 | 4.8 | 47.3 | 49.7 | 1.4 | 43.8 | 49.1 | 3.4 |
| 55.5 | 42.5 | 0.5 | 43.7 | 41.3 | 8.5 | 57.4 | 36.6 | 5.9 | 44.6 | 46.8 | 4.4 |

| | | | | | | | | | | | |
|---|---|---|---|---|---|---|---|---|---|---|---|
| 58.9 | 33.7 | 6.1 | 49.7 | 41.3 | 7.1 | 48.3 | 43.7 | 4.9 | 41.8 | 48.9 | 7 |
| 54.4 | 41 | 2.8 | 26 | 63.7 | 3.1 | 62 | 35 | 1.6 | 47.8 | 41.9 | 5.8 |
| 54.2 | 43 | 2 | 44.7 | 40 | 10.3 | 60.5 | 34.3 | 4.5 | 49.5 | 42 | 4.9 |
| 50.9 | 38.6 | 7.5 | 58.9 | 31.6 | 6.8 | 54.3 | 35.1 | 7.9 | 28 | 53.5 | 8.5 |
| 55.7 | 35.3 | 7.4 | | | | 48.6 | 39 | 7.8 | 53.7 | 37.9 | 7.2 |
| | | | | | | 45.3 | 48.6 | 3.6 | 43.3 | 42.7 | 8.2 |
| | | | | | | | | | 53.3 | 39.9 | 3.8 |
| | | | | | | | | | 33.3 | 54.4 | 8.6 |

Electroencephalogram analysis during sleep in baseline conditions

Electroencephalographic power spectral density during NREMS

| **CLF** | | | | | | | | | | | | |
|---|---|---|---|---|---|---|---|---|---|---|---|---|
| Hz | | | | | | | | | | | | |
| 0.25 | 3.59 | 1.55 | 3.48 | 1.27 | 1.38 | 2.61 | 2.63 | 5.2 | 1.89 | 3.73 | 1.49 | 1.57 |
| 0.5 | 2.7 | 2.31 | 2.7 | 1.67 | 2.38 | 2.82 | 3.25 | 2.11 | 2.51 | 1.88 | 2.63 | 2.03 |
| 0.75 | 3.63 | 3.45 | 3.58 | 2.12 | 3.32 | 3.65 | 3.6 | 2.65 | 3.49 | 2.3 | 3.79 | 2.66 |
| 1 | 4.02 | 4.15 | 4.01 | 2.58 | 3.51 | 4.11 | 3.85 | 3.01 | 3.92 | 2.83 | 4.2 | 2.87 |
| 1.25 | 3.89 | 4.44 | 4.37 | 2.87 | 3.34 | 4.23 | 3.94 | 3.18 | 4.26 | 3.06 | 3.9 | 3 |
| 1.5 | 3.86 | 4.54 | 4.3 | 3.05 | 3.26 | 4.1 | 4.1 | 3.24 | 4.21 | 3.17 | 3.69 | 3.03 |
| 1.75 | 3.64 | 4.5 | 4.13 | 3.18 | 3.19 | 3.84 | 4.01 | 3.17 | 4.15 | 3.19 | 3.42 | 2.94 |
| 2 | 3.37 | 4.62 | 3.9 | 3.27 | 3.1 | 3.58 | 4.16 | 3.1 | 3.94 | 3.16 | 3.21 | 2.98 |
| 2.25 | 3.14 | 4.33 | 3.56 | 3.26 | 3.02 | 3.37 | 4.07 | 3.11 | 3.81 | 3.14 | 3.02 | 3.02 |
| 2.5 | 3.1 | 4.17 | 3.45 | 3.28 | 3.01 | 3.29 | 4.03 | 2.98 | 3.64 | 3.1 | 2.99 | 3.22 |
| 2.75 | 2.91 | 3.91 | 3.3 | 3.31 | 3.14 | 3.13 | 4.06 | 2.84 | 3.35 | 3.01 | 2.96 | 3.11 |
| 3 | 2.91 | 3.68 | 3.08 | 3.34 | 3.16 | 2.92 | 3.86 | 2.73 | 2.96 | 2.91 | 2.9 | 2.93 |
| 3.25 | 2.66 | 3.49 | 2.93 | 3.29 | 3.22 | 2.71 | 3.91 | 2.58 | 2.81 | 2.86 | 2.73 | 2.84 |
| 3.5 | 2.55 | 3.22 | 2.67 | 3.08 | 3.13 | 2.49 | 3.74 | 2.52 | 2.58 | 2.78 | 2.6 | 2.67 |
| 3.75 | 2.3 | 2.98 | 2.37 | 2.95 | 2.82 | 2.33 | 3.7 | 2.38 | 2.29 | 2.59 | 2.35 | 2.62 |
| 4 | 2.18 | 2.68 | 2.16 | 2.73 | 2.56 | 2.18 | 3.49 | 2.3 | 2.1 | 2.43 | 2.26 | 2.54 |
| 4.25 | 2.11 | 2.34 | 1.97 | 2.64 | 2.36 | 2.02 | 3.23 | 2.15 | 2.06 | 2.23 | 2.05 | 2.4 |
| 4.5 | 1.95 | 2.1 | 1.87 | 2.47 | 2.17 | 1.97 | 2.91 | 2.11 | 1.91 | 2.08 | 1.98 | 2.32 |
| 4.75 | 1.93 | 1.86 | 1.73 | 2.35 | 2.01 | 1.85 | 2.6 | 2.05 | 1.8 | 1.96 | 1.8 | 2.23 |
| 5 | 1.83 | 1.67 | 1.65 | 2.28 | 1.9 | 1.82 | 2.32 | 1.98 | 1.73 | 1.86 | 1.77 | 2.09 |
| 5.25 | 1.79 | 1.6 | 1.59 | 2.14 | 1.77 | 1.71 | 2.12 | 1.96 | 1.62 | 1.73 | 1.65 | 2.05 |
| 5.5 | 1.69 | 1.46 | 1.57 | 2.05 | 1.75 | 1.67 | 1.84 | 1.94 | 1.65 | 1.69 | 1.62 | 1.98 |
| 5.75 | 1.61 | 1.44 | 1.5 | 2.06 | 1.64 | 1.65 | 1.69 | 1.87 | 1.55 | 1.59 | 1.57 | 1.92 |
| 6 | 1.57 | 1.4 | 1.48 | 1.94 | 1.57 | 1.62 | 1.49 | 1.83 | 1.55 | 1.54 | 1.53 | 1.88 |
| 6.25 | 1.58 | 1.39 | 1.44 | 1.89 | 1.56 | 1.64 | 1.36 | 1.77 | 1.51 | 1.53 | 1.51 | 1.83 |
| 6.5 | 1.57 | 1.38 | 1.44 | 1.83 | 1.58 | 1.62 | 1.2 | 1.79 | 1.55 | 1.5 | 1.54 | 1.8 |
| 6.75 | 1.49 | 1.31 | 1.45 | 1.77 | 1.53 | 1.62 | 1.07 | 1.7 | 1.46 | 1.51 | 1.56 | 1.77 |
| 7 | 1.46 | 1.3 | 1.37 | 1.77 | 1.56 | 1.53 | 0.95 | 1.7 | 1.39 | 1.45 | 1.54 | 1.67 |
| 7.25 | 1.41 | 1.27 | 1.32 | 1.63 | 1.53 | 1.47 | 0.88 | 1.67 | 1.39 | 1.38 | 1.53 | 1.63 |
| 7.5 | 1.36 | 1.15 | 1.26 | 1.54 | 1.5 | 1.42 | 0.81 | 1.57 | 1.33 | 1.32 | 1.51 | 1.59 |
| 7.75 | 1.34 | 1.14 | 1.17 | 1.47 | 1.42 | 1.35 | 0.72 | 1.47 | 1.28 | 1.25 | 1.47 | 1.53 |
| 8 | 1.28 | 1.06 | 1.11 | 1.39 | 1.4 | 1.25 | 0.67 | 1.4 | 1.23 | 1.2 | 1.4 | 1.45 |
| 8.25 | 1.16 | 1.02 | 1.04 | 1.31 | 1.32 | 1.19 | 0.59 | 1.35 | 1.1 | 1.1 | 1.35 | 1.34 |

| | | | | | | | | | | | | |
|---|---|---|---|---|---|---|---|---|---|---|---|---|
| 8.5 | 1.13 | 0.96 | 0.98 | 1.24 | 1.27 | 1.05 | 0.55 | 1.25 | 1.04 | 1.07 | 1.27 | 1.28 |
| 8.75 | 1.11 | 0.9 | 0.92 | 1.13 | 1.21 | 1.02 | 0.52 | 1.18 | 0.99 | 1.03 | 1.22 | 1.18 |
| 9 | 1.02 | 0.84 | 0.88 | 1.08 | 1.16 | 0.92 | 0.49 | 1.1 | 0.91 | 0.95 | 1.16 | 1.15 |
| 9.25 | 0.97 | 0.78 | 0.85 | 0.99 | 1.08 | 0.89 | 0.44 | 1.02 | 0.84 | 0.9 | 1.11 | 1.06 |
| 9.5 | 0.91 | 0.73 | 0.83 | 0.95 | 1.06 | 0.82 | 0.42 | 0.95 | 0.8 | 0.85 | 1.02 | 0.97 |
| 9.75 | 0.85 | 0.69 | 0.79 | 0.86 | 0.98 | 0.77 | 0.39 | 0.9 | 0.72 | 0.84 | 0.97 | 0.92 |
| 10 | 0.78 | 0.64 | 0.71 | 0.82 | 0.92 | 0.74 | 0.36 | 0.83 | 0.68 | 0.79 | 0.92 | 0.88 |
| 10.25 | 0.74 | 0.59 | 0.7 | 0.75 | 0.84 | 0.68 | 0.34 | 0.77 | 0.66 | 0.74 | 0.83 | 0.8 |
| 10.5 | 0.7 | 0.55 | 0.64 | 0.72 | 0.81 | 0.66 | 0.31 | 0.72 | 0.58 | 0.7 | 0.79 | 0.76 |
| 10.75 | 0.67 | 0.52 | 0.6 | 0.67 | 0.78 | 0.61 | 0.3 | 0.67 | 0.55 | 0.66 | 0.76 | 0.72 |
| 11 | 0.65 | 0.48 | 0.57 | 0.61 | 0.71 | 0.58 | 0.29 | 0.63 | 0.52 | 0.64 | 0.71 | 0.69 |
| 11.25 | 0.6 | 0.45 | 0.52 | 0.59 | 0.67 | 0.56 | 0.27 | 0.6 | 0.48 | 0.62 | 0.67 | 0.65 |
| 11.5 | 0.56 | 0.43 | 0.51 | 0.56 | 0.63 | 0.53 | 0.26 | 0.56 | 0.46 | 0.58 | 0.63 | 0.62 |
| 11.75 | 0.52 | 0.39 | 0.47 | 0.52 | 0.58 | 0.51 | 0.25 | 0.52 | 0.43 | 0.56 | 0.59 | 0.59 |
| 12 | 0.49 | 0.38 | 0.45 | 0.49 | 0.55 | 0.5 | 0.23 | 0.5 | 0.41 | 0.53 | 0.56 | 0.55 |
| 12.25 | 0.47 | 0.36 | 0.42 | 0.45 | 0.54 | 0.45 | 0.22 | 0.46 | 0.38 | 0.49 | 0.53 | 0.52 |
| 12.5 | 0.45 | 0.33 | 0.42 | 0.44 | 0.49 | 0.43 | 0.21 | 0.44 | 0.37 | 0.47 | 0.49 | 0.5 |
| 12.75 | 0.43 | 0.31 | 0.39 | 0.41 | 0.47 | 0.41 | 0.2 | 0.42 | 0.35 | 0.44 | 0.47 | 0.47 |
| 13 | 0.4 | 0.3 | 0.38 | 0.39 | 0.44 | 0.38 | 0.2 | 0.39 | 0.33 | 0.42 | 0.44 | 0.45 |
| 13.25 | 0.38 | 0.27 | 0.37 | 0.36 | 0.4 | 0.37 | 0.18 | 0.37 | 0.31 | 0.42 | 0.41 | 0.43 |
| 13.5 | 0.37 | 0.26 | 0.35 | 0.34 | 0.38 | 0.36 | 0.18 | 0.35 | 0.31 | 0.38 | 0.4 | 0.39 |
| 13.75 | 0.35 | 0.25 | 0.34 | 0.32 | 0.36 | 0.35 | 0.17 | 0.34 | 0.29 | 0.36 | 0.38 | 0.38 |
| 14 | 0.33 | 0.23 | 0.32 | 0.31 | 0.34 | 0.34 | 0.17 | 0.31 | 0.28 | 0.35 | 0.35 | 0.36 |
| 14.25 | 0.31 | 0.23 | 0.3 | 0.29 | 0.33 | 0.31 | 0.16 | 0.29 | 0.26 | 0.33 | 0.33 | 0.34 |
| 14.5 | 0.29 | 0.21 | 0.29 | 0.28 | 0.31 | 0.3 | 0.16 | 0.29 | 0.25 | 0.3 | 0.32 | 0.31 |
| 14.75 | 0.28 | 0.21 | 0.27 | 0.26 | 0.28 | 0.28 | 0.15 | 0.27 | 0.24 | 0.3 | 0.3 | 0.3 |
| 15 | 0.26 | 0.2 | 0.26 | 0.25 | 0.27 | 0.27 | 0.14 | 0.26 | 0.23 | 0.28 | 0.28 | 0.29 |
| 15.25 | 0.25 | 0.19 | 0.25 | 0.23 | 0.25 | 0.26 | 0.14 | 0.25 | 0.22 | 0.27 | 0.27 | 0.27 |
| 15.5 | 0.23 | 0.18 | 0.24 | 0.22 | 0.23 | 0.24 | 0.14 | 0.23 | 0.21 | 0.26 | 0.26 | 0.26 |
| 15.75 | 0.22 | 0.17 | 0.22 | 0.22 | 0.22 | 0.22 | 0.14 | 0.22 | 0.2 | 0.26 | 0.24 | 0.24 |
| 16 | 0.21 | 0.16 | 0.21 | 0.2 | 0.21 | 0.22 | 0.13 | 0.2 | 0.19 | 0.24 | 0.23 | 0.22 |
| 16.25 | 0.2 | 0.15 | 0.21 | 0.19 | 0.2 | 0.21 | 0.13 | 0.2 | 0.18 | 0.22 | 0.22 | 0.21 |
| 16.5 | 0.19 | 0.15 | 0.19 | 0.18 | 0.19 | 0.19 | 0.12 | 0.19 | 0.17 | 0.22 | 0.2 | 0.21 |
| 16.75 | 0.18 | 0.14 | 0.18 | 0.18 | 0.17 | 0.18 | 0.12 | 0.18 | 0.16 | 0.21 | 0.19 | 0.2 |
| 17 | 0.17 | 0.13 | 0.17 | 0.17 | 0.17 | 0.17 | 0.12 | 0.17 | 0.16 | 0.2 | 0.18 | 0.19 |
| 17.25 | 0.16 | 0.12 | 0.17 | 0.16 | 0.16 | 0.17 | 0.11 | 0.17 | 0.15 | 0.19 | 0.18 | 0.18 |
| 17.5 | 0.16 | 0.11 | 0.15 | 0.15 | 0.15 | 0.16 | 0.11 | 0.16 | 0.15 | 0.18 | 0.17 | 0.17 |
| 17.75 | 0.15 | 0.11 | 0.15 | 0.14 | 0.14 | 0.15 | 0.11 | 0.15 | 0.14 | 0.18 | 0.16 | 0.16 |
| 18 | 0.14 | 0.1 | 0.14 | 0.14 | 0.14 | 0.14 | 0.11 | 0.15 | 0.14 | 0.17 | 0.15 | 0.16 |
| 18.25 | 0.13 | 0.1 | 0.14 | 0.13 | 0.14 | 0.13 | 0.11 | 0.14 | 0.13 | 0.16 | 0.14 | 0.15 |
| 18.5 | 0.13 | 0.1 | 0.14 | 0.12 | 0.13 | 0.13 | 0.1 | 0.13 | 0.12 | 0.16 | 0.14 | 0.15 |
| 18.75 | 0.12 | 0.1 | 0.14 | 0.12 | 0.12 | 0.12 | 0.1 | 0.13 | 0.12 | 0.15 | 0.13 | 0.14 |
| 19 | 0.12 | 0.09 | 0.13 | 0.11 | 0.12 | 0.12 | 0.1 | 0.12 | 0.12 | 0.15 | 0.13 | 0.13 |
| 19.25 | 0.12 | 0.09 | 0.13 | 0.11 | 0.11 | 0.11 | 0.1 | 0.12 | 0.11 | 0.14 | 0.12 | 0.13 |
| 19.5 | 0.11 | 0.08 | 0.12 | 0.11 | 0.1 | 0.11 | 0.09 | 0.11 | 0.11 | 0.14 | 0.12 | 0.12 |
| 19.75 | 0.11 | 0.08 | 0.12 | 0.1 | 0.1 | 0.1 | 0.09 | 0.11 | 0.11 | 0.13 | 0.11 | 0.12 |
| 20 | 0.11 | 0.08 | 0.11 | 0.1 | 0.1 | 0.1 | 0.09 | 0.1 | 0.1 | 0.13 | 0.11 | 0.11 |

| **CLM** | | | | | | | | | | | |
|---|---|---|---|---|---|---|---|---|---|---|---|
| Hz | | | | | | | | | | | |
| 0.25 | 1.42 | 2.72 | 1.51 | 0.87 | 1.88 | 1.04 | 2.48 | 6.46 | 3.44 | 0.91 | 2.94 |
| 0.5 | 2.07 | 2.82 | 2.48 | 1.88 | 2.39 | 1.75 | 2.57 | 2.84 | 3.75 | 1.85 | 2.25 |
| 0.75 | 2.88 | 3.74 | 3.05 | 2.9 | 2.8 | 2.38 | 2.83 | 2.92 | 4.13 | 2.61 | 3.04 |
| 1 | 3.44 | 4.09 | 3.36 | 3.51 | 3.04 | 2.83 | 2.88 | 3.15 | 4.01 | 2.79 | 3.19 |
| 1.25 | 3.58 | 4.12 | 3.39 | 3.83 | 3.03 | 3.03 | 2.71 | 2.96 | 3.74 | 2.89 | 3.18 |
| 1.5 | 3.83 | 4.05 | 3.34 | 3.98 | 3.03 | 3.09 | 2.64 | 2.82 | 3.47 | 2.87 | 3.2 |
| 1.75 | 3.84 | 3.93 | 3.27 | 3.97 | 3.03 | 3.22 | 2.65 | 2.75 | 3.24 | 2.92 | 3.14 |
| 2 | 3.73 | 3.77 | 3.17 | 3.95 | 2.91 | 3.2 | 2.63 | 2.66 | 3.04 | 2.99 | 3.06 |
| 2.25 | 3.48 | 3.64 | 3.03 | 3.87 | 2.87 | 3.26 | 2.76 | 2.63 | 2.96 | 2.94 | 2.98 |
| 2.5 | 3.48 | 3.56 | 3.01 | 3.62 | 2.98 | 3.3 | 2.93 | 2.6 | 2.94 | 2.95 | 3 |
| 2.75 | 3.27 | 3.52 | 3.01 | 3.57 | 2.99 | 3.34 | 2.9 | 2.64 | 2.99 | 2.95 | 3.04 |
| 3 | 3.09 | 3.43 | 2.88 | 3.44 | 3 | 3.19 | 2.74 | 2.55 | 3.15 | 3.01 | 2.96 |
| 3.25 | 3.09 | 3.21 | 2.73 | 3.37 | 2.95 | 3.21 | 2.67 | 2.54 | 3.16 | 2.98 | 2.93 |
| 3.5 | 2.95 | 3.04 | 2.59 | 3.12 | 2.88 | 3.17 | 2.58 | 2.37 | 2.98 | 3.11 | 2.83 |
| 3.75 | 2.81 | 2.68 | 2.53 | 2.95 | 2.75 | 2.97 | 2.46 | 2.29 | 2.77 | 3 | 2.64 |
| 4 | 2.59 | 2.45 | 2.38 | 2.75 | 2.65 | 2.81 | 2.45 | 2.22 | 2.57 | 2.86 | 2.53 |
| 4.25 | 2.47 | 2.18 | 2.26 | 2.52 | 2.42 | 2.68 | 2.29 | 2.1 | 2.3 | 2.64 | 2.35 |
| 4.5 | 2.26 | 2.01 | 2.21 | 2.35 | 2.36 | 2.52 | 2.31 | 1.97 | 2.13 | 2.54 | 2.21 |
| 4.75 | 2.17 | 1.86 | 2.13 | 2.11 | 2.24 | 2.37 | 2.2 | 1.9 | 2.04 | 2.39 | 2.15 |
| 5 | 2.12 | 1.75 | 2.05 | 2.03 | 2.11 | 2.19 | 2.17 | 1.82 | 1.96 | 2.24 | 2.05 |
| 5.25 | 2.01 | 1.6 | 2.02 | 1.97 | 2.08 | 2.08 | 2.05 | 1.84 | 1.87 | 2.16 | 1.98 |
| 5.5 | 1.9 | 1.56 | 1.97 | 1.92 | 2.02 | 2.01 | 2.05 | 1.84 | 1.81 | 2.15 | 1.98 |
| 5.75 | 1.82 | 1.51 | 1.97 | 1.91 | 2.01 | 1.88 | 1.97 | 1.78 | 1.75 | 2.06 | 1.88 |
| 6 | 1.73 | 1.45 | 1.91 | 1.92 | 1.9 | 1.85 | 1.93 | 1.79 | 1.7 | 2.05 | 1.84 |
| 6.25 | 1.74 | 1.41 | 1.87 | 1.92 | 1.85 | 1.79 | 1.86 | 1.77 | 1.63 | 2.01 | 1.78 |
| 6.5 | 1.69 | 1.37 | 1.84 | 1.96 | 1.8 | 1.7 | 1.81 | 1.72 | 1.54 | 1.92 | 1.77 |
| 6.75 | 1.65 | 1.34 | 1.86 | 1.82 | 1.76 | 1.69 | 1.79 | 1.71 | 1.44 | 1.86 | 1.71 |
| 7 | 1.54 | 1.29 | 1.76 | 1.71 | 1.68 | 1.62 | 1.69 | 1.68 | 1.42 | 1.83 | 1.69 |
| 7.25 | 1.45 | 1.25 | 1.67 | 1.62 | 1.62 | 1.53 | 1.61 | 1.55 | 1.29 | 1.76 | 1.61 |
| 7.5 | 1.41 | 1.21 | 1.57 | 1.44 | 1.5 | 1.47 | 1.56 | 1.5 | 1.22 | 1.69 | 1.52 |
| 7.75 | 1.38 | 1.16 | 1.5 | 1.33 | 1.42 | 1.43 | 1.51 | 1.4 | 1.12 | 1.57 | 1.48 |
| 8 | 1.27 | 1.1 | 1.41 | 1.22 | 1.34 | 1.32 | 1.39 | 1.37 | 1.05 | 1.44 | 1.38 |
| 8.25 | 1.2 | 1.06 | 1.31 | 1.14 | 1.29 | 1.26 | 1.31 | 1.26 | 1 | 1.43 | 1.31 |
| 8.5 | 1.1 | 0.97 | 1.24 | 1 | 1.17 | 1.17 | 1.23 | 1.15 | 0.93 | 1.31 | 1.27 |
| 8.75 | 1.02 | 0.92 | 1.18 | 0.91 | 1.1 | 1.14 | 1.18 | 1.11 | 0.87 | 1.18 | 1.14 |
| 9 | 0.97 | 0.87 | 1.08 | 0.85 | 1.04 | 1.05 | 1.11 | 1.05 | 0.84 | 1.13 | 1.05 |
| 9.25 | 0.88 | 0.8 | 1.01 | 0.77 | 0.98 | 0.98 | 1.1 | 0.98 | 0.79 | 1.04 | 1.02 |
| 9.5 | 0.83 | 0.79 | 0.97 | 0.69 | 0.9 | 0.92 | 1.04 | 0.94 | 0.75 | 0.97 | 0.94 |
| 9.75 | 0.8 | 0.73 | 0.9 | 0.66 | 0.87 | 0.85 | 0.99 | 0.89 | 0.69 | 0.9 | 0.89 |
| 10 | 0.72 | 0.7 | 0.85 | 0.6 | 0.84 | 0.82 | 0.93 | 0.84 | 0.66 | 0.83 | 0.81 |
| 10.25 | 0.69 | 0.64 | 0.78 | 0.55 | 0.78 | 0.78 | 0.87 | 0.78 | 0.62 | 0.77 | 0.78 |
| 10.5 | 0.64 | 0.58 | 0.75 | 0.52 | 0.72 | 0.72 | 0.83 | 0.75 | 0.59 | 0.74 | 0.72 |
| 10.75 | 0.59 | 0.56 | 0.7 | 0.48 | 0.68 | 0.7 | 0.79 | 0.72 | 0.57 | 0.69 | 0.66 |
| 11 | 0.56 | 0.53 | 0.65 | 0.44 | 0.64 | 0.65 | 0.75 | 0.65 | 0.54 | 0.61 | 0.63 |
| 11.25 | 0.52 | 0.5 | 0.6 | 0.42 | 0.61 | 0.61 | 0.7 | 0.62 | 0.5 | 0.61 | 0.59 |
| 11.5 | 0.51 | 0.47 | 0.58 | 0.4 | 0.56 | 0.55 | 0.66 | 0.58 | 0.49 | 0.58 | 0.57 |

| | | | | | | | | | | | |
|---|---|---|---|---|---|---|---|---|---|---|---|
| 11.75 | 0.48 | 0.45 | 0.54 | 0.37 | 0.52 | 0.53 | 0.63 | 0.57 | 0.45 | 0.54 | 0.53 |
| 12 | 0.44 | 0.44 | 0.52 | 0.35 | 0.5 | 0.5 | 0.6 | 0.52 | 0.42 | 0.5 | 0.49 |
| 12.25 | 0.42 | 0.43 | 0.48 | 0.34 | 0.47 | 0.47 | 0.54 | 0.49 | 0.4 | 0.45 | 0.48 |
| 12.5 | 0.42 | 0.39 | 0.45 | 0.31 | 0.44 | 0.46 | 0.52 | 0.46 | 0.38 | 0.44 | 0.44 |
| 12.75 | 0.38 | 0.37 | 0.43 | 0.3 | 0.41 | 0.43 | 0.48 | 0.44 | 0.36 | 0.42 | 0.41 |
| 13 | 0.36 | 0.36 | 0.4 | 0.28 | 0.39 | 0.4 | 0.47 | 0.41 | 0.35 | 0.38 | 0.38 |
| 13.25 | 0.35 | 0.34 | 0.38 | 0.27 | 0.37 | 0.39 | 0.44 | 0.39 | 0.33 | 0.36 | 0.38 |
| 13.5 | 0.33 | 0.32 | 0.37 | 0.26 | 0.36 | 0.38 | 0.41 | 0.37 | 0.32 | 0.34 | 0.35 |
| 13.75 | 0.32 | 0.31 | 0.35 | 0.25 | 0.34 | 0.36 | 0.39 | 0.35 | 0.3 | 0.32 | 0.33 |
| 14 | 0.3 | 0.29 | 0.33 | 0.23 | 0.32 | 0.34 | 0.37 | 0.33 | 0.29 | 0.3 | 0.31 |
| 14.25 | 0.29 | 0.28 | 0.32 | 0.23 | 0.31 | 0.32 | 0.35 | 0.32 | 0.28 | 0.29 | 0.29 |
| 14.5 | 0.28 | 0.26 | 0.3 | 0.21 | 0.29 | 0.31 | 0.32 | 0.3 | 0.27 | 0.28 | 0.28 |
| 14.75 | 0.26 | 0.26 | 0.28 | 0.2 | 0.28 | 0.29 | 0.3 | 0.28 | 0.26 | 0.26 | 0.27 |
| 15 | 0.25 | 0.23 | 0.27 | 0.19 | 0.26 | 0.28 | 0.29 | 0.27 | 0.25 | 0.25 | 0.25 |
| 15.25 | 0.24 | 0.23 | 0.25 | 0.18 | 0.25 | 0.26 | 0.27 | 0.26 | 0.24 | 0.23 | 0.25 |
| 15.5 | 0.22 | 0.22 | 0.24 | 0.17 | 0.23 | 0.25 | 0.26 | 0.25 | 0.22 | 0.22 | 0.23 |
| 15.75 | 0.21 | 0.21 | 0.23 | 0.16 | 0.22 | 0.24 | 0.25 | 0.24 | 0.21 | 0.21 | 0.22 |
| 16 | 0.21 | 0.2 | 0.21 | 0.16 | 0.21 | 0.23 | 0.24 | 0.23 | 0.21 | 0.21 | 0.2 |
| 16.25 | 0.19 | 0.19 | 0.21 | 0.16 | 0.2 | 0.21 | 0.23 | 0.22 | 0.2 | 0.2 | 0.19 |
| 16.5 | 0.18 | 0.18 | 0.19 | 0.14 | 0.19 | 0.2 | 0.22 | 0.2 | 0.19 | 0.18 | 0.19 |
| 16.75 | 0.18 | 0.17 | 0.18 | 0.14 | 0.18 | 0.19 | 0.21 | 0.19 | 0.18 | 0.18 | 0.18 |
| 17 | 0.17 | 0.17 | 0.17 | 0.14 | 0.18 | 0.18 | 0.2 | 0.19 | 0.17 | 0.17 | 0.17 |
| 17.25 | 0.16 | 0.16 | 0.16 | 0.13 | 0.17 | 0.17 | 0.18 | 0.18 | 0.16 | 0.16 | 0.16 |
| 17.5 | 0.15 | 0.15 | 0.16 | 0.12 | 0.16 | 0.17 | 0.18 | 0.17 | 0.16 | 0.15 | 0.15 |
| 17.75 | 0.14 | 0.15 | 0.15 | 0.12 | 0.16 | 0.15 | 0.17 | 0.17 | 0.15 | 0.14 | 0.15 |
| 18 | 0.14 | 0.14 | 0.15 | 0.12 | 0.15 | 0.15 | 0.16 | 0.16 | 0.15 | 0.14 | 0.14 |
| 18.25 | 0.13 | 0.13 | 0.14 | 0.11 | 0.14 | 0.14 | 0.15 | 0.15 | 0.14 | 0.13 | 0.13 |
| 18.5 | 0.12 | 0.12 | 0.13 | 0.11 | 0.13 | 0.13 | 0.15 | 0.14 | 0.14 | 0.13 | 0.13 |
| 18.75 | 0.12 | 0.12 | 0.13 | 0.11 | 0.13 | 0.13 | 0.14 | 0.14 | 0.13 | 0.12 | 0.12 |
| 19 | 0.11 | 0.12 | 0.12 | 0.1 | 0.12 | 0.12 | 0.14 | 0.13 | 0.12 | 0.12 | 0.12 |
| 19.25 | 0.11 | 0.12 | 0.12 | 0.1 | 0.12 | 0.12 | 0.13 | 0.13 | 0.12 | 0.11 | 0.12 |
| 19.5 | 0.1 | 0.11 | 0.11 | 0.1 | 0.12 | 0.11 | 0.13 | 0.12 | 0.12 | 0.11 | 0.11 |
| 19.75 | 0.1 | 0.1 | 0.11 | 0.09 | 0.11 | 0.11 | 0.12 | 0.12 | 0.11 | 0.11 | 0.11 |
| 20 | 0.1 | 0.1 | 0.1 | 0.09 | 0.11 | 0.11 | 0.12 | 0.12 | 0.11 | 0.1 | 0.1 |

| **TRF** | | | | | | | | | | | | | |
|---|---|---|---|---|---|---|---|---|---|---|---|---|---|
| Hz | | | | | | | | | | | | | |
| 0.25 | 1.28 | 1.04 | 1.4 | 1.39 | 2.22 | 7.35 | 1.55 | 5.04 | 4.2 | 11.5 | 6.35 | 5.17 | 1.61 |
| 0.5 | 2.37 | 2.28 | 2.46 | 2.36 | 2.55 | 2.44 | 2.9 | 2.69 | 3.02 | 2.87 | 2.25 | 2.1 | 1.88 |
| 0.75 | 3.22 | 3.23 | 3.2 | 3.28 | 3.47 | 2.88 | 4.24 | 3.18 | 3.66 | 2.74 | 2.52 | 2.42 | 2.51 |
| 1 | 3.67 | 3.59 | 3.59 | 3.75 | 4.09 | 3.25 | 4.89 | 3.59 | 3.7 | 2.86 | 2.97 | 2.72 | 3.15 |
| 1.25 | 3.82 | 3.6 | 3.69 | 3.9 | 4.35 | 3.55 | 4.87 | 3.79 | 3.42 | 2.94 | 3.02 | 3.01 | 3.51 |
| 1.5 | 3.7 | 3.49 | 3.66 | 3.92 | 4.32 | 3.58 | 4.74 | 3.71 | 3.21 | 2.76 | 3.13 | 3.12 | 3.85 |
| 1.75 | 3.72 | 3.35 | 3.57 | 3.81 | 4.29 | 3.71 | 4.53 | 3.63 | 3.09 | 2.74 | 3.09 | 3.14 | 4.05 |
| 2 | 3.65 | 3.26 | 3.55 | 3.54 | 3.98 | 3.54 | 4.29 | 3.5 | 2.9 | 2.55 | 3.07 | 3.09 | 4.34 |
| 2.25 | 3.66 | 3.2 | 3.36 | 3.45 | 3.74 | 3.52 | 4.05 | 3.24 | 2.81 | 2.45 | 2.92 | 3 | 4.45 |
| 2.5 | 3.74 | 3.2 | 3.34 | 3.33 | 3.64 | 3.41 | 3.79 | 3.13 | 2.69 | 2.26 | 2.76 | 2.99 | 4.65 |

| | | | | | | | | | | | | | |
|---|---|---|---|---|---|---|---|---|---|---|---|---|---|
| 2.75 | 3.68 | 3.29 | 3.27 | 3.19 | 3.53 | 3.19 | 3.58 | 2.99 | 2.68 | 2.23 | 2.65 | 2.86 | 5.08 |
| 3 | 3.41 | 3.21 | 3.18 | 3.09 | 3.37 | 3.09 | 3.28 | 2.95 | 2.62 | 2.07 | 2.56 | 2.77 | 4.99 |
| 3.25 | 3.17 | 3.09 | 3.09 | 2.97 | 3.17 | 2.95 | 3.06 | 2.76 | 2.5 | 2 | 2.54 | 2.66 | 4.48 |
| 3.5 | 2.74 | 2.89 | 2.89 | 2.78 | 2.92 | 2.79 | 2.73 | 2.58 | 2.49 | 1.91 | 2.39 | 2.59 | 3.84 |
| 3.75 | 2.49 | 2.79 | 2.58 | 2.65 | 2.6 | 2.64 | 2.53 | 2.44 | 2.37 | 1.82 | 2.33 | 2.53 | 3.22 |
| 4 | 2.3 | 2.63 | 2.47 | 2.51 | 2.4 | 2.53 | 2.35 | 2.33 | 2.28 | 1.78 | 2.17 | 2.45 | 2.83 |
| 4.25 | 2.1 | 2.36 | 2.28 | 2.4 | 2.18 | 2.31 | 2.21 | 2.19 | 2.17 | 1.74 | 2.16 | 2.36 | 2.34 |
| 4.5 | 2.01 | 2.26 | 2.25 | 2.26 | 2.02 | 2.16 | 2.08 | 2.11 | 2.09 | 1.67 | 2.04 | 2.2 | 2.01 |
| 4.75 | 1.85 | 2.04 | 2.03 | 2.13 | 1.91 | 2.06 | 1.96 | 2 | 1.97 | 1.6 | 1.96 | 2.17 | 1.79 |
| 5 | 1.76 | 1.93 | 1.95 | 1.98 | 1.81 | 1.93 | 1.84 | 1.85 | 1.85 | 1.51 | 1.85 | 2.08 | 1.65 |
| 5.25 | 1.69 | 1.82 | 1.84 | 1.86 | 1.66 | 1.84 | 1.7 | 1.78 | 1.77 | 1.43 | 1.84 | 2 | 1.5 |
| 5.5 | 1.62 | 1.74 | 1.74 | 1.84 | 1.64 | 1.7 | 1.62 | 1.7 | 1.7 | 1.41 | 1.77 | 2 | 1.47 |
| 5.75 | 1.56 | 1.72 | 1.77 | 1.76 | 1.54 | 1.65 | 1.49 | 1.65 | 1.68 | 1.42 | 1.7 | 1.96 | 1.37 |
| 6 | 1.51 | 1.66 | 1.6 | 1.65 | 1.51 | 1.57 | 1.46 | 1.58 | 1.56 | 1.3 | 1.67 | 1.86 | 1.34 |
| 6.25 | 1.52 | 1.64 | 1.57 | 1.65 | 1.46 | 1.54 | 1.36 | 1.54 | 1.53 | 1.33 | 1.59 | 1.81 | 1.28 |
| 6.5 | 1.49 | 1.64 | 1.6 | 1.63 | 1.43 | 1.52 | 1.28 | 1.5 | 1.54 | 1.34 | 1.58 | 1.77 | 1.27 |
| 6.75 | 1.46 | 1.62 | 1.52 | 1.6 | 1.42 | 1.44 | 1.24 | 1.44 | 1.59 | 1.32 | 1.53 | 1.74 | 1.2 |
| 7 | 1.45 | 1.6 | 1.48 | 1.53 | 1.38 | 1.35 | 1.19 | 1.43 | 1.62 | 1.31 | 1.54 | 1.76 | 1.17 |
| 7.25 | 1.35 | 1.53 | 1.41 | 1.46 | 1.29 | 1.3 | 1.13 | 1.36 | 1.57 | 1.28 | 1.48 | 1.61 | 1.06 |
| 7.5 | 1.4 | 1.51 | 1.37 | 1.39 | 1.22 | 1.22 | 1.03 | 1.32 | 1.54 | 1.23 | 1.43 | 1.53 | 0.98 |
| 7.75 | 1.37 | 1.44 | 1.34 | 1.36 | 1.21 | 1.16 | 1 | 1.26 | 1.54 | 1.19 | 1.35 | 1.5 | 0.91 |
| 8 | 1.34 | 1.42 | 1.24 | 1.25 | 1.12 | 1.06 | 0.94 | 1.22 | 1.44 | 1.16 | 1.3 | 1.39 | 0.89 |
| 8.25 | 1.22 | 1.34 | 1.19 | 1.22 | 1.08 | 1.02 | 0.89 | 1.13 | 1.31 | 1.09 | 1.24 | 1.32 | 0.82 |
| 8.5 | 1.19 | 1.29 | 1.12 | 1.12 | 1.02 | 0.94 | 0.83 | 1.06 | 1.21 | 1.01 | 1.18 | 1.25 | 0.76 |
| 8.75 | 1.13 | 1.22 | 1.07 | 1.06 | 0.96 | 0.86 | 0.78 | 1 | 1.19 | 0.98 | 1.1 | 1.16 | 0.72 |
| 9 | 1.07 | 1.15 | 1.02 | 1.01 | 0.9 | 0.82 | 0.75 | 0.93 | 1.13 | 0.9 | 1.05 | 1.08 | 0.68 |
| 9.25 | 0.99 | 1.08 | 0.95 | 0.94 | 0.84 | 0.78 | 0.7 | 0.88 | 1.04 | 0.85 | 1.02 | 1 | 0.63 |
| 9.5 | 0.95 | 1.04 | 0.87 | 0.89 | 0.81 | 0.73 | 0.69 | 0.84 | 0.98 | 0.81 | 0.96 | 0.98 | 0.59 |
| 9.75 | 0.89 | 0.97 | 0.85 | 0.83 | 0.74 | 0.7 | 0.63 | 0.8 | 0.92 | 0.78 | 0.91 | 0.86 | 0.57 |
| 10 | 0.83 | 0.91 | 0.8 | 0.76 | 0.69 | 0.67 | 0.61 | 0.76 | 0.87 | 0.73 | 0.87 | 0.81 | 0.54 |
| 10.25 | 0.77 | 0.89 | 0.78 | 0.72 | 0.66 | 0.64 | 0.59 | 0.71 | 0.79 | 0.7 | 0.82 | 0.77 | 0.5 |
| 10.5 | 0.72 | 0.81 | 0.73 | 0.7 | 0.63 | 0.61 | 0.56 | 0.7 | 0.77 | 0.65 | 0.78 | 0.7 | 0.48 |
| 10.75 | 0.66 | 0.79 | 0.68 | 0.65 | 0.6 | 0.57 | 0.54 | 0.65 | 0.73 | 0.62 | 0.76 | 0.67 | 0.45 |
| 11 | 0.63 | 0.71 | 0.64 | 0.61 | 0.57 | 0.54 | 0.51 | 0.61 | 0.66 | 0.59 | 0.7 | 0.62 | 0.44 |
| 11.25 | 0.58 | 0.66 | 0.61 | 0.57 | 0.52 | 0.51 | 0.49 | 0.58 | 0.66 | 0.58 | 0.69 | 0.58 | 0.4 |
| 11.5 | 0.55 | 0.6 | 0.59 | 0.55 | 0.5 | 0.47 | 0.46 | 0.57 | 0.62 | 0.54 | 0.65 | 0.54 | 0.38 |
| 11.75 | 0.52 | 0.58 | 0.55 | 0.51 | 0.48 | 0.46 | 0.47 | 0.53 | 0.58 | 0.51 | 0.61 | 0.51 | 0.36 |
| 12 | 0.49 | 0.54 | 0.5 | 0.47 | 0.44 | 0.45 | 0.43 | 0.5 | 0.54 | 0.48 | 0.59 | 0.48 | 0.36 |
| 12.25 | 0.47 | 0.5 | 0.49 | 0.47 | 0.42 | 0.41 | 0.42 | 0.48 | 0.52 | 0.45 | 0.53 | 0.44 | 0.34 |
| 12.5 | 0.44 | 0.46 | 0.46 | 0.42 | 0.39 | 0.39 | 0.4 | 0.46 | 0.5 | 0.43 | 0.54 | 0.42 | 0.32 |
| 12.75 | 0.42 | 0.43 | 0.44 | 0.4 | 0.37 | 0.36 | 0.39 | 0.43 | 0.46 | 0.42 | 0.5 | 0.4 | 0.31 |
| 13 | 0.4 | 0.4 | 0.4 | 0.38 | 0.35 | 0.35 | 0.37 | 0.4 | 0.45 | 0.4 | 0.49 | 0.37 | 0.29 |
| 13.25 | 0.37 | 0.38 | 0.39 | 0.35 | 0.33 | 0.33 | 0.36 | 0.39 | 0.42 | 0.37 | 0.46 | 0.35 | 0.29 |
| 13.5 | 0.35 | 0.34 | 0.36 | 0.34 | 0.32 | 0.31 | 0.35 | 0.37 | 0.4 | 0.36 | 0.44 | 0.34 | 0.27 |
| 13.75 | 0.34 | 0.32 | 0.35 | 0.32 | 0.29 | 0.3 | 0.32 | 0.35 | 0.37 | 0.34 | 0.43 | 0.32 | 0.26 |
| 14 | 0.33 | 0.3 | 0.34 | 0.3 | 0.28 | 0.28 | 0.31 | 0.34 | 0.36 | 0.33 | 0.4 | 0.31 | 0.25 |
| 14.25 | 0.29 | 0.29 | 0.32 | 0.29 | 0.27 | 0.27 | 0.29 | 0.32 | 0.33 | 0.32 | 0.38 | 0.28 | 0.24 |
| 14.5 | 0.29 | 0.27 | 0.3 | 0.27 | 0.26 | 0.25 | 0.27 | 0.3 | 0.33 | 0.3 | 0.37 | 0.27 | 0.23 |

| | | | | | | | | | | | | | |
|---|---|---|---|---|---|---|---|---|---|---|---|---|---|
| 14.75 | 0.27 | 0.25 | 0.29 | 0.25 | 0.24 | 0.24 | 0.26 | 0.28 | 0.3 | 0.28 | 0.34 | 0.26 | 0.22 |
| 15 | 0.26 | 0.24 | 0.27 | 0.25 | 0.24 | 0.22 | 0.25 | 0.27 | 0.29 | 0.27 | 0.32 | 0.24 | 0.21 |
| 15.25 | 0.24 | 0.22 | 0.26 | 0.22 | 0.22 | 0.22 | 0.23 | 0.26 | 0.26 | 0.26 | 0.31 | 0.24 | 0.21 |
| 15.5 | 0.23 | 0.21 | 0.24 | 0.22 | 0.21 | 0.2 | 0.22 | 0.24 | 0.26 | 0.25 | 0.28 | 0.22 | 0.19 |
| 15.75 | 0.22 | 0.19 | 0.23 | 0.2 | 0.2 | 0.19 | 0.21 | 0.22 | 0.24 | 0.23 | 0.28 | 0.21 | 0.18 |
| 16 | 0.2 | 0.19 | 0.22 | 0.2 | 0.19 | 0.19 | 0.19 | 0.22 | 0.22 | 0.22 | 0.26 | 0.2 | 0.18 |
| 16.25 | 0.2 | 0.17 | 0.2 | 0.18 | 0.18 | 0.18 | 0.18 | 0.2 | 0.21 | 0.21 | 0.25 | 0.19 | 0.17 |
| 16.5 | 0.19 | 0.17 | 0.19 | 0.17 | 0.17 | 0.17 | 0.17 | 0.2 | 0.2 | 0.2 | 0.24 | 0.19 | 0.16 |
| 16.75 | 0.18 | 0.15 | 0.18 | 0.17 | 0.16 | 0.16 | 0.15 | 0.18 | 0.18 | 0.19 | 0.23 | 0.18 | 0.15 |
| 17 | 0.17 | 0.14 | 0.17 | 0.16 | 0.15 | 0.15 | 0.15 | 0.17 | 0.18 | 0.18 | 0.21 | 0.16 | 0.15 |
| 17.25 | 0.16 | 0.15 | 0.16 | 0.15 | 0.14 | 0.14 | 0.14 | 0.16 | 0.17 | 0.17 | 0.2 | 0.16 | 0.14 |
| 17.5 | 0.15 | 0.13 | 0.16 | 0.14 | 0.14 | 0.13 | 0.14 | 0.15 | 0.16 | 0.16 | 0.19 | 0.16 | 0.13 |
| 17.75 | 0.15 | 0.13 | 0.15 | 0.14 | 0.13 | 0.13 | 0.13 | 0.14 | 0.16 | 0.15 | 0.18 | 0.15 | 0.13 |
| 18 | 0.14 | 0.12 | 0.14 | 0.13 | 0.12 | 0.12 | 0.13 | 0.14 | 0.15 | 0.14 | 0.17 | 0.14 | 0.13 |
| 18.25 | 0.14 | 0.11 | 0.13 | 0.13 | 0.12 | 0.12 | 0.12 | 0.13 | 0.14 | 0.14 | 0.16 | 0.14 | 0.12 |
| 18.5 | 0.13 | 0.11 | 0.13 | 0.12 | 0.11 | 0.11 | 0.11 | 0.12 | 0.13 | 0.13 | 0.15 | 0.13 | 0.12 |
| 18.75 | 0.12 | 0.11 | 0.12 | 0.12 | 0.1 | 0.1 | 0.11 | 0.12 | 0.13 | 0.13 | 0.15 | 0.13 | 0.11 |
| 19 | 0.12 | 0.1 | 0.12 | 0.11 | 0.1 | 0.1 | 0.11 | 0.11 | 0.12 | 0.13 | 0.14 | 0.12 | 0.11 |
| 19.25 | 0.12 | 0.1 | 0.11 | 0.1 | 0.1 | 0.1 | 0.1 | 0.1 | 0.11 | 0.12 | 0.13 | 0.12 | 0.1 |
| 19.5 | 0.11 | 0.09 | 0.11 | 0.1 | 0.09 | 0.09 | 0.09 | 0.1 | 0.11 | 0.11 | 0.13 | 0.11 | 0.1 |
| 19.75 | 0.11 | 0.09 | 0.1 | 0.1 | 0.09 | 0.09 | 0.09 | 0.1 | 0.11 | 0.11 | 0.13 | 0.11 | 0.09 |
| 20 | 0.11 | 0.09 | 0.1 | 0.09 | 0.09 | 0.09 | 0.09 | 0.09 | 0.1 | 0.11 | 0.12 | 0.1 | 0.09 |

| **TRM** | | | | | | | | | | | | | | | |
|---|---|---|---|---|---|---|---|---|---|---|---|---|---|---|---|
| Hz | | | | | | | | | | | | | | | |
| 0.25 | 2.69 | 1.64 | 1.6 | 1.44 | 3.33 | 1.76 | 1.48 | 2.32 | 4.67 | 1.35 | 1.59 | 1.19 | 1.3 | 2.3 | 1.61 |
| 0.5 | 2.72 | 1.9 | 2.36 | 2.68 | 3.07 | 2.79 | 2.18 | 2.63 | 4.02 | 1.92 | 2.36 | 2.03 | 2.21 | 2.63 | 2.91 |
| 0.75 | 3.44 | 2.47 | 2.99 | 3.75 | 3.13 | 3.4 | 3.02 | 3.21 | 3.91 | 2.46 | 2.74 | 2.79 | 2.74 | 3.62 | 3.46 |
| 1 | 3.78 | 2.67 | 3.46 | 4.03 | 3.13 | 3.64 | 3.58 | 3.41 | 4.03 | 2.81 | 2.88 | 3.07 | 2.96 | 4.01 | 3.48 |
| 1.25 | 3.82 | 2.84 | 3.66 | 4.25 | 3 | 3.76 | 3.78 | 3.47 | 3.95 | 2.92 | 2.79 | 3.21 | 2.99 | 4.16 | 3.47 |
| 1.5 | 3.62 | 2.85 | 3.69 | 4.39 | 2.99 | 3.73 | 3.83 | 3.46 | 3.78 | 2.94 | 2.8 | 3.21 | 3.11 | 4.17 | 3.4 |
| 1.75 | 3.39 | 2.87 | 3.84 | 4.36 | 2.86 | 3.61 | 3.77 | 3.48 | 3.56 | 3.02 | 2.76 | 3.13 | 2.95 | 4.09 | 3.37 |
| 2 | 3.34 | 2.9 | 3.83 | 4.32 | 2.82 | 3.47 | 3.67 | 3.39 | 3.35 | 3.14 | 2.88 | 3.15 | 3 | 3.97 | 3.37 |
| 2.25 | 3.27 | 2.95 | 4.01 | 4.18 | 2.89 | 3.4 | 3.44 | 3.5 | 3.13 | 3.18 | 3 | 3.18 | 2.96 | 3.78 | 3.39 |
| 2.5 | 3.16 | 2.92 | 3.99 | 4.12 | 2.81 | 3.28 | 3.26 | 3.4 | 3.06 | 3.28 | 3.22 | 3.11 | 2.92 | 3.64 | 3.45 |
| 2.75 | 2.9 | 2.99 | 3.86 | 4.07 | 2.75 | 3.35 | 3.16 | 3.22 | 3.08 | 3.28 | 3.32 | 3.1 | 2.85 | 3.41 | 3.33 |
| 3 | 2.92 | 2.86 | 3.78 | 3.93 | 2.82 | 3.33 | 2.96 | 3.27 | 2.92 | 3.3 | 3.33 | 3.03 | 2.83 | 3.17 | 3.07 |
| 3.25 | 2.8 | 2.83 | 3.5 | 3.67 | 2.76 | 3.32 | 2.85 | 3.08 | 2.84 | 3.17 | 3.06 | 3.06 | 2.87 | 2.97 | 2.82 |
| 3.5 | 2.58 | 2.82 | 3.3 | 3.29 | 2.68 | 3.08 | 2.77 | 2.87 | 2.68 | 3.09 | 2.67 | 2.82 | 2.78 | 2.65 | 2.61 |
| 3.75 | 2.52 | 2.7 | 2.98 | 2.91 | 2.52 | 2.83 | 2.68 | 2.48 | 2.46 | 2.89 | 2.52 | 2.7 | 2.72 | 2.44 | 2.5 |
| 4 | 2.44 | 2.6 | 2.63 | 2.56 | 2.39 | 2.51 | 2.51 | 2.19 | 2.28 | 2.74 | 2.31 | 2.55 | 2.6 | 2.22 | 2.43 |
| 4.25 | 2.29 | 2.45 | 2.37 | 2.36 | 2.2 | 2.29 | 2.38 | 2 | 2.17 | 2.54 | 2.17 | 2.43 | 2.41 | 2.13 | 2.32 |
| 4.5 | 2.26 | 2.42 | 2.2 | 2.1 | 2.1 | 2.12 | 2.28 | 1.88 | 2.11 | 2.49 | 2.01 | 2.26 | 2.29 | 1.97 | 2.26 |
| 4.75 | 2.04 | 2.33 | 2.04 | 1.91 | 1.94 | 1.99 | 2.17 | 1.76 | 1.97 | 2.3 | 1.93 | 2.11 | 2.17 | 1.82 | 2.16 |

| | | | | | | | | | | | | | | | |
|---|---|---|---|---|---|---|---|---|---|---|---|---|---|---|---|
| 5 | 2.03 | 2.2 | 1.95 | 1.78 | 1.9 | 1.82 | 2.12 | 1.66 | 1.95 | 2.2 | 1.87 | 2.05 | 2.16 | 1.7 | 2.15 |
| 5.25 | 1.95 | 2.1 | 1.87 | 1.67 | 1.83 | 1.72 | 1.95 | 1.65 | 1.85 | 2.02 | 1.78 | 2.06 | 1.99 | 1.69 | 2.11 |
| 5.5 | 1.9 | 2.05 | 1.82 | 1.56 | 1.78 | 1.67 | 1.95 | 1.61 | 1.72 | 1.96 | 1.75 | 1.93 | 1.98 | 1.64 | 2.15 |
| 5.75 | 1.84 | 1.95 | 1.74 | 1.54 | 1.71 | 1.58 | 1.87 | 1.58 | 1.65 | 1.91 | 1.71 | 1.92 | 1.98 | 1.59 | 2.08 |
| 6 | 1.78 | 1.97 | 1.74 | 1.5 | 1.69 | 1.51 | 1.86 | 1.57 | 1.64 | 1.79 | 1.65 | 1.92 | 1.9 | 1.63 | 2.02 |
| 6.25 | 1.75 | 1.96 | 1.67 | 1.5 | 1.6 | 1.5 | 1.76 | 1.5 | 1.61 | 1.81 | 1.54 | 1.88 | 1.9 | 1.63 | 1.88 |
| 6.5 | 1.69 | 1.89 | 1.63 | 1.45 | 1.57 | 1.46 | 1.7 | 1.48 | 1.61 | 1.75 | 1.58 | 1.84 | 1.85 | 1.59 | 1.81 |
| 6.75 | 1.68 | 1.88 | 1.56 | 1.45 | 1.51 | 1.44 | 1.71 | 1.5 | 1.54 | 1.68 | 1.6 | 1.76 | 1.82 | 1.54 | 1.7 |
| 7 | 1.61 | 1.8 | 1.46 | 1.32 | 1.4 | 1.38 | 1.6 | 1.48 | 1.5 | 1.63 | 1.5 | 1.76 | 1.73 | 1.46 | 1.58 |
| 7.25 | 1.55 | 1.71 | 1.42 | 1.26 | 1.34 | 1.33 | 1.51 | 1.43 | 1.41 | 1.56 | 1.4 | 1.66 | 1.67 | 1.36 | 1.51 |
| 7.5 | 1.4 | 1.6 | 1.37 | 1.18 | 1.31 | 1.25 | 1.43 | 1.36 | 1.38 | 1.46 | 1.39 | 1.62 | 1.6 | 1.3 | 1.38 |
| 7.75 | 1.35 | 1.51 | 1.28 | 1.14 | 1.25 | 1.21 | 1.34 | 1.28 | 1.31 | 1.41 | 1.33 | 1.57 | 1.46 | 1.24 | 1.3 |
| 8 | 1.26 | 1.43 | 1.18 | 1.05 | 1.18 | 1.17 | 1.27 | 1.25 | 1.23 | 1.31 | 1.26 | 1.48 | 1.39 | 1.16 | 1.18 |
| 8.25 | 1.16 | 1.33 | 1.1 | 0.96 | 1.11 | 1.09 | 1.18 | 1.17 | 1.11 | 1.24 | 1.21 | 1.39 | 1.31 | 1.08 | 1.08 |
| 8.5 | 1.12 | 1.25 | 1.03 | 0.87 | 1.04 | 1.06 | 1.1 | 1.12 | 1.07 | 1.18 | 1.1 | 1.34 | 1.2 | 1.03 | 1.02 |
| 8.75 | 1 | 1.17 | 0.96 | 0.82 | 1.01 | 0.97 | 1.03 | 1.03 | 1 | 1.14 | 1.06 | 1.27 | 1.16 | 0.94 | 0.97 |
| 9 | 0.94 | 1.1 | 0.92 | 0.75 | 0.92 | 0.92 | 0.98 | 0.99 | 0.93 | 1.06 | 1 | 1.12 | 1.1 | 0.88 | 0.9 |
| 9.25 | 0.88 | 1.05 | 0.84 | 0.71 | 0.87 | 0.89 | 0.89 | 0.94 | 0.89 | 1 | 0.96 | 1.06 | 1 | 0.85 | 0.84 |
| 9.5 | 0.85 | 1 | 0.79 | 0.69 | 0.85 | 0.83 | 0.85 | 0.9 | 0.81 | 0.94 | 0.88 | 1.01 | 0.95 | 0.8 | 0.77 |
| 9.75 | 0.78 | 0.93 | 0.72 | 0.65 | 0.8 | 0.77 | 0.81 | 0.81 | 0.77 | 0.91 | 0.83 | 0.95 | 0.92 | 0.76 | 0.74 |
| 10 | 0.73 | 0.88 | 0.67 | 0.59 | 0.77 | 0.74 | 0.75 | 0.77 | 0.69 | 0.86 | 0.8 | 0.87 | 0.87 | 0.7 | 0.69 |
| 10.25 | 0.7 | 0.82 | 0.62 | 0.55 | 0.69 | 0.71 | 0.74 | 0.73 | 0.67 | 0.8 | 0.76 | 0.81 | 0.83 | 0.67 | 0.66 |
| 10.5 | 0.66 | 0.77 | 0.58 | 0.52 | 0.67 | 0.67 | 0.67 | 0.68 | 0.62 | 0.77 | 0.71 | 0.75 | 0.8 | 0.64 | 0.63 |
| 10.75 | 0.62 | 0.73 | 0.55 | 0.49 | 0.63 | 0.64 | 0.64 | 0.65 | 0.59 | 0.72 | 0.7 | 0.7 | 0.73 | 0.62 | 0.59 |
| 11 | 0.58 | 0.69 | 0.51 | 0.46 | 0.58 | 0.62 | 0.59 | 0.6 | 0.54 | 0.66 | 0.63 | 0.65 | 0.72 | 0.58 | 0.55 |
| 11.25 | 0.55 | 0.64 | 0.47 | 0.43 | 0.55 | 0.58 | 0.56 | 0.55 | 0.5 | 0.63 | 0.62 | 0.6 | 0.68 | 0.53 | 0.52 |
| 11.5 | 0.51 | 0.61 | 0.43 | 0.41 | 0.5 | 0.55 | 0.52 | 0.54 | 0.48 | 0.62 | 0.57 | 0.59 | 0.64 | 0.49 | 0.5 |
| 11.75 | 0.48 | 0.58 | 0.41 | 0.39 | 0.47 | 0.53 | 0.5 | 0.5 | 0.43 | 0.57 | 0.53 | 0.53 | 0.61 | 0.48 | 0.48 |
| 12 | 0.44 | 0.55 | 0.38 | 0.36 | 0.45 | 0.5 | 0.47 | 0.48 | 0.42 | 0.52 | 0.5 | 0.51 | 0.56 | 0.46 | 0.43 |
| 12.25 | 0.44 | 0.54 | 0.37 | 0.34 | 0.41 | 0.48 | 0.42 | 0.46 | 0.39 | 0.5 | 0.48 | 0.46 | 0.53 | 0.42 | 0.41 |
| 12.5 | 0.41 | 0.5 | 0.34 | 0.32 | 0.39 | 0.46 | 0.41 | 0.43 | 0.37 | 0.48 | 0.46 | 0.43 | 0.5 | 0.4 | 0.4 |
| 12.75 | 0.4 | 0.49 | 0.32 | 0.31 | 0.37 | 0.42 | 0.4 | 0.42 | 0.33 | 0.44 | 0.42 | 0.41 | 0.48 | 0.39 | 0.37 |
| 13 | 0.37 | 0.46 | 0.3 | 0.29 | 0.35 | 0.41 | 0.38 | 0.39 | 0.33 | 0.43 | 0.39 | 0.38 | 0.46 | 0.36 | 0.35 |
| 13.25 | 0.36 | 0.43 | 0.28 | 0.27 | 0.33 | 0.4 | 0.36 | 0.36 | 0.3 | 0.41 | 0.38 | 0.35 | 0.42 | 0.34 | 0.33 |
| 13.5 | 0.34 | 0.42 | 0.27 | 0.27 | 0.32 | 0.37 | 0.34 | 0.35 | 0.28 | 0.39 | 0.37 | 0.34 | 0.39 | 0.33 | 0.32 |
| 13.75 | 0.33 | 0.39 | 0.26 | 0.25 | 0.3 | 0.35 | 0.33 | 0.34 | 0.27 | 0.38 | 0.34 | 0.32 | 0.37 | 0.31 | 0.3 |
| 14 | 0.32 | 0.37 | 0.24 | 0.24 | 0.28 | 0.34 | 0.3 | 0.33 | 0.26 | 0.34 | 0.34 | 0.3 | 0.36 | 0.3 | 0.28 |
| 14.25 | 0.3 | 0.36 | 0.24 | 0.22 | 0.27 | 0.32 | 0.29 | 0.31 | 0.24 | 0.34 | 0.31 | 0.29 | 0.34 | 0.28 | 0.27 |
| 14.5 | 0.29 | 0.34 | 0.22 | 0.21 | 0.25 | 0.3 | 0.27 | 0.29 | 0.22 | 0.32 | 0.3 | 0.28 | 0.31 | 0.27 | 0.26 |
| 14.75 | 0.26 | 0.32 | 0.21 | 0.2 | 0.24 | 0.29 | 0.26 | 0.28 | 0.21 | 0.3 | 0.29 | 0.25 | 0.31 | 0.25 | 0.25 |
| 15 | 0.25 | 0.31 | 0.2 | 0.19 | 0.23 | 0.27 | 0.25 | 0.26 | 0.2 | 0.28 | 0.27 | 0.25 | 0.28 | 0.24 | 0.23 |
| 15.25 | 0.24 | 0.29 | 0.18 | 0.18 | 0.22 | 0.26 | 0.23 | 0.25 | 0.19 | 0.27 | 0.26 | 0.23 | 0.27 | 0.23 | 0.22 |
| 15.5 | 0.23 | 0.28 | 0.18 | 0.17 | 0.21 | 0.25 | 0.22 | 0.24 | 0.18 | 0.24 | 0.24 | 0.21 | 0.26 | 0.22 | 0.21 |
| 15.75 | 0.22 | 0.26 | 0.17 | 0.16 | 0.2 | 0.24 | 0.21 | 0.22 | 0.17 | 0.24 | 0.24 | 0.2 | 0.24 | 0.21 | 0.21 |
| 16 | 0.21 | 0.25 | 0.16 | 0.15 | 0.19 | 0.23 | 0.19 | 0.21 | 0.16 | 0.22 | 0.23 | 0.2 | 0.23 | 0.2 | 0.2 |
| 16.25 | 0.2 | 0.24 | 0.15 | 0.14 | 0.18 | 0.21 | 0.19 | 0.21 | 0.15 | 0.21 | 0.22 | 0.19 | 0.22 | 0.19 | 0.19 |
| 16.5 | 0.19 | 0.22 | 0.15 | 0.13 | 0.17 | 0.2 | 0.18 | 0.2 | 0.14 | 0.21 | 0.21 | 0.18 | 0.21 | 0.17 | 0.18 |
| 16.75 | 0.18 | 0.22 | 0.14 | 0.13 | 0.16 | 0.19 | 0.17 | 0.19 | 0.14 | 0.2 | 0.19 | 0.17 | 0.2 | 0.17 | 0.17 |

| | | | | | | | | | | | | | | | |
|---|---|---|---|---|---|---|---|---|---|---|---|---|---|---|---|
| 17 | 0.17 | 0.2 | 0.13 | 0.12 | 0.15 | 0.19 | 0.16 | 0.18 | 0.13 | 0.18 | 0.19 | 0.16 | 0.19 | 0.16 | 0.16 |
| 17.25 | 0.16 | 0.19 | 0.13 | 0.12 | 0.14 | 0.18 | 0.15 | 0.17 | 0.13 | 0.17 | 0.18 | 0.16 | 0.18 | 0.15 | 0.16 |
| 17.5 | 0.16 | 0.18 | 0.12 | 0.11 | 0.14 | 0.16 | 0.15 | 0.16 | 0.12 | 0.16 | 0.16 | 0.15 | 0.17 | 0.15 | 0.15 |
| 17.75 | 0.15 | 0.17 | 0.11 | 0.11 | 0.13 | 0.16 | 0.14 | 0.16 | 0.12 | 0.16 | 0.16 | 0.15 | 0.16 | 0.13 | 0.15 |
| 18 | 0.14 | 0.16 | 0.11 | 0.1 | 0.13 | 0.15 | 0.13 | 0.15 | 0.1 | 0.15 | 0.16 | 0.14 | 0.15 | 0.13 | 0.14 |
| 18.25 | 0.14 | 0.16 | 0.11 | 0.1 | 0.12 | 0.15 | 0.13 | 0.14 | 0.1 | 0.14 | 0.15 | 0.14 | 0.15 | 0.13 | 0.14 |
| 18.5 | 0.13 | 0.15 | 0.1 | 0.09 | 0.12 | 0.14 | 0.12 | 0.14 | 0.1 | 0.14 | 0.14 | 0.13 | 0.14 | 0.12 | 0.13 |
| 18.75 | 0.12 | 0.14 | 0.1 | 0.09 | 0.11 | 0.13 | 0.12 | 0.13 | 0.09 | 0.13 | 0.14 | 0.12 | 0.14 | 0.12 | 0.12 |
| 19 | 0.12 | 0.13 | 0.09 | 0.08 | 0.11 | 0.13 | 0.11 | 0.13 | 0.09 | 0.12 | 0.14 | 0.12 | 0.13 | 0.11 | 0.12 |
| 19.25 | 0.11 | 0.13 | 0.09 | 0.08 | 0.1 | 0.12 | 0.11 | 0.13 | 0.09 | 0.12 | 0.13 | 0.11 | 0.13 | 0.11 | 0.12 |
| 19.5 | 0.11 | 0.12 | 0.09 | 0.07 | 0.1 | 0.12 | 0.1 | 0.12 | 0.08 | 0.11 | 0.13 | 0.1 | 0.12 | 0.1 | 0.11 |
| 19.75 | 0.11 | 0.12 | 0.08 | 0.08 | 0.1 | 0.11 | 0.1 | 0.12 | 0.08 | 0.11 | 0.13 | 0.1 | 0.12 | 0.1 | 0.11 |
| 20 | 0.1 | 0.11 | 0.08 | 0.07 | 0.1 | 0.11 | 0.09 | 0.11 | 0.07 | 0.1 | 0.12 | 0.1 | 0.11 | 0.09 | 0.11 |

Electroencephalographic power spectral density during REMS

| **CLF** | | | | | | | | | | | | |
|---|---|---|---|---|---|---|---|---|---|---|---|---|
| Hz | | | | | | | | | | | | |
| 0.25 | 4.32 | 1.49 | 3.53 | 1.46 | 1.34 | 2.65 | 1.35 | 4.9 | 2.58 | 4.04 | 1.32 | 1.05 |
| 0.5 | 2.48 | 2.06 | 1.8 | 1.93 | 1.47 | 2.43 | 1.56 | 2.01 | 2.46 | 2.5 | 1.57 | 1.18 |
| 0.75 | 1.89 | 1.98 | 1.43 | 1.77 | 1.45 | 2.12 | 1.59 | 1.59 | 1.95 | 1.69 | 1.55 | 1.22 |
| 1 | 1.66 | 1.76 | 1.54 | 1.66 | 1.47 | 1.8 | 1.69 | 1.66 | 1.82 | 1.57 | 1.52 | 1.31 |
| 1.25 | 1.62 | 1.52 | 1.54 | 1.61 | 1.44 | 1.84 | 1.44 | 1.5 | 1.63 | 1.7 | 1.43 | 1.29 |
| 1.5 | 1.56 | 1.36 | 1.73 | 1.54 | 1.39 | 1.81 | 1.5 | 1.57 | 1.65 | 1.55 | 1.35 | 1.27 |
| 1.75 | 1.5 | 1.4 | 1.51 | 1.66 | 1.36 | 1.8 | 1.32 | 1.52 | 1.57 | 1.6 | 1.34 | 1.32 |
| 2 | 1.4 | 1.35 | 1.63 | 1.55 | 1.34 | 1.76 | 1.48 | 1.55 | 1.56 | 1.56 | 1.3 | 1.4 |
| 2.25 | 1.47 | 1.27 | 1.46 | 1.65 | 1.2 | 1.76 | 1.6 | 1.62 | 1.62 | 1.63 | 1.27 | 1.44 |
| 2.5 | 1.45 | 1.37 | 1.51 | 1.54 | 1.27 | 1.67 | 1.63 | 1.5 | 1.62 | 1.61 | 1.38 | 1.56 |
| 2.75 | 1.39 | 1.31 | 1.64 | 1.59 | 1.29 | 1.81 | 1.18 | 1.45 | 1.72 | 1.56 | 1.34 | 1.55 |
| 3 | 1.37 | 1.58 | 1.48 | 1.48 | 1.36 | 1.73 | 1.39 | 1.44 | 1.34 | 1.52 | 1.34 | 1.37 |
| 3.25 | 1.29 | 1.61 | 1.63 | 1.54 | 1.39 | 1.61 | 1.32 | 1.38 | 1.42 | 1.45 | 1.19 | 1.4 |
| 3.5 | 1.19 | 1.56 | 1.48 | 1.57 | 1.19 | 1.58 | 1.22 | 1.29 | 1.26 | 1.24 | 1.15 | 1.32 |
| 3.75 | 1.2 | 1.6 | 1.43 | 1.45 | 1.21 | 1.33 | 1.29 | 1.38 | 1.24 | 1.24 | 1.09 | 1.34 |
| 4 | 1.18 | 1.41 | 1.24 | 1.44 | 1.18 | 1.27 | 1.3 | 1.35 | 1.18 | 1.42 | 1.05 | 1.3 |
| 4.25 | 1.18 | 1.22 | 1.29 | 1.36 | 1.09 | 1.35 | 1.56 | 1.29 | 1.13 | 1.33 | 0.98 | 1.38 |
| 4.5 | 1.16 | 1.15 | 1.37 | 1.35 | 1.04 | 1.29 | 1.8 | 1.4 | 1.19 | 1.18 | 1 | 1.34 |
| 4.75 | 1.15 | 1.2 | 1.47 | 1.47 | 1.09 | 1.3 | 2.67 | 1.43 | 1.23 | 1.18 | 0.96 | 1.4 |
| 5 | 1.2 | 1.16 | 1.31 | 1.47 | 1.09 | 1.21 | 3.19 | 1.4 | 1.19 | 1.02 | 0.99 | 1.45 |
| 5.25 | 1.21 | 1.16 | 1.43 | 1.64 | 1.19 | 1.22 | 3.56 | 1.45 | 1.17 | 1 | 1.04 | 1.42 |
| 5.5 | 1.49 | 1.33 | 1.37 | 1.72 | 1.21 | 1.47 | 4.41 | 1.64 | 1.26 | 1.15 | 1.1 | 1.58 |
| 5.75 | 1.67 | 1.5 | 1.58 | 1.77 | 1.35 | 1.51 | 4 | 1.81 | 1.46 | 1.08 | 1.24 | 1.76 |
| 6 | 2.15 | 1.82 | 1.9 | 2.23 | 1.61 | 2.04 | 5.96 | 2.28 | 1.78 | 1.31 | 1.58 | 2.19 |
| 6.25 | 2.64 | 2.23 | 2.26 | 2.62 | 1.99 | 2.31 | 6 | 2.68 | 2.38 | 1.72 | 1.9 | 2.57 |
| 6.5 | 3.61 | 2.77 | 2.57 | 3.02 | 2.43 | 3.06 | 4.44 | 3.24 | 2.97 | 2.13 | 2.37 | 3.06 |
| 6.75 | 4.22 | 3.32 | 3.07 | 3.8 | 2.95 | 3.64 | 3.84 | 3.54 | 3.11 | 2.36 | 3.13 | 3.66 |
| 7 | 4.36 | 3.87 | 3.55 | 3.94 | 3.91 | 3.84 | 4 | 3.85 | 3.67 | 2.95 | 4 | 3.93 |
| 7.25 | 4.65 | 4.7 | 3.74 | 4.06 | 4.51 | 3.85 | 3.62 | 3.7 | 4.44 | 3.6 | 4.89 | 4.36 |

| | | | | | | | | | | | | |
|---|---|---|---|---|---|---|---|---|---|---|---|---|
| 7.5 | 4.53 | 5.08 | 3.94 | 4.15 | 4.8 | 4.23 | 2.76 | 3.79 | 3.86 | 3.99 | 5.34 | 4.25 |
| 7.75 | 4.04 | 5.09 | 3.69 | 3.71 | 4.99 | 3.86 | 2.78 | 3.35 | 3.87 | 4.03 | 5.55 | 4.31 |
| 8 | 3.53 | 4.37 | 3.24 | 3.59 | 4.86 | 3.41 | 1.77 | 3.08 | 3.19 | 3.25 | 5 | 4 |
| 8.25 | 2.79 | 3.71 | 2.65 | 2.84 | 4.17 | 2.89 | 1.91 | 2.77 | 2.58 | 2.82 | 4.31 | 3.57 |
| 8.5 | 2.44 | 3.09 | 2.06 | 2.33 | 3.75 | 2.41 | 1.45 | 2.4 | 2.38 | 2.24 | 3.79 | 2.92 |
| 8.75 | 1.93 | 2.44 | 1.81 | 2.03 | 2.96 | 1.92 | 1.03 | 2.2 | 1.74 | 1.9 | 3.16 | 2.53 |
| 9 | 1.56 | 1.98 | 1.48 | 1.78 | 2.82 | 1.55 | 1 | 1.99 | 1.39 | 1.7 | 2.45 | 2.14 |
| 9.25 | 1.34 | 1.81 | 1.28 | 1.47 | 2.34 | 1.19 | 0.81 | 1.74 | 1.21 | 1.32 | 1.99 | 1.84 |
| 9.5 | 1.15 | 1.37 | 1.18 | 1.2 | 2.01 | 1.1 | 0.73 | 1.35 | 1.06 | 1.04 | 1.63 | 1.46 |
| 9.75 | 1.04 | 1.09 | 0.95 | 1.06 | 1.72 | 1 | 0.62 | 1.26 | 0.87 | 0.81 | 1.44 | 1.24 |
| 10 | 0.93 | 0.96 | 0.9 | 0.94 | 1.37 | 0.9 | 0.55 | 1.06 | 0.8 | 0.76 | 1.21 | 1.09 |
| 10.25 | 0.76 | 0.86 | 0.84 | 0.82 | 1.17 | 0.8 | 0.45 | 0.96 | 0.78 | 0.66 | 1.08 | 0.98 |
| 10.5 | 0.69 | 0.74 | 0.75 | 0.79 | 0.99 | 0.67 | 0.45 | 0.87 | 0.64 | 0.64 | 0.95 | 0.89 |
| 10.75 | 0.6 | 0.68 | 0.72 | 0.72 | 0.87 | 0.58 | 0.41 | 0.8 | 0.58 | 0.55 | 0.8 | 0.82 |
| 11 | 0.55 | 0.58 | 0.6 | 0.64 | 0.85 | 0.58 | 0.36 | 0.67 | 0.51 | 0.54 | 0.71 | 0.78 |
| 11.25 | 0.5 | 0.52 | 0.53 | 0.56 | 0.76 | 0.53 | 0.39 | 0.67 | 0.49 | 0.54 | 0.67 | 0.68 |
| 11.5 | 0.48 | 0.52 | 0.52 | 0.58 | 0.65 | 0.51 | 0.29 | 0.57 | 0.46 | 0.44 | 0.58 | 0.64 |
| 11.75 | 0.44 | 0.49 | 0.48 | 0.5 | 0.61 | 0.48 | 0.37 | 0.52 | 0.42 | 0.41 | 0.53 | 0.56 |
| 12 | 0.42 | 0.41 | 0.46 | 0.47 | 0.51 | 0.43 | 0.33 | 0.5 | 0.4 | 0.41 | 0.5 | 0.54 |
| 12.25 | 0.39 | 0.42 | 0.44 | 0.47 | 0.45 | 0.4 | 0.3 | 0.47 | 0.38 | 0.39 | 0.45 | 0.52 |
| 12.5 | 0.39 | 0.35 | 0.4 | 0.41 | 0.42 | 0.39 | 0.34 | 0.44 | 0.36 | 0.36 | 0.4 | 0.47 |
| 12.75 | 0.36 | 0.36 | 0.41 | 0.41 | 0.39 | 0.38 | 0.29 | 0.42 | 0.35 | 0.37 | 0.37 | 0.43 |
| 13 | 0.34 | 0.32 | 0.4 | 0.39 | 0.35 | 0.35 | 0.27 | 0.4 | 0.34 | 0.36 | 0.37 | 0.41 |
| 13.25 | 0.32 | 0.31 | 0.43 | 0.4 | 0.37 | 0.35 | 0.26 | 0.36 | 0.34 | 0.34 | 0.35 | 0.4 |
| 13.5 | 0.32 | 0.31 | 0.42 | 0.36 | 0.32 | 0.34 | 0.28 | 0.38 | 0.36 | 0.34 | 0.3 | 0.39 |
| 13.75 | 0.31 | 0.31 | 0.43 | 0.35 | 0.29 | 0.35 | 0.26 | 0.35 | 0.34 | 0.36 | 0.31 | 0.36 |
| 14 | 0.32 | 0.33 | 0.39 | 0.35 | 0.3 | 0.32 | 0.25 | 0.33 | 0.3 | 0.35 | 0.28 | 0.35 |
| 14.25 | 0.3 | 0.3 | 0.42 | 0.34 | 0.29 | 0.32 | 0.23 | 0.31 | 0.36 | 0.36 | 0.28 | 0.35 |
| 14.5 | 0.29 | 0.28 | 0.45 | 0.33 | 0.26 | 0.31 | 0.21 | 0.3 | 0.33 | 0.34 | 0.27 | 0.35 |
| 14.75 | 0.3 | 0.28 | 0.47 | 0.31 | 0.25 | 0.3 | 0.2 | 0.31 | 0.32 | 0.33 | 0.27 | 0.3 |
| 15 | 0.27 | 0.29 | 0.48 | 0.33 | 0.25 | 0.3 | 0.2 | 0.29 | 0.31 | 0.33 | 0.25 | 0.3 |
| 15.25 | 0.27 | 0.27 | 0.47 | 0.29 | 0.23 | 0.3 | 0.19 | 0.27 | 0.32 | 0.34 | 0.25 | 0.3 |
| 15.5 | 0.27 | 0.26 | 0.47 | 0.3 | 0.24 | 0.29 | 0.15 | 0.26 | 0.32 | 0.31 | 0.24 | 0.28 |
| 15.75 | 0.25 | 0.25 | 0.48 | 0.28 | 0.21 | 0.25 | 0.19 | 0.24 | 0.28 | 0.28 | 0.23 | 0.27 |
| 16 | 0.23 | 0.24 | 0.41 | 0.26 | 0.22 | 0.27 | 0.14 | 0.25 | 0.26 | 0.27 | 0.23 | 0.26 |
| 16.25 | 0.24 | 0.26 | 0.38 | 0.27 | 0.19 | 0.26 | 0.15 | 0.23 | 0.26 | 0.28 | 0.21 | 0.24 |
| 16.5 | 0.22 | 0.22 | 0.4 | 0.27 | 0.2 | 0.27 | 0.13 | 0.22 | 0.26 | 0.24 | 0.21 | 0.23 |
| 16.75 | 0.21 | 0.23 | 0.39 | 0.24 | 0.18 | 0.25 | 0.11 | 0.2 | 0.24 | 0.24 | 0.2 | 0.21 |
| 17 | 0.19 | 0.2 | 0.37 | 0.23 | 0.18 | 0.24 | 0.13 | 0.21 | 0.24 | 0.23 | 0.2 | 0.21 |
| 17.25 | 0.18 | 0.21 | 0.37 | 0.22 | 0.16 | 0.23 | 0.11 | 0.19 | 0.23 | 0.23 | 0.18 | 0.2 |
| 17.5 | 0.18 | 0.19 | 0.31 | 0.21 | 0.17 | 0.23 | 0.11 | 0.18 | 0.23 | 0.23 | 0.18 | 0.2 |
| 17.75 | 0.18 | 0.17 | 0.34 | 0.2 | 0.16 | 0.22 | 0.11 | 0.17 | 0.21 | 0.22 | 0.17 | 0.18 |
| 18 | 0.17 | 0.17 | 0.3 | 0.19 | 0.15 | 0.21 | 0.11 | 0.17 | 0.2 | 0.21 | 0.16 | 0.18 |
| 18.25 | 0.17 | 0.17 | 0.28 | 0.19 | 0.14 | 0.19 | 0.11 | 0.15 | 0.2 | 0.19 | 0.16 | 0.18 |
| 18.5 | 0.16 | 0.16 | 0.29 | 0.17 | 0.14 | 0.2 | 0.09 | 0.15 | 0.18 | 0.19 | 0.15 | 0.17 |
| 18.75 | 0.15 | 0.15 | 0.25 | 0.17 | 0.14 | 0.18 | 0.11 | 0.14 | 0.17 | 0.17 | 0.14 | 0.16 |
| 19 | 0.16 | 0.14 | 0.24 | 0.16 | 0.12 | 0.18 | 0.11 | 0.15 | 0.18 | 0.17 | 0.14 | 0.15 |
| 19.25 | 0.15 | 0.14 | 0.22 | 0.14 | 0.12 | 0.17 | 0.09 | 0.13 | 0.18 | 0.16 | 0.14 | 0.15 |

| | | | | | | | | | | | | |
|---|---|---|---|---|---|---|---|---|---|---|---|---|
| 19.5 | 0.15 | 0.14 | 0.24 | 0.15 | 0.12 | 0.17 | 0.09 | 0.14 | 0.16 | 0.16 | 0.14 | 0.14 |
| 19.75 | 0.15 | 0.14 | 0.23 | 0.14 | 0.11 | 0.15 | 0.1 | 0.12 | 0.16 | 0.17 | 0.12 | 0.14 |
| 20 | 0.14 | 0.12 | 0.23 | 0.14 | 0.11 | 0.15 | 0.1 | 0.12 | 0.16 | 0.15 | 0.12 | 0.14 |

| **CLM** | | | | | | | | | | | |
|---|---|---|---|---|---|---|---|---|---|---|---|
| Hz | | | | | | | | | | | |
| 0.25 | 2.92 | 3.12 | 1.14 | 0.78 | 1.31 | 1.05 | 2 | 4.92 | 2.15 | 1.01 | 2.79 |
| 0.5 | 2.33 | 2.06 | 1.53 | 1.17 | 1.48 | 1.67 | 1.5 | 2.02 | 2.08 | 1.37 | 1.51 |
| 0.75 | 2.37 | 2.24 | 1.61 | 1.47 | 1.52 | 1.57 | 1.25 | 1.49 | 1.84 | 1.5 | 1.53 |
| 1 | 2.11 | 1.95 | 1.59 | 1.49 | 1.47 | 1.56 | 1.29 | 1.46 | 1.65 | 1.46 | 1.47 |
| 1.25 | 1.6 | 1.63 | 1.54 | 1.58 | 1.45 | 1.53 | 1.14 | 1.38 | 1.47 | 1.54 | 1.44 |
| 1.5 | 1.6 | 1.79 | 1.67 | 1.74 | 1.49 | 1.57 | 1.12 | 1.41 | 1.6 | 1.46 | 1.41 |
| 1.75 | 1.75 | 1.46 | 1.72 | 1.73 | 1.46 | 1.65 | 1.19 | 1.39 | 1.62 | 1.45 | 1.47 |
| 2 | 1.71 | 1.55 | 1.84 | 1.84 | 1.63 | 1.62 | 1.29 | 1.43 | 1.68 | 1.43 | 1.47 |
| 2.25 | 1.78 | 1.5 | 1.92 | 1.73 | 1.59 | 1.92 | 1.28 | 1.45 | 1.61 | 1.43 | 1.42 |
| 2.5 | 1.9 | 1.54 | 1.84 | 1.88 | 1.52 | 1.91 | 1.33 | 1.5 | 1.8 | 1.46 | 1.43 |
| 2.75 | 1.58 | 1.64 | 1.69 | 1.77 | 1.58 | 1.87 | 1.3 | 1.47 | 1.93 | 1.5 | 1.42 |
| 3 | 1.64 | 1.77 | 1.59 | 1.64 | 1.54 | 1.67 | 1.2 | 1.35 | 1.9 | 1.6 | 1.25 |
| 3.25 | 1.49 | 1.67 | 1.58 | 1.61 | 1.36 | 1.61 | 1.21 | 1.32 | 1.95 | 1.57 | 1.34 |
| 3.5 | 1.63 | 1.67 | 1.51 | 1.63 | 1.38 | 1.51 | 1.14 | 1.17 | 1.66 | 1.5 | 1.32 |
| 3.75 | 1.29 | 1.44 | 1.51 | 1.42 | 1.28 | 1.29 | 1.21 | 1.17 | 1.61 | 1.43 | 1.22 |
| 4 | 1.32 | 1.17 | 1.5 | 1.47 | 1.27 | 1.3 | 1.16 | 1.14 | 1.52 | 1.34 | 1.27 |
| 4.25 | 1.36 | 1.24 | 1.4 | 1.34 | 1.32 | 1.37 | 1.34 | 1.17 | 1.46 | 1.45 | 1.2 |
| 4.5 | 1.33 | 1.16 | 1.44 | 1.43 | 1.42 | 1.4 | 1.36 | 1.3 | 1.44 | 1.33 | 1.24 |
| 4.75 | 1.32 | 1.11 | 1.44 | 1.24 | 1.41 | 1.33 | 1.4 | 1.35 | 1.33 | 1.45 | 1.2 |
| 5 | 1.21 | 1.03 | 1.47 | 1.34 | 1.45 | 1.31 | 1.51 | 1.36 | 1.42 | 1.41 | 1.17 |
| 5.25 | 1.34 | 1.16 | 1.43 | 1.32 | 1.53 | 1.38 | 1.6 | 1.53 | 1.36 | 1.52 | 1.34 |
| 5.5 | 1.28 | 1.12 | 1.61 | 1.64 | 1.59 | 1.54 | 1.96 | 1.64 | 1.43 | 1.68 | 1.46 |
| 5.75 | 1.26 | 1.35 | 1.81 | 1.95 | 1.91 | 1.67 | 2.11 | 1.99 | 1.75 | 2.01 | 1.68 |
| 6 | 1.56 | 1.77 | 2.31 | 2.66 | 2.39 | 2.37 | 2.81 | 2.5 | 2.07 | 2.57 | 2.07 |
| 6.25 | 2.18 | 1.95 | 2.77 | 2.6 | 2.61 | 2.49 | 3.39 | 2.94 | 2.4 | 3.1 | 2.22 |
| 6.5 | 2.24 | 2.4 | 3.18 | 3.96 | 3.33 | 3.06 | 3.54 | 3.31 | 2.72 | 3.71 | 2.83 |
| 6.75 | 3.33 | 3.03 | 3.92 | 4.18 | 3.44 | 3.59 | 4.09 | 3.52 | 3.11 | 4.04 | 3.47 |
| 7 | 3.95 | 4.02 | 4.21 | 4.79 | 4.3 | 4 | 4.1 | 3.64 | 3.41 | 4.22 | 4.19 |
| 7.25 | 4.38 | 4.77 | 4.03 | 4.46 | 4.05 | 3.96 | 3.99 | 3.58 | 3.62 | 4.67 | 4.44 |
| 7.5 | 4.5 | 5.03 | 4.05 | 4.33 | 3.56 | 4.32 | 4.27 | 3.51 | 3.98 | 4.56 | 5.26 |
| 7.75 | 3.78 | 4.53 | 3.53 | 3.46 | 3.78 | 3.61 | 3.57 | 3.42 | 4.22 | 4 | 4.76 |
| 8 | 3.6 | 4.39 | 3.38 | 3.04 | 3.32 | 3.49 | 3.37 | 2.9 | 3.58 | 3.57 | 4.23 |
| 8.25 | 2.92 | 3.33 | 3.01 | 2.48 | 2.66 | 2.99 | 2.74 | 2.56 | 2.93 | 3.07 | 3.2 |
| 8.5 | 2.39 | 2.13 | 2.4 | 1.72 | 2.19 | 2.74 | 2.38 | 2.15 | 2.7 | 2.58 | 2.77 |
| 8.75 | 1.72 | 1.88 | 2.08 | 1.66 | 1.91 | 1.98 | 2.07 | 1.76 | 2.09 | 2.15 | 2.37 |
| 9 | 1.65 | 1.61 | 1.8 | 1.38 | 1.72 | 1.9 | 1.72 | 1.59 | 1.58 | 1.77 | 1.92 |
| 9.25 | 1.24 | 1.25 | 1.55 | 1.06 | 1.34 | 1.39 | 1.55 | 1.41 | 1.5 | 1.55 | 1.58 |
| 9.5 | 1.3 | 1 | 1.38 | 0.86 | 1.12 | 1.29 | 1.34 | 1.2 | 1.21 | 1.31 | 1.33 |
| 9.75 | 1 | 0.94 | 1.18 | 0.76 | 1.09 | 1.17 | 1.21 | 1.06 | 1.03 | 1.17 | 1.18 |

| | | | | | | | | | | | |
|---|---|---|---|---|---|---|---|---|---|---|---|
| 10 | 0.86 | 0.86 | 1.1 | 0.69 | 0.91 | 0.98 | 1.14 | 1.02 | 0.89 | 1 | 1.03 |
| 10.25 | 0.67 | 0.73 | 0.91 | 0.59 | 0.89 | 0.92 | 0.98 | 0.92 | 0.81 | 0.95 | 1 |
| 10.5 | 0.58 | 0.59 | 0.81 | 0.52 | 0.83 | 0.82 | 1.01 | 0.82 | 0.82 | 0.82 | 0.83 |
| 10.75 | 0.55 | 0.57 | 0.76 | 0.49 | 0.7 | 0.75 | 0.85 | 0.75 | 0.7 | 0.72 | 0.75 |
| 11 | 0.54 | 0.44 | 0.67 | 0.44 | 0.65 | 0.62 | 0.84 | 0.69 | 0.59 | 0.69 | 0.7 |
| 11.25 | 0.48 | 0.43 | 0.64 | 0.39 | 0.6 | 0.58 | 0.75 | 0.65 | 0.46 | 0.6 | 0.64 |
| 11.5 | 0.43 | 0.42 | 0.55 | 0.39 | 0.54 | 0.54 | 0.68 | 0.6 | 0.5 | 0.56 | 0.57 |
| 11.75 | 0.41 | 0.39 | 0.52 | 0.42 | 0.54 | 0.51 | 0.63 | 0.53 | 0.47 | 0.5 | 0.52 |
| 12 | 0.42 | 0.44 | 0.49 | 0.39 | 0.46 | 0.48 | 0.59 | 0.53 | 0.43 | 0.45 | 0.47 |
| 12.25 | 0.39 | 0.35 | 0.45 | 0.41 | 0.44 | 0.43 | 0.57 | 0.5 | 0.41 | 0.44 | 0.45 |
| 12.5 | 0.33 | 0.33 | 0.42 | 0.39 | 0.48 | 0.4 | 0.53 | 0.45 | 0.36 | 0.41 | 0.41 |
| 12.75 | 0.38 | 0.34 | 0.4 | 0.42 | 0.4 | 0.38 | 0.51 | 0.46 | 0.37 | 0.39 | 0.41 |
| 13 | 0.31 | 0.35 | 0.41 | 0.44 | 0.39 | 0.39 | 0.5 | 0.43 | 0.37 | 0.36 | 0.38 |
| 13.25 | 0.31 | 0.32 | 0.36 | 0.43 | 0.39 | 0.38 | 0.45 | 0.46 | 0.36 | 0.36 | 0.36 |
| 13.5 | 0.36 | 0.29 | 0.34 | 0.48 | 0.36 | 0.38 | 0.44 | 0.41 | 0.35 | 0.31 | 0.36 |
| 13.75 | 0.36 | 0.31 | 0.33 | 0.49 | 0.35 | 0.38 | 0.43 | 0.37 | 0.32 | 0.31 | 0.34 |
| 14 | 0.35 | 0.28 | 0.32 | 0.54 | 0.35 | 0.33 | 0.38 | 0.37 | 0.32 | 0.3 | 0.32 |
| 14.25 | 0.31 | 0.3 | 0.31 | 0.52 | 0.33 | 0.33 | 0.39 | 0.36 | 0.33 | 0.28 | 0.33 |
| 14.5 | 0.33 | 0.33 | 0.3 | 0.54 | 0.33 | 0.36 | 0.37 | 0.32 | 0.32 | 0.28 | 0.32 |
| 14.75 | 0.37 | 0.3 | 0.29 | 0.55 | 0.32 | 0.32 | 0.36 | 0.34 | 0.3 | 0.27 | 0.3 |
| 15 | 0.35 | 0.3 | 0.27 | 0.52 | 0.29 | 0.31 | 0.34 | 0.29 | 0.31 | 0.24 | 0.29 |
| 15.25 | 0.3 | 0.31 | 0.26 | 0.47 | 0.29 | 0.29 | 0.34 | 0.31 | 0.33 | 0.25 | 0.28 |
| 15.5 | 0.26 | 0.29 | 0.23 | 0.43 | 0.29 | 0.29 | 0.31 | 0.3 | 0.31 | 0.24 | 0.27 |
| 15.75 | 0.26 | 0.29 | 0.23 | 0.4 | 0.28 | 0.27 | 0.3 | 0.29 | 0.29 | 0.24 | 0.27 |
| 16 | 0.28 | 0.26 | 0.23 | 0.4 | 0.27 | 0.28 | 0.28 | 0.26 | 0.27 | 0.21 | 0.24 |
| 16.25 | 0.3 | 0.27 | 0.22 | 0.38 | 0.26 | 0.26 | 0.27 | 0.27 | 0.28 | 0.22 | 0.23 |
| 16.5 | 0.27 | 0.27 | 0.2 | 0.36 | 0.23 | 0.25 | 0.27 | 0.24 | 0.28 | 0.2 | 0.23 |
| 16.75 | 0.27 | 0.23 | 0.21 | 0.31 | 0.23 | 0.24 | 0.24 | 0.25 | 0.25 | 0.2 | 0.22 |
| 17 | 0.25 | 0.23 | 0.19 | 0.3 | 0.21 | 0.23 | 0.24 | 0.24 | 0.22 | 0.19 | 0.21 |
| 17.25 | 0.26 | 0.23 | 0.2 | 0.28 | 0.21 | 0.23 | 0.23 | 0.22 | 0.22 | 0.18 | 0.2 |
| 17.5 | 0.26 | 0.22 | 0.18 | 0.24 | 0.19 | 0.21 | 0.21 | 0.21 | 0.21 | 0.17 | 0.18 |
| 17.75 | 0.23 | 0.2 | 0.18 | 0.26 | 0.19 | 0.21 | 0.21 | 0.2 | 0.21 | 0.16 | 0.17 |
| 18 | 0.21 | 0.19 | 0.17 | 0.23 | 0.2 | 0.21 | 0.19 | 0.19 | 0.2 | 0.16 | 0.18 |
| 18.25 | 0.22 | 0.18 | 0.15 | 0.22 | 0.18 | 0.18 | 0.19 | 0.19 | 0.19 | 0.15 | 0.16 |
| 18.5 | 0.2 | 0.21 | 0.16 | 0.22 | 0.17 | 0.19 | 0.18 | 0.18 | 0.19 | 0.15 | 0.16 |
| 18.75 | 0.21 | 0.19 | 0.15 | 0.19 | 0.17 | 0.18 | 0.18 | 0.18 | 0.19 | 0.14 | 0.15 |
| 19 | 0.19 | 0.18 | 0.14 | 0.2 | 0.16 | 0.19 | 0.16 | 0.17 | 0.18 | 0.13 | 0.14 |
| 19.25 | 0.2 | 0.18 | 0.15 | 0.18 | 0.15 | 0.17 | 0.17 | 0.16 | 0.16 | 0.13 | 0.14 |
| 19.5 | 0.18 | 0.2 | 0.14 | 0.2 | 0.15 | 0.16 | 0.13 | 0.15 | 0.17 | 0.13 | 0.13 |
| 19.75 | 0.17 | 0.18 | 0.13 | 0.17 | 0.14 | 0.15 | 0.14 | 0.14 | 0.17 | 0.12 | 0.13 |
| 20 | 0.14 | 0.16 | 0.13 | 0.17 | 0.14 | 0.15 | 0.14 | 0.14 | 0.16 | 0.11 | 0.12 |

| **TRF** | | | | | | | | | | | | | |
|---|---|---|---|---|---|---|---|---|---|---|---|---|---|
| Hz | | | | | | | | | | | | | |
| 0.25 | 0.73 | 1.12 | 1.18 | 1.22 | 2.47 | 10.79 | 1.67 | 6.29 | 4.11 | 13.65 | 5.17 | 4.09 | 1.97 |

| | | | | | | | | | | | | | |
|---|---|---|---|---|---|---|---|---|---|---|---|---|---|
| 0.5 | 1.28 | 1.3 | 1.55 | 1.61 | 1.87 | 2.9 | 2.47 | 2.01 | 2.62 | 2.8 | 1.54 | 1.3 | 1.6 |
| 0.75 | 1.31 | 1.25 | 1.46 | 1.74 | 1.9 | 1.81 | 2.29 | 1.61 | 1.98 | 1.51 | 1.28 | 1.23 | 1.33 |
| 1 | 1.28 | 1.15 | 1.55 | 1.53 | 1.61 | 1.66 | 2.23 | 1.54 | 1.89 | 1.24 | 1.28 | 1.28 | 1.18 |
| 1.25 | 1.35 | 1.1 | 1.46 | 1.39 | 1.59 | 1.67 | 1.94 | 1.33 | 1.78 | 1.14 | 1.27 | 1.28 | 1.06 |
| 1.5 | 1.49 | 1.1 | 1.48 | 1.37 | 1.38 | 1.65 | 1.86 | 1.3 | 1.74 | 1.14 | 1.42 | 1.32 | 1.12 |
| 1.75 | 1.52 | 1.08 | 1.6 | 1.42 | 1.58 | 1.53 | 1.9 | 1.43 | 1.77 | 1.03 | 1.47 | 1.46 | 1.13 |
| 2 | 1.73 | 1.1 | 1.54 | 1.33 | 1.44 | 1.46 | 2.11 | 1.54 | 1.79 | 1.09 | 1.53 | 1.46 | 1.13 |
| 2.25 | 1.73 | 1.16 | 1.59 | 1.44 | 1.51 | 1.62 | 2.35 | 1.38 | 1.74 | 1.11 | 1.57 | 1.54 | 1.33 |
| 2.5 | 1.68 | 1.17 | 1.56 | 1.43 | 1.39 | 1.58 | 2.33 | 1.41 | 1.77 | 1.06 | 1.53 | 1.52 | 1.5 |
| 2.75 | 1.65 | 1.07 | 1.54 | 1.3 | 1.51 | 1.55 | 2.39 | 1.3 | 1.67 | 1.03 | 1.59 | 1.4 | 1.56 |
| 3 | 1.53 | 1.12 | 1.37 | 1.2 | 1.42 | 1.45 | 2.41 | 1.32 | 1.54 | 0.97 | 1.4 | 1.41 | 1.81 |
| 3.25 | 1.29 | 1.1 | 1.5 | 1.21 | 1.4 | 1.45 | 2.16 | 1.32 | 1.47 | 0.95 | 1.43 | 1.38 | 2.04 |
| 3.5 | 1.18 | 0.98 | 1.45 | 1.15 | 1.12 | 1.37 | 2.02 | 1.11 | 1.42 | 0.87 | 1.44 | 1.3 | 2.09 |
| 3.75 | 1.03 | 1.03 | 1.32 | 1.1 | 1.11 | 1.15 | 2.18 | 1.2 | 1.37 | 0.89 | 1.35 | 1.26 | 1.95 |
| 4 | 1.09 | 1.08 | 1.31 | 1.09 | 1.12 | 1.15 | 1.84 | 1.19 | 1.3 | 0.82 | 1.3 | 1.3 | 1.8 |
| 4.25 | 1.02 | 1.14 | 1.32 | 1.11 | 1.05 | 1.24 | 1.96 | 1.27 | 1.3 | 0.88 | 1.33 | 1.39 | 1.75 |
| 4.5 | 1 | 1.18 | 1.22 | 1.1 | 1.11 | 1.12 | 1.88 | 1.21 | 1.25 | 0.89 | 1.36 | 1.41 | 1.53 |
| 4.75 | 1.03 | 1.24 | 1.29 | 1.26 | 1.24 | 1.16 | 1.82 | 1.22 | 1.23 | 0.83 | 1.37 | 1.41 | 1.31 |
| 5 | 1.2 | 1.38 | 1.27 | 1.26 | 1.16 | 1.24 | 1.75 | 1.29 | 1.21 | 0.89 | 1.39 | 1.47 | 1.28 |
| 5.25 | 1.39 | 1.7 | 1.35 | 1.4 | 1.25 | 1.09 | 1.69 | 1.28 | 1.14 | 0.91 | 1.35 | 1.5 | 1.21 |
| 5.5 | 1.44 | 1.98 | 1.46 | 1.61 | 1.39 | 1.15 | 1.71 | 1.43 | 1.23 | 0.9 | 1.45 | 1.63 | 1.25 |
| 5.75 | 1.82 | 2.5 | 1.69 | 1.97 | 1.7 | 1.31 | 1.73 | 1.64 | 1.25 | 1.05 | 1.49 | 1.88 | 1.33 |
| 6 | 2.48 | 3.15 | 2.15 | 2.38 | 2.05 | 1.64 | 1.79 | 1.87 | 1.63 | 1.26 | 1.86 | 2.36 | 1.82 |
| 6.25 | 3.09 | 3.76 | 2.54 | 3.02 | 2.69 | 1.7 | 1.9 | 2.11 | 1.82 | 1.46 | 2.25 | 2.85 | 2.14 |
| 6.5 | 4.22 | 4.79 | 3.12 | 4.09 | 3.42 | 2.06 | 1.93 | 2.51 | 2.37 | 1.95 | 2.84 | 3.31 | 2.74 |
| 6.75 | 4.43 | 5.46 | 4.03 | 4.42 | 4.17 | 2.98 | 2.34 | 3.27 | 2.99 | 2.3 | 3.29 | 3.71 | 3.41 |
| 7 | 4.99 | 5.7 | 4.33 | 4.97 | 4.84 | 3.73 | 2.6 | 3.49 | 3.92 | 2.86 | 3.71 | 3.62 | 3.61 |
| 7.25 | 4.94 | 5.29 | 4.71 | 5.53 | 5.36 | 4.66 | 2.79 | 3.69 | 4.55 | 2.91 | 3.63 | 3.87 | 4.13 |
| 7.5 | 4.58 | 4.88 | 4.6 | 5.25 | 5.37 | 4.51 | 3.04 | 4.01 | 4.68 | 2.88 | 3.79 | 3.7 | 4.32 |
| 7.75 | 3.82 | 3.98 | 4.1 | 4.55 | 4.46 | 4.03 | 2.98 | 4.27 | 4.54 | 2.95 | 3.49 | 3.68 | 3.65 |
| 8 | 3.39 | 3.51 | 3.9 | 3.76 | 3.64 | 3.76 | 2.64 | 3.76 | 4.19 | 2.69 | 3.24 | 3.39 | 3.13 |
| 8.25 | 2.73 | 2.85 | 3.22 | 3.09 | 2.95 | 2.51 | 2.26 | 3.54 | 2.84 | 2.38 | 3.09 | 3.22 | 2.79 |
| 8.5 | 2.37 | 2.34 | 2.58 | 2.5 | 2.62 | 1.93 | 1.89 | 2.78 | 2.47 | 1.99 | 2.38 | 3.06 | 2.3 |
| 8.75 | 1.93 | 2.2 | 2.25 | 2.15 | 2.11 | 1.58 | 1.5 | 2.3 | 2 | 1.65 | 2.15 | 2.38 | 1.97 |
| 9 | 1.58 | 1.76 | 1.86 | 1.71 | 1.76 | 1.27 | 1.3 | 1.67 | 1.81 | 1.4 | 1.74 | 2.16 | 1.52 |
| 9.25 | 1.43 | 1.55 | 1.48 | 1.4 | 1.42 | 1.06 | 1.17 | 1.5 | 1.36 | 1.13 | 1.55 | 1.73 | 1.48 |
| 9.5 | 1.2 | 1.31 | 1.27 | 1.22 | 1.22 | 0.89 | 1.09 | 1.25 | 1.12 | 0.97 | 1.28 | 1.47 | 1.22 |
| 9.75 | 1.02 | 1.12 | 1.15 | 1.07 | 1 | 0.75 | 0.99 | 1.14 | 1.04 | 0.91 | 1.11 | 1.22 | 1.09 |
| 10 | 0.93 | 1.02 | 0.97 | 0.96 | 0.9 | 0.69 | 0.83 | 0.98 | 0.81 | 0.75 | 1.01 | 1.18 | 0.95 |
| 10.25 | 0.84 | 0.89 | 0.88 | 0.84 | 0.81 | 0.57 | 0.76 | 0.92 | 0.76 | 0.71 | 0.9 | 1.05 | 0.79 |
| 10.5 | 0.78 | 0.84 | 0.82 | 0.76 | 0.68 | 0.55 | 0.7 | 0.77 | 0.67 | 0.6 | 0.84 | 0.91 | 0.76 |
| 10.75 | 0.66 | 0.72 | 0.76 | 0.65 | 0.63 | 0.46 | 0.66 | 0.72 | 0.61 | 0.57 | 0.79 | 0.81 | 0.67 |
| 11 | 0.58 | 0.66 | 0.69 | 0.6 | 0.58 | 0.42 | 0.63 | 0.67 | 0.52 | 0.49 | 0.7 | 0.72 | 0.6 |
| 11.25 | 0.55 | 0.61 | 0.58 | 0.52 | 0.52 | 0.42 | 0.61 | 0.62 | 0.56 | 0.43 | 0.7 | 0.66 | 0.54 |
| 11.5 | 0.51 | 0.55 | 0.56 | 0.48 | 0.5 | 0.44 | 0.57 | 0.61 | 0.5 | 0.44 | 0.66 | 0.58 | 0.48 |
| 11.75 | 0.48 | 0.49 | 0.51 | 0.45 | 0.41 | 0.37 | 0.53 | 0.54 | 0.47 | 0.4 | 0.6 | 0.55 | 0.45 |
| 12 | 0.42 | 0.44 | 0.46 | 0.43 | 0.39 | 0.36 | 0.49 | 0.51 | 0.44 | 0.36 | 0.54 | 0.49 | 0.4 |
| 12.25 | 0.38 | 0.41 | 0.44 | 0.38 | 0.37 | 0.38 | 0.51 | 0.46 | 0.41 | 0.34 | 0.51 | 0.48 | 0.36 |

| | | | | | | | | | | | | | |
|---|---|---|---|---|---|---|---|---|---|---|---|---|---|
| 12.5 | 0.39 | 0.4 | 0.41 | 0.37 | 0.34 | 0.35 | 0.44 | 0.48 | 0.39 | 0.32 | 0.48 | 0.44 | 0.35 |
| 12.75 | 0.37 | 0.38 | 0.38 | 0.33 | 0.31 | 0.34 | 0.44 | 0.4 | 0.39 | 0.32 | 0.49 | 0.4 | 0.36 |
| 13 | 0.34 | 0.36 | 0.41 | 0.32 | 0.33 | 0.32 | 0.41 | 0.4 | 0.34 | 0.31 | 0.46 | 0.39 | 0.31 |
| 13.25 | 0.32 | 0.35 | 0.36 | 0.31 | 0.3 | 0.31 | 0.42 | 0.36 | 0.34 | 0.28 | 0.44 | 0.36 | 0.31 |
| 13.5 | 0.34 | 0.31 | 0.34 | 0.3 | 0.29 | 0.33 | 0.39 | 0.39 | 0.31 | 0.29 | 0.42 | 0.35 | 0.3 |
| 13.75 | 0.32 | 0.32 | 0.34 | 0.29 | 0.29 | 0.31 | 0.39 | 0.38 | 0.34 | 0.28 | 0.44 | 0.33 | 0.28 |
| 14 | 0.31 | 0.3 | 0.32 | 0.28 | 0.28 | 0.32 | 0.35 | 0.36 | 0.3 | 0.29 | 0.42 | 0.31 | 0.28 |
| 14.25 | 0.28 | 0.28 | 0.31 | 0.27 | 0.28 | 0.35 | 0.38 | 0.38 | 0.32 | 0.26 | 0.41 | 0.31 | 0.3 |
| 14.5 | 0.28 | 0.28 | 0.32 | 0.27 | 0.27 | 0.35 | 0.36 | 0.34 | 0.31 | 0.26 | 0.41 | 0.29 | 0.27 |
| 14.75 | 0.28 | 0.27 | 0.29 | 0.25 | 0.27 | 0.33 | 0.37 | 0.32 | 0.29 | 0.25 | 0.36 | 0.29 | 0.29 |
| 15 | 0.27 | 0.25 | 0.29 | 0.25 | 0.25 | 0.35 | 0.31 | 0.33 | 0.28 | 0.23 | 0.36 | 0.27 | 0.28 |
| 15.25 | 0.24 | 0.25 | 0.27 | 0.25 | 0.26 | 0.35 | 0.33 | 0.31 | 0.28 | 0.24 | 0.34 | 0.25 | 0.29 |
| 15.5 | 0.23 | 0.22 | 0.28 | 0.24 | 0.25 | 0.35 | 0.33 | 0.3 | 0.26 | 0.23 | 0.34 | 0.24 | 0.25 |
| 15.75 | 0.23 | 0.23 | 0.25 | 0.22 | 0.23 | 0.28 | 0.32 | 0.29 | 0.25 | 0.24 | 0.32 | 0.24 | 0.25 |
| 16 | 0.22 | 0.21 | 0.26 | 0.22 | 0.22 | 0.29 | 0.3 | 0.29 | 0.25 | 0.24 | 0.33 | 0.24 | 0.24 |
| 16.25 | 0.23 | 0.2 | 0.24 | 0.2 | 0.22 | 0.29 | 0.29 | 0.28 | 0.23 | 0.21 | 0.29 | 0.23 | 0.22 |
| 16.5 | 0.22 | 0.19 | 0.23 | 0.2 | 0.21 | 0.28 | 0.31 | 0.27 | 0.23 | 0.22 | 0.28 | 0.21 | 0.21 |
| 16.75 | 0.19 | 0.18 | 0.21 | 0.19 | 0.2 | 0.25 | 0.27 | 0.25 | 0.21 | 0.21 | 0.28 | 0.21 | 0.2 |
| 17 | 0.18 | 0.18 | 0.21 | 0.18 | 0.19 | 0.23 | 0.28 | 0.23 | 0.21 | 0.2 | 0.26 | 0.2 | 0.19 |
| 17.25 | 0.18 | 0.17 | 0.2 | 0.18 | 0.2 | 0.2 | 0.26 | 0.23 | 0.19 | 0.19 | 0.24 | 0.19 | 0.2 |
| 17.5 | 0.18 | 0.16 | 0.18 | 0.17 | 0.2 | 0.2 | 0.28 | 0.22 | 0.2 | 0.19 | 0.23 | 0.19 | 0.19 |
| 17.75 | 0.17 | 0.14 | 0.2 | 0.16 | 0.18 | 0.21 | 0.23 | 0.21 | 0.17 | 0.17 | 0.22 | 0.18 | 0.17 |
| 18 | 0.16 | 0.14 | 0.17 | 0.16 | 0.17 | 0.19 | 0.24 | 0.19 | 0.18 | 0.17 | 0.22 | 0.17 | 0.17 |
| 18.25 | 0.16 | 0.13 | 0.18 | 0.15 | 0.16 | 0.18 | 0.23 | 0.2 | 0.16 | 0.16 | 0.21 | 0.16 | 0.17 |
| 18.5 | 0.15 | 0.13 | 0.17 | 0.15 | 0.15 | 0.16 | 0.22 | 0.17 | 0.17 | 0.15 | 0.2 | 0.15 | 0.17 |
| 18.75 | 0.15 | 0.13 | 0.16 | 0.14 | 0.15 | 0.15 | 0.21 | 0.17 | 0.15 | 0.16 | 0.2 | 0.15 | 0.15 |
| 19 | 0.15 | 0.12 | 0.15 | 0.14 | 0.14 | 0.17 | 0.23 | 0.16 | 0.16 | 0.14 | 0.19 | 0.14 | 0.15 |
| 19.25 | 0.14 | 0.12 | 0.14 | 0.13 | 0.15 | 0.14 | 0.22 | 0.16 | 0.15 | 0.14 | 0.18 | 0.13 | 0.15 |
| 19.5 | 0.13 | 0.12 | 0.15 | 0.13 | 0.14 | 0.14 | 0.21 | 0.15 | 0.15 | 0.14 | 0.17 | 0.13 | 0.16 |
| 19.75 | 0.13 | 0.12 | 0.14 | 0.13 | 0.13 | 0.14 | 0.2 | 0.15 | 0.14 | 0.12 | 0.17 | 0.12 | 0.14 |
| 20 | 0.13 | 0.11 | 0.15 | 0.12 | 0.13 | 0.14 | 0.19 | 0.14 | 0.13 | 0.13 | 0.16 | 0.12 | 0.15 |

| **TRM** | | | | | | | | | | | | | | | |
|---|---|---|---|---|---|---|---|---|---|---|---|---|---|---|---|
| Hz | | | | | | | | | | | | | | | |
| 0.25 | 3.25 | 1.12 | 1.92 | 2.2 | 1.82 | 1.83 | 1.69 | 2.83 | 3.06 | 2.29 | 1.08 | 0.99 | 1.16 | 2.31 | 0.88 |
| 0.5 | 2.29 | 1.5 | 2.16 | 2.9 | 1.21 | 2.26 | 1.7 | 1.71 | 2.42 | 2.19 | 1.28 | 1.32 | 1.41 | 1.96 | 1.26 |
| 0.75 | 2.07 | 1.57 | 2.02 | 2.47 | 1.11 | 2.01 | 1.71 | 1.34 | 1.88 | 1.96 | 1.13 | 1.45 | 1.5 | 1.74 | 1.22 |
| 1 | 1.55 | 1.63 | 1.87 | 1.92 | 1.12 | 1.7 | 1.61 | 1.3 | 1.79 | 1.77 | 1.18 | 1.31 | 1.4 | 1.66 | 1.25 |
| 1.25 | 1.64 | 1.71 | 1.55 | 1.68 | 0.99 | 1.81 | 1.68 | 1.25 | 1.6 | 1.61 | 1.08 | 1.27 | 1.45 | 1.67 | 1.25 |
| 1.5 | 1.53 | 1.76 | 1.43 | 1.64 | 1.08 | 1.54 | 1.59 | 1.17 | 1.6 | 1.51 | 1.16 | 1.28 | 1.52 | 1.62 | 1.32 |
| 1.75 | 1.68 | 1.95 | 1.5 | 1.83 | 1.05 | 1.42 | 1.62 | 1.28 | 1.51 | 1.62 | 1.2 | 1.27 | 1.61 | 1.7 | 1.37 |
| 2 | 1.69 | 2.28 | 1.5 | 1.51 | 1.02 | 1.49 | 1.65 | 1.42 | 1.48 | 1.71 | 1.32 | 1.38 | 1.66 | 1.67 | 1.41 |
| 2.25 | 1.66 | 2.37 | 1.5 | 1.67 | 1.15 | 1.54 | 1.76 | 1.63 | 1.48 | 1.72 | 1.46 | 1.37 | 1.74 | 1.69 | 1.47 |
| 2.5 | 1.87 | 2.23 | 1.75 | 1.85 | 1.14 | 1.52 | 1.82 | 1.82 | 1.41 | 1.67 | 1.36 | 1.35 | 1.6 | 1.68 | 1.38 |
| 2.75 | 1.72 | 2.15 | 1.69 | 1.77 | 1.17 | 1.68 | 1.87 | 1.64 | 1.45 | 1.67 | 1.3 | 1.29 | 1.61 | 1.58 | 1.43 |
| 3 | 1.7 | 2.01 | 1.38 | 1.84 | 1.07 | 1.49 | 1.61 | 1.61 | 1.4 | 1.59 | 1.36 | 1.33 | 1.57 | 1.63 | 1.21 |
| 3.25 | 1.54 | 1.85 | 1.4 | 1.65 | 1 | 1.48 | 1.64 | 1.48 | 1.51 | 1.67 | 1.29 | 1.24 | 1.5 | 1.55 | 1.28 |

| | | | | | | | | | | | | | | | | |
|---|---|---|---|---|---|---|---|---|---|---|---|---|---|---|---|---|
| 3.5 | 1.61 | 1.72 | 1.29 | 1.49 | 0.94 | 1.5 | 1.55 | 1.21 | 1.4 | 1.53 | 1.13 | 1.18 | 1.52 | 1.41 | 1.24 |
| 3.75 | 1.43 | 1.61 | 1.21 | 1.34 | 0.91 | 1.35 | 1.47 | 1.14 | 1.27 | 1.43 | 1.17 | 1.17 | 1.44 | 1.34 | 1.21 |
| 4 | 1.46 | 1.63 | 1.06 | 1.24 | 0.9 | 1.3 | 1.42 | 0.98 | 1.18 | 1.36 | 1.2 | 1.27 | 1.43 | 1.27 | 1.2 |
| 4.25 | 1.56 | 1.54 | 1.08 | 1.13 | 0.83 | 1.27 | 1.41 | 1.01 | 1.37 | 1.38 | 1.22 | 1.26 | 1.55 | 1.19 | 1.25 |
| 4.5 | 1.57 | 1.58 | 1.08 | 1.19 | 0.95 | 1.22 | 1.48 | 1.1 | 1.29 | 1.34 | 1.25 | 1.29 | 1.49 | 1.24 | 1.34 |
| 4.75 | 1.51 | 1.5 | 1.14 | 1.12 | 1.02 | 1.17 | 1.51 | 0.94 | 1.35 | 1.3 | 1.16 | 1.47 | 1.56 | 1.2 | 1.45 |
| 5 | 1.34 | 1.51 | 1.11 | 1.2 | 1.04 | 1.24 | 1.33 | 1.16 | 1.34 | 1.41 | 1.29 | 1.6 | 1.58 | 1.17 | 1.69 |
| 5.25 | 1.35 | 1.46 | 1.23 | 1.22 | 1.05 | 1.1 | 1.38 | 1.23 | 1.41 | 1.47 | 1.3 | 1.8 | 1.7 | 1.23 | 1.94 |
| 5.5 | 1.59 | 1.57 | 1.31 | 1.33 | 1.4 | 1.23 | 1.61 | 1.3 | 1.6 | 1.57 | 1.54 | 2.15 | 1.89 | 1.45 | 2.2 |
| 5.75 | 1.58 | 1.59 | 1.76 | 1.51 | 1.54 | 1.35 | 1.83 | 1.53 | 1.75 | 1.84 | 1.66 | 2.46 | 2.11 | 1.55 | 2.65 |
| 6 | 2.07 | 1.83 | 2.18 | 1.97 | 2.25 | 1.59 | 2.21 | 1.89 | 2.28 | 2.47 | 2.18 | 3.49 | 2.82 | 2.2 | 3.41 |
| 6.25 | 2.38 | 2.16 | 3.01 | 2.53 | 2.36 | 1.98 | 2.65 | 2.34 | 2.45 | 2.88 | 2.76 | 4.32 | 3.43 | 2.63 | 4.01 |
| 6.5 | 2.74 | 2.72 | 3.63 | 3.16 | 2.65 | 2.26 | 3.07 | 2.88 | 3.11 | 3.6 | 3.32 | 4.69 | 3.85 | 3.09 | 4.26 |
| 6.75 | 3.24 | 2.83 | 4.39 | 3.47 | 2.91 | 2.48 | 3.7 | 3.36 | 3.78 | 4.18 | 3.75 | 5.21 | 4.22 | 3.51 | 4.2 |
| 7 | 3.39 | 3.06 | 4.71 | 4.23 | 2.94 | 3.03 | 3.87 | 3.54 | 3.98 | 4.31 | 3.83 | 4.89 | 4.23 | 4.02 | 4.5 |
| 7.25 | 3.79 | 3.35 | 4.65 | 4.81 | 3.01 | 3.65 | 4.13 | 3.67 | 4.44 | 4.29 | 3.87 | 4.83 | 3.98 | 4.52 | 4.52 |
| 7.5 | 4.12 | 3.34 | 4.77 | 4.52 | 2.7 | 3.88 | 3.78 | 4.09 | 4.23 | 3.95 | 3.92 | 4.19 | 3.81 | 4.4 | 4.17 |
| 7.75 | 3.73 | 3.35 | 4.38 | 3.88 | 2.26 | 4.08 | 3.81 | 3.79 | 4.32 | 3.49 | 3.47 | 3.63 | 3.07 | 4.21 | 3.78 |
| 8 | 3.53 | 2.96 | 3.44 | 3.59 | 1.87 | 4.06 | 3.4 | 3.19 | 3.54 | 2.77 | 2.83 | 3.05 | 2.69 | 3.54 | 3.12 |
| 8.25 | 2.83 | 2.76 | 2.95 | 2.92 | 1.61 | 3.55 | 2.71 | 3.05 | 3.25 | 2.44 | 2.89 | 2.51 | 2.26 | 3.16 | 2.72 |
| 8.5 | 2.43 | 2.33 | 2.38 | 2.27 | 1.32 | 2.95 | 2.38 | 2.39 | 2.66 | 1.81 | 2.37 | 2.1 | 1.99 | 2.51 | 2.33 |
| 8.75 | 1.88 | 2.16 | 2.05 | 1.86 | 1.17 | 2.39 | 1.98 | 2.21 | 2.18 | 1.63 | 1.76 | 1.78 | 1.74 | 2.16 | 2 |
| 9 | 1.62 | 1.78 | 1.81 | 1.48 | 1.06 | 1.83 | 1.66 | 1.84 | 1.77 | 1.35 | 1.41 | 1.52 | 1.58 | 1.68 | 1.68 |
| 9.25 | 1.18 | 1.43 | 1.37 | 1.39 | 0.9 | 1.64 | 1.34 | 1.43 | 1.58 | 1.15 | 1.34 | 1.3 | 1.42 | 1.48 | 1.42 |
| 9.5 | 1.11 | 1.21 | 1.32 | 1.12 | 0.78 | 1.3 | 1.2 | 1.19 | 1.31 | 1.02 | 1.21 | 1.13 | 1.14 | 1.22 | 1.19 |
| 9.75 | 1.02 | 1.17 | 1.12 | 0.87 | 0.67 | 1.09 | 1.01 | 1.14 | 1.04 | 0.83 | 1.04 | 1.01 | 1.05 | 1.13 | 1.06 |
| 10 | 0.95 | 0.98 | 0.91 | 0.83 | 0.68 | 0.94 | 0.94 | 1.08 | 0.91 | 0.81 | 0.82 | 0.91 | 0.95 | 0.82 | 0.94 |
| 10.25 | 0.88 | 0.87 | 0.78 | 0.73 | 0.61 | 0.84 | 0.81 | 0.89 | 0.76 | 0.8 | 0.8 | 0.79 | 0.91 | 0.77 | 0.87 |
| 10.5 | 0.72 | 0.81 | 0.73 | 0.65 | 0.51 | 0.82 | 0.76 | 0.77 | 0.72 | 0.74 | 0.7 | 0.72 | 0.79 | 0.71 | 0.78 |
| 10.75 | 0.63 | 0.76 | 0.64 | 0.54 | 0.44 | 0.65 | 0.68 | 0.7 | 0.64 | 0.67 | 0.7 | 0.67 | 0.75 | 0.63 | 0.7 |
| 11 | 0.59 | 0.66 | 0.6 | 0.55 | 0.43 | 0.66 | 0.64 | 0.65 | 0.58 | 0.62 | 0.57 | 0.6 | 0.68 | 0.58 | 0.57 |
| 11.25 | 0.53 | 0.64 | 0.53 | 0.47 | 0.37 | 0.58 | 0.58 | 0.54 | 0.53 | 0.58 | 0.59 | 0.57 | 0.64 | 0.55 | 0.55 |
| 11.5 | 0.48 | 0.6 | 0.48 | 0.46 | 0.35 | 0.57 | 0.53 | 0.53 | 0.52 | 0.59 | 0.52 | 0.54 | 0.61 | 0.48 | 0.53 |
| 11.75 | 0.44 | 0.55 | 0.42 | 0.4 | 0.36 | 0.5 | 0.5 | 0.53 | 0.43 | 0.5 | 0.52 | 0.47 | 0.56 | 0.47 | 0.5 |
| 12 | 0.46 | 0.5 | 0.39 | 0.4 | 0.34 | 0.48 | 0.46 | 0.41 | 0.41 | 0.49 | 0.48 | 0.41 | 0.53 | 0.43 | 0.44 |
| 12.25 | 0.43 | 0.48 | 0.38 | 0.36 | 0.32 | 0.47 | 0.46 | 0.43 | 0.4 | 0.45 | 0.42 | 0.41 | 0.49 | 0.4 | 0.43 |
| 12.5 | 0.4 | 0.45 | 0.36 | 0.35 | 0.3 | 0.43 | 0.44 | 0.39 | 0.38 | 0.4 | 0.38 | 0.39 | 0.49 | 0.36 | 0.45 |
| 12.75 | 0.37 | 0.44 | 0.33 | 0.35 | 0.27 | 0.46 | 0.4 | 0.38 | 0.34 | 0.43 | 0.39 | 0.38 | 0.45 | 0.35 | 0.42 |
| 13 | 0.34 | 0.4 | 0.32 | 0.33 | 0.28 | 0.42 | 0.38 | 0.34 | 0.33 | 0.41 | 0.42 | 0.37 | 0.43 | 0.35 | 0.41 |
| 13.25 | 0.32 | 0.41 | 0.31 | 0.33 | 0.27 | 0.4 | 0.37 | 0.35 | 0.31 | 0.4 | 0.36 | 0.32 | 0.42 | 0.31 | 0.37 |
| 13.5 | 0.31 | 0.39 | 0.3 | 0.28 | 0.25 | 0.38 | 0.34 | 0.37 | 0.3 | 0.4 | 0.36 | 0.33 | 0.42 | 0.29 | 0.36 |
| 13.75 | 0.35 | 0.37 | 0.27 | 0.3 | 0.25 | 0.42 | 0.38 | 0.34 | 0.28 | 0.37 | 0.34 | 0.31 | 0.4 | 0.3 | 0.32 |
| 14 | 0.31 | 0.37 | 0.28 | 0.29 | 0.25 | 0.36 | 0.34 | 0.31 | 0.29 | 0.37 | 0.35 | 0.29 | 0.37 | 0.29 | 0.33 |
| 14.25 | 0.29 | 0.37 | 0.3 | 0.3 | 0.24 | 0.38 | 0.34 | 0.31 | 0.28 | 0.31 | 0.32 | 0.3 | 0.36 | 0.31 | 0.32 |
| 14.5 | 0.32 | 0.35 | 0.28 | 0.28 | 0.24 | 0.38 | 0.33 | 0.31 | 0.27 | 0.32 | 0.33 | 0.28 | 0.36 | 0.26 | 0.33 |
| 14.75 | 0.31 | 0.35 | 0.28 | 0.28 | 0.22 | 0.36 | 0.29 | 0.3 | 0.27 | 0.31 | 0.3 | 0.28 | 0.33 | 0.26 | 0.32 |
| 15 | 0.3 | 0.34 | 0.28 | 0.27 | 0.21 | 0.39 | 0.31 | 0.28 | 0.27 | 0.33 | 0.31 | 0.25 | 0.32 | 0.26 | 0.28 |
| 15.25 | 0.27 | 0.32 | 0.27 | 0.27 | 0.2 | 0.36 | 0.32 | 0.26 | 0.27 | 0.31 | 0.3 | 0.25 | 0.3 | 0.25 | 0.27 |

| | | | | | | | | | | | | | | | |
|---|---|---|---|---|---|---|---|---|---|---|---|---|---|---|---|
| 15.5 | 0.31 | 0.33 | 0.25 | 0.28 | 0.19 | 0.35 | 0.31 | 0.27 | 0.25 | 0.29 | 0.27 | 0.23 | 0.29 | 0.25 | 0.27 |
| 15.75 | 0.28 | 0.32 | 0.24 | 0.25 | 0.2 | 0.35 | 0.29 | 0.26 | 0.24 | 0.28 | 0.26 | 0.22 | 0.29 | 0.25 | 0.26 |
| 16 | 0.27 | 0.29 | 0.23 | 0.26 | 0.2 | 0.35 | 0.26 | 0.25 | 0.23 | 0.26 | 0.27 | 0.21 | 0.27 | 0.26 | 0.26 |
| 16.25 | 0.27 | 0.3 | 0.23 | 0.24 | 0.17 | 0.34 | 0.26 | 0.24 | 0.23 | 0.25 | 0.24 | 0.21 | 0.25 | 0.22 | 0.25 |
| 16.5 | 0.24 | 0.27 | 0.2 | 0.22 | 0.16 | 0.33 | 0.25 | 0.24 | 0.22 | 0.24 | 0.24 | 0.18 | 0.24 | 0.23 | 0.23 |
| 16.75 | 0.23 | 0.27 | 0.2 | 0.21 | 0.15 | 0.3 | 0.24 | 0.23 | 0.19 | 0.22 | 0.24 | 0.18 | 0.24 | 0.23 | 0.22 |
| 17 | 0.21 | 0.26 | 0.2 | 0.22 | 0.14 | 0.29 | 0.22 | 0.22 | 0.2 | 0.21 | 0.23 | 0.19 | 0.23 | 0.19 | 0.21 |
| 17.25 | 0.2 | 0.26 | 0.2 | 0.21 | 0.15 | 0.28 | 0.23 | 0.21 | 0.2 | 0.22 | 0.24 | 0.18 | 0.22 | 0.2 | 0.19 |
| 17.5 | 0.2 | 0.24 | 0.18 | 0.18 | 0.13 | 0.29 | 0.22 | 0.21 | 0.18 | 0.21 | 0.21 | 0.18 | 0.19 | 0.18 | 0.19 |
| 17.75 | 0.19 | 0.24 | 0.18 | 0.18 | 0.13 | 0.26 | 0.21 | 0.2 | 0.18 | 0.2 | 0.2 | 0.16 | 0.21 | 0.18 | 0.19 |
| 18 | 0.2 | 0.22 | 0.17 | 0.18 | 0.13 | 0.25 | 0.21 | 0.2 | 0.16 | 0.2 | 0.19 | 0.15 | 0.19 | 0.18 | 0.18 |
| 18.25 | 0.2 | 0.2 | 0.17 | 0.17 | 0.11 | 0.25 | 0.19 | 0.2 | 0.18 | 0.18 | 0.18 | 0.16 | 0.18 | 0.17 | 0.18 |
| 18.5 | 0.18 | 0.2 | 0.15 | 0.17 | 0.11 | 0.23 | 0.18 | 0.17 | 0.16 | 0.17 | 0.18 | 0.15 | 0.18 | 0.16 | 0.16 |
| 18.75 | 0.17 | 0.19 | 0.16 | 0.16 | 0.12 | 0.24 | 0.18 | 0.18 | 0.14 | 0.18 | 0.18 | 0.14 | 0.16 | 0.16 | 0.16 |
| 19 | 0.16 | 0.18 | 0.15 | 0.15 | 0.11 | 0.23 | 0.17 | 0.17 | 0.14 | 0.16 | 0.17 | 0.14 | 0.16 | 0.16 | 0.15 |
| 19.25 | 0.19 | 0.2 | 0.14 | 0.15 | 0.11 | 0.23 | 0.16 | 0.17 | 0.13 | 0.16 | 0.17 | 0.13 | 0.15 | 0.15 | 0.15 |
| 19.5 | 0.16 | 0.18 | 0.14 | 0.13 | 0.11 | 0.21 | 0.16 | 0.16 | 0.13 | 0.15 | 0.16 | 0.12 | 0.16 | 0.15 | 0.14 |
| 19.75 | 0.15 | 0.17 | 0.14 | 0.14 | 0.1 | 0.19 | 0.16 | 0.15 | 0.12 | 0.15 | 0.14 | 0.12 | 0.15 | 0.15 | 0.14 |
| 20 | 0.16 | 0.16 | 0.12 | 0.14 | 0.09 | 0.18 | 0.14 | 0.15 | 0.12 | 0.15 | 0.14 | 0.12 | 0.14 | 0.14 | 0.13 |

Individual median (range) of the EEG peak frequency during NREMS

| CLF | CLM | TRF | TRM |
|---|---|---|---|
| 1 | 1.75 | 1.25 | 1.25 |
| 2 | 1.25 | 1.25 | 2.75 |
| 1.25 | 1.25 | 1.25 | 2.25 |
| 3 | 1.5 | 1.5 | 1.5 |
| 1 | 1 | 1.25 | 1 |
| 1.25 | 2.75 | 1.75 | 1.25 |
| 2 | 2.5 | 1 | 1.5 |
| 1.5 | 1 | 1.25 | 2.25 |
| 1.25 | 0.75 | 1 | 1 |
| 1.75 | 3.5 | 1.25 | 3 |
| 1 | 1.5 | 1.5 | 3 |
| 2.5 | | 1.75 | 1.25 |
| | | 2.75 | 1.5 |
| | | | 1.5 |
| | | | 1 |

Individual median (range) of the EEG peak frequency during REMS

| CLF | CLM | TRF | TRM |
|---|---|---|---|
| 7.25 | 7.5 | 7 | 7.5 |
| 7.75 | 7.5 | 7 | 7.75 |
| 7.5 | 7 | 7.25 | 7.5 |
| 7.5 | 7 | 7.25 | 7.25 |
| 7.75 | 7 | 7.5 | 7.25 |

| | | | |
|---|---|---|---|
| 7.5 | 7.5 | 7.25 | 7.75 |
| 6.25 | 7.5 | 7.5 | 7.25 |
| 7 | 7 | 7.75 | 7.5 |
| 7.25 | 7.75 | 7.5 | 7.25 |
| 7.75 | 7.25 | 7.75 | 7 |
| 7.75 | 7.5 | 7.5 | 7.5 |
| 7.25 | | 7.25 | 6.75 |
| | | 7.5 | 7 |
| | | | 7.25 |
| | | | 7.25 |

Power in the delta frequency range during NREMS in baseline recordings

| **CLF** | | | | | | | | | | | | |
|---|---|---|---|---|---|---|---|---|---|---|---|---|
| ZT | | | | | | | | | | | | |
| 2 | 152.7 | 170.3 | 83.18 | 115.7 | 143 | 142 | 78.84 | 125.4 | 112.3 | 116.8 | 131.8 | 114.5 |
| 4 | 124.4 | 127.1 | 125.9 | 110.4 | 128.7 | 112.8 | 65.99 | 101.5 | 107.5 | 111.4 | 119.6 | 99.41 |
| 6 | 123.9 | 116.4 | 120.5 | 103.8 | 113.3 | 108.2 | 78.51 | 95.18 | 119.2 | 108.4 | 106.9 | 98.94 |
| 8 | 111.7 | 108.4 | 113.6 | 103.5 | 108.6 | 101.5 | 78.11 | 101.8 | 94.82 | 103.5 | 106.9 | 102.7 |
| 10 | 104.1 | 102.1 | 102.5 | 100.5 | 99.85 | 100.3 | 99.33 | 96.35 | 92.52 | 98.42 | 96.48 | 101.3 |
| 12 | 95.93 | 97.89 | 97.55 | 99.48 | 100.2 | 99.72 | 100.7 | 103.7 | 107.5 | 101.6 | 103.5 | 98.73 |
| 14 | 100.5 | 106.5 | 109.7 | 104.8 | 109.3 | 133.5 | 70.02 | 105.5 | 114.9 | 106.5 | *9999* | 103.4 |
| 16 | 120.3 | 114.1 | 119.1 | 138.9 | 133.6 | 127 | 67.43 | 97.67 | 175 | 137.2 | 142.1 | 128.4 |
| 18 | 134.1 | 115.3 | 70.31 | 160.6 | 124.8 | 106.9 | 105.2 | 139 | 167.8 | 45.1 | *9999* | 210.4 |
| 20 | 137.8 | 140.3 | 121.4 | 132.5 | 158.9 | 172.2 | 97.91 | 129.5 | 66.86 | 171.2 | 91.75 | 116.2 |
| 22 | 138.2 | 75.83 | 93.59 | 138.9 | 159.2 | 153.6 | 86.01 | 140.7 | 76.75 | 146.2 | 156.6 | 149.4 |
| 24 | 100.1 | 43.38 | 51.22 | 133.4 | 187.2 | 111.7 | 79.13 | *9999* | 121.6 | 150.6 | 115 | 134.3 |

| **CLM** | | | | | | | | | | | |
|---|---|---|---|---|---|---|---|---|---|---|---|
| ZT | | | | | | | | | | | |
| 2 | 101.5 | 180.3 | 123.5 | 135.4 | 136.8 | 122.4 | 129.6 | 121.5 | 119.4 | 128.8 | 155.5 |
| 4 | 134.2 | 134 | 111.4 | 115.5 | 114.4 | 123.7 | 121.4 | 107.4 | 103.3 | 118.7 | 126.2 |
| 6 | 122.8 | 120.4 | 102.7 | 114.3 | 105 | 110.1 | 108.8 | 104.9 | 102.1 | 111.1 | 115.4 |
| 8 | 105.4 | 116 | 99.39 | 100.2 | 96.19 | 102.6 | 109.5 | 100.8 | 101.4 | 106.9 | 106 |
| 10 | 101.8 | 99.09 | 97.74 | 100.9 | 86.59 | 100.1 | 98 | 101.4 | 104.1 | 101.5 | 99.6 |
| 12 | 98.21 | 100.9 | 102.3 | 99.06 | 113.4 | 99.94 | 102 | 98.63 | 95.87 | 98.53 | 100.4 |
| 14 | 131.5 | 94.23 | 86.03 | 85.12 | 70.15 | 93.65 | 97.4 | 63.51 | 112.5 | 115 | 122.3 |
| 16 | 108.8 | 117.4 | 128.2 | 116.3 | 129.6 | 136.8 | 123.6 | 82.31 | 114.1 | 105.3 | 124.5 |
| 18 | 128 | 126.9 | 139 | 133.7 | 95.82 | 129.5 | 85.78 | 129.6 | 107.6 | 127.3 | 140.7 |
| 20 | 127.2 | 128.8 | 126.9 | 125.7 | 101.4 | 128.5 | 123.3 | 100.9 | 143.7 | 127.5 | 139 |
| 22 | 97.6 | 118.6 | 126 | 132.7 | 96.89 | 113.1 | 114.9 | 125.9 | 105.5 | 117.7 | 189.7 |
| 24 | 122.5 | 129.6 | 124.3 | 115.6 | 76.45 | 112.5 | 104.1 | 98.95 | 103.4 | 119 | 166.9 |

| **TRF** | | | | | | | | | | | | | |
|---|---|---|---|---|---|---|---|---|---|---|---|---|---|
| ZT | | | | | | | | | | | | | |
| 2 | 161 | 170.8 | 145.9 | 144.7 | 144.2 | 150.6 | 143.2 | 129.8 | 144.9 | 120.6 | 155.4 | 110.3 | 148.3 |

| | | | | | | | | | | | | | |
|---|---|---|---|---|---|---|---|---|---|---|---|---|---|
| 4 | 124.8 | 141 | 123.4 | 130.5 | 124 | 123.6 | 117 | 116.2 | 123.6 | 121.7 | 116.3 | 96.73 | 123.2 |
| 6 | 124 | 131.2 | 116 | 114.6 | 110.4 | 105.4 | 103.4 | 108.9 | 114.5 | 108.6 | 111.9 | 96.51 | 115.9 |
| 8 | 107.3 | 118.2 | 105.7 | 110.1 | 98.44 | 110.8 | 96.3 | 106.1 | 101.7 | 112.1 | 104.4 | 101.5 | 110.4 |
| 10 | 104.5 | 103 | 102.5 | 100.3 | 105.5 | 102.6 | 103 | 99.89 | 106.6 | 103.3 | 97.75 | 102.7 | 98.61 |
| 12 | 95.51 | 96.96 | 97.48 | 99.68 | 94.54 | 97.44 | 96.98 | 100.1 | 93.44 | 96.69 | 102.3 | 97.33 | 101.4 |
| 14 | 129.5 | 89.13 | 61.33 | 99.67 | 73.76 | 82.38 | 81.95 | 100.4 | 70.55 | 110.3 | 116 | 68.11 | 83.84 |
| 16 | 148.4 | 115.7 | 125.6 | 109.2 | 140.4 | 120 | 124.8 | 121.1 | 111.7 | 111.7 | 113.3 | 82.99 | 174.1 |
| 18 | 134 | 132.5 | 117.2 | 145.1 | 77.29 | 131.3 | 129.2 | 23.77 | *9999* | 129.7 | 120.9 | 97.64 | 71.72 |
| 20 | 126.7 | 126.6 | 138.4 | 39.68 | 181.6 | 26.32 | 131.5 | 157.2 | 83.51 | 113.1 | 129.9 | 139.5 | 198.9 |
| 22 | 154.9 | *9999* | 131.9 | 164 | 163 | 147.8 | 128.6 | 131.5 | 135.2 | 136.1 | 119.6 | 128 | 158.6 |
| 24 | 133 | 182.2 | 114.6 | 141 | 147.7 | 138 | 109.2 | 119 | 129.8 | 102.6 | 58.85 | 108.3 | 192.1 |

| **TRM** | | | | | | | | | | | | | | | |
|---|---|---|---|---|---|---|---|---|---|---|---|---|---|---|---|
| Hz | | | | | | | | | | | | | | | |
| 2 | 135.6 | 114.3 | 136.9 | 176.2 | 98.26 | 137.1 | 143.2 | 133.2 | 127.2 | 166.8 | 173.4 | 137.4 | 138 | 124.2 | 100.2 |
| 4 | 113.5 | 104 | 117 | 131.9 | 120 | 124.5 | 121.2 | 140.3 | 153.4 | 122.4 | 161.2 | 112 | 121.5 | 115.2 | 107 |
| 6 | 107 | 103.8 | 104.4 | 119.2 | 119.5 | 131.2 | 108.3 | 117.2 | 155.4 | 110.4 | 90.23 | 104.6 | 116 | 117.1 | 111.1 |
| 8 | 107.6 | 105.4 | 101.2 | 109.7 | 168.3 | 114.2 | 100.7 | 111 | 106.4 | 99.43 | 90.69 | 97.24 | 106.2 | 104.2 | 104.8 |
| 10 | 99.22 | 103.2 | 97.7 | 99.26 | 78.46 | 102.1 | 96.3 | 99.86 | 97.24 | 106.9 | 77.16 | 103.5 | 103.7 | 102.1 | 98.91 |
| 12 | 100.8 | 96.85 | 102.3 | 100.7 | 121.6 | 97.87 | 103.7 | 100.1 | 102.8 | 93.08 | 145.7 | 96.46 | 96.27 | 97.88 | 101.1 |
| 14 | 138.3 | 101.6 | 103.7 | 119.5 | 137.4 | 114.3 | 108.4 | 144.1 | 126.9 | 70.59 | 285 | 71.59 | 137.4 | 104.1 | 92.26 |
| 16 | 118.3 | 119.2 | 120.4 | 128.7 | 135.4 | 105 | 138.9 | 131.6 | 137.2 | 119.4 | 291.1 | 141.2 | 119.2 | 109.4 | 95.74 |
| 18 | 133.1 | 108.2 | 126.8 | 115.2 | 83.06 | 145.5 | 135.5 | 120.7 | 117.3 | 128.5 | 307.3 | 145 | 78.6 | 108.2 | 117 |
| 20 | 127.5 | 125.7 | 136.3 | 139.1 | 90.51 | 165.7 | 142.1 | 121.3 | 133.2 | 125.1 | 574.5 | 131.6 | 148.5 | 113.3 | 105.4 |
| 22 | 111.5 | 112.5 | 115.8 | 119.1 | 91.3 | 153.2 | 119.3 | 110.4 | 109 | 101.9 | 503.6 | 125.2 | 136.5 | 108.6 | 115.7 |
| 24 | 122.9 | 111.2 | 116.1 | 109.8 | 112.2 | 124.5 | 123.4 | 99.93 | 112.6 | 99.76 | 1.89 | 100.2 | 119.1 | 103.8 | 94.94 |

*9999* represents a missing data point

Electroencephalogram analysis after 6 h of sleep deprivation

Electroencephalographic power spectral density during NREMS after 6 h of sleep deprivation

| **CLF** | | | | | | | | | | | | |
|---|---|---|---|---|---|---|---|---|---|---|---|---|
| Hz | | | | | | | | | | | | |
| 0.25 | 2.95 | 1.66 | 3.35 | 1.33 | 2.02 | 1.67 | 2.52 | 4.1 | 1.51 | 2.47 | 1.19 | 1.29 |
| 0.5 | 2.55 | 2.46 | 2.83 | 1.98 | 2.26 | 2.73 | 4.84 | 2 | 2.36 | 1.98 | 2.59 | 1.99 |
| 0.75 | 3.62 | 3.44 | 3.53 | 2.73 | 3.13 | 3.82 | 5.05 | 2.68 | 3.7 | 2.65 | 3.9 | 3.02 |
| 1 | 4.02 | 4.13 | 4.02 | 3.16 | 3.2 | 4.35 | 4.78 | 3.27 | 4.32 | 3.06 | 4.22 | 3.11 |
| 1.25 | 4.09 | 4.36 | 4.21 | 3.43 | 3.19 | 4.19 | 4.63 | 3.37 | 4.29 | 3.17 | 4.11 | 3.3 |
| 1.5 | 4 | 4.6 | 4.13 | 3.47 | 3.07 | 4.08 | 4.56 | 3.32 | 4.3 | 3.17 | 3.74 | 3.26 |
| 1.75 | 3.69 | 4.51 | 3.99 | 3.54 | 3.06 | 3.84 | 4.53 | 3.23 | 4.01 | 3.14 | 3.47 | 3.28 |
| 2 | 3.57 | 4.29 | 3.79 | 3.51 | 2.87 | 3.67 | 4.36 | 3.16 | 3.87 | 3.03 | 3.27 | 3.2 |
| 2.25 | 3.28 | 4.17 | 3.59 | 3.45 | 2.83 | 3.36 | 4.31 | 3.04 | 3.61 | 3.06 | 3.09 | 3.22 |
| 2.5 | 3.07 | 3.94 | 3.34 | 3.32 | 2.85 | 3.22 | 4.09 | 2.96 | 3.4 | 2.92 | 2.99 | 3.19 |

| | | | | | | | | | | | | |
|---|---|---|---|---|---|---|---|---|---|---|---|---|
| 2.75 | 2.97 | 3.8 | 3.07 | 3.21 | 2.88 | 3.14 | 3.87 | 2.88 | 3.18 | 2.91 | 3.01 | 3.32 |
| 3 | 2.87 | 3.55 | 3.07 | 3.13 | 2.98 | 2.92 | 3.61 | 2.75 | 3.06 | 2.89 | 2.95 | 3.09 |
| 3.25 | 2.7 | 3.57 | 2.94 | 3.06 | 3.01 | 2.74 | 3.36 | 2.56 | 2.79 | 2.89 | 2.88 | 2.97 |
| 3.5 | 2.59 | 3.3 | 2.66 | 2.95 | 3.16 | 2.5 | 3.23 | 2.43 | 2.55 | 2.8 | 2.62 | 2.86 |
| 3.75 | 2.36 | 2.88 | 2.4 | 2.68 | 2.92 | 2.41 | 2.96 | 2.43 | 2.4 | 2.59 | 2.48 | 2.68 |
| 4 | 2.21 | 2.52 | 2.19 | 2.56 | 2.64 | 2.2 | 2.78 | 2.32 | 2.2 | 2.4 | 2.23 | 2.53 |
| 4.25 | 2.06 | 2.2 | 1.97 | 2.41 | 2.33 | 2.01 | 2.65 | 2.15 | 2.05 | 2.17 | 2.12 | 2.43 |
| 4.5 | 1.96 | 1.97 | 1.83 | 2.28 | 2.1 | 1.98 | 2.39 | 2.11 | 1.9 | 2.03 | 1.98 | 2.29 |
| 4.75 | 1.84 | 1.78 | 1.74 | 2.14 | 1.97 | 1.87 | 2.31 | 2.02 | 1.82 | 1.87 | 1.84 | 2.19 |
| 5 | 1.8 | 1.64 | 1.64 | 2.03 | 1.88 | 1.79 | 2.06 | 1.92 | 1.7 | 1.8 | 1.75 | 1.98 |
| 5.25 | 1.71 | 1.54 | 1.55 | 1.93 | 1.74 | 1.71 | 1.87 | 1.82 | 1.66 | 1.7 | 1.62 | 1.87 |
| 5.5 | 1.62 | 1.49 | 1.49 | 1.85 | 1.66 | 1.67 | 1.7 | 1.78 | 1.57 | 1.62 | 1.56 | 1.77 |
| 5.75 | 1.56 | 1.45 | 1.45 | 1.8 | 1.62 | 1.62 | 1.57 | 1.79 | 1.51 | 1.56 | 1.49 | 1.76 |
| 6 | 1.49 | 1.4 | 1.43 | 1.74 | 1.58 | 1.56 | 1.4 | 1.74 | 1.5 | 1.55 | 1.47 | 1.67 |
| 6.25 | 1.51 | 1.43 | 1.38 | 1.71 | 1.55 | 1.6 | 1.27 | 1.69 | 1.46 | 1.48 | 1.46 | 1.65 |
| 6.5 | 1.49 | 1.4 | 1.4 | 1.67 | 1.52 | 1.64 | 1.13 | 1.68 | 1.46 | 1.52 | 1.49 | 1.58 |
| 6.75 | 1.46 | 1.41 | 1.41 | 1.67 | 1.57 | 1.6 | 1.02 | 1.68 | 1.42 | 1.47 | 1.51 | 1.56 |
| 7 | 1.47 | 1.37 | 1.34 | 1.61 | 1.54 | 1.53 | 0.92 | 1.67 | 1.4 | 1.47 | 1.49 | 1.58 |
| 7.25 | 1.43 | 1.3 | 1.3 | 1.53 | 1.55 | 1.48 | 0.84 | 1.58 | 1.36 | 1.38 | 1.48 | 1.49 |
| 7.5 | 1.36 | 1.25 | 1.31 | 1.5 | 1.55 | 1.41 | 0.75 | 1.55 | 1.32 | 1.36 | 1.47 | 1.49 |
| 7.75 | 1.34 | 1.18 | 1.19 | 1.44 | 1.51 | 1.34 | 0.69 | 1.46 | 1.27 | 1.29 | 1.4 | 1.41 |
| 8 | 1.26 | 1.11 | 1.15 | 1.38 | 1.44 | 1.29 | 0.65 | 1.43 | 1.2 | 1.21 | 1.35 | 1.35 |
| 8.25 | 1.21 | 1.05 | 1.04 | 1.28 | 1.38 | 1.16 | 0.58 | 1.37 | 1.1 | 1.15 | 1.31 | 1.29 |
| 8.5 | 1.14 | 1.03 | 1.04 | 1.21 | 1.35 | 1.11 | 0.54 | 1.33 | 1.05 | 1.08 | 1.24 | 1.22 |
| 8.75 | 1.08 | 0.95 | 0.96 | 1.11 | 1.28 | 1.02 | 0.48 | 1.21 | 0.96 | 1.02 | 1.16 | 1.19 |
| 9 | 1.05 | 0.86 | 0.95 | 1.03 | 1.2 | 0.96 | 0.46 | 1.16 | 0.92 | 0.99 | 1.11 | 1.12 |
| 9.25 | 1 | 0.82 | 0.9 | 1.01 | 1.14 | 0.91 | 0.41 | 1.09 | 0.85 | 0.93 | 1.08 | 1.03 |
| 9.5 | 0.91 | 0.79 | 0.85 | 0.96 | 1.08 | 0.86 | 0.39 | 1.04 | 0.78 | 0.89 | 0.99 | 0.98 |
| 9.75 | 0.85 | 0.73 | 0.8 | 0.86 | 1.02 | 0.8 | 0.37 | 0.97 | 0.74 | 0.85 | 0.93 | 0.92 |
| 10 | 0.83 | 0.66 | 0.77 | 0.83 | 0.97 | 0.75 | 0.36 | 0.9 | 0.7 | 0.8 | 0.89 | 0.87 |
| 10.25 | 0.76 | 0.63 | 0.71 | 0.78 | 0.92 | 0.7 | 0.33 | 0.85 | 0.65 | 0.77 | 0.82 | 0.81 |
| 10.5 | 0.74 | 0.58 | 0.66 | 0.72 | 0.86 | 0.67 | 0.31 | 0.78 | 0.59 | 0.71 | 0.78 | 0.76 |
| 10.75 | 0.69 | 0.54 | 0.63 | 0.68 | 0.83 | 0.62 | 0.29 | 0.74 | 0.58 | 0.7 | 0.74 | 0.75 |
| 11 | 0.66 | 0.51 | 0.6 | 0.63 | 0.78 | 0.6 | 0.27 | 0.67 | 0.51 | 0.66 | 0.69 | 0.69 |
| 11.25 | 0.61 | 0.47 | 0.57 | 0.6 | 0.74 | 0.56 | 0.26 | 0.63 | 0.48 | 0.63 | 0.66 | 0.65 |
| 11.5 | 0.58 | 0.45 | 0.53 | 0.56 | 0.69 | 0.55 | 0.25 | 0.59 | 0.48 | 0.6 | 0.63 | 0.62 |
| 11.75 | 0.54 | 0.41 | 0.5 | 0.53 | 0.66 | 0.53 | 0.23 | 0.56 | 0.43 | 0.57 | 0.58 | 0.57 |
| 12 | 0.52 | 0.39 | 0.5 | 0.51 | 0.61 | 0.5 | 0.22 | 0.52 | 0.42 | 0.53 | 0.56 | 0.54 |
| 12.25 | 0.5 | 0.37 | 0.47 | 0.48 | 0.57 | 0.48 | 0.21 | 0.51 | 0.4 | 0.51 | 0.53 | 0.52 |
| 12.5 | 0.45 | 0.35 | 0.45 | 0.45 | 0.54 | 0.46 | 0.2 | 0.48 | 0.38 | 0.48 | 0.49 | 0.51 |
| 12.75 | 0.44 | 0.33 | 0.42 | 0.43 | 0.49 | 0.43 | 0.19 | 0.44 | 0.36 | 0.46 | 0.47 | 0.48 |
| 13 | 0.41 | 0.31 | 0.4 | 0.4 | 0.47 | 0.41 | 0.18 | 0.42 | 0.34 | 0.43 | 0.46 | 0.45 |
| 13.25 | 0.39 | 0.3 | 0.38 | 0.38 | 0.44 | 0.41 | 0.18 | 0.4 | 0.33 | 0.41 | 0.42 | 0.44 |
| 13.5 | 0.38 | 0.28 | 0.38 | 0.36 | 0.41 | 0.39 | 0.17 | 0.38 | 0.31 | 0.39 | 0.41 | 0.41 |
| 13.75 | 0.36 | 0.26 | 0.35 | 0.34 | 0.4 | 0.37 | 0.17 | 0.36 | 0.31 | 0.37 | 0.39 | 0.39 |
| 14 | 0.34 | 0.24 | 0.34 | 0.32 | 0.37 | 0.35 | 0.16 | 0.34 | 0.29 | 0.35 | 0.36 | 0.37 |
| 14.25 | 0.31 | 0.23 | 0.33 | 0.31 | 0.36 | 0.33 | 0.16 | 0.34 | 0.28 | 0.33 | 0.35 | 0.35 |
| 14.5 | 0.3 | 0.23 | 0.32 | 0.29 | 0.33 | 0.32 | 0.15 | 0.31 | 0.27 | 0.32 | 0.33 | 0.33 |

| | | | | | | | | | | | | |
|---|---|---|---|---|---|---|---|---|---|---|---|---|
| 14.75 | 0.28 | 0.21 | 0.3 | 0.28 | 0.31 | 0.3 | 0.15 | 0.3 | 0.26 | 0.31 | 0.31 | 0.31 |
| 15 | 0.27 | 0.2 | 0.29 | 0.26 | 0.28 | 0.29 | 0.14 | 0.28 | 0.25 | 0.28 | 0.3 | 0.3 |
| 15.25 | 0.26 | 0.19 | 0.28 | 0.25 | 0.27 | 0.27 | 0.14 | 0.26 | 0.23 | 0.28 | 0.28 | 0.29 |
| 15.5 | 0.25 | 0.18 | 0.26 | 0.24 | 0.26 | 0.26 | 0.14 | 0.25 | 0.22 | 0.26 | 0.26 | 0.27 |
| 15.75 | 0.24 | 0.17 | 0.25 | 0.23 | 0.24 | 0.24 | 0.13 | 0.24 | 0.22 | 0.25 | 0.25 | 0.25 |
| 16 | 0.22 | 0.16 | 0.24 | 0.21 | 0.23 | 0.23 | 0.13 | 0.23 | 0.21 | 0.23 | 0.24 | 0.24 |
| 16.25 | 0.21 | 0.16 | 0.22 | 0.2 | 0.21 | 0.22 | 0.12 | 0.22 | 0.2 | 0.22 | 0.23 | 0.22 |
| 16.5 | 0.2 | 0.15 | 0.21 | 0.19 | 0.2 | 0.21 | 0.12 | 0.2 | 0.19 | 0.21 | 0.22 | 0.21 |
| 16.75 | 0.19 | 0.14 | 0.2 | 0.18 | 0.19 | 0.19 | 0.12 | 0.19 | 0.18 | 0.2 | 0.2 | 0.2 |
| 17 | 0.18 | 0.13 | 0.19 | 0.17 | 0.18 | 0.18 | 0.12 | 0.18 | 0.17 | 0.2 | 0.2 | 0.19 |
| 17.25 | 0.17 | 0.13 | 0.18 | 0.16 | 0.17 | 0.17 | 0.12 | 0.18 | 0.17 | 0.19 | 0.18 | 0.18 |
| 17.5 | 0.17 | 0.12 | 0.18 | 0.15 | 0.16 | 0.17 | 0.11 | 0.17 | 0.16 | 0.18 | 0.17 | 0.17 |
| 17.75 | 0.15 | 0.12 | 0.17 | 0.15 | 0.16 | 0.16 | 0.11 | 0.16 | 0.15 | 0.17 | 0.17 | 0.17 |
| 18 | 0.15 | 0.11 | 0.16 | 0.14 | 0.15 | 0.15 | 0.1 | 0.15 | 0.14 | 0.17 | 0.16 | 0.15 |
| 18.25 | 0.14 | 0.11 | 0.16 | 0.14 | 0.14 | 0.14 | 0.1 | 0.15 | 0.14 | 0.16 | 0.15 | 0.15 |
| 18.5 | 0.14 | 0.1 | 0.15 | 0.13 | 0.13 | 0.13 | 0.1 | 0.14 | 0.13 | 0.15 | 0.14 | 0.14 |
| 18.75 | 0.13 | 0.1 | 0.15 | 0.12 | 0.13 | 0.13 | 0.1 | 0.13 | 0.13 | 0.15 | 0.14 | 0.14 |
| 19 | 0.12 | 0.09 | 0.14 | 0.12 | 0.12 | 0.12 | 0.1 | 0.13 | 0.12 | 0.14 | 0.13 | 0.13 |
| 19.25 | 0.12 | 0.09 | 0.13 | 0.11 | 0.12 | 0.11 | 0.09 | 0.12 | 0.12 | 0.14 | 0.13 | 0.12 |
| 19.5 | 0.11 | 0.09 | 0.13 | 0.1 | 0.11 | 0.11 | 0.09 | 0.12 | 0.11 | 0.13 | 0.12 | 0.12 |
| 19.75 | 0.11 | 0.08 | 0.12 | 0.1 | 0.11 | 0.1 | 0.09 | 0.11 | 0.11 | 0.13 | 0.11 | 0.12 |
| 20 | 0.1 | 0.08 | 0.12 | 0.1 | 0.1 | 0.1 | 0.09 | 0.11 | 0.1 | 0.12 | 0.11 | 0.11 |

| **CLM** | | | | | | | | | | | |
|---|---|---|---|---|---|---|---|---|---|---|---|
| Hz | | | | | | | | | | | |
| 0.25 | 1.47 | 3.01 | 1.16 | 0.79 | 1.58 | 1.13 | 3.54 | 5.2 | 2.11 | 0.97 | 2.46 |
| 0.5 | 2.15 | 2.88 | 2.31 | 1.81 | 2.19 | 2.09 | 2.59 | 3.24 | 2.99 | 1.92 | 2.31 |
| 0.75 | 2.92 | 3.84 | 3.14 | 2.74 | 2.52 | 2.82 | 2.83 | 3.31 | 3.76 | 2.66 | 2.98 |
| 1 | 3.49 | 4.12 | 3.59 | 3.41 | 2.51 | 3.24 | 2.75 | 3.33 | 4.05 | 2.98 | 3.16 |
| 1.25 | 3.56 | 4.06 | 3.69 | 3.63 | 2.56 | 3.22 | 2.71 | 3.12 | 4 | 2.98 | 3.29 |
| 1.5 | 3.59 | 4.03 | 3.72 | 3.78 | 2.66 | 3.16 | 2.53 | 2.92 | 3.86 | 2.94 | 3.14 |
| 1.75 | 3.59 | 3.84 | 3.69 | 3.9 | 2.76 | 3.19 | 2.58 | 2.77 | 3.61 | 2.96 | 3.09 |
| 2 | 3.45 | 3.77 | 3.45 | 3.77 | 2.94 | 3.16 | 2.56 | 2.63 | 3.4 | 2.92 | 3.04 |
| 2.25 | 3.31 | 3.53 | 3.22 | 3.76 | 3.18 | 3.15 | 2.62 | 2.53 | 3.12 | 2.87 | 3.02 |
| 2.5 | 3.22 | 3.45 | 3.18 | 3.68 | 3.57 | 3.15 | 2.74 | 2.53 | 2.97 | 2.87 | 2.94 |
| 2.75 | 3.11 | 3.29 | 3.09 | 3.55 | 3.87 | 3.04 | 2.8 | 2.54 | 2.84 | 2.86 | 2.96 |
| 3 | 2.97 | 3.16 | 2.95 | 3.46 | 3.91 | 2.94 | 2.67 | 2.5 | 2.83 | 2.91 | 2.83 |
| 3.25 | 2.92 | 3.1 | 2.92 | 3.33 | 3.82 | 3.02 | 2.57 | 2.38 | 2.88 | 2.9 | 2.8 |
| 3.5 | 2.76 | 2.94 | 2.76 | 3.11 | 3.39 | 2.87 | 2.55 | 2.38 | 2.96 | 2.89 | 2.72 |
| 3.75 | 2.64 | 2.68 | 2.56 | 2.9 | 3.07 | 2.78 | 2.47 | 2.19 | 2.86 | 2.83 | 2.54 |
| 4 | 2.55 | 2.34 | 2.3 | 2.67 | 2.73 | 2.6 | 2.33 | 2.1 | 2.66 | 2.77 | 2.44 |
| 4.25 | 2.37 | 2.15 | 2.23 | 2.51 | 2.4 | 2.41 | 2.27 | 2 | 2.39 | 2.67 | 2.34 |
| 4.5 | 2.27 | 1.95 | 2.1 | 2.35 | 2.15 | 2.29 | 2.15 | 1.92 | 2.21 | 2.49 | 2.18 |
| 4.75 | 2.17 | 1.84 | 2.01 | 2.2 | 1.99 | 2.13 | 2.14 | 1.83 | 2.03 | 2.32 | 2.1 |
| 5 | 2.07 | 1.74 | 1.97 | 2.05 | 1.9 | 2 | 2 | 1.79 | 1.93 | 2.18 | 2.03 |
| 5.25 | 1.98 | 1.61 | 1.94 | 1.99 | 1.8 | 1.96 | 2 | 1.69 | 1.83 | 2.14 | 1.95 |
| 5.5 | 1.9 | 1.52 | 1.82 | 1.93 | 1.77 | 1.84 | 1.94 | 1.65 | 1.75 | 2.07 | 1.89 |

| | | | | | | | | | | | |
|---|---|---|---|---|---|---|---|---|---|---|---|
| 5.75 | 1.8 | 1.46 | 1.79 | 1.92 | 1.77 | 1.84 | 1.92 | 1.66 | 1.68 | 1.98 | 1.81 |
| 6 | 1.77 | 1.39 | 1.8 | 1.89 | 1.73 | 1.76 | 1.85 | 1.67 | 1.66 | 1.95 | 1.78 |
| 6.25 | 1.71 | 1.39 | 1.71 | 1.89 | 1.73 | 1.75 | 1.8 | 1.69 | 1.58 | 1.94 | 1.81 |
| 6.5 | 1.7 | 1.37 | 1.75 | 1.93 | 1.73 | 1.73 | 1.79 | 1.68 | 1.59 | 1.91 | 1.75 |
| 6.75 | 1.63 | 1.38 | 1.65 | 1.84 | 1.71 | 1.71 | 1.77 | 1.68 | 1.51 | 1.8 | 1.71 |
| 7 | 1.64 | 1.35 | 1.65 | 1.73 | 1.68 | 1.7 | 1.64 | 1.64 | 1.45 | 1.81 | 1.67 |
| 7.25 | 1.54 | 1.3 | 1.57 | 1.63 | 1.66 | 1.58 | 1.6 | 1.58 | 1.39 | 1.77 | 1.64 |
| 7.5 | 1.51 | 1.25 | 1.5 | 1.53 | 1.58 | 1.5 | 1.57 | 1.55 | 1.32 | 1.69 | 1.59 |
| 7.75 | 1.4 | 1.17 | 1.43 | 1.41 | 1.5 | 1.44 | 1.5 | 1.5 | 1.21 | 1.63 | 1.49 |
| 8 | 1.31 | 1.1 | 1.31 | 1.28 | 1.46 | 1.39 | 1.44 | 1.4 | 1.17 | 1.55 | 1.46 |
| 8.25 | 1.24 | 1.03 | 1.28 | 1.19 | 1.35 | 1.3 | 1.39 | 1.33 | 1.05 | 1.41 | 1.39 |
| 8.5 | 1.14 | 0.99 | 1.2 | 1.1 | 1.29 | 1.23 | 1.31 | 1.25 | 1 | 1.32 | 1.3 |
| 8.75 | 1.07 | 0.94 | 1.17 | 1.02 | 1.2 | 1.15 | 1.21 | 1.17 | 0.94 | 1.24 | 1.22 |
| 9 | 0.98 | 0.88 | 1.08 | 0.92 | 1.12 | 1.08 | 1.15 | 1.12 | 0.88 | 1.18 | 1.13 |
| 9.25 | 0.94 | 0.84 | 1 | 0.84 | 1.07 | 1.05 | 1.11 | 1.05 | 0.82 | 1.1 | 1.07 |
| 9.5 | 0.88 | 0.78 | 0.96 | 0.76 | 0.98 | 0.97 | 1.08 | 0.99 | 0.79 | 0.99 | 1.01 |
| 9.75 | 0.84 | 0.72 | 0.88 | 0.7 | 0.92 | 0.91 | 1.01 | 0.91 | 0.73 | 0.95 | 0.93 |
| 10 | 0.76 | 0.69 | 0.83 | 0.64 | 0.87 | 0.86 | 0.98 | 0.89 | 0.69 | 0.87 | 0.9 |
| 10.25 | 0.73 | 0.64 | 0.77 | 0.6 | 0.77 | 0.8 | 0.9 | 0.82 | 0.67 | 0.82 | 0.83 |
| 10.5 | 0.7 | 0.61 | 0.72 | 0.55 | 0.73 | 0.78 | 0.86 | 0.78 | 0.63 | 0.76 | 0.76 |
| 10.75 | 0.66 | 0.58 | 0.69 | 0.51 | 0.68 | 0.73 | 0.83 | 0.75 | 0.59 | 0.71 | 0.73 |
| 11 | 0.62 | 0.55 | 0.65 | 0.47 | 0.61 | 0.69 | 0.77 | 0.7 | 0.56 | 0.66 | 0.67 |
| 11.25 | 0.58 | 0.52 | 0.61 | 0.45 | 0.57 | 0.65 | 0.73 | 0.67 | 0.52 | 0.62 | 0.64 |
| 11.5 | 0.55 | 0.5 | 0.57 | 0.42 | 0.56 | 0.62 | 0.69 | 0.63 | 0.5 | 0.59 | 0.62 |
| 11.75 | 0.53 | 0.47 | 0.53 | 0.4 | 0.51 | 0.57 | 0.64 | 0.59 | 0.47 | 0.55 | 0.56 |
| 12 | 0.5 | 0.44 | 0.5 | 0.37 | 0.48 | 0.55 | 0.6 | 0.55 | 0.45 | 0.52 | 0.53 |
| 12.25 | 0.48 | 0.42 | 0.49 | 0.35 | 0.44 | 0.53 | 0.57 | 0.52 | 0.43 | 0.49 | 0.5 |
| 12.5 | 0.45 | 0.4 | 0.46 | 0.33 | 0.41 | 0.5 | 0.54 | 0.51 | 0.41 | 0.45 | 0.46 |
| 12.75 | 0.41 | 0.38 | 0.44 | 0.31 | 0.39 | 0.48 | 0.5 | 0.47 | 0.39 | 0.43 | 0.44 |
| 13 | 0.4 | 0.37 | 0.41 | 0.3 | 0.36 | 0.46 | 0.48 | 0.45 | 0.37 | 0.4 | 0.43 |
| 13.25 | 0.39 | 0.36 | 0.4 | 0.28 | 0.34 | 0.44 | 0.45 | 0.42 | 0.36 | 0.39 | 0.4 |
| 13.5 | 0.37 | 0.34 | 0.37 | 0.26 | 0.32 | 0.41 | 0.42 | 0.4 | 0.34 | 0.36 | 0.38 |
| 13.75 | 0.35 | 0.32 | 0.35 | 0.25 | 0.3 | 0.39 | 0.4 | 0.38 | 0.33 | 0.34 | 0.35 |
| 14 | 0.33 | 0.32 | 0.34 | 0.24 | 0.29 | 0.36 | 0.39 | 0.35 | 0.31 | 0.33 | 0.33 |
| 14.25 | 0.32 | 0.3 | 0.33 | 0.23 | 0.27 | 0.35 | 0.36 | 0.35 | 0.3 | 0.31 | 0.31 |
| 14.5 | 0.3 | 0.29 | 0.31 | 0.22 | 0.25 | 0.33 | 0.34 | 0.33 | 0.3 | 0.29 | 0.31 |
| 14.75 | 0.29 | 0.28 | 0.29 | 0.21 | 0.24 | 0.3 | 0.32 | 0.32 | 0.27 | 0.27 | 0.28 |
| 15 | 0.27 | 0.26 | 0.27 | 0.2 | 0.22 | 0.29 | 0.31 | 0.29 | 0.27 | 0.26 | 0.27 |
| 15.25 | 0.27 | 0.25 | 0.26 | 0.19 | 0.22 | 0.27 | 0.28 | 0.28 | 0.26 | 0.25 | 0.26 |
| 15.5 | 0.25 | 0.23 | 0.25 | 0.18 | 0.2 | 0.26 | 0.27 | 0.26 | 0.24 | 0.24 | 0.24 |
| 15.75 | 0.24 | 0.22 | 0.23 | 0.17 | 0.19 | 0.25 | 0.26 | 0.26 | 0.23 | 0.22 | 0.22 |
| 16 | 0.23 | 0.22 | 0.22 | 0.17 | 0.18 | 0.23 | 0.24 | 0.25 | 0.22 | 0.21 | 0.22 |
| 16.25 | 0.21 | 0.2 | 0.21 | 0.16 | 0.18 | 0.22 | 0.23 | 0.23 | 0.21 | 0.2 | 0.21 |
| 16.5 | 0.21 | 0.2 | 0.2 | 0.15 | 0.16 | 0.21 | 0.23 | 0.22 | 0.2 | 0.2 | 0.2 |
| 16.75 | 0.19 | 0.18 | 0.19 | 0.14 | 0.16 | 0.2 | 0.21 | 0.21 | 0.19 | 0.18 | 0.18 |
| 17 | 0.18 | 0.18 | 0.18 | 0.14 | 0.15 | 0.19 | 0.2 | 0.2 | 0.18 | 0.17 | 0.17 |
| 17.25 | 0.17 | 0.17 | 0.17 | 0.14 | 0.15 | 0.18 | 0.19 | 0.2 | 0.18 | 0.16 | 0.17 |
| 17.5 | 0.17 | 0.16 | 0.16 | 0.13 | 0.14 | 0.17 | 0.18 | 0.18 | 0.17 | 0.15 | 0.16 |

| | | | | | | | | | | | |
|---|---|---|---|---|---|---|---|---|---|---|---|
| 17.75 | 0.16 | 0.16 | 0.15 | 0.12 | 0.13 | 0.16 | 0.18 | 0.18 | 0.16 | 0.15 | 0.15 |
| 18 | 0.15 | 0.15 | 0.15 | 0.12 | 0.13 | 0.15 | 0.16 | 0.17 | 0.15 | 0.15 | 0.15 |
| 18.25 | 0.14 | 0.14 | 0.14 | 0.11 | 0.12 | 0.15 | 0.16 | 0.16 | 0.15 | 0.14 | 0.14 |
| 18.5 | 0.14 | 0.14 | 0.14 | 0.11 | 0.12 | 0.14 | 0.15 | 0.15 | 0.15 | 0.13 | 0.14 |
| 18.75 | 0.13 | 0.13 | 0.13 | 0.1 | 0.12 | 0.13 | 0.15 | 0.15 | 0.14 | 0.13 | 0.13 |
| 19 | 0.12 | 0.13 | 0.12 | 0.1 | 0.11 | 0.13 | 0.14 | 0.14 | 0.13 | 0.12 | 0.12 |
| 19.25 | 0.12 | 0.12 | 0.12 | 0.1 | 0.11 | 0.12 | 0.14 | 0.14 | 0.12 | 0.12 | 0.12 |
| 19.5 | 0.11 | 0.11 | 0.11 | 0.09 | 0.11 | 0.12 | 0.13 | 0.13 | 0.12 | 0.11 | 0.12 |
| 19.75 | 0.11 | 0.11 | 0.1 | 0.09 | 0.1 | 0.11 | 0.12 | 0.13 | 0.12 | 0.1 | 0.11 |
| 20 | 0.1 | 0.11 | 0.1 | 0.08 | 0.1 | 0.11 | 0.12 | 0.12 | 0.11 | 0.1 | 0.1 |

| **TRF** | | | | | | | | | | | | | |
|---|---|---|---|---|---|---|---|---|---|---|---|---|---|
| Hz | | | | | | | | | | | | | |
| 0.25 | 1.62 | 0.91 | 1.48 | 1.18 | 1.71 | 5.39 | 1.5 | 3.08 | 4.63 | 6.29 | 2.4 | 2.86 | 1.07 |
| 0.5 | 2.23 | 1.73 | 2.67 | 2.39 | 2.62 | 2.68 | 2.45 | 2.7 | 3.69 | 2.56 | 2.25 | 2.07 | 1.71 |
| 0.75 | 2.92 | 2.34 | 3.55 | 3.48 | 3.86 | 3.09 | 3.43 | 3.58 | 3.48 | 2.88 | 2.78 | 2.91 | 2.4 |
| 1 | 3.23 | 2.57 | 4.03 | 3.99 | 4.35 | 3.51 | 3.82 | 4.1 | 3.52 | 3.36 | 3.23 | 3.41 | 2.8 |
| 1.25 | 3.33 | 2.64 | 4.04 | 4.13 | 4.44 | 3.67 | 3.86 | 4.02 | 3.44 | 3.53 | 3.39 | 3.62 | 3.24 |
| 1.5 | 3.3 | 2.79 | 4.06 | 4.1 | 4.34 | 3.7 | 3.84 | 4 | 3.39 | 3.48 | 3.37 | 3.73 | 3.41 |
| 1.75 | 3.26 | 2.99 | 3.82 | 3.9 | 4.14 | 3.6 | 3.55 | 3.78 | 3.39 | 3.33 | 3.21 | 3.59 | 3.64 |
| 2 | 3.3 | 3.13 | 3.61 | 3.77 | 3.95 | 3.49 | 3.5 | 3.64 | 3.49 | 3.02 | 3 | 3.43 | 3.86 |
| 2.25 | 3.4 | 3.09 | 3.47 | 3.61 | 3.61 | 3.4 | 3.25 | 3.48 | 3.54 | 2.89 | 2.91 | 3.3 | 4.13 |
| 2.5 | 3.41 | 3.19 | 3.46 | 3.5 | 3.47 | 3.35 | 3.09 | 3.31 | 3.57 | 2.66 | 2.82 | 3.16 | 4.57 |
| 2.75 | 3.38 | 3.24 | 3.29 | 3.33 | 3.29 | 3.27 | 3.02 | 3.04 | 3.59 | 2.51 | 2.69 | 2.98 | 4.97 |
| 3 | 3.16 | 3.34 | 3.19 | 3.24 | 3.31 | 3.06 | 2.86 | 2.99 | 3.21 | 2.45 | 2.56 | 2.89 | 4.92 |
| 3.25 | 2.96 | 3.27 | 2.98 | 3.07 | 3.06 | 3 | 2.68 | 2.83 | 2.89 | 2.28 | 2.43 | 2.69 | 4.63 |
| 3.5 | 2.71 | 3.19 | 2.74 | 2.84 | 2.9 | 2.75 | 2.51 | 2.71 | 2.61 | 2.15 | 2.3 | 2.56 | 4.1 |
| 3.75 | 2.41 | 2.82 | 2.57 | 2.68 | 2.72 | 2.59 | 2.28 | 2.49 | 2.3 | 1.95 | 2.22 | 2.44 | 3.5 |
| 4 | 2.23 | 2.62 | 2.34 | 2.48 | 2.38 | 2.44 | 2.17 | 2.3 | 2.04 | 1.91 | 2.14 | 2.37 | 2.98 |
| 4.25 | 2.02 | 2.43 | 2.18 | 2.27 | 2.18 | 2.27 | 2.08 | 2.14 | 1.97 | 1.85 | 2.04 | 2.27 | 2.52 |
| 4.5 | 1.89 | 2.24 | 2.08 | 2.15 | 1.93 | 2.13 | 1.94 | 2.02 | 1.81 | 1.73 | 2.06 | 2.12 | 2.12 |
| 4.75 | 1.78 | 2.16 | 1.92 | 2.01 | 1.8 | 1.99 | 1.87 | 1.89 | 1.7 | 1.63 | 1.96 | 2.07 | 1.85 |
| 5 | 1.7 | 1.99 | 1.82 | 1.93 | 1.71 | 1.87 | 1.78 | 1.8 | 1.62 | 1.58 | 1.88 | 1.98 | 1.67 |
| 5.25 | 1.65 | 1.87 | 1.72 | 1.82 | 1.64 | 1.78 | 1.64 | 1.7 | 1.51 | 1.48 | 1.82 | 1.84 | 1.49 |
| 5.5 | 1.57 | 1.82 | 1.63 | 1.72 | 1.59 | 1.7 | 1.58 | 1.63 | 1.46 | 1.44 | 1.78 | 1.79 | 1.41 |
| 5.75 | 1.49 | 1.78 | 1.56 | 1.71 | 1.52 | 1.62 | 1.52 | 1.58 | 1.42 | 1.4 | 1.69 | 1.73 | 1.34 |
| 6 | 1.47 | 1.69 | 1.53 | 1.6 | 1.48 | 1.54 | 1.41 | 1.48 | 1.37 | 1.36 | 1.64 | 1.68 | 1.31 |
| 6.25 | 1.47 | 1.74 | 1.47 | 1.55 | 1.47 | 1.53 | 1.34 | 1.49 | 1.31 | 1.35 | 1.59 | 1.63 | 1.28 |
| 6.5 | 1.46 | 1.66 | 1.5 | 1.52 | 1.48 | 1.52 | 1.3 | 1.48 | 1.31 | 1.33 | 1.56 | 1.6 | 1.28 |
| 6.75 | 1.43 | 1.72 | 1.49 | 1.53 | 1.4 | 1.43 | 1.27 | 1.46 | 1.25 | 1.31 | 1.57 | 1.61 | 1.26 |
| 7 | 1.39 | 1.67 | 1.45 | 1.49 | 1.36 | 1.4 | 1.18 | 1.4 | 1.29 | 1.33 | 1.59 | 1.56 | 1.22 |
| 7.25 | 1.35 | 1.65 | 1.38 | 1.4 | 1.34 | 1.35 | 1.13 | 1.36 | 1.28 | 1.32 | 1.49 | 1.57 | 1.17 |
| 7.5 | 1.3 | 1.64 | 1.34 | 1.37 | 1.26 | 1.28 | 1.05 | 1.29 | 1.24 | 1.29 | 1.47 | 1.51 | 1.1 |
| 7.75 | 1.26 | 1.57 | 1.27 | 1.3 | 1.24 | 1.21 | 1.01 | 1.25 | 1.18 | 1.2 | 1.46 | 1.43 | 1.02 |
| 8 | 1.22 | 1.58 | 1.19 | 1.18 | 1.19 | 1.12 | 0.93 | 1.17 | 1.11 | 1.15 | 1.37 | 1.39 | 0.95 |
| 8.25 | 1.2 | 1.53 | 1.16 | 1.18 | 1.12 | 1.03 | 0.89 | 1.11 | 1.05 | 1.12 | 1.31 | 1.33 | 0.91 |

| | | | | | | | | | | | | | |
|---|---|---|---|---|---|---|---|---|---|---|---|---|---|
| 8.5 | 1.14 | 1.45 | 1.08 | 1.1 | 1.07 | 0.98 | 0.84 | 1.06 | 0.98 | 1.06 | 1.27 | 1.28 | 0.84 |
| 8.75 | 1.07 | 1.4 | 1.02 | 1.03 | 0.99 | 0.94 | 0.81 | 0.99 | 0.95 | 1.02 | 1.2 | 1.22 | 0.79 |
| 9 | 1.03 | 1.31 | 1.01 | 0.98 | 0.93 | 0.86 | 0.76 | 0.95 | 0.87 | 0.94 | 1.15 | 1.17 | 0.74 |
| 9.25 | 0.96 | 1.25 | 0.93 | 0.92 | 0.9 | 0.81 | 0.74 | 0.89 | 0.85 | 0.92 | 1.13 | 1.09 | 0.69 |
| 9.5 | 0.91 | 1.2 | 0.9 | 0.86 | 0.84 | 0.77 | 0.71 | 0.87 | 0.82 | 0.87 | 1.06 | 1.04 | 0.65 |
| 9.75 | 0.84 | 1.07 | 0.85 | 0.81 | 0.79 | 0.72 | 0.69 | 0.81 | 0.76 | 0.83 | 0.99 | 0.95 | 0.6 |
| 10 | 0.8 | 1.06 | 0.78 | 0.76 | 0.73 | 0.7 | 0.65 | 0.77 | 0.72 | 0.77 | 0.96 | 0.92 | 0.58 |
| 10.25 | 0.75 | 0.99 | 0.74 | 0.71 | 0.7 | 0.66 | 0.62 | 0.73 | 0.67 | 0.75 | 0.92 | 0.82 | 0.55 |
| 10.5 | 0.72 | 0.92 | 0.7 | 0.66 | 0.65 | 0.63 | 0.61 | 0.69 | 0.66 | 0.7 | 0.84 | 0.78 | 0.52 |
| 10.75 | 0.65 | 0.86 | 0.67 | 0.62 | 0.6 | 0.59 | 0.59 | 0.66 | 0.63 | 0.66 | 0.81 | 0.72 | 0.5 |
| 11 | 0.62 | 0.82 | 0.65 | 0.57 | 0.57 | 0.57 | 0.58 | 0.64 | 0.61 | 0.63 | 0.76 | 0.67 | 0.46 |
| 11.25 | 0.59 | 0.75 | 0.61 | 0.55 | 0.54 | 0.55 | 0.53 | 0.6 | 0.6 | 0.61 | 0.76 | 0.63 | 0.45 |
| 11.5 | 0.55 | 0.68 | 0.56 | 0.51 | 0.52 | 0.52 | 0.51 | 0.56 | 0.58 | 0.59 | 0.7 | 0.56 | 0.42 |
| 11.75 | 0.53 | 0.66 | 0.55 | 0.48 | 0.48 | 0.49 | 0.49 | 0.54 | 0.57 | 0.55 | 0.69 | 0.53 | 0.4 |
| 12 | 0.5 | 0.6 | 0.51 | 0.46 | 0.45 | 0.47 | 0.49 | 0.51 | 0.53 | 0.52 | 0.65 | 0.52 | 0.39 |
| 12.25 | 0.48 | 0.56 | 0.48 | 0.43 | 0.43 | 0.44 | 0.46 | 0.48 | 0.52 | 0.5 | 0.63 | 0.49 | 0.37 |
| 12.5 | 0.45 | 0.5 | 0.46 | 0.4 | 0.42 | 0.43 | 0.46 | 0.46 | 0.5 | 0.46 | 0.59 | 0.44 | 0.36 |
| 12.75 | 0.42 | 0.48 | 0.44 | 0.39 | 0.39 | 0.4 | 0.43 | 0.44 | 0.47 | 0.44 | 0.56 | 0.43 | 0.33 |
| 13 | 0.4 | 0.46 | 0.42 | 0.37 | 0.36 | 0.38 | 0.42 | 0.42 | 0.46 | 0.43 | 0.54 | 0.41 | 0.33 |
| 13.25 | 0.38 | 0.42 | 0.39 | 0.35 | 0.34 | 0.37 | 0.41 | 0.41 | 0.44 | 0.4 | 0.52 | 0.38 | 0.32 |
| 13.5 | 0.36 | 0.39 | 0.37 | 0.33 | 0.33 | 0.35 | 0.38 | 0.39 | 0.43 | 0.4 | 0.51 | 0.35 | 0.3 |
| 13.75 | 0.35 | 0.37 | 0.36 | 0.32 | 0.31 | 0.33 | 0.37 | 0.37 | 0.41 | 0.38 | 0.48 | 0.34 | 0.29 |
| 14 | 0.33 | 0.34 | 0.34 | 0.3 | 0.3 | 0.31 | 0.35 | 0.34 | 0.4 | 0.36 | 0.46 | 0.32 | 0.28 |
| 14.25 | 0.31 | 0.33 | 0.32 | 0.28 | 0.28 | 0.3 | 0.34 | 0.32 | 0.39 | 0.36 | 0.45 | 0.3 | 0.27 |
| 14.5 | 0.29 | 0.3 | 0.31 | 0.27 | 0.26 | 0.28 | 0.32 | 0.31 | 0.36 | 0.32 | 0.42 | 0.29 | 0.26 |
| 14.75 | 0.28 | 0.28 | 0.29 | 0.25 | 0.25 | 0.27 | 0.3 | 0.29 | 0.34 | 0.31 | 0.4 | 0.27 | 0.25 |
| 15 | 0.26 | 0.26 | 0.27 | 0.24 | 0.23 | 0.25 | 0.28 | 0.27 | 0.32 | 0.3 | 0.37 | 0.26 | 0.24 |
| 15.25 | 0.26 | 0.24 | 0.26 | 0.23 | 0.22 | 0.24 | 0.28 | 0.26 | 0.3 | 0.28 | 0.36 | 0.25 | 0.23 |
| 15.5 | 0.24 | 0.23 | 0.24 | 0.21 | 0.21 | 0.23 | 0.26 | 0.25 | 0.28 | 0.27 | 0.35 | 0.23 | 0.22 |
| 15.75 | 0.23 | 0.22 | 0.23 | 0.21 | 0.2 | 0.22 | 0.24 | 0.23 | 0.27 | 0.26 | 0.32 | 0.22 | 0.21 |
| 16 | 0.22 | 0.21 | 0.21 | 0.19 | 0.19 | 0.21 | 0.23 | 0.22 | 0.25 | 0.25 | 0.3 | 0.21 | 0.2 |
| 16.25 | 0.21 | 0.19 | 0.2 | 0.18 | 0.18 | 0.2 | 0.21 | 0.21 | 0.23 | 0.24 | 0.28 | 0.2 | 0.19 |
| 16.5 | 0.2 | 0.18 | 0.19 | 0.17 | 0.17 | 0.19 | 0.2 | 0.19 | 0.22 | 0.22 | 0.27 | 0.19 | 0.19 |
| 16.75 | 0.2 | 0.17 | 0.18 | 0.17 | 0.16 | 0.17 | 0.19 | 0.18 | 0.21 | 0.21 | 0.26 | 0.17 | 0.18 |
| 17 | 0.19 | 0.16 | 0.17 | 0.16 | 0.15 | 0.16 | 0.18 | 0.17 | 0.2 | 0.2 | 0.24 | 0.17 | 0.17 |
| 17.25 | 0.18 | 0.15 | 0.16 | 0.15 | 0.15 | 0.16 | 0.17 | 0.16 | 0.2 | 0.19 | 0.23 | 0.15 | 0.16 |
| 17.5 | 0.17 | 0.15 | 0.15 | 0.14 | 0.14 | 0.15 | 0.17 | 0.15 | 0.18 | 0.18 | 0.21 | 0.15 | 0.16 |
| 17.75 | 0.17 | 0.13 | 0.14 | 0.14 | 0.13 | 0.14 | 0.16 | 0.15 | 0.17 | 0.17 | 0.2 | 0.14 | 0.15 |
| 18 | 0.16 | 0.14 | 0.14 | 0.13 | 0.12 | 0.14 | 0.15 | 0.14 | 0.16 | 0.16 | 0.19 | 0.14 | 0.14 |
| 18.25 | 0.15 | 0.13 | 0.13 | 0.12 | 0.12 | 0.13 | 0.14 | 0.13 | 0.15 | 0.16 | 0.18 | 0.13 | 0.14 |
| 18.5 | 0.15 | 0.12 | 0.13 | 0.12 | 0.11 | 0.12 | 0.14 | 0.12 | 0.15 | 0.15 | 0.17 | 0.12 | 0.13 |
| 18.75 | 0.15 | 0.12 | 0.12 | 0.11 | 0.11 | 0.12 | 0.14 | 0.12 | 0.14 | 0.14 | 0.16 | 0.12 | 0.13 |
| 19 | 0.14 | 0.11 | 0.12 | 0.11 | 0.1 | 0.11 | 0.13 | 0.11 | 0.13 | 0.13 | 0.16 | 0.12 | 0.12 |
| 19.25 | 0.14 | 0.11 | 0.11 | 0.1 | 0.1 | 0.11 | 0.12 | 0.11 | 0.12 | 0.13 | 0.15 | 0.11 | 0.11 |
| 19.5 | 0.13 | 0.1 | 0.11 | 0.1 | 0.1 | 0.1 | 0.11 | 0.1 | 0.12 | 0.12 | 0.15 | 0.11 | 0.11 |
| 19.75 | 0.13 | 0.1 | 0.1 | 0.1 | 0.09 | 0.1 | 0.11 | 0.1 | 0.12 | 0.12 | 0.14 | 0.1 | 0.11 |
| 20 | 0.12 | 0.09 | 0.1 | 0.09 | 0.09 | 0.09 | 0.11 | 0.09 | 0.11 | 0.11 | 0.13 | 0.1 | 0.1 |

| **TRM** | | | | | | | | | | | | | | | |
|---|---|---|---|---|---|---|---|---|---|---|---|---|---|---|---|
| Hz | | | | | | | | | | | | | | | |
| 0.25 | 2.1 | 1.45 | 1.3 | 1.34 | 0.92 | 1.76 | 1.47 | 2.83 | 2.07 | 1.25 | 1.44 | 0.99 | 1.3 | 1.87 | 1.33 |
| 0.5 | 2.8 | 2.25 | 2.3 | 2.31 | 1.53 | 2.81 | 2.27 | 2.57 | 2.48 | 2 | 2.05 | 2.06 | 2.35 | 2.75 | 2.64 |
| 0.75 | 3.6 | 2.85 | 3.22 | 3.07 | 2.02 | 3.55 | 3.22 | 3.03 | 3.46 | 2.63 | 2.64 | 3.09 | 3.01 | 4.01 | 3.63 |
| 1 | 3.77 | 3.25 | 3.58 | 3.46 | 2.15 | 3.88 | 3.78 | 3.31 | 3.86 | 3 | 3.04 | 3.6 | 3.06 | 4.37 | 3.92 |
| 1.25 | 3.69 | 3.32 | 3.71 | 3.69 | 2.32 | 4 | 4.04 | 3.29 | 3.98 | 3.09 | 3.19 | 3.63 | 2.98 | 4.34 | 3.84 |
| 1.5 | 3.54 | 3.37 | 3.89 | 3.9 | 2.44 | 3.82 | 3.95 | 3.26 | 4.07 | 3.07 | 3.07 | 3.53 | 2.88 | 4.26 | 3.67 |
| 1.75 | 3.43 | 3.34 | 3.89 | 3.97 | 2.72 | 3.77 | 3.87 | 3.14 | 3.77 | 3.07 | 3.07 | 3.48 | 2.94 | 4.01 | 3.47 |
| 2 | 3.16 | 3.19 | 3.81 | 3.84 | 2.94 | 3.6 | 3.67 | 3.13 | 3.56 | 3.13 | 3.1 | 3.47 | 2.89 | 3.74 | 3.38 |
| 2.25 | 3 | 3.16 | 3.95 | 3.83 | 3.3 | 3.43 | 3.39 | 3.21 | 3.32 | 3.27 | 3.17 | 3.25 | 2.85 | 3.67 | 3.4 |
| 2.5 | 2.98 | 3.04 | 3.91 | 3.79 | 3.73 | 3.35 | 3.21 | 3.23 | 3.19 | 3.31 | 3.34 | 3.28 | 2.85 | 3.54 | 3.39 |
| 2.75 | 2.96 | 3.05 | 3.82 | 3.65 | 4.36 | 3.31 | 3.06 | 3.16 | 3.18 | 3.21 | 3.54 | 3.14 | 2.89 | 3.38 | 3.24 |
| 3 | 2.92 | 3.04 | 3.7 | 3.54 | 4.7 | 3.34 | 2.96 | 3.04 | 3.12 | 3.12 | 3.36 | 3.06 | 2.9 | 3.03 | 3.15 |
| 3.25 | 2.84 | 2.97 | 3.5 | 3.57 | 4.77 | 3.4 | 2.83 | 2.99 | 2.9 | 3.08 | 3.12 | 2.93 | 2.76 | 2.77 | 2.97 |
| 3.5 | 2.72 | 2.84 | 3.3 | 3.52 | 4.3 | 3.2 | 2.72 | 2.83 | 2.72 | 2.95 | 2.92 | 2.84 | 2.69 | 2.6 | 2.74 |
| 3.75 | 2.52 | 2.72 | 3.09 | 3.47 | 3.25 | 2.95 | 2.58 | 2.47 | 2.61 | 2.82 | 2.58 | 2.65 | 2.6 | 2.41 | 2.49 |
| 4 | 2.37 | 2.55 | 2.78 | 3.11 | 2.6 | 2.6 | 2.42 | 2.19 | 2.41 | 2.62 | 2.37 | 2.5 | 2.51 | 2.17 | 2.39 |
| 4.25 | 2.31 | 2.37 | 2.42 | 2.77 | 2.18 | 2.33 | 2.29 | 1.97 | 2.29 | 2.47 | 2.19 | 2.29 | 2.34 | 2.03 | 2.22 |
| 4.5 | 2.22 | 2.27 | 2.21 | 2.31 | 2.04 | 2.16 | 2.19 | 1.82 | 2.08 | 2.33 | 2.01 | 2.18 | 2.25 | 1.9 | 2.12 |
| 4.75 | 2.1 | 2.18 | 2.02 | 2.08 | 1.82 | 1.95 | 2.11 | 1.76 | 2.04 | 2.23 | 1.86 | 2.06 | 2.18 | 1.77 | 2.03 |
| 5 | 1.99 | 2.11 | 1.89 | 1.89 | 1.76 | 1.83 | 2.01 | 1.7 | 1.95 | 2.01 | 1.74 | 1.93 | 1.99 | 1.74 | 1.99 |
| 5.25 | 1.94 | 1.98 | 1.79 | 1.77 | 1.72 | 1.71 | 1.89 | 1.66 | 1.89 | 1.95 | 1.65 | 1.85 | 1.97 | 1.66 | 1.95 |
| 5.5 | 1.88 | 1.91 | 1.69 | 1.68 | 1.7 | 1.57 | 1.8 | 1.61 | 1.77 | 1.89 | 1.58 | 1.81 | 1.85 | 1.58 | 1.94 |
| 5.75 | 1.79 | 1.86 | 1.62 | 1.6 | 1.66 | 1.56 | 1.73 | 1.58 | 1.74 | 1.82 | 1.55 | 1.75 | 1.83 | 1.57 | 1.91 |
| 6 | 1.73 | 1.77 | 1.57 | 1.58 | 1.64 | 1.48 | 1.7 | 1.56 | 1.65 | 1.77 | 1.5 | 1.75 | 1.75 | 1.55 | 1.88 |
| 6.25 | 1.71 | 1.68 | 1.59 | 1.51 | 1.66 | 1.41 | 1.68 | 1.51 | 1.67 | 1.79 | 1.47 | 1.74 | 1.8 | 1.53 | 1.85 |
| 6.5 | 1.72 | 1.61 | 1.54 | 1.46 | 1.7 | 1.37 | 1.59 | 1.54 | 1.61 | 1.72 | 1.46 | 1.69 | 1.77 | 1.51 | 1.75 |
| 6.75 | 1.64 | 1.62 | 1.52 | 1.48 | 1.72 | 1.35 | 1.58 | 1.51 | 1.6 | 1.67 | 1.43 | 1.7 | 1.72 | 1.51 | 1.73 |
| 7 | 1.59 | 1.57 | 1.46 | 1.4 | 1.62 | 1.28 | 1.5 | 1.51 | 1.57 | 1.62 | 1.41 | 1.61 | 1.69 | 1.43 | 1.62 |
| 7.25 | 1.56 | 1.51 | 1.39 | 1.35 | 1.64 | 1.25 | 1.43 | 1.46 | 1.5 | 1.58 | 1.36 | 1.62 | 1.68 | 1.38 | 1.49 |
| 7.5 | 1.5 | 1.41 | 1.33 | 1.26 | 1.59 | 1.2 | 1.42 | 1.43 | 1.41 | 1.49 | 1.32 | 1.56 | 1.6 | 1.3 | 1.39 |
| 7.75 | 1.36 | 1.38 | 1.26 | 1.2 | 1.51 | 1.17 | 1.31 | 1.35 | 1.35 | 1.4 | 1.24 | 1.45 | 1.52 | 1.23 | 1.3 |
| 8 | 1.28 | 1.32 | 1.2 | 1.12 | 1.52 | 1.12 | 1.24 | 1.31 | 1.31 | 1.36 | 1.18 | 1.44 | 1.4 | 1.18 | 1.2 |
| 8.25 | 1.2 | 1.21 | 1.13 | 1.06 | 1.42 | 1.04 | 1.17 | 1.23 | 1.21 | 1.29 | 1.13 | 1.37 | 1.37 | 1.1 | 1.12 |
| 8.5 | 1.1 | 1.15 | 1.05 | 1.01 | 1.35 | 0.98 | 1.09 | 1.16 | 1.14 | 1.24 | 1.07 | 1.29 | 1.3 | 1.05 | 1.05 |
| 8.75 | 1.04 | 1.09 | 0.98 | 0.9 | 1.27 | 0.93 | 1.02 | 1.09 | 1.07 | 1.13 | 1 | 1.22 | 1.22 | 0.97 | 0.97 |
| 9 | 0.98 | 1.04 | 0.93 | 0.87 | 1.17 | 0.87 | 0.95 | 1.05 | 1.04 | 1.05 | 0.96 | 1.17 | 1.13 | 0.91 | 0.91 |
| 9.25 | 0.91 | 0.98 | 0.87 | 0.79 | 1.12 | 0.83 | 0.92 | 0.99 | 0.98 | 1.03 | 0.9 | 1.06 | 1.1 | 0.86 | 0.85 |
| 9.5 | 0.87 | 0.94 | 0.8 | 0.75 | 1.04 | 0.79 | 0.86 | 0.93 | 0.91 | 0.95 | 0.87 | 1 | 1.05 | 0.82 | 0.8 |
| 9.75 | 0.81 | 0.91 | 0.75 | 0.7 | 0.98 | 0.74 | 0.81 | 0.85 | 0.83 | 0.9 | 0.83 | 0.96 | 0.98 | 0.79 | 0.75 |
| 10 | 0.79 | 0.84 | 0.68 | 0.66 | 0.88 | 0.71 | 0.78 | 0.82 | 0.79 | 0.85 | 0.76 | 0.88 | 0.93 | 0.74 | 0.71 |
| 10.25 | 0.74 | 0.81 | 0.65 | 0.6 | 0.84 | 0.68 | 0.74 | 0.76 | 0.73 | 0.82 | 0.75 | 0.79 | 0.86 | 0.69 | 0.66 |
| 10.5 | 0.69 | 0.75 | 0.62 | 0.57 | 0.76 | 0.65 | 0.69 | 0.72 | 0.68 | 0.77 | 0.7 | 0.77 | 0.84 | 0.67 | 0.63 |
| 10.75 | 0.64 | 0.71 | 0.56 | 0.53 | 0.71 | 0.6 | 0.67 | 0.67 | 0.64 | 0.73 | 0.64 | 0.71 | 0.81 | 0.63 | 0.59 |
| 11 | 0.61 | 0.69 | 0.53 | 0.5 | 0.64 | 0.58 | 0.6 | 0.64 | 0.59 | 0.68 | 0.62 | 0.66 | 0.76 | 0.6 | 0.56 |
| 11.25 | 0.57 | 0.65 | 0.49 | 0.47 | 0.59 | 0.55 | 0.57 | 0.6 | 0.56 | 0.65 | 0.59 | 0.62 | 0.72 | 0.55 | 0.53 |
| 11.5 | 0.54 | 0.61 | 0.45 | 0.45 | 0.56 | 0.52 | 0.55 | 0.56 | 0.53 | 0.61 | 0.55 | 0.58 | 0.68 | 0.54 | 0.51 |

| | | | | | | | | | | | | | | | |
|---|---|---|---|---|---|---|---|---|---|---|---|---|---|---|---|
| 11.75 | 0.51 | 0.58 | 0.43 | 0.42 | 0.52 | 0.5 | 0.52 | 0.54 | 0.5 | 0.59 | 0.52 | 0.53 | 0.63 | 0.5 | 0.48 |
| 12 | 0.48 | 0.55 | 0.41 | 0.41 | 0.47 | 0.48 | 0.5 | 0.51 | 0.46 | 0.56 | 0.49 | 0.5 | 0.61 | 0.48 | 0.45 |
| 12.25 | 0.46 | 0.52 | 0.39 | 0.38 | 0.46 | 0.46 | 0.47 | 0.48 | 0.44 | 0.53 | 0.47 | 0.47 | 0.57 | 0.46 | 0.42 |
| 12.5 | 0.44 | 0.49 | 0.36 | 0.37 | 0.42 | 0.43 | 0.44 | 0.46 | 0.42 | 0.5 | 0.44 | 0.45 | 0.56 | 0.43 | 0.4 |
| 12.75 | 0.42 | 0.48 | 0.34 | 0.35 | 0.39 | 0.41 | 0.44 | 0.44 | 0.39 | 0.46 | 0.43 | 0.41 | 0.5 | 0.4 | 0.37 |
| 13 | 0.4 | 0.45 | 0.32 | 0.33 | 0.37 | 0.4 | 0.41 | 0.42 | 0.37 | 0.44 | 0.4 | 0.39 | 0.48 | 0.38 | 0.36 |
| 13.25 | 0.38 | 0.45 | 0.3 | 0.32 | 0.34 | 0.37 | 0.39 | 0.39 | 0.35 | 0.42 | 0.39 | 0.36 | 0.46 | 0.37 | 0.34 |
| 13.5 | 0.37 | 0.41 | 0.29 | 0.29 | 0.32 | 0.36 | 0.37 | 0.38 | 0.32 | 0.4 | 0.36 | 0.34 | 0.43 | 0.35 | 0.32 |
| 13.75 | 0.35 | 0.4 | 0.27 | 0.28 | 0.3 | 0.34 | 0.35 | 0.36 | 0.3 | 0.38 | 0.35 | 0.32 | 0.41 | 0.34 | 0.3 |
| 14 | 0.33 | 0.38 | 0.25 | 0.27 | 0.29 | 0.33 | 0.34 | 0.34 | 0.28 | 0.37 | 0.32 | 0.31 | 0.38 | 0.32 | 0.29 |
| 14.25 | 0.32 | 0.37 | 0.25 | 0.26 | 0.26 | 0.31 | 0.32 | 0.33 | 0.27 | 0.35 | 0.31 | 0.29 | 0.36 | 0.3 | 0.27 |
| 14.5 | 0.3 | 0.34 | 0.24 | 0.25 | 0.25 | 0.3 | 0.3 | 0.32 | 0.26 | 0.32 | 0.29 | 0.27 | 0.35 | 0.29 | 0.26 |
| 14.75 | 0.29 | 0.33 | 0.22 | 0.23 | 0.24 | 0.28 | 0.3 | 0.3 | 0.24 | 0.31 | 0.28 | 0.25 | 0.32 | 0.27 | 0.25 |
| 15 | 0.28 | 0.31 | 0.21 | 0.22 | 0.22 | 0.27 | 0.28 | 0.28 | 0.23 | 0.29 | 0.27 | 0.24 | 0.3 | 0.25 | 0.24 |
| 15.25 | 0.26 | 0.3 | 0.19 | 0.21 | 0.22 | 0.26 | 0.26 | 0.27 | 0.21 | 0.28 | 0.26 | 0.23 | 0.29 | 0.24 | 0.23 |
| 15.5 | 0.25 | 0.28 | 0.19 | 0.2 | 0.2 | 0.24 | 0.25 | 0.26 | 0.2 | 0.26 | 0.25 | 0.22 | 0.26 | 0.23 | 0.21 |
| 15.75 | 0.24 | 0.26 | 0.18 | 0.19 | 0.19 | 0.23 | 0.23 | 0.25 | 0.19 | 0.25 | 0.23 | 0.21 | 0.25 | 0.21 | 0.21 |
| 16 | 0.23 | 0.25 | 0.17 | 0.18 | 0.18 | 0.22 | 0.22 | 0.23 | 0.18 | 0.25 | 0.22 | 0.19 | 0.25 | 0.21 | 0.2 |
| 16.25 | 0.21 | 0.24 | 0.16 | 0.17 | 0.18 | 0.21 | 0.21 | 0.22 | 0.17 | 0.22 | 0.21 | 0.19 | 0.23 | 0.19 | 0.19 |
| 16.5 | 0.2 | 0.23 | 0.15 | 0.16 | 0.17 | 0.2 | 0.2 | 0.21 | 0.16 | 0.21 | 0.2 | 0.17 | 0.23 | 0.19 | 0.18 |
| 16.75 | 0.2 | 0.21 | 0.14 | 0.16 | 0.16 | 0.19 | 0.19 | 0.21 | 0.15 | 0.2 | 0.19 | 0.17 | 0.2 | 0.17 | 0.17 |
| 17 | 0.18 | 0.21 | 0.14 | 0.15 | 0.15 | 0.19 | 0.18 | 0.2 | 0.15 | 0.19 | 0.18 | 0.16 | 0.19 | 0.16 | 0.16 |
| 17.25 | 0.17 | 0.19 | 0.13 | 0.14 | 0.14 | 0.17 | 0.17 | 0.18 | 0.14 | 0.18 | 0.18 | 0.15 | 0.19 | 0.16 | 0.16 |
| 17.5 | 0.16 | 0.18 | 0.12 | 0.13 | 0.14 | 0.16 | 0.16 | 0.18 | 0.14 | 0.17 | 0.17 | 0.14 | 0.17 | 0.15 | 0.15 |
| 17.75 | 0.16 | 0.17 | 0.12 | 0.13 | 0.13 | 0.16 | 0.16 | 0.17 | 0.13 | 0.16 | 0.16 | 0.14 | 0.17 | 0.14 | 0.15 |
| 18 | 0.15 | 0.16 | 0.11 | 0.12 | 0.13 | 0.15 | 0.15 | 0.16 | 0.12 | 0.16 | 0.16 | 0.13 | 0.16 | 0.14 | 0.14 |
| 18.25 | 0.14 | 0.16 | 0.11 | 0.12 | 0.12 | 0.15 | 0.14 | 0.16 | 0.12 | 0.15 | 0.15 | 0.13 | 0.15 | 0.13 | 0.13 |
| 18.5 | 0.14 | 0.15 | 0.1 | 0.11 | 0.12 | 0.14 | 0.13 | 0.15 | 0.11 | 0.14 | 0.14 | 0.12 | 0.15 | 0.13 | 0.13 |
| 18.75 | 0.13 | 0.14 | 0.1 | 0.1 | 0.11 | 0.13 | 0.13 | 0.15 | 0.11 | 0.14 | 0.14 | 0.12 | 0.14 | 0.12 | 0.12 |
| 19 | 0.12 | 0.13 | 0.09 | 0.1 | 0.11 | 0.13 | 0.12 | 0.14 | 0.1 | 0.13 | 0.13 | 0.11 | 0.13 | 0.11 | 0.12 |
| 19.25 | 0.12 | 0.13 | 0.09 | 0.09 | 0.1 | 0.12 | 0.12 | 0.13 | 0.1 | 0.12 | 0.13 | 0.11 | 0.13 | 0.11 | 0.12 |
| 19.5 | 0.11 | 0.12 | 0.09 | 0.09 | 0.1 | 0.12 | 0.11 | 0.13 | 0.09 | 0.12 | 0.12 | 0.1 | 0.12 | 0.11 | 0.11 |
| 19.75 | 0.11 | 0.11 | 0.08 | 0.09 | 0.09 | 0.11 | 0.11 | 0.12 | 0.09 | 0.11 | 0.12 | 0.1 | 0.12 | 0.1 | 0.11 |
| 20 | 0.11 | 0.11 | 0.08 | 0.08 | 0.1 | 0.11 | 0.1 | 0.12 | 0.09 | 0.11 | 0.11 | 0.1 | 0.11 | 0.1 | 0.1 |

Electroencephalographic power spectral density during REMS after 6 h of sleep deprivation

| **CLF** | | | | | | | | | | | | |
|---|---|---|---|---|---|---|---|---|---|---|---|---|
| Hz | | | | | | | | | | | | |
| 0.25 | 4.59 | 1.73 | 4.82 | 1.6 | 1.73 | 1.86 | 1.11 | 4.37 | 2.53 | 3.77 | 1.13 | 1.3 |
| 0.5 | 2.76 | 2.27 | 2.07 | 2.21 | 1.55 | 2.44 | 1.39 | 2.32 | 2.45 | 2.53 | 1.81 | 1.47 |
| 0.75 | 1.95 | 1.94 | 1.62 | 1.94 | 1.49 | 2.13 | 1.54 | 1.79 | 2.06 | 1.87 | 1.85 | 1.45 |
| 1 | 1.76 | 1.67 | 1.36 | 1.68 | 1.43 | 1.99 | 1.59 | 1.55 | 1.87 | 1.71 | 1.55 | 1.45 |
| 1.25 | 1.59 | 1.53 | 1.39 | 1.67 | 1.46 | 1.6 | 1.51 | 1.54 | 1.7 | 1.56 | 1.55 | 1.5 |
| 1.5 | 1.53 | 1.46 | 1.51 | 1.68 | 1.49 | 1.7 | 1.81 | 1.51 | 1.7 | 1.68 | 1.49 | 1.6 |
| 1.75 | 1.56 | 1.51 | 1.68 | 1.6 | 1.47 | 1.94 | 1.38 | 1.59 | 1.63 | 1.56 | 1.47 | 1.65 |

| | | | | | | | | | | | | |
|---|---|---|---|---|---|---|---|---|---|---|---|---|
| 2 | 1.41 | 1.52 | 1.72 | 1.6 | 1.49 | 2 | 1.97 | 1.52 | 1.58 | 1.51 | 1.47 | 1.51 |
| 2.25 | 1.47 | 1.6 | 1.86 | 1.6 | 1.44 | 1.93 | 1.62 | 1.51 | 1.7 | 1.42 | 1.49 | 1.58 |
| 2.5 | 1.48 | 1.65 | 1.85 | 1.73 | 1.5 | 1.9 | 1.7 | 1.5 | 1.61 | 1.58 | 1.62 | 1.7 |
| 2.75 | 1.45 | 1.75 | 1.72 | 1.72 | 1.5 | 1.84 | 1.42 | 1.47 | 1.71 | 1.54 | 1.58 | 1.72 |
| 3 | 1.47 | 1.59 | 1.65 | 1.61 | 1.55 | 1.69 | 1.11 | 1.45 | 1.61 | 1.67 | 1.57 | 1.61 |
| 3.25 | 1.43 | 1.75 | 1.53 | 1.65 | 1.43 | 1.57 | 1.31 | 1.44 | 1.44 | 1.52 | 1.48 | 1.46 |
| 3.5 | 1.3 | 1.47 | 1.54 | 1.61 | 1.33 | 1.55 | 1.19 | 1.39 | 1.41 | 1.48 | 1.33 | 1.44 |
| 3.75 | 1.28 | 1.47 | 1.39 | 1.47 | 1.18 | 1.58 | 1.16 | 1.37 | 1.34 | 1.39 | 1.18 | 1.4 |
| 4 | 1.27 | 1.32 | 1.42 | 1.45 | 1.16 | 1.43 | 1.1 | 1.31 | 1.18 | 1.19 | 1.19 | 1.38 |
| 4.25 | 1.22 | 1.35 | 1.44 | 1.35 | 1.07 | 1.43 | 1.44 | 1.33 | 1.31 | 1.25 | 1.21 | 1.35 |
| 4.5 | 1.25 | 1.2 | 1.39 | 1.44 | 1.03 | 1.46 | 1.74 | 1.44 | 1.21 | 1.16 | 1.17 | 1.48 |
| 4.75 | 1.2 | 1.14 | 1.3 | 1.37 | 1.09 | 1.37 | 2.25 | 1.4 | 1.22 | 1.13 | 1.07 | 1.35 |
| 5 | 1.25 | 1.34 | 1.23 | 1.38 | 1.06 | 1.43 | 2.57 | 1.5 | 1.26 | 1.09 | 1.04 | 1.34 |
| 5.25 | 1.23 | 1.37 | 1.22 | 1.41 | 1.13 | 1.38 | 3.14 | 1.5 | 1.22 | 1.17 | 1.07 | 1.37 |
| 5.5 | 1.37 | 1.5 | 1.27 | 1.52 | 1.24 | 1.42 | 3.79 | 1.52 | 1.18 | 1.38 | 1.11 | 1.52 |
| 5.75 | 1.64 | 1.66 | 1.31 | 1.76 | 1.31 | 1.49 | 4.55 | 1.66 | 1.3 | 1.19 | 1.25 | 1.6 |
| 6 | 1.88 | 2.07 | 1.73 | 2.09 | 1.7 | 1.89 | 5.87 | 1.94 | 1.59 | 1.59 | 1.58 | 1.99 |
| 6.25 | 2.4 | 2.6 | 1.85 | 2.33 | 2.11 | 2.11 | 5.64 | 2.34 | 2.01 | 1.94 | 1.78 | 2.26 |
| 6.5 | 2.97 | 3.17 | 2.31 | 2.94 | 2.49 | 2.79 | 5.56 | 2.65 | 2.35 | 2.51 | 2.26 | 2.91 |
| 6.75 | 3.63 | 3.74 | 2.65 | 3.4 | 3.14 | 3.2 | 5.73 | 3.19 | 3.15 | 3.11 | 2.85 | 3.58 |
| 7 | 4.14 | 4.48 | 3.72 | 4.18 | 3.89 | 4.08 | 3.87 | 3.63 | 3.35 | 3.5 | 3.76 | 3.9 |
| 7.25 | 4.9 | 5.05 | 4.33 | 4.1 | 4.6 | 4.38 | 3.81 | 3.71 | 4.05 | 3.81 | 4.57 | 4.27 |
| 7.5 | 4.92 | 4.39 | 4.65 | 4.68 | 5.13 | 4.75 | 3.38 | 3.89 | 4.1 | 4.43 | 5.26 | 4.39 |
| 7.75 | 4.3 | 3.92 | 4.3 | 4.05 | 4.83 | 4.1 | 2.68 | 4.05 | 3.58 | 3.09 | 4.88 | 4.14 |
| 8 | 3.38 | 3.58 | 3.65 | 3.4 | 4.6 | 3.52 | 1.62 | 3.51 | 3.03 | 2.82 | 4.86 | 3.66 |
| 8.25 | 2.9 | 3.16 | 2.43 | 2.83 | 3.94 | 2.73 | 1.39 | 3.07 | 2.57 | 2.37 | 3.91 | 3.26 |
| 8.5 | 2.47 | 2.42 | 2.16 | 2.54 | 3.45 | 2.28 | 1.25 | 2.75 | 2.12 | 1.84 | 3.31 | 2.82 |
| 8.75 | 2 | 2.07 | 1.64 | 1.97 | 3 | 1.84 | 0.77 | 2.21 | 1.63 | 1.54 | 2.72 | 2.28 |
| 9 | 1.67 | 1.69 | 1.29 | 1.55 | 2.41 | 1.36 | 0.64 | 2.01 | 1.4 | 1.23 | 2.31 | 2.07 |
| 9.25 | 1.29 | 1.38 | 1.21 | 1.32 | 2.04 | 1.12 | 0.7 | 1.65 | 1.23 | 0.95 | 1.89 | 1.75 |
| 9.5 | 1.1 | 1.19 | 1.08 | 1.22 | 1.69 | 0.99 | 0.56 | 1.36 | 1 | 0.94 | 1.61 | 1.44 |
| 9.75 | 0.92 | 1.14 | 0.91 | 0.99 | 1.41 | 0.88 | 0.56 | 1.31 | 0.85 | 0.77 | 1.31 | 1.25 |
| 10 | 0.84 | 0.96 | 0.85 | 0.83 | 1.28 | 0.74 | 0.48 | 1.09 | 0.76 | 0.7 | 1.18 | 1.12 |
| 10.25 | 0.71 | 0.84 | 0.64 | 0.71 | 1.12 | 0.72 | 0.44 | 0.91 | 0.61 | 0.64 | 1.01 | 0.97 |
| 10.5 | 0.61 | 0.67 | 0.56 | 0.68 | 1.02 | 0.67 | 0.46 | 0.85 | 0.6 | 0.49 | 0.92 | 0.89 |
| 10.75 | 0.58 | 0.71 | 0.57 | 0.65 | 0.89 | 0.56 | 0.39 | 0.75 | 0.52 | 0.46 | 0.78 | 0.78 |
| 11 | 0.52 | 0.57 | 0.51 | 0.57 | 0.75 | 0.51 | 0.4 | 0.69 | 0.5 | 0.49 | 0.73 | 0.69 |
| 11.25 | 0.49 | 0.52 | 0.44 | 0.54 | 0.63 | 0.48 | 0.37 | 0.61 | 0.47 | 0.38 | 0.62 | 0.66 |
| 11.5 | 0.45 | 0.51 | 0.41 | 0.51 | 0.63 | 0.47 | 0.32 | 0.59 | 0.4 | 0.42 | 0.6 | 0.58 |
| 11.75 | 0.41 | 0.47 | 0.42 | 0.45 | 0.52 | 0.4 | 0.34 | 0.54 | 0.43 | 0.37 | 0.53 | 0.56 |
| 12 | 0.4 | 0.44 | 0.41 | 0.43 | 0.49 | 0.39 | 0.26 | 0.49 | 0.37 | 0.35 | 0.49 | 0.47 |
| 12.25 | 0.35 | 0.4 | 0.38 | 0.4 | 0.45 | 0.37 | 0.36 | 0.47 | 0.34 | 0.33 | 0.44 | 0.47 |
| 12.5 | 0.34 | 0.38 | 0.37 | 0.41 | 0.39 | 0.37 | 0.32 | 0.44 | 0.32 | 0.33 | 0.42 | 0.44 |
| 12.75 | 0.32 | 0.38 | 0.35 | 0.39 | 0.37 | 0.36 | 0.35 | 0.39 | 0.37 | 0.32 | 0.41 | 0.39 |
| 13 | 0.3 | 0.35 | 0.35 | 0.37 | 0.38 | 0.34 | 0.33 | 0.37 | 0.31 | 0.32 | 0.39 | 0.38 |
| 13.25 | 0.32 | 0.35 | 0.36 | 0.36 | 0.33 | 0.33 | 0.26 | 0.37 | 0.28 | 0.34 | 0.35 | 0.37 |
| 13.5 | 0.31 | 0.33 | 0.37 | 0.33 | 0.32 | 0.33 | 0.3 | 0.35 | 0.35 | 0.32 | 0.34 | 0.35 |
| 13.75 | 0.34 | 0.33 | 0.38 | 0.34 | 0.31 | 0.31 | 0.29 | 0.33 | 0.34 | 0.28 | 0.33 | 0.33 |

| | | | | | | | | | | | | |
|---|---|---|---|---|---|---|---|---|---|---|---|---|
| 14 | 0.3 | 0.31 | 0.37 | 0.32 | 0.29 | 0.33 | 0.32 | 0.32 | 0.33 | 0.3 | 0.3 | 0.32 |
| 14.25 | 0.29 | 0.33 | 0.41 | 0.33 | 0.28 | 0.3 | 0.27 | 0.3 | 0.34 | 0.28 | 0.28 | 0.33 |
| 14.5 | 0.3 | 0.3 | 0.47 | 0.32 | 0.26 | 0.3 | 0.24 | 0.3 | 0.32 | 0.33 | 0.27 | 0.31 |
| 14.75 | 0.28 | 0.29 | 0.42 | 0.32 | 0.25 | 0.28 | 0.23 | 0.29 | 0.33 | 0.27 | 0.27 | 0.28 |
| 15 | 0.29 | 0.3 | 0.45 | 0.31 | 0.25 | 0.29 | 0.2 | 0.27 | 0.31 | 0.29 | 0.26 | 0.29 |
| 15.25 | 0.26 | 0.26 | 0.51 | 0.28 | 0.24 | 0.3 | 0.18 | 0.28 | 0.29 | 0.28 | 0.27 | 0.27 |
| 15.5 | 0.26 | 0.27 | 0.4 | 0.29 | 0.22 | 0.28 | 0.13 | 0.26 | 0.31 | 0.25 | 0.25 | 0.26 |
| 15.75 | 0.26 | 0.26 | 0.37 | 0.29 | 0.22 | 0.27 | 0.16 | 0.25 | 0.28 | 0.24 | 0.24 | 0.26 |
| 16 | 0.22 | 0.24 | 0.4 | 0.27 | 0.21 | 0.3 | 0.14 | 0.24 | 0.29 | 0.24 | 0.24 | 0.26 |
| 16.25 | 0.23 | 0.24 | 0.35 | 0.24 | 0.2 | 0.27 | 0.17 | 0.24 | 0.27 | 0.24 | 0.22 | 0.23 |
| 16.5 | 0.22 | 0.23 | 0.39 | 0.25 | 0.19 | 0.26 | 0.18 | 0.23 | 0.27 | 0.24 | 0.22 | 0.23 |
| 16.75 | 0.21 | 0.23 | 0.31 | 0.24 | 0.18 | 0.24 | 0.14 | 0.22 | 0.25 | 0.21 | 0.2 | 0.23 |
| 17 | 0.21 | 0.2 | 0.3 | 0.23 | 0.18 | 0.24 | 0.13 | 0.21 | 0.24 | 0.23 | 0.19 | 0.21 |
| 17.25 | 0.19 | 0.19 | 0.3 | 0.22 | 0.17 | 0.23 | 0.13 | 0.19 | 0.23 | 0.2 | 0.19 | 0.21 |
| 17.5 | 0.18 | 0.2 | 0.27 | 0.21 | 0.17 | 0.21 | 0.12 | 0.19 | 0.22 | 0.19 | 0.18 | 0.2 |
| 17.75 | 0.17 | 0.19 | 0.28 | 0.22 | 0.16 | 0.21 | 0.11 | 0.18 | 0.22 | 0.2 | 0.18 | 0.19 |
| 18 | 0.16 | 0.18 | 0.28 | 0.2 | 0.16 | 0.2 | 0.12 | 0.17 | 0.19 | 0.17 | 0.17 | 0.19 |
| 18.25 | 0.17 | 0.17 | 0.25 | 0.2 | 0.15 | 0.21 | 0.09 | 0.16 | 0.22 | 0.18 | 0.17 | 0.18 |
| 18.5 | 0.16 | 0.17 | 0.24 | 0.19 | 0.14 | 0.19 | 0.1 | 0.16 | 0.18 | 0.16 | 0.15 | 0.17 |
| 18.75 | 0.15 | 0.17 | 0.26 | 0.19 | 0.13 | 0.17 | 0.1 | 0.15 | 0.2 | 0.17 | 0.15 | 0.17 |
| 19 | 0.16 | 0.16 | 0.23 | 0.17 | 0.12 | 0.19 | 0.11 | 0.15 | 0.19 | 0.16 | 0.15 | 0.16 |
| 19.25 | 0.15 | 0.14 | 0.23 | 0.17 | 0.12 | 0.17 | 0.11 | 0.13 | 0.19 | 0.16 | 0.14 | 0.16 |
| 19.5 | 0.16 | 0.14 | 0.22 | 0.16 | 0.12 | 0.17 | 0.11 | 0.14 | 0.18 | 0.16 | 0.14 | 0.14 |
| 19.75 | 0.15 | 0.14 | 0.2 | 0.16 | 0.12 | 0.16 | 0.08 | 0.13 | 0.18 | 0.16 | 0.14 | 0.14 |
| 20 | 0.13 | 0.13 | 0.2 | 0.16 | 0.11 | 0.16 | 0.09 | 0.12 | 0.16 | 0.13 | 0.13 | 0.14 |

| **CLM** | | | | | | | | | | | |
|---|---|---|---|---|---|---|---|---|---|---|---|
| Hz | | | | | | | | | | | |
| 0.25 | 2.02 | 3.65 | 1.37 | 0.67 | 1.05 | 1.2 | 4.83 | 5.39 | 1.89 | 1.1 | 3.06 |
| 0.5 | 2.12 | 2.35 | 1.94 | 1.09 | 1.33 | 1.87 | 2.06 | 2.39 | 2.19 | 1.51 | 1.6 |
| 0.75 | 2.18 | 2.15 | 1.76 | 1.31 | 1.27 | 1.73 | 1.43 | 1.75 | 1.97 | 1.53 | 1.58 |
| 1 | 1.94 | 1.77 | 1.52 | 1.46 | 1.2 | 1.68 | 1.4 | 1.48 | 1.71 | 1.54 | 1.51 |
| 1.25 | 1.72 | 1.57 | 1.42 | 1.56 | 1.14 | 1.61 | 1.28 | 1.43 | 1.52 | 1.43 | 1.6 |
| 1.5 | 1.62 | 1.55 | 1.48 | 1.52 | 1.17 | 1.65 | 1.2 | 1.48 | 1.45 | 1.48 | 1.52 |
| 1.75 | 1.68 | 1.68 | 1.62 | 1.63 | 1.16 | 1.66 | 1.2 | 1.4 | 1.55 | 1.48 | 1.48 |
| 2 | 1.67 | 1.52 | 1.72 | 1.67 | 1.26 | 1.82 | 1.21 | 1.41 | 1.64 | 1.53 | 1.48 |
| 2.25 | 1.92 | 1.68 | 1.83 | 1.75 | 1.47 | 1.96 | 1.31 | 1.5 | 1.91 | 1.51 | 1.39 |
| 2.5 | 1.84 | 1.52 | 1.88 | 1.77 | 1.63 | 1.88 | 1.43 | 1.41 | 1.91 | 1.54 | 1.48 |
| 2.75 | 1.89 | 1.68 | 1.73 | 1.77 | 1.75 | 1.76 | 1.4 | 1.47 | 1.82 | 1.63 | 1.52 |
| 3 | 1.91 | 1.78 | 1.8 | 1.68 | 1.73 | 1.64 | 1.38 | 1.35 | 1.79 | 1.62 | 1.41 |
| 3.25 | 1.59 | 1.68 | 1.61 | 1.64 | 1.51 | 1.55 | 1.21 | 1.29 | 1.86 | 1.62 | 1.34 |
| 3.5 | 1.68 | 1.75 | 1.46 | 1.59 | 1.32 | 1.46 | 1.21 | 1.28 | 1.86 | 1.6 | 1.27 |
| 3.75 | 1.51 | 1.34 | 1.46 | 1.5 | 1.19 | 1.48 | 1.19 | 1.18 | 1.73 | 1.54 | 1.24 |
| 4 | 1.39 | 1.48 | 1.44 | 1.43 | 1.12 | 1.4 | 1.11 | 1.1 | 1.67 | 1.44 | 1.22 |
| 4.25 | 1.6 | 1.28 | 1.46 | 1.36 | 1.15 | 1.45 | 1.22 | 1.21 | 1.58 | 1.47 | 1.26 |

| | | | | | | | | | | | |
|---|---|---|---|---|---|---|---|---|---|---|---|
| 4.5 | 1.4 | 1.22 | 1.38 | 1.39 | 1.06 | 1.39 | 1.21 | 1.17 | 1.52 | 1.47 | 1.18 |
| 4.75 | 1.38 | 1.17 | 1.33 | 1.45 | 1.18 | 1.37 | 1.31 | 1.19 | 1.5 | 1.47 | 1.28 |
| 5 | 1.38 | 1.11 | 1.36 | 1.38 | 1.2 | 1.36 | 1.38 | 1.22 | 1.34 | 1.44 | 1.34 |
| 5.25 | 1.35 | 1.05 | 1.39 | 1.57 | 1.32 | 1.46 | 1.49 | 1.37 | 1.28 | 1.47 | 1.31 |
| 5.5 | 1.33 | 1.2 | 1.46 | 1.62 | 1.53 | 1.62 | 1.67 | 1.47 | 1.28 | 1.6 | 1.42 |
| 5.75 | 1.42 | 1.31 | 1.64 | 1.98 | 1.91 | 1.74 | 1.95 | 1.69 | 1.42 | 1.87 | 1.65 |
| 6 | 1.71 | 1.62 | 2.17 | 2.55 | 2.53 | 2.49 | 2.47 | 2.26 | 1.67 | 2.37 | 1.9 |
| 6.25 | 2.06 | 1.76 | 2.46 | 3.03 | 3.13 | 3.1 | 2.93 | 2.39 | 2.21 | 2.93 | 2.37 |
| 6.5 | 2.41 | 2.26 | 2.98 | 3.6 | 3.91 | 3.65 | 3.23 | 2.86 | 2.34 | 3.46 | 2.88 |
| 6.75 | 3.17 | 3.21 | 3.54 | 4.36 | 4.51 | 4.01 | 3.68 | 3.11 | 2.63 | 3.95 | 3.68 |
| 7 | 3.87 | 4.08 | 3.87 | 4.71 | 4.84 | 4.68 | 3.79 | 3.67 | 3.36 | 4.28 | 4.16 |
| 7.25 | 4.43 | 4.65 | 4.3 | 4.71 | 5.22 | 4.47 | 4.15 | 3.9 | 3.98 | 4.5 | 4.61 |
| 7.5 | 4.81 | 5.49 | 4.77 | 4.56 | 4.92 | 4.07 | 4.02 | 3.98 | 4.89 | 4.43 | 4.52 |
| 7.75 | 4.09 | 4.93 | 4.33 | 4.05 | 4.5 | 3.49 | 3.75 | 3.79 | 5.01 | 3.97 | 4.3 |
| 8 | 3.3 | 3.41 | 3.47 | 3.1 | 3.44 | 2.9 | 3.28 | 3.18 | 4.11 | 3.43 | 3.81 |
| 8.25 | 2.75 | 2.95 | 3.27 | 2.47 | 2.96 | 2.33 | 2.76 | 2.63 | 3.28 | 3.03 | 3.16 |
| 8.5 | 2.41 | 2.14 | 2.69 | 2.01 | 2.55 | 2.03 | 2.31 | 2.29 | 2.53 | 2.55 | 2.61 |
| 8.75 | 1.84 | 1.81 | 2.12 | 1.55 | 2.23 | 1.66 | 2.07 | 1.88 | 1.96 | 2.14 | 2.32 |
| 9 | 1.45 | 1.42 | 1.81 | 1.29 | 1.92 | 1.5 | 1.78 | 1.59 | 1.49 | 1.96 | 2.01 |
| 9.25 | 1.14 | 1.1 | 1.45 | 1.09 | 1.63 | 1.27 | 1.51 | 1.3 | 1.28 | 1.55 | 1.64 |
| 9.5 | 0.99 | 0.99 | 1.27 | 0.9 | 1.4 | 1.11 | 1.32 | 1.17 | 1.09 | 1.3 | 1.33 |
| 9.75 | 0.8 | 0.82 | 1.09 | 0.75 | 1.19 | 1.02 | 1.24 | 0.99 | 0.99 | 1.15 | 1.23 |
| 10 | 0.68 | 0.75 | 0.96 | 0.72 | 0.97 | 0.88 | 1.05 | 0.89 | 0.78 | 1.03 | 1.14 |
| 10.25 | 0.74 | 0.62 | 0.83 | 0.64 | 0.98 | 0.78 | 0.93 | 0.84 | 0.81 | 0.91 | 0.95 |
| 10.5 | 0.62 | 0.58 | 0.72 | 0.54 | 0.84 | 0.73 | 0.9 | 0.72 | 0.68 | 0.79 | 0.85 |
| 10.75 | 0.61 | 0.5 | 0.64 | 0.56 | 0.76 | 0.65 | 0.8 | 0.7 | 0.58 | 0.71 | 0.74 |
| 11 | 0.53 | 0.45 | 0.61 | 0.47 | 0.66 | 0.55 | 0.73 | 0.62 | 0.54 | 0.64 | 0.68 |
| 11.25 | 0.49 | 0.43 | 0.55 | 0.42 | 0.57 | 0.52 | 0.71 | 0.6 | 0.46 | 0.59 | 0.64 |
| 11.5 | 0.48 | 0.41 | 0.51 | 0.39 | 0.59 | 0.52 | 0.64 | 0.6 | 0.42 | 0.53 | 0.57 |
| 11.75 | 0.43 | 0.37 | 0.46 | 0.39 | 0.48 | 0.47 | 0.59 | 0.51 | 0.46 | 0.52 | 0.54 |
| 12 | 0.42 | 0.38 | 0.45 | 0.37 | 0.47 | 0.46 | 0.53 | 0.48 | 0.4 | 0.47 | 0.45 |
| 12.25 | 0.39 | 0.32 | 0.44 | 0.37 | 0.41 | 0.45 | 0.51 | 0.45 | 0.39 | 0.45 | 0.46 |
| 12.5 | 0.36 | 0.34 | 0.37 | 0.35 | 0.4 | 0.43 | 0.47 | 0.43 | 0.35 | 0.4 | 0.42 |
| 12.75 | 0.34 | 0.32 | 0.37 | 0.38 | 0.38 | 0.4 | 0.45 | 0.41 | 0.36 | 0.39 | 0.38 |
| 13 | 0.36 | 0.32 | 0.34 | 0.38 | 0.35 | 0.38 | 0.4 | 0.38 | 0.34 | 0.36 | 0.38 |
| 13.25 | 0.34 | 0.3 | 0.35 | 0.39 | 0.33 | 0.41 | 0.43 | 0.37 | 0.34 | 0.34 | 0.37 |
| 13.5 | 0.39 | 0.31 | 0.32 | 0.39 | 0.34 | 0.38 | 0.42 | 0.37 | 0.33 | 0.34 | 0.35 |
| 13.75 | 0.37 | 0.3 | 0.3 | 0.42 | 0.33 | 0.38 | 0.36 | 0.36 | 0.32 | 0.33 | 0.33 |
| 14 | 0.36 | 0.33 | 0.3 | 0.44 | 0.29 | 0.36 | 0.36 | 0.34 | 0.31 | 0.29 | 0.35 |
| 14.25 | 0.33 | 0.31 | 0.31 | 0.43 | 0.28 | 0.34 | 0.35 | 0.34 | 0.29 | 0.28 | 0.32 |
| 14.5 | 0.38 | 0.34 | 0.27 | 0.48 | 0.26 | 0.35 | 0.36 | 0.31 | 0.31 | 0.27 | 0.33 |
| 14.75 | 0.34 | 0.31 | 0.26 | 0.47 | 0.26 | 0.31 | 0.34 | 0.34 | 0.3 | 0.28 | 0.31 |
| 15 | 0.36 | 0.33 | 0.26 | 0.46 | 0.26 | 0.3 | 0.31 | 0.31 | 0.32 | 0.27 | 0.28 |
| 15.25 | 0.33 | 0.3 | 0.26 | 0.43 | 0.25 | 0.3 | 0.31 | 0.29 | 0.31 | 0.25 | 0.28 |
| 15.5 | 0.32 | 0.3 | 0.26 | 0.42 | 0.24 | 0.29 | 0.29 | 0.29 | 0.29 | 0.26 | 0.27 |
| 15.75 | 0.34 | 0.29 | 0.23 | 0.38 | 0.22 | 0.27 | 0.27 | 0.28 | 0.28 | 0.23 | 0.27 |
| 16 | 0.31 | 0.29 | 0.23 | 0.38 | 0.21 | 0.27 | 0.25 | 0.26 | 0.28 | 0.22 | 0.24 |
| 16.25 | 0.3 | 0.28 | 0.23 | 0.35 | 0.2 | 0.27 | 0.26 | 0.26 | 0.25 | 0.22 | 0.22 |

| | | | | | | | | | | | |
|---|---|---|---|---|---|---|---|---|---|---|---|
| 16.5 | 0.26 | 0.26 | 0.23 | 0.32 | 0.21 | 0.26 | 0.25 | 0.24 | 0.27 | 0.21 | 0.22 |
| 16.75 | 0.27 | 0.25 | 0.22 | 0.3 | 0.19 | 0.23 | 0.23 | 0.23 | 0.22 | 0.2 | 0.21 |
| 17 | 0.26 | 0.23 | 0.2 | 0.29 | 0.19 | 0.22 | 0.21 | 0.22 | 0.22 | 0.19 | 0.2 |
| 17.25 | 0.26 | 0.21 | 0.21 | 0.27 | 0.17 | 0.23 | 0.19 | 0.22 | 0.21 | 0.18 | 0.19 |
| 17.5 | 0.25 | 0.23 | 0.18 | 0.25 | 0.18 | 0.21 | 0.2 | 0.21 | 0.21 | 0.17 | 0.2 |
| 17.75 | 0.25 | 0.21 | 0.18 | 0.24 | 0.16 | 0.2 | 0.19 | 0.2 | 0.18 | 0.17 | 0.18 |
| 18 | 0.2 | 0.2 | 0.18 | 0.22 | 0.16 | 0.19 | 0.18 | 0.21 | 0.19 | 0.16 | 0.17 |
| 18.25 | 0.22 | 0.2 | 0.18 | 0.21 | 0.15 | 0.2 | 0.17 | 0.18 | 0.18 | 0.16 | 0.17 |
| 18.5 | 0.2 | 0.2 | 0.16 | 0.2 | 0.15 | 0.18 | 0.17 | 0.18 | 0.18 | 0.15 | 0.16 |
| 18.75 | 0.19 | 0.19 | 0.16 | 0.19 | 0.14 | 0.18 | 0.15 | 0.17 | 0.18 | 0.14 | 0.16 |
| 19 | 0.16 | 0.18 | 0.17 | 0.18 | 0.14 | 0.18 | 0.16 | 0.17 | 0.17 | 0.14 | 0.15 |
| 19.25 | 0.17 | 0.17 | 0.15 | 0.18 | 0.13 | 0.16 | 0.15 | 0.16 | 0.16 | 0.13 | 0.14 |
| 19.5 | 0.18 | 0.17 | 0.15 | 0.18 | 0.12 | 0.15 | 0.14 | 0.16 | 0.16 | 0.13 | 0.14 |
| 19.75 | 0.16 | 0.17 | 0.14 | 0.17 | 0.12 | 0.14 | 0.14 | 0.16 | 0.16 | 0.12 | 0.14 |
| 20 | 0.14 | 0.16 | 0.13 | 0.16 | 0.12 | 0.14 | 0.13 | 0.15 | 0.16 | 0.12 | 0.14 |

| **TRF** | | | | | | | | | | | | | |
|---|---|---|---|---|---|---|---|---|---|---|---|---|---|
| Hz | | | | | | | | | | | | | |
| 0.25 | 1.19 | 1.03 | 1.58 | 1.31 | 2.22 | 7.87 | 2 | 6.04 | 4.06 | 9.56 | 1.98 | 3.54 | 1.54 |
| 0.5 | 1.35 | 1.29 | 1.82 | 1.87 | 1.95 | 2.62 | 1.98 | 2.36 | 2.15 | 2.94 | 1.46 | 1.94 | 1.5 |
| 0.75 | 1.3 | 1.34 | 1.71 | 1.74 | 1.86 | 1.83 | 1.73 | 1.81 | 1.87 | 1.67 | 1.26 | 1.41 | 1.32 |
| 1 | 1.17 | 1.28 | 1.7 | 1.57 | 1.71 | 1.63 | 1.64 | 1.59 | 1.68 | 1.38 | 1.22 | 1.42 | 1.06 |
| 1.25 | 1.18 | 1.16 | 1.79 | 1.5 | 1.57 | 1.58 | 1.55 | 1.51 | 1.6 | 1.27 | 1.26 | 1.4 | 1.08 |
| 1.5 | 1.26 | 1.15 | 1.55 | 1.5 | 1.57 | 1.49 | 1.4 | 1.48 | 1.64 | 1.19 | 1.38 | 1.38 | 1.05 |
| 1.75 | 1.27 | 1.12 | 1.76 | 1.4 | 1.63 | 1.9 | 1.5 | 1.56 | 1.79 | 1.21 | 1.42 | 1.39 | 1.14 |
| 2 | 1.3 | 1.18 | 1.79 | 1.48 | 1.78 | 1.78 | 1.6 | 1.61 | 1.71 | 1.27 | 1.57 | 1.48 | 1.13 |
| 2.25 | 1.52 | 1.17 | 1.77 | 1.52 | 1.72 | 1.75 | 1.69 | 1.62 | 1.76 | 1.26 | 1.71 | 1.53 | 1.41 |
| 2.5 | 1.55 | 1.27 | 1.94 | 1.59 | 1.75 | 1.78 | 1.78 | 1.6 | 1.76 | 1.21 | 1.68 | 1.5 | 1.58 |
| 2.75 | 1.65 | 1.22 | 1.73 | 1.54 | 1.65 | 1.77 | 1.85 | 1.59 | 1.93 | 1.21 | 1.63 | 1.42 | 1.92 |
| 3 | 1.4 | 1.28 | 1.69 | 1.43 | 1.6 | 1.59 | 1.87 | 1.57 | 1.93 | 1.2 | 1.55 | 1.38 | 1.93 |
| 3.25 | 1.22 | 1.17 | 1.49 | 1.31 | 1.47 | 1.57 | 1.66 | 1.44 | 1.83 | 1.06 | 1.52 | 1.35 | 2.19 |
| 3.5 | 1.09 | 1.12 | 1.41 | 1.2 | 1.29 | 1.59 | 1.53 | 1.38 | 1.57 | 1.04 | 1.41 | 1.31 | 2.38 |
| 3.75 | 1 | 1.15 | 1.27 | 1.26 | 1.27 | 1.42 | 1.58 | 1.25 | 1.74 | 0.97 | 1.39 | 1.27 | 2.31 |
| 4 | 1 | 1.12 | 1.17 | 1.18 | 1.28 | 1.31 | 1.51 | 1.27 | 1.59 | 0.92 | 1.39 | 1.33 | 2.46 |
| 4.25 | 0.96 | 1.18 | 1.26 | 1.29 | 1.23 | 1.48 | 1.5 | 1.24 | 1.54 | 1.02 | 1.39 | 1.42 | 2.37 |
| 4.5 | 1.02 | 1.29 | 1.29 | 1.35 | 1.31 | 1.41 | 1.47 | 1.38 | 1.5 | 0.87 | 1.39 | 1.42 | 1.92 |
| 4.75 | 0.99 | 1.33 | 1.29 | 1.35 | 1.29 | 1.36 | 1.36 | 1.25 | 1.38 | 0.94 | 1.35 | 1.35 | 1.64 |
| 5 | 1.17 | 1.47 | 1.29 | 1.45 | 1.38 | 1.23 | 1.41 | 1.32 | 1.19 | 0.82 | 1.36 | 1.39 | 1.39 |
| 5.25 | 1.21 | 1.7 | 1.35 | 1.55 | 1.34 | 1.4 | 1.35 | 1.37 | 1.15 | 0.96 | 1.35 | 1.47 | 1.21 |
| 5.5 | 1.46 | 2.14 | 1.5 | 1.82 | 1.69 | 1.47 | 1.36 | 1.45 | 1.3 | 0.93 | 1.35 | 1.53 | 1.23 |
| 5.75 | 1.7 | 2.54 | 1.75 | 2.27 | 1.98 | 1.36 | 1.43 | 1.59 | 1.19 | 1.02 | 1.48 | 1.57 | 1.44 |
| 6 | 2.18 | 3.3 | 2.54 | 2.85 | 2.46 | 2.16 | 1.43 | 1.92 | 1.42 | 1.25 | 1.66 | 1.95 | 1.62 |
| 6.25 | 2.82 | 3.91 | 3.09 | 3.62 | 3.21 | 2.33 | 1.56 | 2.47 | 1.65 | 1.45 | 1.97 | 2.24 | 1.84 |
| 6.5 | 3.38 | 4.4 | 4 | 4.54 | 4.29 | 3.1 | 1.79 | 2.87 | 1.63 | 2.01 | 2.42 | 2.6 | 2.71 |
| 6.75 | 3.81 | 5.39 | 4.26 | 5.32 | 4.6 | 4.15 | 1.93 | 3.24 | 2.4 | 2.39 | 2.92 | 3.09 | 3.49 |
| 7 | 4.26 | 4.9 | 5.05 | 5.13 | 5.23 | 3.9 | 2.06 | 3.57 | 2.93 | 2.7 | 3.41 | 3.39 | 4.11 |
| 7.25 | 4.49 | 5.28 | 4.78 | 4.66 | 5.09 | 4.54 | 2.4 | 4.28 | 3.57 | 3.62 | 4.01 | 3.76 | 4.26 |

| | | | | | | | | | | | | | |
|---|---|---|---|---|---|---|---|---|---|---|---|---|---|
| 7.5 | 4.09 | 4.29 | 4.28 | 4.21 | 4.26 | 3.62 | 2.53 | 4.12 | 4.25 | 3.41 | 4.55 | 4.06 | 4.06 |
| 7.75 | 3.6 | 4.15 | 3.68 | 3.53 | 3.63 | 3.31 | 2.2 | 4.18 | 4.19 | 3.56 | 4.28 | 4.02 | 3.22 |
| 8 | 2.96 | 3.44 | 2.82 | 3.02 | 2.8 | 2.36 | 1.91 | 3.2 | 3.83 | 2.79 | 4.03 | 3.88 | 2.96 |
| 8.25 | 2.56 | 2.8 | 2.51 | 2.58 | 2.49 | 1.82 | 1.92 | 2.71 | 3.42 | 2.18 | 3.44 | 3.46 | 2.3 |
| 8.5 | 2.24 | 2.39 | 2.22 | 2.1 | 2.02 | 1.56 | 1.57 | 2.27 | 2.49 | 1.83 | 2.86 | 3.06 | 1.8 |
| 8.75 | 1.93 | 1.99 | 1.7 | 1.77 | 1.67 | 1.44 | 1.32 | 1.81 | 2.22 | 1.7 | 2.34 | 2.44 | 1.67 |
| 9 | 1.69 | 1.73 | 1.56 | 1.48 | 1.37 | 1.14 | 1.18 | 1.6 | 1.86 | 1.37 | 1.91 | 2.21 | 1.39 |
| 9.25 | 1.38 | 1.43 | 1.23 | 1.18 | 1.23 | 0.89 | 0.97 | 1.2 | 1.5 | 1.09 | 1.65 | 1.92 | 1.17 |
| 9.5 | 1.25 | 1.32 | 1.15 | 1.07 | 1.01 | 0.76 | 0.87 | 1.14 | 1.2 | 0.89 | 1.42 | 1.61 | 1.05 |
| 9.75 | 1.09 | 1.17 | 0.95 | 0.96 | 0.88 | 0.67 | 0.82 | 0.98 | 0.98 | 0.83 | 1.25 | 1.37 | 0.93 |
| 10 | 0.93 | 1.01 | 0.87 | 0.82 | 0.76 | 0.63 | 0.71 | 0.9 | 0.92 | 0.76 | 1.07 | 1.18 | 0.89 |
| 10.25 | 0.82 | 1 | 0.75 | 0.79 | 0.69 | 0.53 | 0.67 | 0.82 | 0.77 | 0.64 | 0.98 | 1.06 | 0.75 |
| 10.5 | 0.78 | 0.88 | 0.71 | 0.69 | 0.61 | 0.51 | 0.59 | 0.7 | 0.69 | 0.64 | 0.85 | 0.92 | 0.69 |
| 10.75 | 0.69 | 0.72 | 0.64 | 0.63 | 0.55 | 0.5 | 0.56 | 0.67 | 0.62 | 0.58 | 0.77 | 0.86 | 0.61 |
| 11 | 0.64 | 0.66 | 0.56 | 0.56 | 0.53 | 0.48 | 0.5 | 0.56 | 0.59 | 0.52 | 0.71 | 0.76 | 0.54 |
| 11.25 | 0.57 | 0.61 | 0.52 | 0.54 | 0.48 | 0.48 | 0.49 | 0.57 | 0.52 | 0.46 | 0.65 | 0.67 | 0.53 |
| 11.5 | 0.53 | 0.55 | 0.46 | 0.49 | 0.43 | 0.44 | 0.48 | 0.5 | 0.47 | 0.47 | 0.59 | 0.58 | 0.45 |
| 11.75 | 0.49 | 0.53 | 0.47 | 0.46 | 0.41 | 0.38 | 0.44 | 0.47 | 0.43 | 0.38 | 0.58 | 0.54 | 0.44 |
| 12 | 0.46 | 0.46 | 0.46 | 0.39 | 0.37 | 0.36 | 0.41 | 0.47 | 0.38 | 0.4 | 0.52 | 0.53 | 0.41 |
| 12.25 | 0.42 | 0.47 | 0.41 | 0.38 | 0.37 | 0.35 | 0.43 | 0.43 | 0.34 | 0.36 | 0.46 | 0.5 | 0.36 |
| 12.5 | 0.41 | 0.4 | 0.37 | 0.36 | 0.36 | 0.35 | 0.39 | 0.38 | 0.37 | 0.34 | 0.46 | 0.44 | 0.35 |
| 12.75 | 0.37 | 0.38 | 0.37 | 0.34 | 0.34 | 0.36 | 0.37 | 0.36 | 0.34 | 0.33 | 0.45 | 0.41 | 0.34 |
| 13 | 0.39 | 0.34 | 0.35 | 0.33 | 0.33 | 0.37 | 0.36 | 0.37 | 0.39 | 0.31 | 0.41 | 0.4 | 0.32 |
| 13.25 | 0.34 | 0.33 | 0.34 | 0.31 | 0.31 | 0.37 | 0.34 | 0.33 | 0.36 | 0.32 | 0.4 | 0.35 | 0.3 |
| 13.5 | 0.31 | 0.31 | 0.35 | 0.3 | 0.3 | 0.42 | 0.35 | 0.34 | 0.28 | 0.3 | 0.39 | 0.34 | 0.32 |
| 13.75 | 0.32 | 0.29 | 0.32 | 0.3 | 0.29 | 0.44 | 0.34 | 0.31 | 0.31 | 0.31 | 0.37 | 0.34 | 0.28 |
| 14 | 0.32 | 0.29 | 0.32 | 0.3 | 0.29 | 0.37 | 0.31 | 0.33 | 0.27 | 0.31 | 0.37 | 0.31 | 0.32 |
| 14.25 | 0.3 | 0.27 | 0.29 | 0.27 | 0.29 | 0.32 | 0.31 | 0.35 | 0.33 | 0.26 | 0.37 | 0.31 | 0.29 |
| 14.5 | 0.28 | 0.27 | 0.27 | 0.28 | 0.29 | 0.37 | 0.32 | 0.32 | 0.28 | 0.27 | 0.37 | 0.29 | 0.3 |
| 14.75 | 0.28 | 0.27 | 0.27 | 0.26 | 0.26 | 0.38 | 0.32 | 0.32 | 0.32 | 0.26 | 0.34 | 0.27 | 0.29 |
| 15 | 0.28 | 0.25 | 0.29 | 0.25 | 0.27 | 0.33 | 0.31 | 0.3 | 0.31 | 0.25 | 0.33 | 0.26 | 0.28 |
| 15.25 | 0.26 | 0.23 | 0.27 | 0.25 | 0.26 | 0.34 | 0.29 | 0.3 | 0.32 | 0.25 | 0.35 | 0.27 | 0.27 |
| 15.5 | 0.24 | 0.23 | 0.25 | 0.24 | 0.25 | 0.3 | 0.28 | 0.29 | 0.3 | 0.26 | 0.32 | 0.26 | 0.25 |
| 15.75 | 0.25 | 0.2 | 0.26 | 0.22 | 0.23 | 0.31 | 0.28 | 0.28 | 0.32 | 0.25 | 0.3 | 0.25 | 0.25 |
| 16 | 0.25 | 0.2 | 0.24 | 0.21 | 0.23 | 0.29 | 0.27 | 0.27 | 0.3 | 0.23 | 0.31 | 0.24 | 0.24 |
| 16.25 | 0.24 | 0.2 | 0.23 | 0.21 | 0.2 | 0.26 | 0.26 | 0.25 | 0.28 | 0.23 | 0.3 | 0.22 | 0.23 |
| 16.5 | 0.22 | 0.18 | 0.21 | 0.19 | 0.2 | 0.25 | 0.25 | 0.26 | 0.28 | 0.23 | 0.28 | 0.22 | 0.21 |
| 16.75 | 0.21 | 0.18 | 0.22 | 0.2 | 0.2 | 0.23 | 0.26 | 0.24 | 0.32 | 0.22 | 0.27 | 0.21 | 0.2 |
| 17 | 0.21 | 0.17 | 0.2 | 0.19 | 0.19 | 0.23 | 0.25 | 0.24 | 0.24 | 0.2 | 0.27 | 0.21 | 0.2 |
| 17.25 | 0.21 | 0.16 | 0.19 | 0.19 | 0.19 | 0.22 | 0.25 | 0.22 | 0.27 | 0.19 | 0.25 | 0.2 | 0.2 |
| 17.5 | 0.2 | 0.16 | 0.19 | 0.17 | 0.19 | 0.2 | 0.24 | 0.22 | 0.27 | 0.19 | 0.25 | 0.19 | 0.19 |
| 17.75 | 0.19 | 0.15 | 0.18 | 0.17 | 0.18 | 0.21 | 0.23 | 0.2 | 0.26 | 0.19 | 0.23 | 0.19 | 0.19 |
| 18 | 0.19 | 0.15 | 0.18 | 0.16 | 0.17 | 0.21 | 0.23 | 0.19 | 0.26 | 0.17 | 0.22 | 0.18 | 0.19 |
| 18.25 | 0.18 | 0.13 | 0.17 | 0.15 | 0.16 | 0.2 | 0.22 | 0.18 | 0.23 | 0.17 | 0.22 | 0.16 | 0.18 |
| 18.5 | 0.16 | 0.13 | 0.17 | 0.15 | 0.16 | 0.18 | 0.21 | 0.18 | 0.2 | 0.18 | 0.21 | 0.17 | 0.17 |
| 18.75 | 0.17 | 0.13 | 0.16 | 0.15 | 0.16 | 0.19 | 0.21 | 0.17 | 0.21 | 0.17 | 0.21 | 0.17 | 0.16 |
| 19 | 0.16 | 0.12 | 0.15 | 0.15 | 0.14 | 0.18 | 0.2 | 0.16 | 0.21 | 0.15 | 0.19 | 0.15 | 0.16 |
| 19.25 | 0.15 | 0.12 | 0.15 | 0.14 | 0.14 | 0.18 | 0.19 | 0.16 | 0.19 | 0.15 | 0.19 | 0.14 | 0.15 |

| | | | | | | | | | | | | | |
|---|---|---|---|---|---|---|---|---|---|---|---|---|---|
| 19.5 | 0.15 | 0.12 | 0.15 | 0.13 | 0.13 | 0.14 | 0.19 | 0.15 | 0.18 | 0.15 | 0.19 | 0.14 | 0.16 |
| 19.75 | 0.14 | 0.11 | 0.14 | 0.13 | 0.14 | 0.16 | 0.19 | 0.14 | 0.15 | 0.14 | 0.18 | 0.14 | 0.15 |
| 20 | 0.15 | 0.11 | 0.13 | 0.12 | 0.13 | 0.16 | 0.17 | 0.15 | 0.16 | 0.14 | 0.17 | 0.13 | 0.13 |

| **TRM** | | | | | | | | | | | | | | | |
|---|---|---|---|---|---|---|---|---|---|---|---|---|---|---|---|
| Hz | | | | | | | | | | | | | | | |
| 0.25 | 3.21 | 1.61 | 1.41 | 1.61 | 0.96 | 1.89 | 1.67 | 3.1 | 1.68 | 1.46 | 1.43 | 0.91 | 1.21 | 1.83 | 0.92 |
| 0.5 | 2.25 | 1.82 | 2.02 | 2.11 | 1.28 | 2.58 | 1.87 | 2 | 1.84 | 1.91 | 1.42 | 1.44 | 1.65 | 2.06 | 1.36 |
| 0.75 | 1.94 | 1.7 | 1.92 | 2.25 | 1.24 | 1.97 | 1.8 | 1.49 | 1.56 | 1.89 | 1.38 | 1.52 | 1.52 | 2.02 | 1.29 |
| 1 | 1.73 | 1.72 | 1.67 | 1.91 | 1.13 | 1.6 | 1.62 | 1.32 | 1.48 | 1.63 | 1.28 | 1.46 | 1.49 | 1.62 | 1.18 |
| 1.25 | 1.51 | 1.62 | 1.5 | 1.5 | 1.03 | 1.54 | 1.59 | 1.14 | 1.4 | 1.5 | 1.15 | 1.34 | 1.41 | 1.55 | 1.17 |
| 1.5 | 1.51 | 1.73 | 1.44 | 1.59 | 1.02 | 1.62 | 1.52 | 1.19 | 1.48 | 1.51 | 1.14 | 1.32 | 1.38 | 1.52 | 1.18 |
| 1.75 | 1.63 | 1.72 | 1.45 | 1.65 | 1.11 | 1.61 | 1.64 | 1.18 | 1.46 | 1.61 | 1.12 | 1.37 | 1.44 | 1.61 | 1.17 |
| 2 | 1.66 | 1.94 | 1.7 | 1.6 | 1.13 | 1.58 | 1.59 | 1.29 | 1.52 | 1.63 | 1.2 | 1.35 | 1.53 | 1.53 | 1.28 |
| 2.25 | 1.65 | 2.09 | 1.67 | 1.7 | 1.37 | 1.64 | 1.79 | 1.41 | 1.64 | 1.88 | 1.33 | 1.39 | 1.66 | 1.7 | 1.31 |
| 2.5 | 1.66 | 2.19 | 1.66 | 1.62 | 1.75 | 1.5 | 1.81 | 1.59 | 1.61 | 1.87 | 1.47 | 1.16 | 1.56 | 1.6 | 1.33 |
| 2.75 | 1.72 | 2.19 | 1.61 | 1.66 | 2.23 | 1.55 | 1.86 | 1.67 | 1.64 | 1.89 | 1.46 | 1.23 | 1.6 | 1.66 | 1.35 |
| 3 | 1.74 | 2.13 | 1.51 | 1.78 | 2.46 | 1.52 | 1.77 | 1.68 | 1.59 | 1.74 | 1.4 | 1.16 | 1.39 | 1.56 | 1.22 |
| 3.25 | 1.59 | 1.99 | 1.45 | 1.63 | 2.16 | 1.57 | 1.71 | 1.59 | 1.48 | 1.62 | 1.26 | 1.15 | 1.38 | 1.37 | 1.21 |
| 3.5 | 1.54 | 1.99 | 1.34 | 1.61 | 1.71 | 1.56 | 1.56 | 1.4 | 1.41 | 1.52 | 1.19 | 1.15 | 1.43 | 1.38 | 1.16 |
| 3.75 | 1.62 | 1.81 | 1.23 | 1.59 | 1.3 | 1.47 | 1.49 | 1.2 | 1.39 | 1.45 | 1.08 | 1.14 | 1.37 | 1.29 | 1.06 |
| 4 | 1.49 | 1.72 | 1.11 | 1.49 | 1.16 | 1.37 | 1.47 | 1.13 | 1.35 | 1.41 | 1 | 1.04 | 1.34 | 1.17 | 1.1 |
| 4.25 | 1.58 | 1.61 | 1.1 | 1.42 | 1.04 | 1.29 | 1.45 | 1.11 | 1.29 | 1.51 | 1.01 | 1.19 | 1.26 | 1.16 | 1.15 |
| 4.5 | 1.51 | 1.62 | 1.16 | 1.25 | 1.09 | 1.26 | 1.51 | 1.13 | 1.35 | 1.48 | 1.1 | 1.24 | 1.36 | 1.08 | 1.23 |
| 4.75 | 1.51 | 1.49 | 1.13 | 1.17 | 1.06 | 1.26 | 1.39 | 1.03 | 1.25 | 1.5 | 1.11 | 1.31 | 1.42 | 1.11 | 1.34 |
| 5 | 1.41 | 1.46 | 1.11 | 1.18 | 1.2 | 1.34 | 1.32 | 1.02 | 1.41 | 1.58 | 1.2 | 1.52 | 1.39 | 0.94 | 1.38 |
| 5.25 | 1.5 | 1.52 | 1.21 | 1.21 | 1.35 | 1.18 | 1.35 | 1.07 | 1.44 | 1.59 | 1.22 | 1.74 | 1.48 | 1.08 | 1.6 |
| 5.5 | 1.43 | 1.61 | 1.43 | 1.28 | 1.63 | 1.28 | 1.42 | 1.21 | 1.6 | 1.69 | 1.31 | 2 | 1.62 | 1.24 | 1.87 |
| 5.75 | 1.68 | 1.69 | 1.61 | 1.4 | 2.11 | 1.25 | 1.49 | 1.34 | 1.83 | 1.8 | 1.57 | 2.54 | 1.9 | 1.36 | 2.28 |
| 6 | 1.95 | 1.91 | 2.18 | 1.65 | 2.64 | 1.63 | 1.72 | 1.64 | 2.33 | 2.4 | 1.98 | 3.44 | 2.53 | 1.73 | 2.95 |
| 6.25 | 2.14 | 2.03 | 2.72 | 2.06 | 3.42 | 1.8 | 2.18 | 1.94 | 2.63 | 3.08 | 2.33 | 4.03 | 2.83 | 1.97 | 3.54 |
| 6.5 | 2.74 | 2.04 | 3.42 | 2.85 | 4.31 | 2.3 | 2.31 | 2.35 | 3.08 | 3.22 | 2.77 | 4.76 | 3.54 | 2.67 | 4.12 |
| 6.75 | 3.04 | 2.58 | 4.06 | 3.15 | 4.74 | 2.75 | 2.59 | 2.65 | 3.59 | 4.15 | 3.5 | 5.66 | 4.16 | 3.55 | 4.54 |
| 7 | 3.65 | 2.8 | 4.79 | 4.25 | 4.83 | 3.32 | 3.26 | 3.16 | 4.18 | 4.38 | 3.92 | 5.61 | 4.85 | 4.43 | 4.89 |
| 7.25 | 4.11 | 3.21 | 5.08 | 4.59 | 5.03 | 4.12 | 3.74 | 3.64 | 4.52 | 4.38 | 4.41 | 5.28 | 4.89 | 5.4 | 5.18 |
| 7.5 | 4.2 | 3.47 | 4.9 | 5.02 | 4.67 | 4.07 | 4.09 | 4.15 | 4.8 | 3.82 | 4.11 | 4.5 | 4.68 | 5.19 | 4.85 |
| 7.75 | 3.82 | 3.61 | 4.4 | 4.44 | 4.15 | 4.3 | 4.08 | 4.22 | 4.22 | 3.23 | 3.64 | 3.69 | 3.85 | 5.07 | 4.31 |
| 8 | 3.51 | 3.26 | 3.8 | 3.76 | 3.6 | 3.7 | 3.74 | 3.81 | 3.44 | 2.94 | 3.2 | 3.17 | 3.18 | 4.06 | 3.68 |
| 8.25 | 2.82 | 3.05 | 2.88 | 3.26 | 2.85 | 3.57 | 3.54 | 3.44 | 2.91 | 2.38 | 2.69 | 2.48 | 2.56 | 3.17 | 2.98 |
| 8.5 | 2.46 | 2.46 | 2.46 | 2.68 | 2.46 | 2.61 | 2.86 | 2.8 | 2.35 | 1.99 | 2.17 | 2.09 | 2.19 | 2.43 | 2.46 |
| 8.75 | 1.84 | 2.01 | 1.97 | 1.98 | 2.05 | 2.11 | 2.35 | 2.23 | 2.08 | 1.56 | 1.8 | 1.72 | 1.84 | 1.92 | 2.13 |
| 9 | 1.48 | 1.69 | 1.75 | 1.77 | 1.79 | 1.63 | 1.96 | 1.95 | 1.77 | 1.33 | 1.52 | 1.55 | 1.48 | 1.57 | 1.8 |
| 9.25 | 1.23 | 1.39 | 1.37 | 1.24 | 1.52 | 1.3 | 1.58 | 1.54 | 1.38 | 1.12 | 1.23 | 1.24 | 1.3 | 1.33 | 1.52 |
| 9.5 | 1.12 | 1.19 | 1.24 | 1.09 | 1.3 | 1.16 | 1.4 | 1.38 | 1.28 | 1.01 | 1.07 | 1.16 | 1.18 | 1.15 | 1.26 |
| 9.75 | 0.93 | 1 | 0.97 | 0.96 | 1.07 | 1.04 | 1.16 | 1.16 | 1.06 | 0.89 | 0.93 | 0.97 | 1.05 | 0.98 | 1.14 |

| | | | | | | | | | | | | | | | |
|---|---|---|---|---|---|---|---|---|---|---|---|---|---|---|---|
| 10 | 0.8 | 0.91 | 0.89 | 0.79 | 0.94 | 0.98 | 1 | 1.01 | 0.96 | 0.86 | 0.83 | 0.82 | 0.97 | 0.83 | 1 |
| 10.25 | 0.73 | 0.84 | 0.81 | 0.73 | 0.83 | 0.82 | 0.91 | 0.85 | 0.8 | 0.72 | 0.79 | 0.75 | 0.87 | 0.73 | 0.88 |
| 10.5 | 0.65 | 0.73 | 0.68 | 0.66 | 0.72 | 0.72 | 0.79 | 0.77 | 0.68 | 0.69 | 0.69 | 0.65 | 0.81 | 0.65 | 0.77 |
| 10.75 | 0.6 | 0.68 | 0.63 | 0.6 | 0.66 | 0.65 | 0.67 | 0.67 | 0.64 | 0.64 | 0.6 | 0.61 | 0.75 | 0.55 | 0.68 |
| 11 | 0.56 | 0.65 | 0.58 | 0.55 | 0.59 | 0.59 | 0.61 | 0.6 | 0.57 | 0.57 | 0.57 | 0.55 | 0.7 | 0.55 | 0.64 |
| 11.25 | 0.53 | 0.57 | 0.51 | 0.52 | 0.55 | 0.52 | 0.56 | 0.54 | 0.52 | 0.52 | 0.49 | 0.48 | 0.58 | 0.46 | 0.59 |
| 11.5 | 0.52 | 0.56 | 0.48 | 0.45 | 0.48 | 0.51 | 0.51 | 0.51 | 0.48 | 0.48 | 0.45 | 0.46 | 0.56 | 0.42 | 0.52 |
| 11.75 | 0.48 | 0.51 | 0.43 | 0.43 | 0.44 | 0.44 | 0.46 | 0.47 | 0.47 | 0.51 | 0.43 | 0.44 | 0.53 | 0.4 | 0.48 |
| 12 | 0.46 | 0.51 | 0.42 | 0.42 | 0.4 | 0.45 | 0.48 | 0.47 | 0.42 | 0.44 | 0.39 | 0.41 | 0.5 | 0.37 | 0.46 |
| 12.25 | 0.42 | 0.47 | 0.38 | 0.4 | 0.39 | 0.38 | 0.43 | 0.42 | 0.41 | 0.42 | 0.37 | 0.36 | 0.45 | 0.38 | 0.42 |
| 12.5 | 0.41 | 0.44 | 0.36 | 0.37 | 0.35 | 0.41 | 0.39 | 0.38 | 0.37 | 0.43 | 0.36 | 0.34 | 0.44 | 0.34 | 0.38 |
| 12.75 | 0.39 | 0.41 | 0.33 | 0.38 | 0.33 | 0.38 | 0.4 | 0.36 | 0.37 | 0.43 | 0.34 | 0.32 | 0.4 | 0.32 | 0.37 |
| 13 | 0.34 | 0.41 | 0.33 | 0.35 | 0.3 | 0.37 | 0.38 | 0.36 | 0.34 | 0.4 | 0.33 | 0.3 | 0.39 | 0.32 | 0.34 |
| 13.25 | 0.34 | 0.41 | 0.33 | 0.34 | 0.29 | 0.38 | 0.34 | 0.33 | 0.37 | 0.39 | 0.32 | 0.3 | 0.39 | 0.3 | 0.34 |
| 13.5 | 0.36 | 0.38 | 0.33 | 0.33 | 0.29 | 0.4 | 0.37 | 0.32 | 0.33 | 0.38 | 0.3 | 0.3 | 0.36 | 0.29 | 0.31 |
| 13.75 | 0.35 | 0.38 | 0.31 | 0.32 | 0.28 | 0.38 | 0.35 | 0.31 | 0.3 | 0.36 | 0.29 | 0.29 | 0.36 | 0.3 | 0.32 |
| 14 | 0.33 | 0.38 | 0.32 | 0.32 | 0.28 | 0.35 | 0.34 | 0.31 | 0.34 | 0.37 | 0.29 | 0.28 | 0.36 | 0.3 | 0.3 |
| 14.25 | 0.36 | 0.36 | 0.3 | 0.32 | 0.24 | 0.37 | 0.35 | 0.29 | 0.34 | 0.36 | 0.28 | 0.26 | 0.34 | 0.3 | 0.31 |
| 14.5 | 0.32 | 0.36 | 0.3 | 0.3 | 0.24 | 0.39 | 0.31 | 0.28 | 0.32 | 0.36 | 0.28 | 0.27 | 0.33 | 0.3 | 0.3 |
| 14.75 | 0.32 | 0.33 | 0.3 | 0.29 | 0.23 | 0.35 | 0.32 | 0.3 | 0.32 | 0.34 | 0.28 | 0.24 | 0.33 | 0.3 | 0.3 |
| 15 | 0.3 | 0.33 | 0.29 | 0.29 | 0.23 | 0.39 | 0.32 | 0.29 | 0.31 | 0.34 | 0.26 | 0.24 | 0.31 | 0.28 | 0.28 |
| 15.25 | 0.29 | 0.33 | 0.27 | 0.27 | 0.21 | 0.39 | 0.32 | 0.29 | 0.29 | 0.32 | 0.25 | 0.23 | 0.28 | 0.28 | 0.26 |
| 15.5 | 0.3 | 0.33 | 0.29 | 0.25 | 0.22 | 0.33 | 0.28 | 0.29 | 0.3 | 0.3 | 0.25 | 0.23 | 0.28 | 0.27 | 0.25 |
| 15.75 | 0.3 | 0.32 | 0.26 | 0.27 | 0.2 | 0.34 | 0.31 | 0.28 | 0.28 | 0.27 | 0.25 | 0.21 | 0.28 | 0.29 | 0.25 |
| 16 | 0.27 | 0.32 | 0.26 | 0.28 | 0.19 | 0.32 | 0.27 | 0.27 | 0.26 | 0.27 | 0.23 | 0.2 | 0.25 | 0.24 | 0.24 |
| 16.25 | 0.26 | 0.3 | 0.24 | 0.25 | 0.18 | 0.35 | 0.26 | 0.26 | 0.26 | 0.26 | 0.23 | 0.18 | 0.24 | 0.23 | 0.23 |
| 16.5 | 0.26 | 0.29 | 0.23 | 0.24 | 0.16 | 0.29 | 0.26 | 0.25 | 0.25 | 0.25 | 0.21 | 0.18 | 0.23 | 0.25 | 0.22 |
| 16.75 | 0.24 | 0.28 | 0.21 | 0.24 | 0.17 | 0.32 | 0.25 | 0.25 | 0.24 | 0.25 | 0.21 | 0.18 | 0.22 | 0.24 | 0.2 |
| 17 | 0.25 | 0.27 | 0.22 | 0.23 | 0.17 | 0.3 | 0.25 | 0.25 | 0.23 | 0.25 | 0.21 | 0.17 | 0.21 | 0.22 | 0.21 |
| 17.25 | 0.23 | 0.25 | 0.21 | 0.23 | 0.16 | 0.27 | 0.23 | 0.24 | 0.22 | 0.23 | 0.19 | 0.18 | 0.21 | 0.22 | 0.19 |
| 17.5 | 0.23 | 0.25 | 0.2 | 0.21 | 0.14 | 0.27 | 0.23 | 0.22 | 0.2 | 0.24 | 0.19 | 0.16 | 0.19 | 0.2 | 0.19 |
| 17.75 | 0.22 | 0.24 | 0.18 | 0.21 | 0.15 | 0.22 | 0.22 | 0.2 | 0.21 | 0.21 | 0.19 | 0.15 | 0.19 | 0.2 | 0.18 |
| 18 | 0.2 | 0.23 | 0.17 | 0.2 | 0.14 | 0.24 | 0.21 | 0.2 | 0.2 | 0.21 | 0.18 | 0.15 | 0.18 | 0.18 | 0.17 |
| 18.25 | 0.2 | 0.22 | 0.18 | 0.18 | 0.13 | 0.26 | 0.21 | 0.2 | 0.19 | 0.19 | 0.18 | 0.15 | 0.18 | 0.19 | 0.17 |
| 18.5 | 0.17 | 0.2 | 0.16 | 0.19 | 0.13 | 0.23 | 0.2 | 0.18 | 0.2 | 0.17 | 0.17 | 0.14 | 0.18 | 0.19 | 0.16 |
| 18.75 | 0.19 | 0.21 | 0.17 | 0.17 | 0.13 | 0.22 | 0.19 | 0.19 | 0.2 | 0.18 | 0.16 | 0.13 | 0.17 | 0.19 | 0.16 |
| 19 | 0.18 | 0.19 | 0.15 | 0.16 | 0.12 | 0.2 | 0.2 | 0.19 | 0.19 | 0.18 | 0.16 | 0.13 | 0.15 | 0.17 | 0.15 |
| 19.25 | 0.18 | 0.19 | 0.15 | 0.16 | 0.11 | 0.21 | 0.19 | 0.18 | 0.19 | 0.19 | 0.15 | 0.12 | 0.15 | 0.17 | 0.14 |
| 19.5 | 0.17 | 0.18 | 0.15 | 0.16 | 0.11 | 0.22 | 0.18 | 0.17 | 0.16 | 0.17 | 0.15 | 0.13 | 0.15 | 0.16 | 0.13 |
| 19.75 | 0.17 | 0.17 | 0.15 | 0.15 | 0.11 | 0.21 | 0.17 | 0.16 | 0.15 | 0.15 | 0.14 | 0.12 | 0.14 | 0.16 | 0.13 |
| 20 | 0.15 | 0.17 | 0.13 | 0.15 | 0.1 | 0.21 | 0.15 | 0.15 | 0.16 | 0.15 | 0.14 | 0.11 | 0.14 | 0.15 | 0.12 |

Individual median (range) of the EEG peak frequency during NREMS after 6 h of sleep deprivation

| CLF | CLM | TRF | TRM |
|---|---|---|---|
| 1.25 | 1.5 | 2.5 | 1 |
| 1.5 | 1 | 3 | 1.5 |

| | | | |
|---|---|---|---|
| 1.25 | 1.5 | 1.5 | 2.25 |
| 1.75 | 1.75 | 1.25 | 1.75 |
| 1 | 3 | 1.25 | 3.25 |
| 1 | 1 | 1.25 | 1.25 |
| 0.75 | 0.75 | 1.25 | 1.25 |
| 1.25 | 1 | 1.25 | 1 |
| 1 | 1 | 2.75 | 1.5 |
| 1.25 | 1 | 1.25 | 2.5 |
| 1 | 1.25 | 1.25 | 2.75 |
| 2.75 | | 1.5 | 1.25 |
| | | 2.75 | 1 |
| | | | 1 |
| | | | 1 |

Individual median (range) of the EEG peak frequency during REMS after 6 h of sleep deprivation

| CLF | CLM | TRF | TRM |
|---|---|---|---|
| 7.5 | 7.5 | 7.25 | 7.5 |
| 7.25 | 7.5 | 6.75 | 7.75 |
| 7.5 | 7.5 | 7 | 7.25 |
| 7.5 | 7 | 6.75 | 7.5 |
| 7.5 | 7.25 | 7 | 7.25 |
| 7.5 | 7 | 7.25 | 7.75 |
| 6 | 7.25 | 7.5 | 7.5 |
| 7.75 | 7.5 | 7.25 | 7.75 |
| 7.5 | 7.75 | 7.5 | 7.5 |
| 7.5 | 7.25 | 7.25 | 7 |
| 7.5 | 7.25 | 7.5 | 7.25 |
| 7.5 | | 7.5 | 6.75 |
| | | 7.25 | 7.25 |
| | | | 7.25 |
| | | | 7.25 |

Power in the delta frequency range during NREMS after 6 h of sleep deprivation

| **CLF** | | | | | | | | | | | | |
|---|---|---|---|---|---|---|---|---|---|---|---|---|
| ZT | | | | | | | | | | | | |
| 2 | | | | | | | | | | | | |
| 4 | | | | | | | | | | | | |
| 6 | | | | | | | | | | | | |
| 8 | 186 | 165.9 | 112.4 | 214.2 | 176.5 | 184.2 | *9999* | 163.6 | 161.3 | 136.9 | 176.7 | 150.5 |
| 10 | 137.9 | 127.6 | 85.87 | 147.6 | 126.5 | 130.2 | 89.2 | 121.1 | 124.7 | 102.6 | 132 | 115.5 |
| 12 | 120.6 | 111.3 | 82.86 | 118.1 | 108.3 | 118.1 | 88.71 | 113.2 | 124.1 | 96.25 | 118.7 | 101.7 |
| 14 | 121 | 114.2 | 93.95 | 123.4 | 109 | 108.3 | 70.68 | 103.6 | 101.8 | 85.97 | 128.8 | 103.2 |
| 16 | 104.8 | 125 | 85.88 | 108.6 | 108.4 | 110.6 | 71.43 | 109.1 | 107.8 | 93.62 | 99.53 | 102 |
| 18 | 127.5 | 126.4 | 85.85 | 111 | 111.4 | 144.2 | 85.96 | *9999* | 112.3 | 88.23 | 38.5 | 115.9 |
| 20 | 25.8 | 124.7 | 82.04 | 121.5 | 104.7 | 128.6 | 77.97 | *9999* | 92.33 | 86.3 | 135.8 | *9999* |

| | | | | | | | | | | | | |
|---|---|---|---|---|---|---|---|---|---|---|---|---|
| 22 | 143.1 | 115.2 | 93.63 | 115.3 | 132.1 | 115.3 | 50 | 116.8 | 104.9 | 106.8 | 122 | 128.1 |
| 24 | 127.5 | 124.2 | 82.08 | 131.9 | 121.7 | 89.42 | 49.35 | 119.7 | 99.03 | 86 | 101.7 | 113.8 |

| **CLM** | | | | | | | | | | | |
|---|---|---|---|---|---|---|---|---|---|---|---|
| ZT | | | | | | | | | | | |
| 2 | | | | | | | | | | | |
| 4 | | | | | | | | | | | |
| 6 | | | | | | | | | | | |
| 8 | 172.7 | 157.4 | 169.7 | 177.7 | 196.1 | 167 | 166.2 | 134.4 | 188.2 | 180 | 180.9 |
| 10 | 124.8 | 123.5 | 131.8 | 132.8 | 136.6 | 118.1 | 120.5 | 107.4 | 125.1 | 128.9 | 132.1 |
| 12 | 113.1 | 104.4 | 118.8 | 119.5 | 130.1 | 102.7 | 105.5 | 98.37 | 122.7 | 105.1 | 125.3 |
| 14 | 123 | 114.1 | 129.2 | 139.4 | 157.7 | 97.27 | 96.61 | 99.63 | 107.5 | 120.7 | 131.9 |
| 16 | 117.8 | 114.6 | 141.4 | 139 | 122.9 | 112.2 | 107 | 106.3 | 107.9 | 111 | 122.4 |
| 18 | 123.7 | 98.5 | 128.9 | 130.1 | 138.2 | 114.4 | 93.15 | 110.3 | 104 | 118.6 | 131.6 |
| 20 | 104.5 | 89.91 | 130.4 | 133.5 | 128 | 117.9 | 131 | 104.4 | 94.56 | 122.5 | 134.8 |
| 22 | 102.7 | 92.05 | 115.6 | 120.1 | 117.2 | 99.05 | 97.92 | 105.7 | 105.9 | 107.1 | 110.3 |
| 24 | 105.8 | 107.3 | 121 | 120.5 | 141.6 | 95.85 | 80.87 | 119.3 | 97.05 | 116.5 | 130.4 |

| **TRF** | | | | | | | | | | | | | |
|---|---|---|---|---|---|---|---|---|---|---|---|---|---|
| ZT | | | | | | | | | | | | | |
| 2 | | | | | | | | | | | | | |
| 4 | | | | | | | | | | | | | |
| 6 | | | | | | | | | | | | | |
| 8 | 225.1 | 162 | 149.2 | 218.4 | 158.8 | 163.3 | 137.1 | 159.4 | 133 | 178.9 | 166.7 | 185.3 | 178.8 |
| 10 | 172.1 | 133.3 | 113.6 | 159.4 | 136.5 | 117.5 | 100.4 | 120.2 | 102.1 | 140.4 | 120.4 | 146 | 138.7 |
| 12 | 162.3 | 113.1 | 91.3 | 133.1 | 110.7 | 102.5 | 92.09 | 108.8 | 89.22 | 118.2 | 109.9 | 126.7 | 115.2 |
| 14 | 75.89 | 103.2 | 66.9 | 124.4 | 101.4 | 108.3 | 99.27 | 114.7 | 78.7 | 132.6 | 106.4 | 123 | 133.3 |
| 16 | 159.6 | 126.4 | 98.09 | 114.2 | 101.3 | 95.81 | 86.18 | 99.46 | 106.7 | 110.8 | 106.2 | 118.3 | 115.4 |
| 18 | 123 | 114.6 | 90.09 | 125.3 | 104.5 | 101.6 | 80.76 | 100.9 | 79.23 | 103.1 | 103.6 | 130.9 | 137 |
| 20 | 171.9 | *9999* | 93.08 | 67.93 | 132.2 | 106.5 | 94.69 | 124.2 | *9999* | 135.5 | 111.7 | 127.7 | *9999* |
| 22 | 167.6 | 157.7 | 96.98 | 160.7 | 94.08 | 88.22 | 84.49 | 107.2 | 92.55 | 140.6 | 102.6 | 131.8 | 145.8 |
| 24 | 149.8 | 141.3 | 88.1 | 149.8 | 83.38 | 135.8 | 24.59 | 1.97 | 107.6 | 131.6 | 98.65 | 136 | 111.6 |

| **TRM** | | | | | | | | | | | | | | | |
|---|---|---|---|---|---|---|---|---|---|---|---|---|---|---|---|
| ZT | | | | | | | | | | | | | | | |
| 2 | | | | | | | | | | | | | | | |
| 4 | | | | | | | | | | | | | | | |
| 6 | | | | | | | | | | | | | | | |
| 8 | 167.3 | 237 | 191.4 | 177.6 | 223.7 | 304.9 | 174.8 | 153.7 | 169.9 | 168.7 | 271.8 | 169 | 181.9 | 117.3 | 187.7 |
| 10 | 113.7 | 169.4 | 129.5 | 142.2 | 154.4 | 264.2 | 90.03 | 115.8 | 116.5 | 125 | 196.4 | 128.3 | 125.8 | 92.88 | 148.1 |
| 12 | 114 | 153.3 | 112 | 112.6 | 125.3 | 232.5 | 83.5 | 93.45 | 103.4 | 105.7 | 161.5 | 111.6 | 104 | 85.43 | 120.9 |
| 14 | 111.9 | 132.5 | 106.8 | 117.8 | 159.5 | 199.3 | 79.9 | 109.1 | 112 | 125.5 | 157.3 | *9999* | 105.9 | 109 | 101.3 |
| 16 | 110.4 | 133.5 | 102.4 | 102.9 | 169.1 | 229.5 | 76.46 | 95.4 | 101.4 | 109.3 | 179.7 | 134.6 | 106.6 | 90.18 | 116.3 |
| 18 | 113 | 131.1 | 125.1 | 118.3 | 158.7 | 188.5 | 83.2 | 96 | 97.5 | 147.1 | 166.3 | 141.8 | 102.3 | 81.97 | 105.9 |
| 20 | 106.7 | 129.9 | 127 | 101 | 134.3 | 267.2 | 75.03 | 94.61 | 99.03 | 112 | 139.8 | 116.1 | 127.6 | 106.5 | 125.2 |
| 22 | 108.4 | 149.9 | 114.2 | 103.7 | 122.6 | 215.2 | 66.96 | 83.97 | 83.64 | 103.3 | 181.5 | 119.5 | 102.5 | 104.1 | 106.7 |

| | | | | | | | | | | | | | | | |
|---|---|---|---|---|---|---|---|---|---|---|---|---|---|---|---|
| 24 | 116 | 146.8 | 115.1 | 116.5 | 127.2 | 196.6 | 70.81 | 73.86 | 91.85 | 93.01 | 157.6 | 95.68 | 107.6 | 110.1 | 85.73 |

# Plasma corticosterone levels

Plasma corticosterone levels (µg/dL) before the restraining stress test (T0) and after stress test (T1)

| CLF T0 | CLF T1 | TRF T0 | TRF T1 |
|---|---|---|---|
| 15.19369 | 21.56 | 16.30694 | 34.05044 |
| 23.94519 | 30.734 | 14.54556 | 16.88298 |
| 13.03457 | 17.72752 | 12.44826 | 18.63098 |
| 18.84027 | 25.55265 | 17.77612 | 19.05279 |
| 15.19889 | 21.57098 | 14.69461 | 26.03897 |
| 23.95912 | 30.75599 | 13.83056 | 44.29307 |
| 13.03799 | 17.73504 | 16.31314 | 34.07655 |
| 9.978192 | 19.49702 | 14.55022 | 16.8897 |
| 18.84886 | 25.56838 | 17.78368 | 19.06159 |

Plasma corticosterone increase at T1 with respect to T0 (µg/dL)

| CLF $T_1$-$T_0$ | TRF $T_1$-$T_0$ |
|---|---|
| 6.366 | 17.744 |
| 6.789 | 2.337 |
| 4.693 | 6.183 |
| 6.712 | 1.277 |
| 6.372 | 11.344 |
| 6.797 | 30.463 |
| 4.697 | 17.763 |
| 9.519 | 2.339 |
| 6.72 | 1.278 |

# Gene expression analysis

Relative mRNA expression of Pparα

| CLF | CLM | TRF | TRM |
|---|---|---|---|
| 1.466947 | 1.02894 | 1.135696 | 1.017487 |
| 1.387616 | 1.095679 | 1.039968 | 1.149517 |
| 0.89786 | 1.264077 | 0.567772 | 0.82247 |
| 1.086165 | 1.091205 | 1.158467 | 0.961096 |
| 1.662925 | 0.738086 | 1.165996 | 1.465132 |
| 1.703041 | 0.980057 | 0.857806 | 1.355961 |
| | 1.08792 | 1.062253 | |
| | 0.817132 | 0.935296 | |

Relative mRNA expression of Pparγ

| CLF | CLM | TRF | TRM |
|---|---|---|---|

| | | | |
|---|---|---|---|
| 1.144158 | 0.866536 | 1.19596 | 0.730582 |
| 1.148897 | 0.90181 | 0.891772 | 0.799423 |
| 1.263939 | 0.925244 | 1.082215 | 0.745524 |
| 1.706288 | 0.910375 | 1.266565 | 0.689815 |
| 1.661352 | 1.025752 | 1.183947 | 1.381328 |
| 1.732309 | 1.098133 | 1.134425 | 1.241402 |
| | 0.818599 | 0.866954 | |
| | | 0.877952 | |

Relative mRNA expression of KDM6α

| CLF | CLM | TRF | TRM |
|---|---|---|---|
| 1.757528 | 1.448445 | 1.626448 | 0.698771 |
| 1.990654 | 1.038494 | 1.608532 | 1.084163 |
| 1.704184 | 0.954859 | 1.454886 | 1.168013 |
| 1.90234 | 0.697168 | 2.186713 | 1.145299 |
| 2.337101 | 0.843286 | 2.223758 | 1.369382 |
| 2.213892 | 0.970455 | 1.958439 | 1.450523 |
| | 1.100517 | 1.469198 | |
| | 1.108843 | 1.785642 | |

Relative mRNA expression of Nr3c1

| CLF | CLM | TRF | TRM |
|---|---|---|---|
| 1.120876 | 1.465212 | 1.597744 | 1.608891 |
| 2.337189 | 1.312373 | 1.656077 | 2.153124 |
| 1.6264 | 1.404132 | 2.990002 | 1.590621 |
| 2.623315 | 0.842557 | 2.142528 | 2.408277 |
| 2.229134 | 1.332656 | 2.197384 | 2.311497 |
| 2.755274 | 1.131396 | 1.049318 | 2.433992 |
| 1.217928 | 0.544389 | 1.105981 | 1.414777 |
| | 0.535542 | | |

Relative mRNA expression of IL-6

| CLF | CLM | TRF | TRM |
|---|---|---|---|
| 1.107384 | 1.201062 | 1.622215 | 0.737486 |
| 1.201763 | 0.890378 | 2.054439 | 0.872304 |
| 0.938343 | 0.935717 | 2.015411 | 1.192646 |
| 1.948148 | 0.928917 | 3.258854 | 1.181978 |
| 1.274492 | 1.292668 | 2.831648 | 0.643134 |
| 1.311471 | 0.837735 | 1.898874 | 1.051617 |
| 1.179236 | 0.993448 | 1.596652 | |
| | | 1.040343 | |

Relative mRNA expression of IL-1β

| CLF | CLM | TRF | TRM |
|---|---|---|---|
| 1.912018 | 1.074798 | 1.717602 | 1.886702 |
| 1.263177 | 1.474074 | 1.674762 | 0.738805 |
| 1.711367 | 1.342994 | 3.559063 | 1.518156 |
| 1.717079 | 0.633359 | 1.428629 | 2.004622 |
| 1.066748 | 0.817919 | 1.069259 | 1.719677 |
| 2.031641 | 1.052855 | 2.299791 | 1.828548 |
| 1.932887 | 0.86169 | 3.59685 | 0.99784 |
| 2.359608 | | 2.834059 | 2.512303 |

Relative mRNA expression of TNFα

| CLF | CLM | TRF | TRM |
|---|---|---|---|
| 1.309707 | 1.302588 | 1.248306 | 2.251752 |
| 0.908424 | 1.292677 | 1.154123 | 1.153103 |
| 1.071408 | 1.176013 | 1.322383 | 1.050997 |
| 0.99335 | 1.062665 | 1.329418 | 2.079933 |
| 1.0681 | 1.035574 | 1.173939 | 1.509502 |
| 0.917335 | 1.05708 | 1.173094 | 0.879967 |
| 1.091891 | 0.712121 | | 1.409418 |
| 1.325207 | 0.609609 | | |

Relative mRNA expression of KDM5c

| CLF | CLM | TRF | TRM |
|---|---|---|---|
| 1.191046 | 0.935504 | 0.615828 | 0.996671 |
| 1.262049 | 1.097916 | 0.740606 | 1.287318 |
| 1.504648 | 0.656368 | 0.731353 | 1.331423 |
| 1.572718 | 0.932904 | 1.145613 | 0.989144 |
| 1.519918 | 1.207168 | 1.571828 | 0.653312 |
| 2.027032 | 0.767192 | 0.755157 | 0.65336 |
| 1.343308 | 1.429234 | 1.381439 | 1.219306 |
| | 1.201229 | 1.00088 | 1.046486 |

Relative mRNA expression of KDM6b

| CLF | CLM | TRF | TRM |
|---|---|---|---|
| 2.025278 | 1.145659 | 0.479663 | 1.187731 |
| 1.230342 | 0.985433 | 0.417718 | 0.905221 |
| 1.53788 | 0.828495 | 0.981724 | 1.191781 |
| 2.228559 | 1.195319 | 1.075037 | 1.602495 |
| 0.833698 | 1.247259 | 0.908712 | 1.068052 |
| 1.778358 | 0.838974 | 0.866339 | 0.653128 |
| 1.60542 | 0.849007 | 0.754985 | 1.296532 |
| | 1.006762 | 1.324532 | 0.91106 |
