# Supplementary material for: Long lasting effects of perinatal exposure to the Chlorpyrifos pesticide on sleep, breathing, and neuroinflammation in adult mice
Source: PLoS One. 2025 Aug 1;20(8):e0328581. doi: 10.1371/journal.pone.0328581 (PMC12316233; doi:10.1371/journal.pone.0328581)
Supplement: S2 File — (PDF) [file pone.0328581.s009.pdf]

Supplementary Methods

Behavioural tests

Mechanical allodynia test (von Frey test)

Mechanical allodynia is a painful sensation caused by innocuous stimuli like light touch. Mice were placed in test cages with a metal grid bottom at least 2 h prior to testing to allow habituation to a novel environment. Paw withdrawal latency to mechanical stimulation was assessed with an automated testing device consisting of a steel rod (2 mm) that was pushed against the plantar surface of the hind paw with an increasing force until the paw was withdrawn (Dynamic Plantar von Frey Aesthesiometer, Ugo Basile, Varese, Italy). This device automatically recorded the maximum force applied and the latency of hind paw withdrawal. A linear increase in force over time was applied for 10 s up to a force of 5 g, after which the force remained constant. A maximum time of 120 s was allowed to record the response. When the animal withdrew its hind paw, the mechanical stimulus was automatically removed [1]. Paw withdrawal latency and actual force at the time of paw withdrawal were calculated by averaging the results of 6 consecutive trials.

Y-maze spontaneous alternation

The Y-maze spontaneous alternation is a behavioural test to assess short-term memory in mice. The test was performed in a Y-shaped maze (Ugo Basile, Varese, Italy) with three grey plastic arms (35 x 8 cm) at a 120° angle from each other. The animal was entered at the centre of the maze and allowed to freely explore the three arms for 5 minutes. This behaviour is driven by the innate curiosity of rodents to explore previously unvisited areas. All four limbs were required to enter an arm for the entry to be considered valid. One alternation is defined as consecutive entries into 3 different arms. The maze was cleaned with 70% ethanol after each trial. The percentage of spontaneous alternations is defined as: (total alternations/total arm entries − 1) x 100.

Elevated plus maze (EPM)

The elevated plus maze assesses anxiety-related behaviour in rodent models of central nervous system disorders. The apparatus consisted of two open arms (30 × 8 cm), two enclosed arms of the same size, and a central area (8 × 8 cm) at 50 cm above the floor. At the beginning of the test, mice were placed in the central part of the maze facing one of the open arms. The number of entries made into open and closed arms and the time spent in open and closed arms were recorded over a 5-minute session. An arm entry was defined as all four paws into an arm. Anxiety is reflected by the mouse spending more time in the enclosed arms. The percentage of time spent and entries into the open arm, as well as the total number of entries, were compared [2].

Novel object recognition task (NOR)

The novel object recognition (NOR) test is a commonly used behavioral assay for the investigation of various aspects of learning and memory in mice. The NOR test was performed in an open-field arena (50 × 50 cm). The behaviour of the mice was monitored using a video camera placed above the centre of the arena. Test chambers were cleaned with 70% ethanol between tests of different mice. The task procedure consisted of three phases: habituation, familiarisation, and test phase. During habituation, each animal was allowed to explore the arena without any object for 20 minutes. The animal was then removed from the arena and placed in its home-cage. During the familiarisation phase, the animal was placed for 10 min in the open-field arena containing two identical sample objects. After 1 h, the test phase started, and the animal was entered for 10 minutes in the arena where one of the two objects was replaced with a novel object. Novel object preference was expressed as a preference ratio, which was calculated by dividing the amount of time spent exploring the novel object by the total amount of time spent in object exploration during the test session [3]. NOR analysis was based on the definition of an explorative behaviour as the time spent with the head oriented towards and within 2–3 cm of the object, and with the vibrissae moving. Data obtained during the 10

minutes of the test period are presented. An exploratory preference index for an object was calculated as the percentage of time spent exploring that object over the total time spent exploring the objects [4]. A preference index above 25% indicated the preference for an object.

Open-field (OF) test

The open-field (OF) test is a sensorimotor test used to determine a mouse exploratory behaviour. By measuring the mouse preference to stay close to the walls of the open field versus time spent in the centre of the arena, a gross assessment of anxiety-related behaviour can be made. Briefly, the OF apparatus consisted of a square arena measuring 50 cm × 50 cm, divided into two areas: the centre zone and the surrounding area. The OF apparatus was in a dimly illuminated and quiet animal behaviour testing room. Animals were introduced into the center of the arena one at a time and were allowed to explore for 10 minutes. The animal was then removed and immediately placed in its home cages. The arena was cleaned with a 70% ethanol solution and dried thoroughly between tests on different mice to eliminate any potential odour cues left by previous tested subject. Data are expressed as the number of entries and the cumulative time spent in the centre and periphery of the OF. Reduced locomotion and increased time spent close to the borders of the open field are consistent with increased anxiety.

Bibliography

[1] Campana G, Rimondini R. Mechanical nociception measurement in mice and rats with automated Von Frey equipment. Methods Mol Biol Clifton NJ 2015;1230:229–31. https://doi.org/10.1007/978-1-4939-1708-2_18.

[2] Caberlotto L, Rimondini R, Hansson A, Eriksson S, Heilig M. Corticotropin-releasing hormone (CRH) mRNA expression in rat central amygdala in cannabinoid tolerance and withdrawal: evidence for an allostatic shift? Neuropsychopharmacol Off Publ Am Coll Neuropsychopharmacol 2004;29:15–22. https://doi.org/10.1038/SJ.NPP.1300296.

[3] Hammond RS, Tull LE, Stackman RW. On the delay-dependent involvement of the hippocampus in object recognition memory. Neurobiol Learn Mem 2004;82:26–34. https://doi.org/10.1016/J.NLM.2004.03.005.

[4] Wang D, Noda Y, Zhou Y, Mouri A, Mizoguchi H, Nitta A, et al. The allosteric potentiation of nicotinic acetylcholine receptors by galantamine ameliorates the cognitive dysfunction in beta amyloid25-35 i.c.v.-injected mice: involvement of dopaminergic systems. Neuropsychopharmacol Off Publ Am Coll Neuropsychopharmacol 2007;32:1261–71. https://doi.org/10.1038/SJ.NPP.1301256.
